## [Peer Review File · Nature Communications]

Chiral, air stable, and reliable Pd(0) precatalysts applicable to asymmetric allylic alkylation chemistryReviewers' Comments:

Reviewer #1:

Remarks to the Author:

This report describes the synthesis, characterization and reactivity of a series of bench stable chiral Pd(0) precatalysts based on two of the important ligand classes used in Pd-catalyzed asymmetric allylation chemistry. Pd complexes with Trost-type and PHOX-type ligands can either be generated in situ, or isolated as single-component precatalysts, by taking advantage of rapid ligand substitution chemistry exhibited by the DMPDAB-Pd-MAH precursor.

These complexes have been fully characterized, including X-ray diffraction characterization of three examples, which permitting examination of their conformational characteristics in solution and the solid-state.

Direct observation of an intramolecular hydrogen bond between an amide N-H group and a C=O moiety of the coordinated maleic anhydride models a key interaction in the proposed mechanism for stereoselection with the Trost ligand class.

It is observed that Trost-type complexes 1-4 do not suffer from rapid decomposition via oxidation and that they can be handled as solids or even in solution under air. The authors applied these to nine distinct Pd-catalyzed asymmetric allylation reactions in order to establish the potential of these precatalysts.

In all cases, these new precatalysts appear to favorably compare with traditional catalytic systems, maintaining enantioselectivity, while simultaneously enabling lower catalyst loadings.

As the investigators describe, the 1:1 single-component nature of 1-6 yields a perfectly controlled Pd/L stoichiometry, and circumvents the need to ensure ligand metalation is complete prior to substrate addition.

To demonstrate the utility of these precatalysts, these were applied towards the enantioselective allylation of a hydantoin derivative at 0.2 mol% catalyst loading. The discovery and optimization of this new reaction was supported by microscale array-based experiments.

These single-component precatalysts appear to have attractive features of selectivity, activity, and stability that makes them attractive alternatives to other established in-situ catalyst generation procedures.

Overall, I think this work is well written and the conclusions are reasonable, based on the data presented. This also represents an area of important catalysis science of broad interest to the chemistry community.

I therefore support publication of the manuscript in Nature Communications.

Reviewer #2:

Remarks to the Author:

In this manuscript, Arseniyadis and Leitch applied their recently reported DMPDAB-Pd-MAH precatalyst for preparing several air-stable complexes for the AAA reaction. The complexes displayed improved stability under air and were successfully used as catalysts in several model reactions, demonstrating superiority over the general conditions that rely on the Pd₂(dba)₃/I system. The

authors nicely showed the significant advantages in cost, robustness, and operational simplicity of their catalysts. I think the new enantioselective allylation of hydantoins example will inspire other groups to use their chemistry in the future.

The paper is written well, yet some parts can be shorter. The SI is of high quality. Overall, this is an important study that will have a strong impact on the field of metal catalysis.

I recommend publishing after addressing my comments:

1. The reproducibility issue: "Instead, these reactions rely on in situ catalyst generation, often using prolonged pretreatment of an achiral Pd source (e.g. Pd₂(dba)₃ or [Pd(allyl)Cl]₂) with excess chiral ligand to ensure complete metalation. In some cases, this also requires heating for extended periods of time, rendering the process significantly more prone to reproducibility issues. Furthermore, the use of Pd₂(dba)₃ is itself a reproducibility concern, given the known issues of quality and stability associated with this compound, with (multiple) recrystallization required to ensure high purity" (page 2) This is a long-standing problem in the field and the instability of Pd₂(dba)₃ is often discussed. That being said, the authors don't provide any results regarding the reproducibility of their new catalysts. They should repeat a selected reaction under the general and new conditions at least 3 times for statistical validation (give the average yield and the standard deviation). The results and discussion should include a discussion regarding the reproducibility of their reactions.

2. The discussion regarding the ratios of the 1-endo and 1-exo isomers in different solvents is very interesting and well-performed. However, it is too long and part of it can be transferred to the SI (for example, the results of the exo/endo ratios in different solvent mixtures, Figure 5, 3 lines from the bottom).

Page 6, line 1: Give the IUPAC name of the solvent "CPME".

Page 6, line 5: What "CPCM" stands for?

3. Page 6, line 21: "indefinitely stable" – I think using the word indefinitely in scientific papers is inappropriate.

4. Page 7. It is unclear from the text if the authors performed the control experiments with the Pd₂(dba)₃/L out of the glove box. As a synthetic chemist, I am attracted to robust reactions that can be performed in the hood. The authors' claim that the AAA reactions can be performed efficiently in a simple lab setting becomes weaker if they don't point out that the control reactions were performed outside the glove box.

5. Page 7 – The results of the Pd-catalyzed asymmetric allylation of phthalimide (11) as a nitrogen-based nucleophile using racemic epoxide 12 are puzzling since the ee that is obtained when using the same ligand but different Pd sources are not identical. Is it a known phenomenon? If so, add references. If not, maybe a control experiment with the "inactive" [PNNP]Pd complex to see if it can catalyze a racemic background reaction will clarify this issue. The authors should explain this observation.

6. Page 11 "Precatalyst-enabled discovery and optimization of the enantioselective allylation of hydantoins" As a reader, I felt that this part is too long. While the first paragraph, which describes the importance of this reaction, is essential, the description of the four-round optimization process using the microscale array-based experiments is tedious and unnecessary. I suggest to transfer it to the SI. The authors should provide the final conditions and the large-scale experiment and finish the manuscript with these significant results.

In conclusion, I recommend publication after minor revisions.

Reviewer #3:

Remarks to the Author:

This manuscript describes the preparation, characterization, and utility of maleic anhydride-ligated Pd(0) precatalysts for allylic functionalization. In particular, Pd(0) precatalysts incorporating privileged Trost-type and phosphinooxazoline-type (PHOX) ligands are reported, and their reactivity established to comparable favorably to or exceed established in situ catalyst preparations that are most commonly encountered using benchmark malonate and phthalimide nucleophiles (allylic alkylation and amination, respectively), as well as in decarboxylative allylic alkylation of allyl enol carbonates and alkylation using silylketene acetals, which are quite probably the most commonly used modern variation of the Tsuji–Trost type reaction due to the ability to translate allyl enol carbonate and silylketene acetal geometries to the putative enolates. The latter variation here was first reported by Arseniyadis only recently. This not only demonstrates the efficacy and generality of the pre-catalysts themselves but immediately establishes them as competitive with the current state of the art in situ catalyst preparations across the breadth of contemporary Pd-catalyzed allylic functionalization reactions that one encounters every day in the highest impact chemistry journals.

This leads me to comment more specifically on the “need” for such precatalysts. I have worked in the Tsuji–Trost field for over twenty years and by far the most complex feature catalysis efficacy is the ‘black box’ of in situ catalyst preparation. Unfortunately, this is widely recognized as a problem in Pd-catalyzed cross coupling reactions (as demonstrated by the success and impact of Buchwald’s precatalysts) but is far less recognized in the Tsuji–Trost field (in my opinion this is because the broader community thinks it is more knowledgeable about Tsuji–Trost chemistry than it is). For example, in situ catalyst formation normally occurs by stirring the ligand of choice with Pd2dba3; there is often significant time required for the active catalyst to be formed, and even then, this is often not quantitative, and the speciation is difficult to quantify. This has all been established by detailed mechanistic studies of Fairlamb and Jutand, which establishes that dba is difficult to remove from Pd completely. This can be benign but in the worst case can be deleterious to catalysis. A standard approach is to use 4-methoxy dba which is more amenable to substitution but still suffers from the same issues – often at the most inopportune times. In short, there is a compelling and long-standing need for stable and readily prepared Pd(0) precatalysts that can be used directly in Pd-catalyzed allylic functionalization processes. These should avoid any catalyst induction period and eliminate the use of dba-type supporting ligands that can result in complex speciation and deleteriously effect catalysis. The study described here addresses these needs easily. I am excited to see where these well-defined pre-catalysts will result in reactivity being extended to new substrate patterns that sluggishly engaged in Pd(allyl)formation and cannot therefore be used.

Finally, I would like to commend on the high quality of the spectroscopic and structural studies, which, in combination, reveal modular design features through well-defined coordination chemistry. That the DAB–Pd–MAH precursor can be used directly in HT-type reactivity screening is a huge advantage and something that has been challenging so far using standard in situ preparations (due to variable induction periods, differing speciation, competing mono vs polynuclear active species).

It is regrettable that the magnitude of the problem of pre-catalyst understanding in Pd-catalyzed allylic functionalization is not better appreciated, which is a direct result of many researchers overvaluing their own knowledge and understanding of this area. I have no doubt that these precatalysts will have the same transformative effect for Pd-catalyzed allylic functionalization chemistry as the Buchwald (and other) precatalysts have had on Pd-catalyzed cross coupling reactions.

In conclusion, I have no hesitation whatsoever in recommending this excellent study for publication. (see below for one suggestion).

Suggestion:

This is perhaps a personal preference, but the “map” diagrams to the right hand side of the figures. These have regrettably become fashionable in publications, but they are utterly meaningless. They do

little to present quantitative data that one can use, and these should be removed, and the parent numerical data presented for the reader. If the authors are determined to use these, please (for the sake of accessibility and readability) move them to the SI.

Reviewer #1

This report describes the synthesis, characterization and reactivity of a series of bench stable chiral Pd(0) precatalysts based on two of the important ligand classes used in Pd-catalyzed asymmetric allylation chemistry. Pd complexes with Trost-type and PHOX-type ligands can either be generated in situ, or isolated as single-component precatalysts, by taking advantage of rapid ligand substitution chemistry exhibited by the DMPDAB-Pd-MAH precursor.

These complexes have been fully characterized, including X-ray diffraction characterization of three examples, which permitting examination of their conformational characteristics in solution and the solidstate.

Direct observation of an intramolecular hydrogen bond between an amide N–H group and a C=O moiety of the coordinated maleic anhydride models a key interaction in the proposed mechanism for stereoinduction with the Trost ligand class.

It is observed that Trost-type complexes 1-4 do not suffer from rapid decomposition via oxidation and that they can be handled as solids or even in solution under air. The authors applied these to nine distinct Pd-catalyzed asymmetric allylation reactions in order to establish the potential of these precatalysts.

In all cases, these new precatalysts appear to favorably compare with traditional catalytic systems, maintaining enantioselectivity, while simultaneously enabling lower catalyst loadings.

As the investigators describe, the 13 single-component nature of 1-6 yields a perfectly controlled Pd/L stoichiometry, and circumvents the need to ensure ligand metalation is complete prior to substrate addition.

To demonstrate the utility of these precatalysts, these were applied towards the enantioselective allylation of a hydantoin derivative at 0.2 mol% catalyst loading. The discovery and optimization of this new reaction was supported by microscale array-based experiments.

These single-component precatalysts appear to have attractive features of selectivity, activity, and stability that makes them attractive alternatives to other established in-situ catalyst generation procedures.

Overall, I think this work is well written and the conclusions are reasonable, based on the data presented. This also represents an area of important catalysis science of broad interest to the chemistry community.

I therefore support publication of the manuscript in *Nature Communications*.

We thank the reviewer for their positive comments regarding our work. No changes are requested.

Reviewer #2

In this manuscript, Arseniyadis and Leitch applied their recently reported DMPDAB–Pd–MAH precatalyst for preparing several air-stable complexes for the AAA reaction. The complexes displayed improved stability under air and were successfully used as catalysts in several model reactions, demonstrating superiority over the general conditions that rely on the Pd₂(dba)₃/I system. The authors nicely showed the significant advantages in cost, robustness, and operational simplicity of their catalysts. I think the new enantioselective allylation of hydantoins example will inspire other groups to use their chemistry in the future.

The paper is written well, yet some parts can be shorter. The SI is of high quality. Overall, this is an important study that will have a strong impact on the field of metal catalysis.

I recommend publishing after addressing my comments:

1. The reproducibility issue: “Instead, these reactions rely on *in situ* catalyst generation, often using prolonged pretreatment of an achiral Pd source (e.g. Pd₂(dba)₃ or [Pd(allyl)Cl]₂) with excess chiral ligand to ensure complete metalation. In some cases, this also requires heating for extended periods of time, rendering the process significantly more prone to reproducibility issues. Furthermore, the use of Pd₂(dba)₃ is itself a reproducibility concern, given the known issues of quality and stability associated with this compound, with (multiple) recrystallization required to ensure high purity” (Page 2) This is a long-standing problem in the field and the instability of Pd₂(dba)₃ is often discussed. That being said, the authors don’t provide any results regarding the reproducibility of their new catalysts. They should repeat a selected reaction under the general and new conditions at least 3 times for statistical validation (give the average yield and the standard deviation). The results and discussion should include a discussion regarding the reproducibility of their reactions.

Reviewer #2 is right, we should have mentioned and evidenced the reproducibility of the reactions. Most of the reactions were repeated twice and showed no noticeable discrepancies. That being said, we have now run a series of four reactions on hydantoin 22 by two different experimentalists (two reactions each) using the following conditions: 1 mol% of (S,S)-^{Ph}DACH-Pd-MAH, 1.5 equiv. of NaHMDS in THF at 0 °C. The results have been included in the SI (page S96).

2. The discussion regarding the ratios of the 1-endo and 1-exo isomers in different solvents is very interesting and well-performed. However, it is too long and part of it can be transferred to the SI (for example, the results of the exo/endo ratios in different solvent mixtures, Figure 5, 3 lines from the bottom).

This is a good point, and we are cognizant that an overly-long discussion of solution conformations may dilute the overall message of the paper. To make this section more succinct, we have moved the following text to the SI (within the section “Investigation of Complex 1 Conformer Ratios” that starts on page S76):

We have ruled out a monomer/dimer equilibrium as the source of these two components by observing no change to the peak area ratio of major to minor species at different initial concentrations of **1**. We have also established the interconversion of these two species by analyzing their molar ratio as a function of solvent composition in a DCM/THF mixture (Figure 3B). As the volume fraction of THF increases, the amount of the minor conformer decreases exponentially, converging to the 14:1 ratio observed in 100% THF.

We have also moved the following passage to the SI (same section as above):

To test this hypothesis, we examined the conformer ratio in 10:1 mixtures of DCM and other hydrogenbond-accepting solvents (Figure S112). While a 10:1 DCM/THF mixture gives a 65:35 ratio of **1-exo** to **1-endo**, 10:1 DCM/DMF and 10:1 DCM/MeOH mixtures give an 80:20 **1-exo** to **1-endo** ratio. Addition of weakly hydrogen-bond-accepting solvents such as cyclopentylmethyl ether (CPME) and NEt₃ results in no change to the conformer ratio relative to that observed in DCM.

Finally, we have removed the solvent effect plot from Figure 3 (was Figure 3B) and made a single-column sized figure that only includes the ³¹P NMR spectra (Figure 3A), the schematic and calculated structures (now 3B), and the calculated electronic energies (now 3C).

Page 6, line 1: Give the IUPAC name of the solvent "CPME".

The IUPAC name of CMPE, cyclopentylmethyl ether, is now clearly indicated in the SI.

Page 6, line 5: What "CPCM" stands for?

It is an abbreviation for "conductor-like polarizable continuum", which is a common implicit model for solvent effects in computational chemistry. We have now defined it at its first use.

3. Page 6, line 21: "indefinitely stable" – I think using the word indefinitely in scientific papers is inappropriate.

We agree and it has been changed to "very stable".

4. Page 7: It is unclear from the text if the authors performed the control experiments with the Pd₂(dba)₃/L out of the glove box. As a synthetic chemist, I am attracted to robust reactions that can be performed in the hood. The authors' claim that the AAA reactions can be performed efficiently in a simple lab setting becomes weaker if they don't point out that the control reactions were performed outside the glove box.

We have added the statement "In every case, catalytic reactions were set up and executed without the use of a glovebox." to the first paragraph in the section "Deploying single component precatalysts in asymmetric allylic alkylations."

5. Page 7: The results of the Pd-catalyzed asymmetric allylation of phthalimide (**11**) as a nitrogen-based nucleophile using racemic epoxide **12** are puzzling since the ee that is obtained when using the same ligand but different Pd sources are not identical. Is it a known phenomenon? If so, add references. If not, maybe a control experiment with the "inactive" [PNNP]Pd complex to see if it can catalyze a racemic background reaction will clarify this issue. The authors should explain this observation.

This is a good point. We have re-examined the original reports of this reaction, and have an explanation for the observed enantioselectivity differences. Since [PNNP]Pd^{II} has previously been shown to be inactive in other AAA reactions, we do not think a racemic background reaction is possible. However, catalyst decomposition to [PNNP]Pd^{II} is absolutely a potential source of lowered enantioselectivity for a different reason: this reaction is reported to have "significantly decreased ... ee" with catalyst loadings below 0.4 mol% (refs 70 and 72). Furthermore, ^{NAP}DACH–Pd–dba is known to convert spontaneously to [PNNP]Pd^{II} even in the absence of dioxygen (ref 62). Thus, using the other Pd sources (especially Pd₂dba₃•CHCl₃) means lower concentration of active Pd⁰ is likely, which is known to reduce enantioselectivity. Accordingly, we have added the following passage to the text (pg. 7):

Using [Pd(allyl)Cl]₂ or Pd₂(dba)₃•CHCl₃ as the Pd source with ^{NAP}DACH (**L2**) as the ligand (0.8 mol% Pd, 1:1.5 ratio of Pd/L2), we obtained good yields of the homoallyl alcohol **13** (99% and 82% respectively); however, we were not able to achieve the reported level of enantioselectivity even after rigorous purification of every component of the reaction mixture (72% and 64% versus the reported 96%). The reduced enantioselectivities observed for these Pd sources, especially when using Pd₂dba₃•CHCl₃, may be due to a lower effective catalyst concentration due to oxidative decomposition to [PNNP]Pd^{II}. Trost *et al.* reported that the enantioselectivity of this reaction is sensitive to catalyst loading, such that "lowering the catalyst further [below 0.4 mol%] significantly decreased the ee."^{70,72} Amatore *et al.* reported that **L2**–Pd–dba

converts to the corresponding [PNNP]Pd^{II} even in the absence of dioxygen. Notably, the single-component precatalyst ^{NAP}DACH-Pd-MAH (**2**) achieves excellent yield while attaining higher enantioselectivity than the *in situ* systems, without the need for excess **L2** or preactivation (99% yield, 81% *ee*). This result highlights the robustness of our Pd(0) precatalysts, which are effective even for reactions that are clearly sensitive to the specific reaction conditions.

(underlined passage is new)

Also, we have added an explicit statement in the introduction: "Thus, Pd(0) complexes of the Trost ligands were considered unstable and therefore unsuitable as precatalysts, as the [PNNP]Pd^{II} species is known to be catalytically inactive."⁶³ (underlined statement is new). The authors of ref. 63 explicitly show through multiple experiments that this Pd^{II} species is not capable of activating allylic electrophiles.

6. Page 11: "Precatalyst-enabled discovery and optimization of the enantioselective allylation of hydantoins" As a reader, I felt that this part is too long. While the first paragraph, which describes the importance of this reaction, is essential, the description of the four-round optimization process using the microscale array-based experiments is tedious and unnecessary. I suggest to transfer it to the SI. The authors should provide the final conditions and the large-scale experiment and finish the manuscript with these significant results.

*Here, we respectfully disagree with the reviewer's assessment of this section as unnecessary. While they are absolutely correct that the details of reaction optimization are not required to appreciate the final result, we have included this here to show – in a general sense – how to utilize the combination of ^{DMP}DAB-Pd-MAH and the L-Pd-MAH complexes to rapidly screen and optimize AAA reactions. Reviewer 3 (see below, bolded passage) specifically comments on the benefits of enabling this type of reactivity, which is challenging/less reliable using standard *in situ* protocols. Therefore, we have maintained this aspect of the discussion.*

That being said, upon re-reading this part, we do agree that some of the description is too detailed for the main text. We have converted these three paragraphs into one, with several statements pertaining to specific reaction conditions removed (all of this information is also in the SI).

In conclusion, I recommend publication after minor revisions.

Thank you for your constructive comments – they have definitely improved the quality of the manuscript.

Reviewer #3

This manuscript describes the preparation, characterization, and utility of maleic anhydride-ligated Pd(0) precatalysts for allylic functionalization. In particular, Pd(0) precatalysts incorporating privileged Trost-type and phosphinoxazoline-type (PHOX) ligands are reported, and their reactivity established to be comparable favorably to or exceed established *in situ* catalyst preparations that are most commonly encountered using benchmark malonate and phthalimide nucleophiles (allylic alkylation and amination, respectively), as well as in decarboxylative allylic alkylation of allyl enol carbonates and alkylation using silylketene acetals, which are quite

probably the most commonly used modern variation of the Tsuji–Trost type reaction due to the ability to translate allyl enol carbonate and silylketene acetal geometries to the putative enolates. The latter variation here was first reported by Arseniyadis only recently. This not only demonstrates the efficacy and generality of the pre-catalysts themselves but immediately establishes them as competitive with the current state of the art in situ catalyst preparations across the breadth of contemporary Pd-catalyzed allylic functionalization reactions that one encounters every day in the highest impact chemistry journals.

This leads me to comment more specifically on the “need” for such precatalysts. I have worked in the Tsuji–Trost field for over twenty years and by far the most complex feature catalysis efficacy is the ‘black box’ of in situ catalyst preparation. Unfortunately, this is widely recognized as a problem in Pd-catalyzed cross coupling reactions (as demonstrated by the success and impact of Buchwald’s precatalysts) but is far less recognized in the Tsuji–Trost field (in my opinion this is because the broader community thinks it is more knowledgeable about Tsuji–Trost chemistry than it is). For example, in situ catalyst formation normally occurs by stirring the ligand of choice with Pd₂dba₃; there is often significant time required for the active catalyst to be formed, and even then, this is often not quantitative, and the speciation is difficult to quantify. This has all been established by detailed mechanistic studies of Fairlamb and Jutand, which establishes that dba is difficult to remove from Pd completely. This can be benign but in the worst case can be deleterious to catalysis. A standard approach is to use 4-methoxy dba which is more amenable to substitution but still suffers from the same issues – often at the most inopportune times. In short, there is a compelling and long-standing need for stable and readily prepared Pd(0) precatalysts that can be used directly in Pd-catalyzed allylic functionalization processes. These should avoid any catalyst induction period and eliminate the use of dba-type supporting ligands that can result in complex speciation and deleteriously effect catalysis. The study described here addresses these needs easily. I am excited to see where these well-defined pre-catalysts will result in reactivity being extended to new substrate patterns that sluggishly engaged in Pd(allyl)formation and cannot therefore be used.

Finally, I would like to commend on the high quality of the spectroscopic and structural studies, which, in combination, reveal modular design features through well-defined coordination chemistry. **That the DAB–Pd–MAH precursor can be used directly in HT-type reactivity screening is a huge advantage and something that has been challenging so far using standard in situ preparations (due to variable induction periods, differing speciation, competing mono vs polynuclear active species).**

It is regrettable that the magnitude of the problem of pre-catalyst understanding in Pd-catalyzed allylic functionalization is not better appreciated, which is a direct result of many researchers overvaluing their own knowledge and understanding of this area. I have no doubt that these precatalysts will have the same transformative effect for Pd-catalyzed allylic functionalization chemistry as the Buchwald (and other) precatalysts have had on Pd-catalyzed cross coupling reactions.

In conclusion, I have no hesitation whatsoever in recommending this excellent study for publication. (see below for one suggestion).

Suggestion:

This is perhaps a personal preference, but the “map” diagrams to the right hand side of the figures. These have regrettably become fashionable in publications, but they are utterly meaningless. They do little to present

quantitative data that one can use, and these should be removed, and the parent numerical data presented for the reader. If the authors are determined to use these, please (for the sake of accessibility and readability) move them to the SI.

We appreciate the reviewer's position that radar plots are not well-suited to presenting specific quantitative data. They are, however, a way to visualize multivariate data in a more holistic way, especially (as in our case) to compare different situations. Our intent with these plots is to show how the single component L-Pd-MAH precatalysts compare to in situ systems across multiple measures of effectiveness. The source data for these plots are all included in the colour-coded boxes in the figures.

This being said, there is one aspect of the radar plots that we have changed, which is the qualitative "practicality" measure, which at the moment is on an arbitrary scale (due to its qualitative nature). We have changed this to "L/Pd premixing time" to be on a quantitative scale.

Thank you all again for your constructive comments, and for your consideration of our revised manuscript.

Reviewers' Comments:

Reviewer #1:

Remarks to the Author:

The authors have appeared to take the reviewers' comments to heart, and have adjusted the manuscript accordingly, including moving some items to the SI section for reasons of brevity.

I think this is reflected in an overall better and more readable paper, and I recommended that it be published as is.

Reviewer #2:

Remarks to the Author:

[Note from the Editor: Reviewer #2 made comments to the Editor only, is satisfied with the revision and recommends publication.]

Reviewer #3:

Remarks to the Author:

I approve the revised version of the manuscript.
Congratulations to the authors.