# Supplementary material for: Chiral, air stable, and reliable Pd(0) precatalysts applicable to asymmetric allylic alkylation chemistry
Source: Nat Commun. 2023 Dec 5;14:8058. doi: 10.1038/s41467-023-43512-8 (PMC10698162; doi:10.1038/s41467-023-43512-8)
Supplement: Supplementary file 1 — Supplementary Information [file 41467_2023_43512_MOESM1_ESM.pdf]

Supplementary Information

Chiral, air stable, and reliable Pd(0) precatalysts applicable to asymmetric allylic alkylation chemistry

Jingjun Huang,†,a Thomas Keenan,†,b Francois Richard,[b] Jingru Lu,[a] Sarah E. Jenny,[c] Alexandre Jean,[d] Stellios Arseniyadis,*,b and David C. Leitch*,a

[a] University of Victoria, Department of Chemistry, 3800 Finnerty Road, Victoria, BC V8P 5C2, Canada
[b] Queen Mary University of London, Department of Chemistry, Mile End Road, London E1 4NS, UK
[c] Temple University, Department of Chemistry, 1901 N. Broad St, Philadelphia, PA 19122, USA
[d]Industrial Research Centre, Oril Industrie, 13 rue Desgenétais, 76210, Bolbec, France
†These authors contributed equally.

Table of Contents

General Considerations **S3**
Synthesis and Characterization of Palladium Complexes **S4**
(*S,S*)-PhDACH-Pd-MAH (**1**) S4
(*S,S*)-NAPDACH-Pd-MAH (**2**) S16
VT NMR Spectroscopy of **2** S25
(*S,S*)-PhSTIL (**L3**) S26
(*S,S*)-PhSTIL-Pd-MAH (**3**) S29
(*S,S*)-PhANDEN-Pd-MAH (**4**) S35
(*S*)-*t*BuPHOX (L**5**) S47
(*S*)-*t*BuPHOX-Pd-MAH (**5**) S47
(*S*)-*i*PrPHOX-Pd-MAH (**6**) S59
***In Situ* Metalation Analysis** **S68**
Solution Stability of Precatalysts **S71**
Stability of (*S,S*)-PhDACH-Pd-MAH (**1**) in air S75
Stability of (*S,S*)-PhDACH-Pd-dba in N_2 and air S76
Investigation of Complex 1 Conformer Ratios **S79**
Spectroscopic Studies S79
Computational Details S81

Catalytic Asymmetric Allylic Alkylation Reactions **S82**
DYKAT malonation of cyclohex-2-en-1-yl methyl carbonate (**7**) S82
Desymmetrisation of *meso bis*(acetate) **9** S81
Allylic amination of butadiene monoxide (**12**) with phthalimide (**11**).......... S86
Decarboxylative allylation of enol carboxylate **14** S87
PHOX-Pd catalyzed allylation of dimethyl malonate using allylic acetate **16** S90
Improved Pd-AAA of furanone derivatives S92
Direct approach.......... S91
Pd-DAAA approach.......... S91
Enol silane nucleophile approach S92
Allylation of hydantoin **22** S93
Parallel screening experiments S94
Optimization at low catalyst loading S99
Gram scale synthesis of **23** S99
Representative HPLC chromatograms for enantiopurity determination **S101**
X-ray Crystallographic Details **S108**
(*S,S*)-^{NAP}DACH-Pd-MAH (**2**) S105
(*S,S*)-^{Ph}ANDEN -Pd-MAH (**4**).......... S120
[PNNP(^{Ph}ANDEN)]-Pd^{II} (**4**[O]).......... S133
(*S*)-^{iPr}PHOX-Pd-MAH (**6**).......... S145
Supplementary References **S153**

General Considerations

Materials. All solvents and common organic reagents were purchased from commercial suppliers and used without further purification. ^{DMP}DAB-Pd-MAH was prepared according to literature procedures.[1a] Pd_2dba_3•$CHCl_3$ was prepared fresh, recrystallized, and assessed for purity by ^{1}H NMR spectroscopy according to literature procedures.[1b] (*S,S*)-^{Ph}DACH (**L1**), (*S,S*)-^{NAP}DACH (**L2**), and (*S*)-^{iPr}PHOX (**L6**) were purchased from Strem Chemicals and used as received. (*S,S*)-^{Ph}STIL (**L3**),[2] (*S,S*)-^{Ph}ANDEN (**L4**),[3] and (*S*)-^{tBu}PHOX (**L5**)[4] were prepared following literature procedures (adapted procedures for **L3** and **L5** are given below). Anhydrous solvents (SureSeal) were purchased from MilliporeSigma and used as received.

Techniques. All air-free manipulations in the preparation of palladium precatalysts **1**-**6** were performed under a dry nitrogen atmosphere using an MBraun glovebox. All air free manipulations for the catalytic evaluation and preparative-scale allylation reactions were performed under a dry nitrogen atmosphere using nitrogen-filled balloons. Analytical TLCs were performed with Merck silica gel plates pre-coated with silica gel 60 F254 (0.2 mm). Visualisation was effected by quenching of UV fluorescence (λ max = 254 nm or 360 nm) and by staining with p-anisaldehyde, potassium permanganate or vanillin TLC stain solutions, followed by heating. Flash column chromatography employed VWR (230–400 mesh) silica gel.

Analysis and Spectroscopy. All NMR spectra were acquired on a Bruker AVANCE 300 MHz spectrometer, a Bruker AVANCE 360 MHz, a Bruker AVANCE 400 MHz spectrometer, or a Bruker AVANCE Neo 500 MHz spectrometer. ^{1}H NMR spectra were calibrated to residual solvent peaks ($CHCl_3$ δ 7.26, methanol-d_4 δ 3.31, acetone-d_6 δ 2.05). Data are reported as follows: chemical shift in ppm, multiplicity (s = singlet, brs = broad singlet, d = doublet, t = triplet, q = quartet, m = multiplet or overlap of non equivalent resonances), coupling constants, and integration. ^{13}C NMR spectra were calibrated to residual solvent peaks ($CHCl_3$ δ 77.16, methanol-d_4 δ 49.00, acetone-d_6 δ 29.08). ^{31}P NMR chemical shifts are calibrated to external standards (85% H_3PO_4 at 0 ppm). All NMR spectroscopic data is processed using Bruker TopSpin 4.07 or Mestrenova. High-resolution electrospray ionization mass spectrometric analysis (HR-ESIMS) was performed using a Thermo Scientific Ultimate 3000 ESI-Orbitrap Exactive Plus. High performance liquid chromatography analysis for enantiopurity determinations was carried out on an Agilent 1100 system, using a mixture of *n*-hexane and isopropyl alcohol with Daicel CHIRALPAK columns (250 x 4.6 mm; 5 µm). Specific method details and retention times are given for each analysed compound. Optical rotations were measured with an Optical activity AA-100 polarimeter operating on the sodium D-line (589 nm), using a 50 mm path-length cell.

Synthesis and Characterization of Palladium Complexes

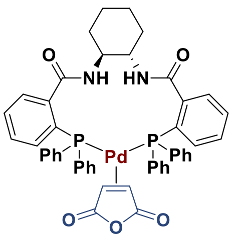

^{Ph}DACH-Pd-MAH

(*S,S*)-^{Ph}DACH-Pd-MAH (1) – A 4-dram vial was charged with ^{DMP}DAB-Pd-MAH (200.1 mg, 0.43 mmol), (*S,S*)-^{Ph}DACH (**L1**) (294.8 mg, 0.43 mmol), and 7 mL of anhydrous, inhibitor-free THF. The mixture was stirred at rt for two hours. THF was then evaporated under vacuum, followed by trituration/decantation cycles using a 1:1 hexane/Et_2O solution until the washings were colorless (6 times). The product was dried under vacuum to give a pale-yellow solid (339.0 mg, 89%).

^{1}H NMR (500 MHz, THF-d_8) δ 8.06 (d, *J* = 5.6 Hz, 1H), 7.82 (d, *J* = 6.3 Hz, 1H), 7.59-6.65 (m, 28H), 4.38 (m, 1H), 4.08 (m, 1H), 3.64 (m, 1H), 3.37 (sept, *J* = 5.1 Hz, 1H), 2.21 (m, 1H), 2.05 (m, 1H), 1.79 (m, 1H), 1.65-1.53 (m, 2H), 1.33-1.24 (m, 1H), 1.12-1.24 (m, 2H).

^{13}C NMR (125 MHz, THF-d_8) δ 170.6, 170.3, 169.0, 168.0, 143.7, 139.7, 137.7, 136.1, 134.2, 133.9, 133.1, 132.5, 133.0, 129.6, 129.5_4, 129.4_7, 129.4, 129.3_2, 129.2_8, 129.0_4, 129.0_0, 128.7_3, 128.6_9, 128.6_6, 128.2, 128.0, 127.9, 127.8, 127.4, 127.3, 127.0, 126.9, 57.0, 52.4, 32.1, 31.2, 24.8, 23.7.

^{31}P NMR (200 MHz, THF-d_8) δ 24.9 (d, $^2J_{P-P}$ = 4.9 Hz), 23.9 (d, $^2J_{P-P}$ = 4.8 Hz).

Two conformers were observed when **1** was dissolved in CD_2Cl_2.

^{1}H NMR (500 MHz, CD_2Cl_2) δ *Major conformer*: 7.86 (d, *J* = 5.1 Hz, 1H), 7.60-6.65 (m, 56H, Ar-H), 6.14 (d, *J* = 6.1 Hz, 1H), 4.44-4.39 (m, 1H), 3.99-3.92 (m, 1H), 3.71-3.65 (m, 1H), 3.38 (septet, *J* = 5.2 Hz, 1H), 2.28-2.24 (m, 1H), 2.12-2.08 (m, 2H), 2.02-1.99 (m, 1H), 1.82-1.76 (m, 2H), 1.67-1.63 (m, 2H), 1.45-1.12 (m, 6H), 1.16-1.10 (m, 2H). *Minor conformer:* 7.60-6.65 (m, 56H, Ar-H), 4.44-4.39 (m, 1H), 3.99-3.92 (m, 1H), 3.87-3.80 (m, 1H), 3.59-3.52 (m, 1H), 2.28-2.24 (m, 1H), 2.12-2.08 (m, 2H), 2.02-1.99 (m, 1H), 1.82-1.76 (m, 2H), 1.67-1.63 (m, 2H), 1.45-1.12 (m, 6H), 1.16-1.10 (m, 2H).

^{31}P NMR (200 MHz, CD_2Cl_2) δ *Major conformer*: 24.5 (d, $^2J_{P-P}$ = 7.1 Hz), 23.0 (d, $^2J_{P-P}$ = 7.1 Hz). *Minor conformer:* 26.5 (d, $^2J_{P-P}$ = 12.0 Hz), 22.1 (d, $^2J_{P-P}$ = 11.9 Hz).

HRMS (ESI): m/z calcd for $C_{48}H_{42}N_2O_5P_2Pd$, $[M + Na]^+$ (major isotopomer): 917.1496, found: 917.1510.

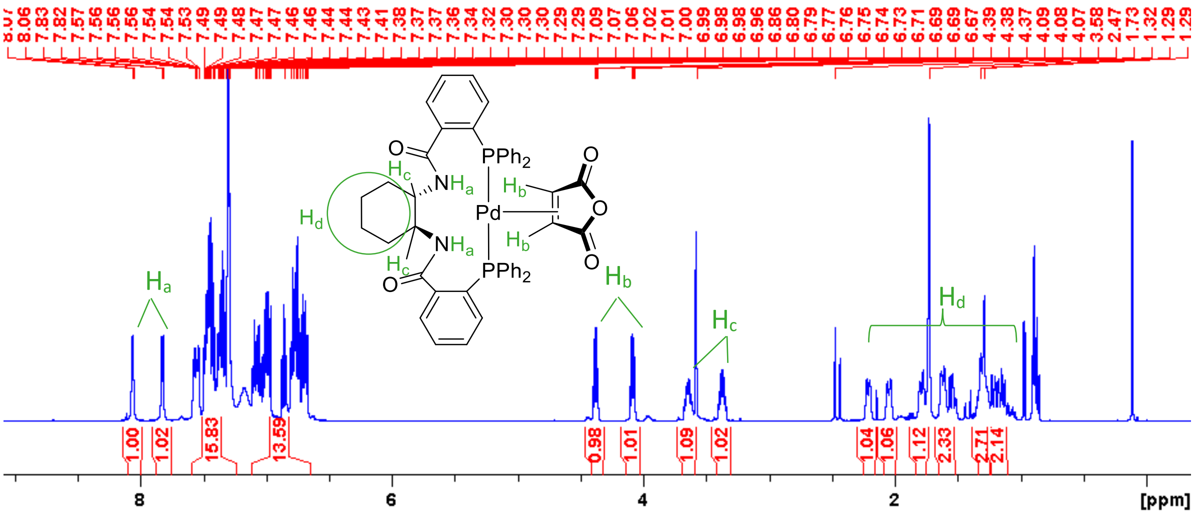

Supplementary Figure 1. ^{1}H NMR spectrum (500 MHz, THF-d_8) of **1**. Key proton signals are assigned. The singlets at 2.47 ppm and 2.43 ppm are H_2O and HDO. Multiplet at 0.85-0.90 ppm is hexanes. Singlets at 1.73 ppm and 3.58 ppm are THF-d_8 residual signals.

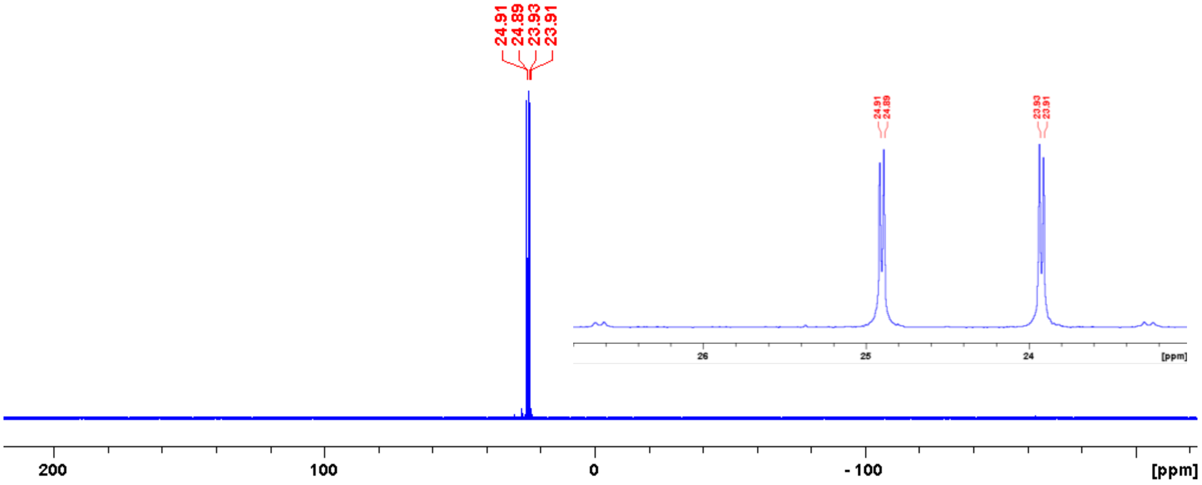

Supplementary Figure 2. $^{31}P\{^{1}H\}$ NMR spectrum (200 MHz, THF-d_8) of **1**.

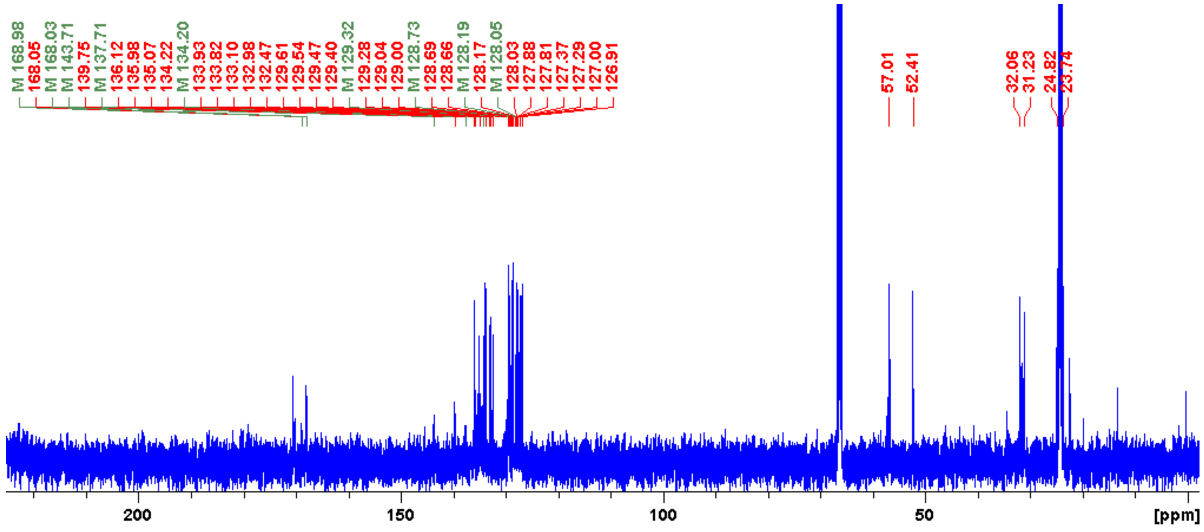

Supplementary Figure 3. $^{13}C\{^{1}H\}$ NMR spectrum (125 MHz, THF-d_8) of **1** (Green labels are manually-picked peaks).

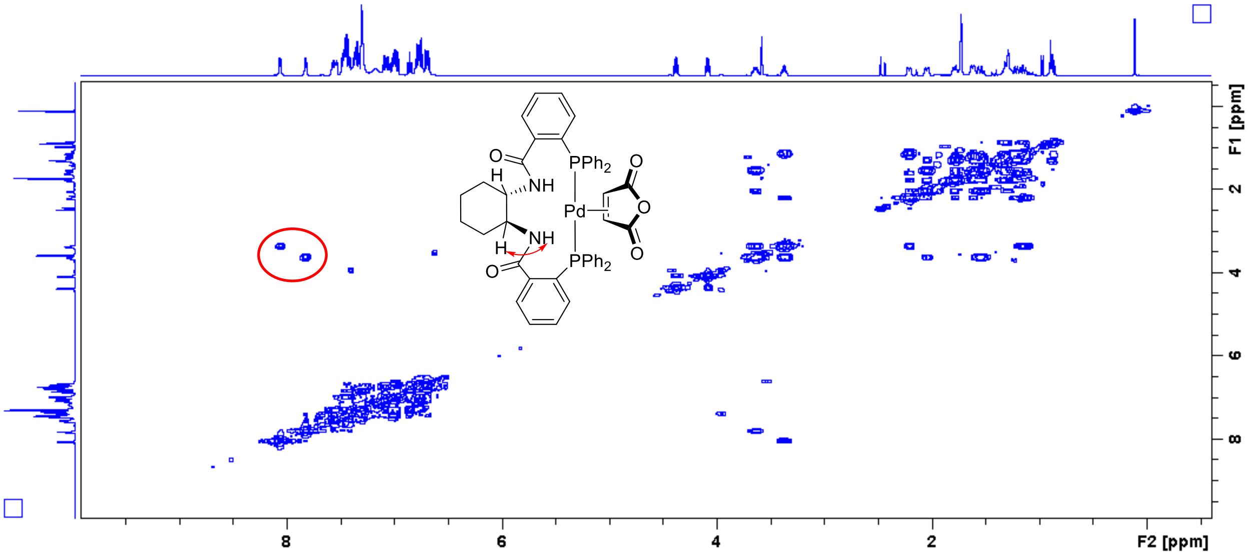

Supplementary Figure 4. ^{1}H-^{1}H COSY NMR spectrum of **1**. Confirmation of NH protons.

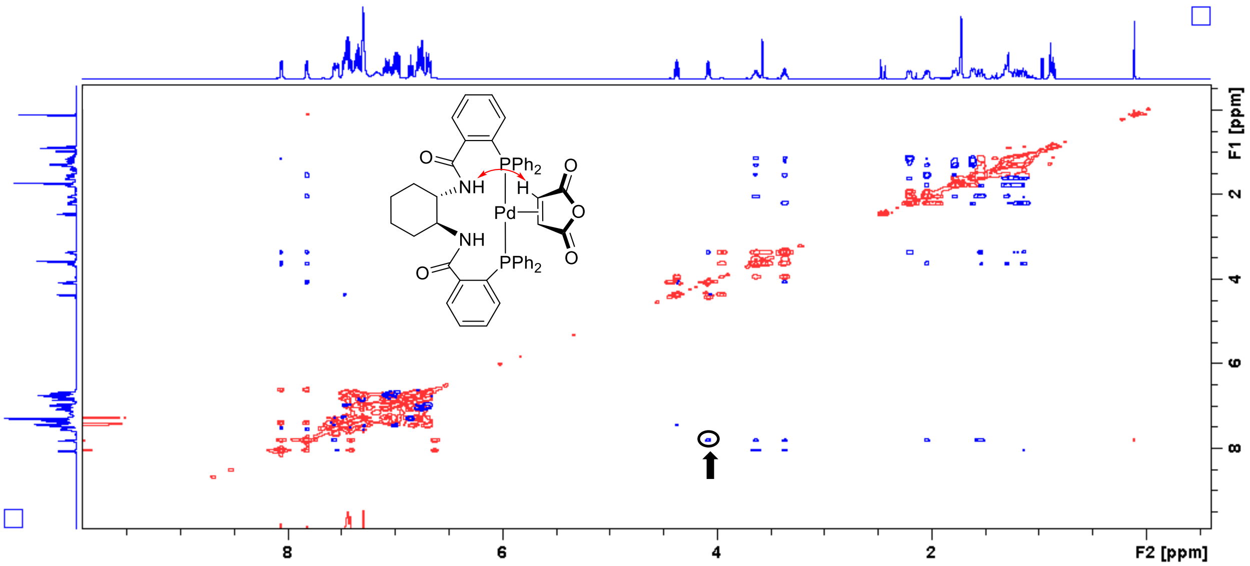

Supplementary Figure 5. ^{1}H-^{1}H NOESY NMR spectrum of **1**. The only correlation between NH and MAH-H indicates the conformer without hydrogen bonding.

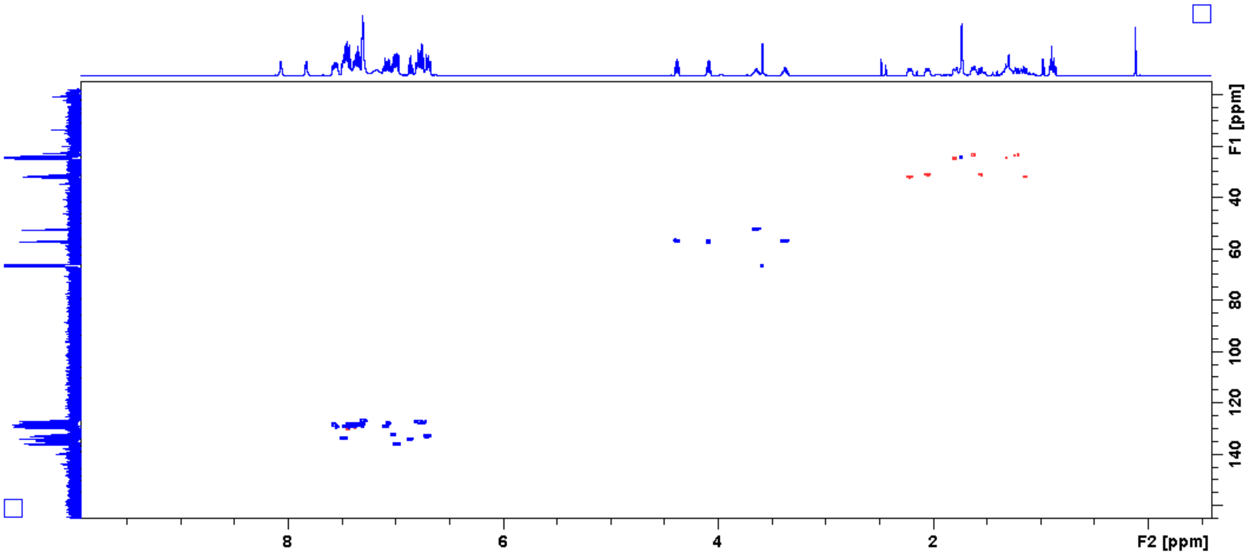

Supplementary Figure 6. ^{1}H-^{13}C HSQC NMR spectrum of **1**.

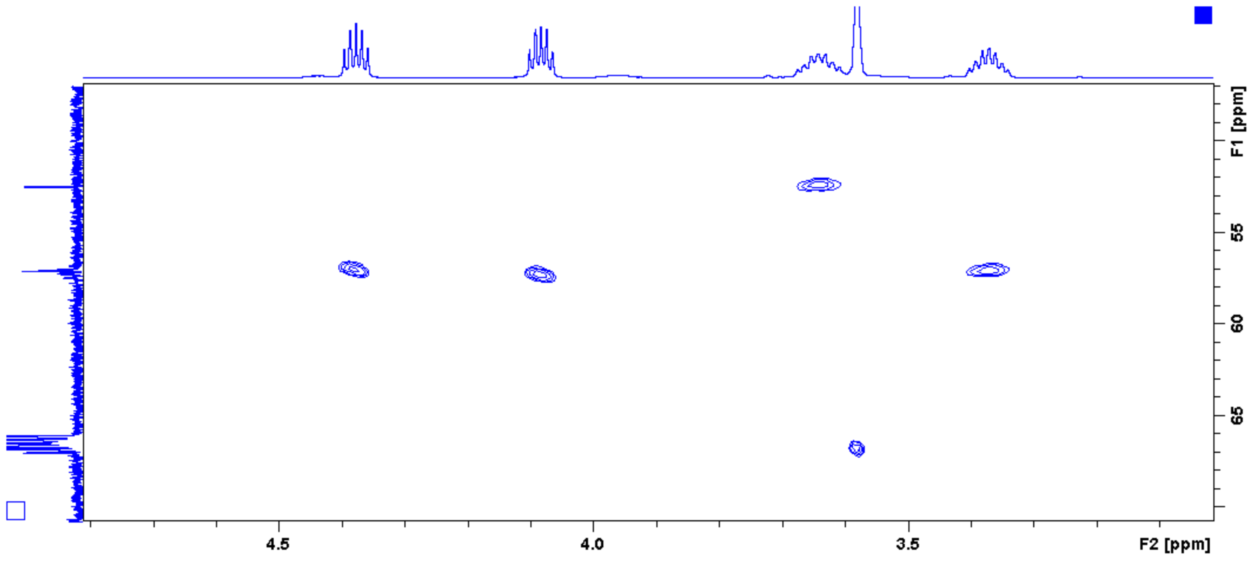

Supplementary Figure 7. ^{1}H-^{13}C HSQC NMR spectrum of **1**. Expansion of overlapped carbon peaks at 57.01 ppm.

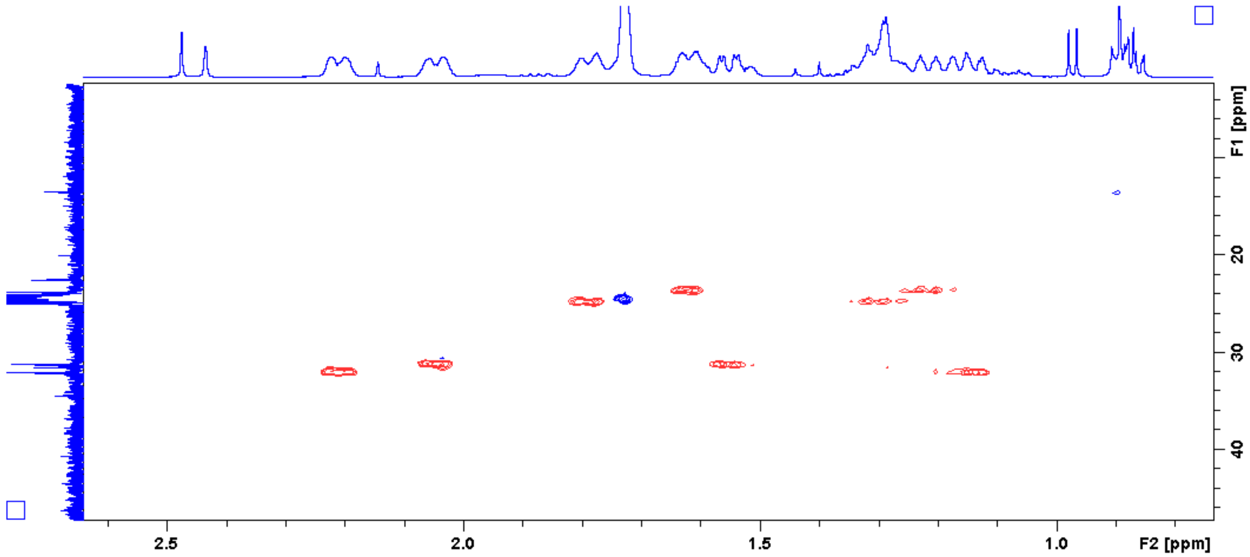

Supplementary Figure 8. ^{1}H-^{13}C HSQC NMR spectrum expansion of **1**. Confirmation of cyclohexyl protons.

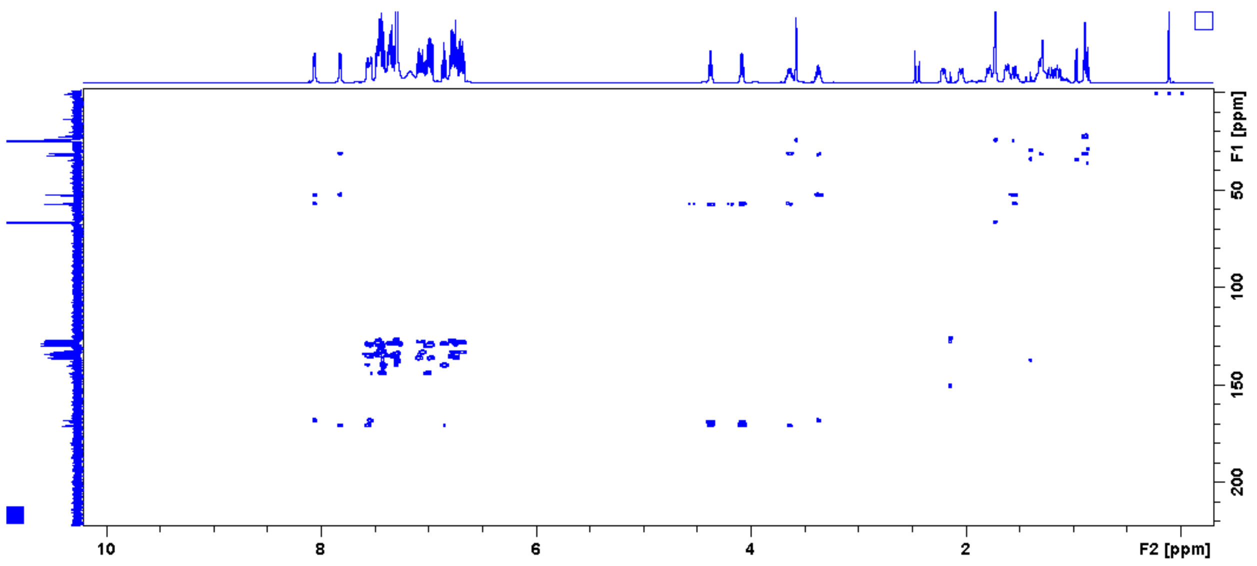

Supplementary Figure 9. ^1H-^{13}C HMBC NMR spectrum of **1**.

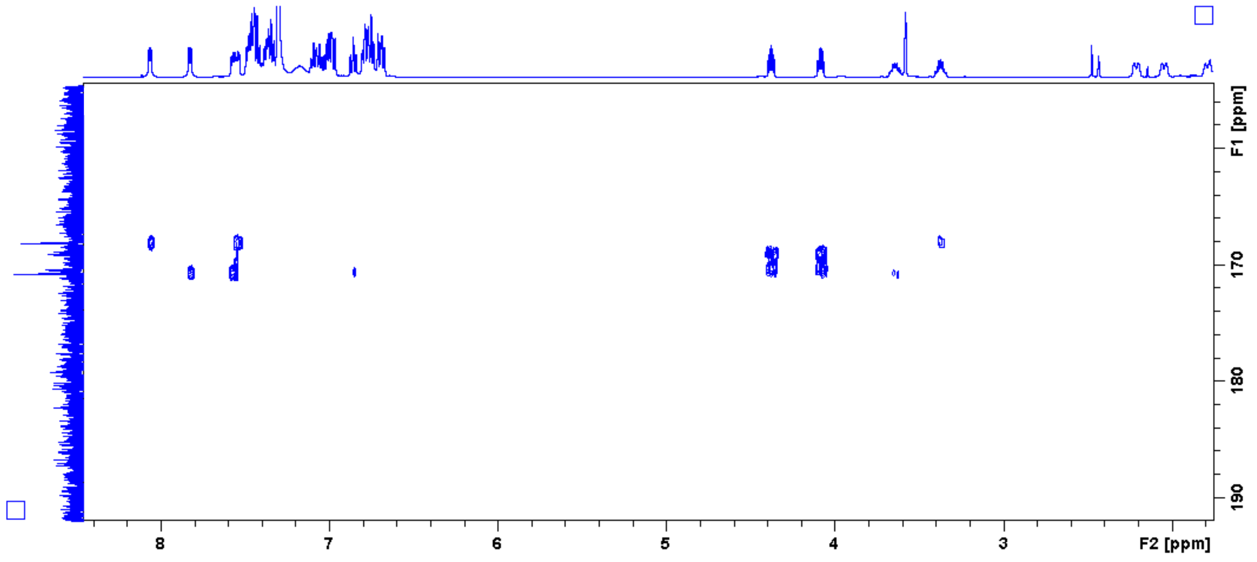

Supplementary Figure 10. ^1H-^{13}C HMBC NMR spectrum expansion of **1**. Identification of the carbonyl groups of MAH.

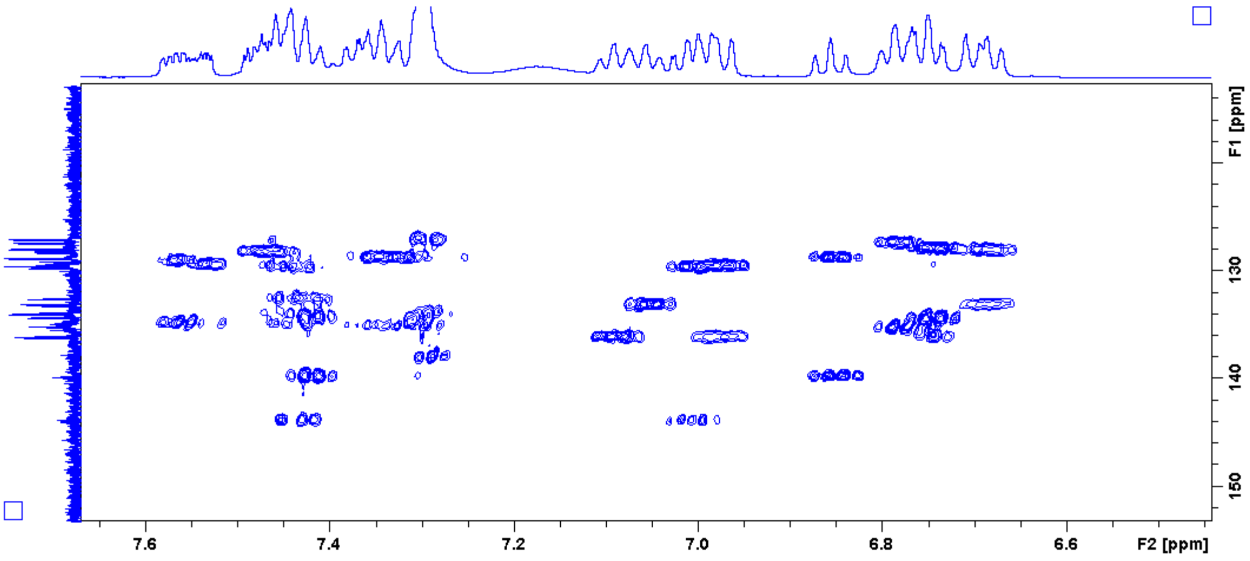

Supplementary Figure 11. ^{1}H-^{13}C HMBC NMR spectrum of **1**. Aliphatic region expansion. Compensation of carbon determination by ^{1}H-^{13}C HSQC NMR spectrum.

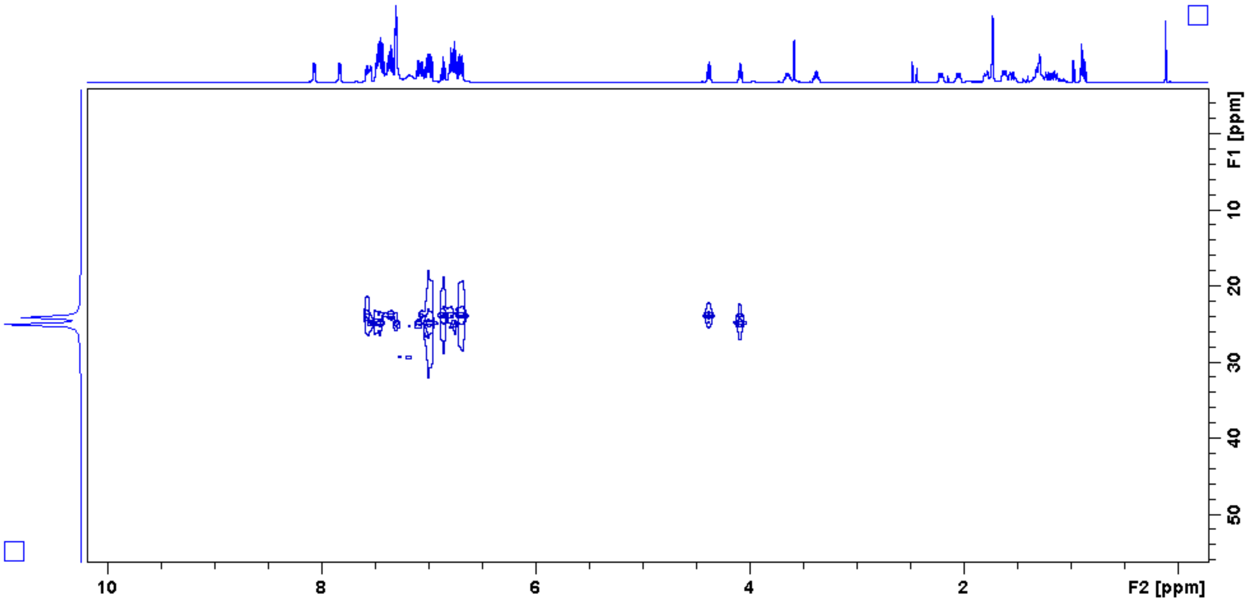

Supplementary Figure 12. ^{1}H-^{31}P HMBC NMR spectrum of **1**.

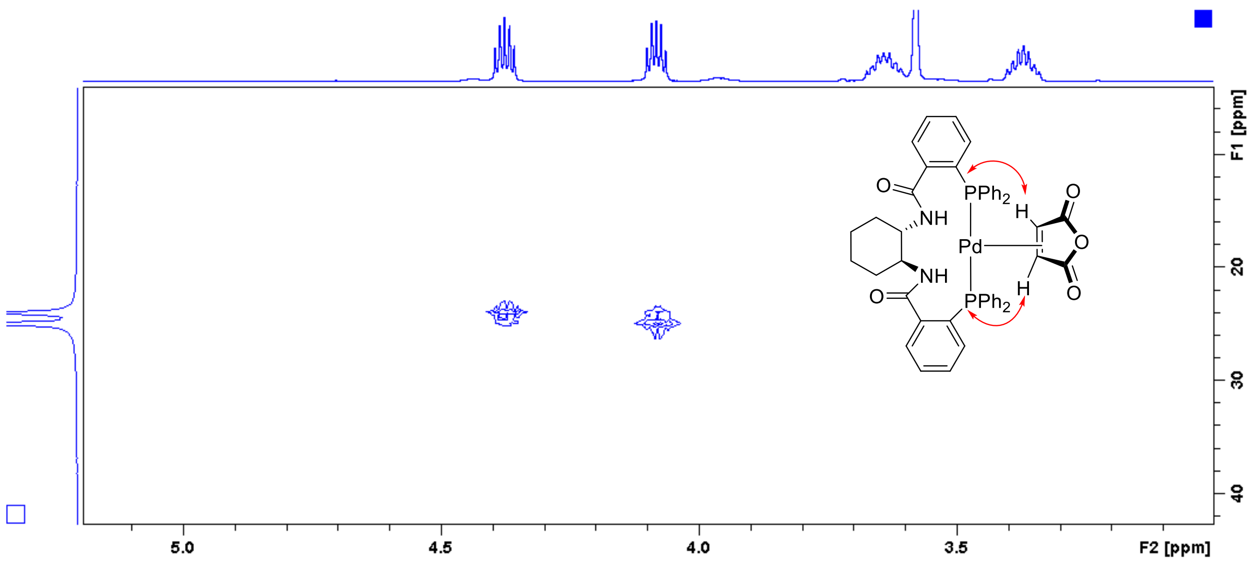

Supplementary Figure 13. ^{1}H-^{31}P HMBC NMR spectrum expansion of **1**.
Correlations between MAH protons and the two P atoms are observed.

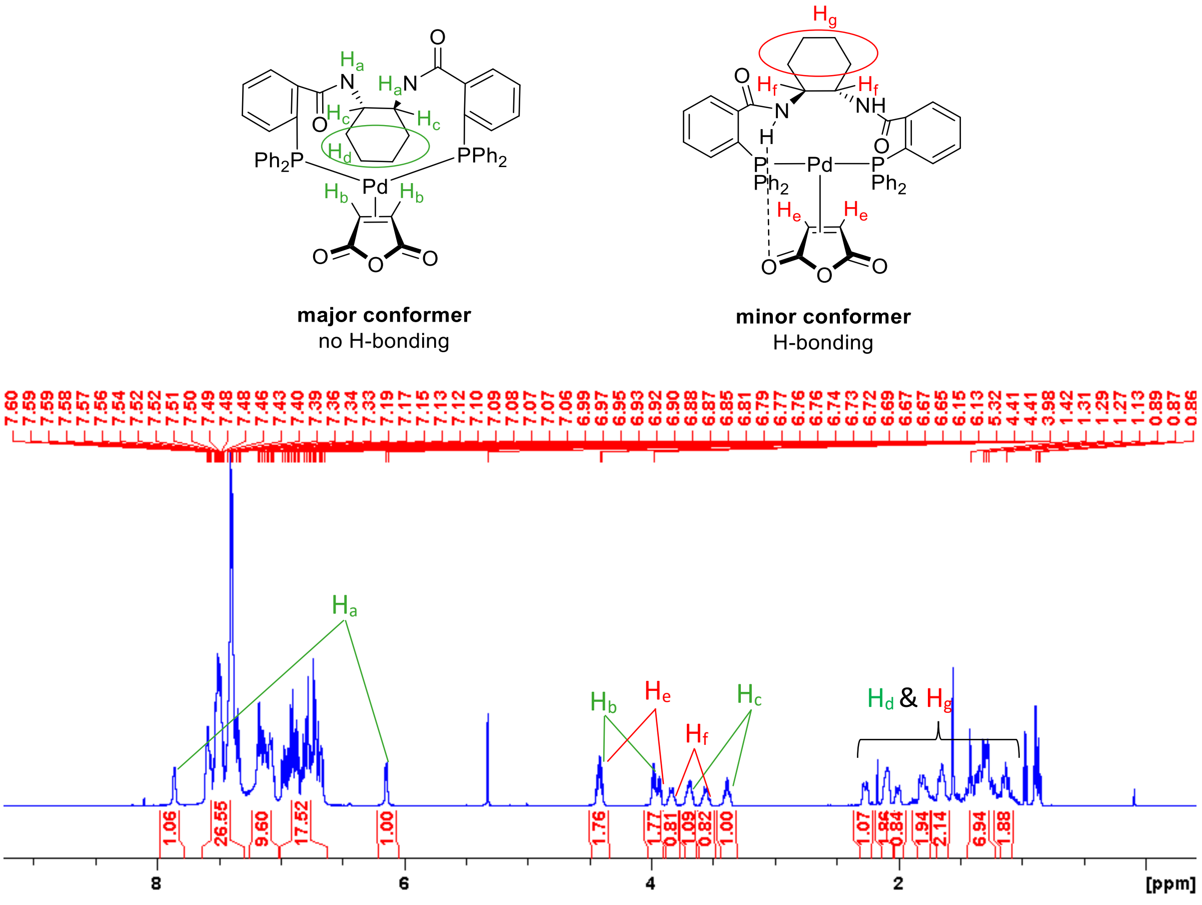

Supplementary Figure 14. ^{1}H NMR spectrum (500 MHz, CD_2Cl_2) of **1**. Singlet at 5.32 ppm is the CD_2Cl_2 residual signal.
Multiplet from 0.84 to 0.90 ppm is the signal of hexanes.

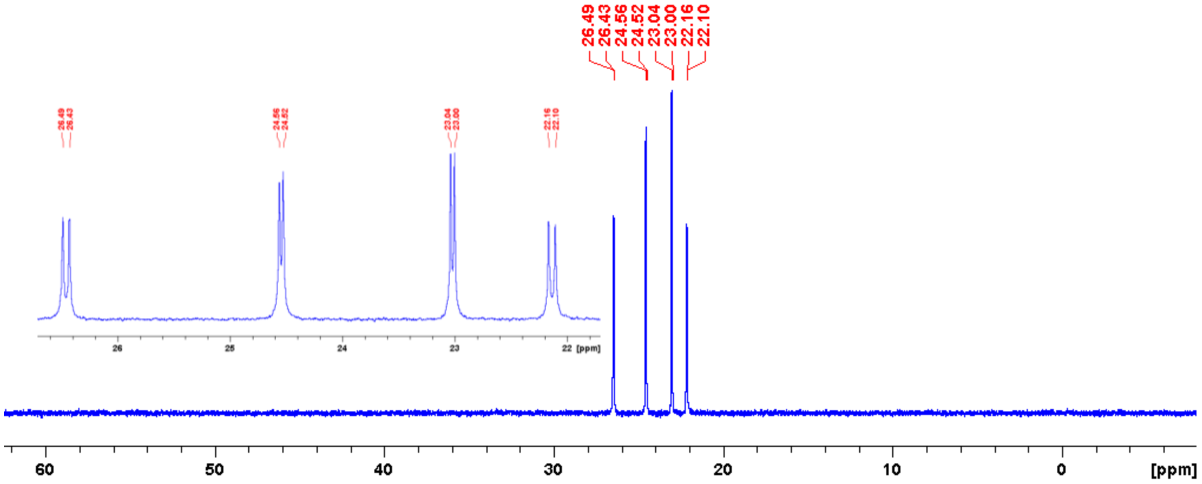

Supplementary Figure 15. $^{31}P\{^{1}H\}$ NMR spectrum (200 MHz, CD_2Cl_2) of **1**.

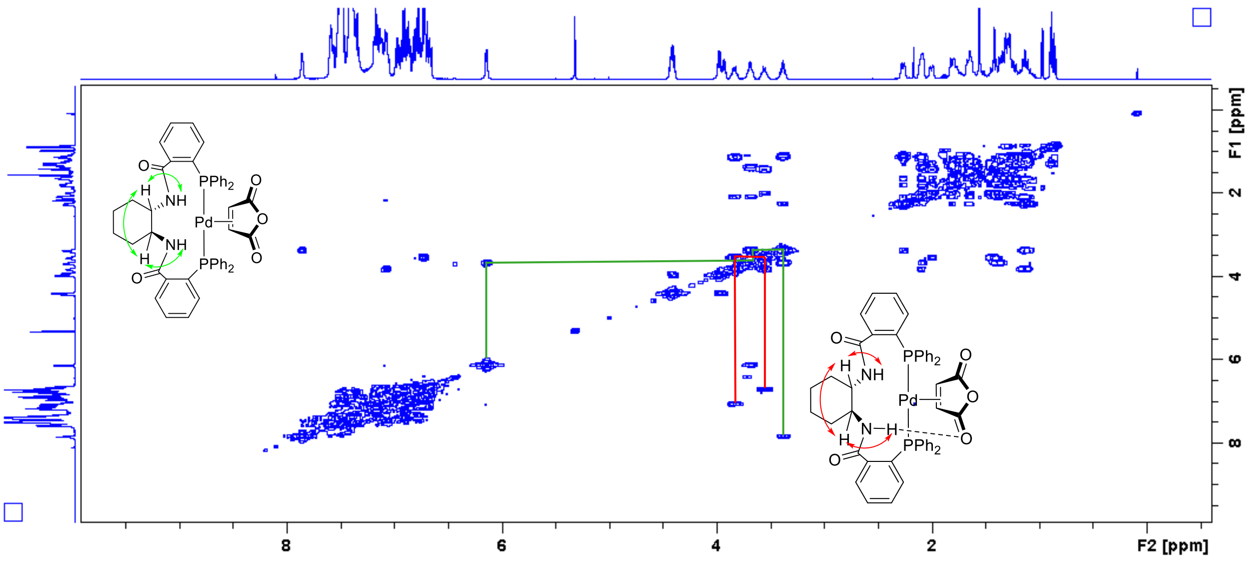

Supplementary Figure 16. ^1H-^1H COSY spectrum (CD_2Cl_2) of **1**. NH identification of major conformer and minor conformer (green: major conformer, red: minor conformer).

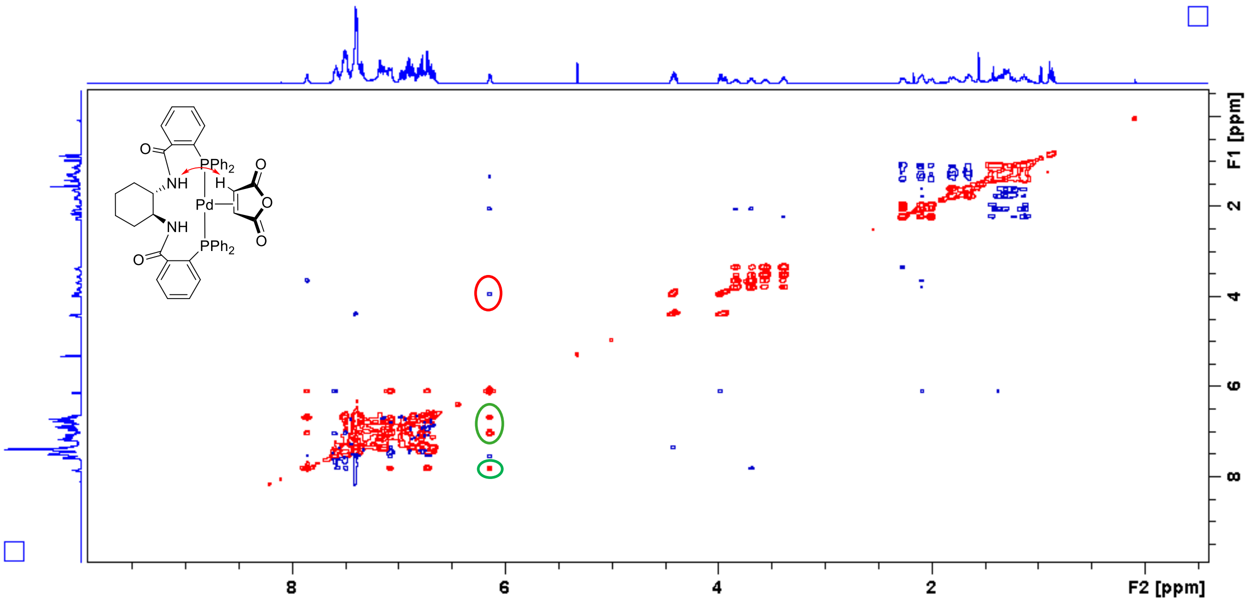

Supplementary Figure 17. ^1H-^1H NOESY spectrum (CD_2Cl_2) of **1**. The correlation between MAH and NH indicates that the major conformer has no hydrogen bond (red circle). Proton exchanges between these two conformers are observed (green circle).

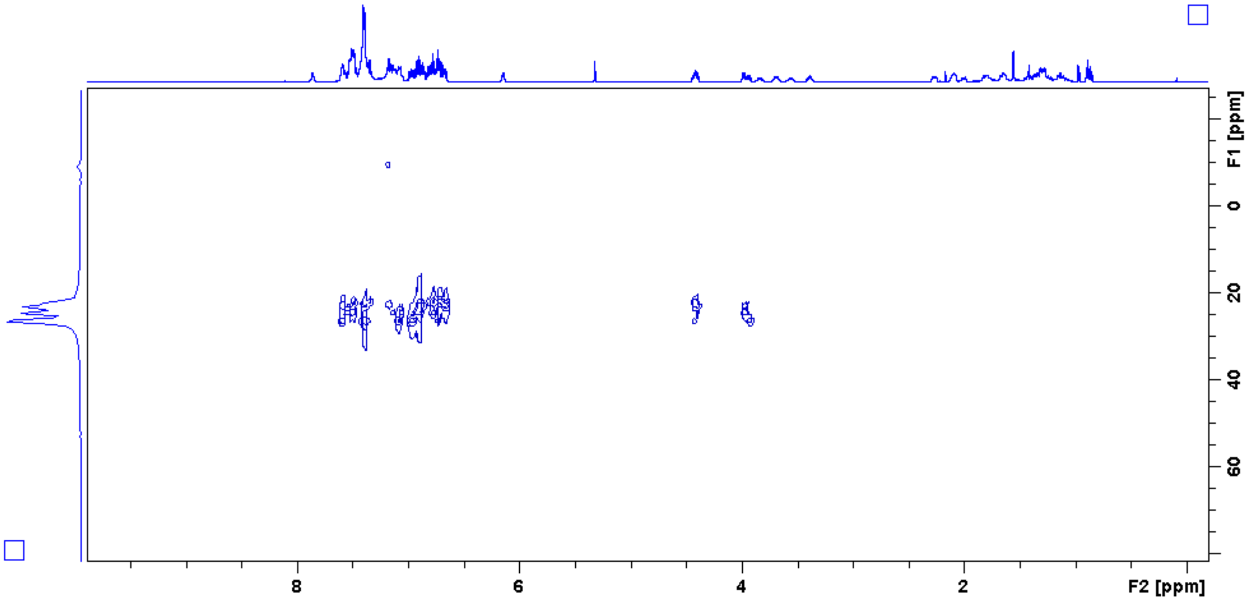

Supplementary Figure 18. ^{1}H-^{31}P HMBC spectrum (CD_2Cl_2) of **1**.

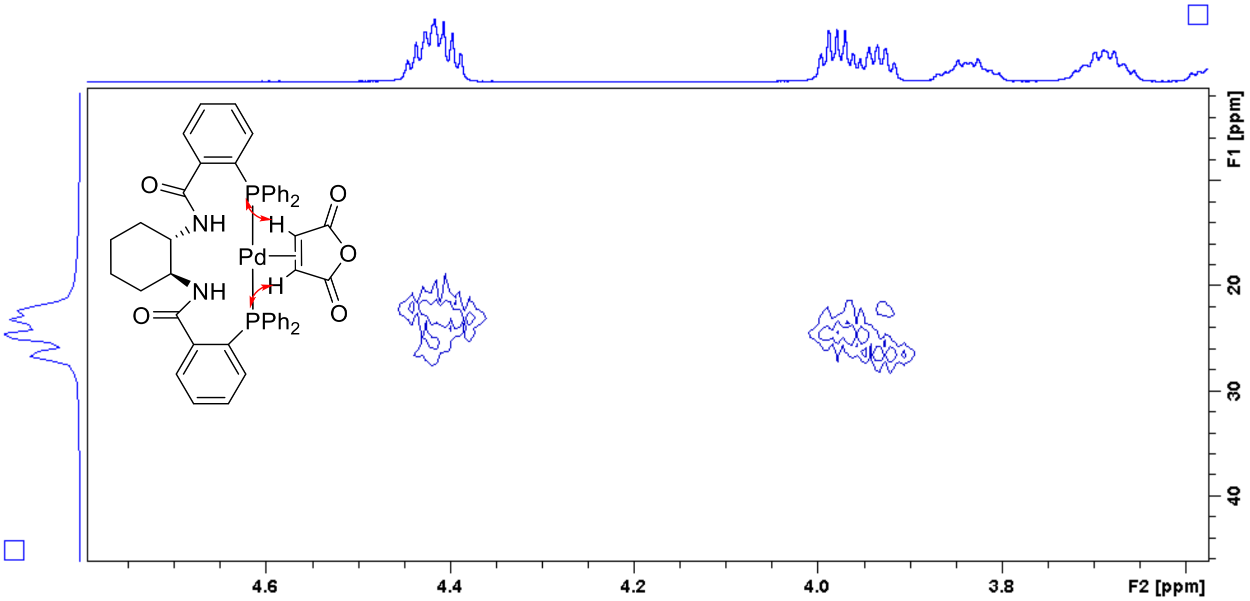

Supplementary Figure 19. ^{1}H-^{31}P HMBC NMR spectrum expansion (CD_2Cl_2) of **1**. Correlations between MAH protons and P atoms are observed.

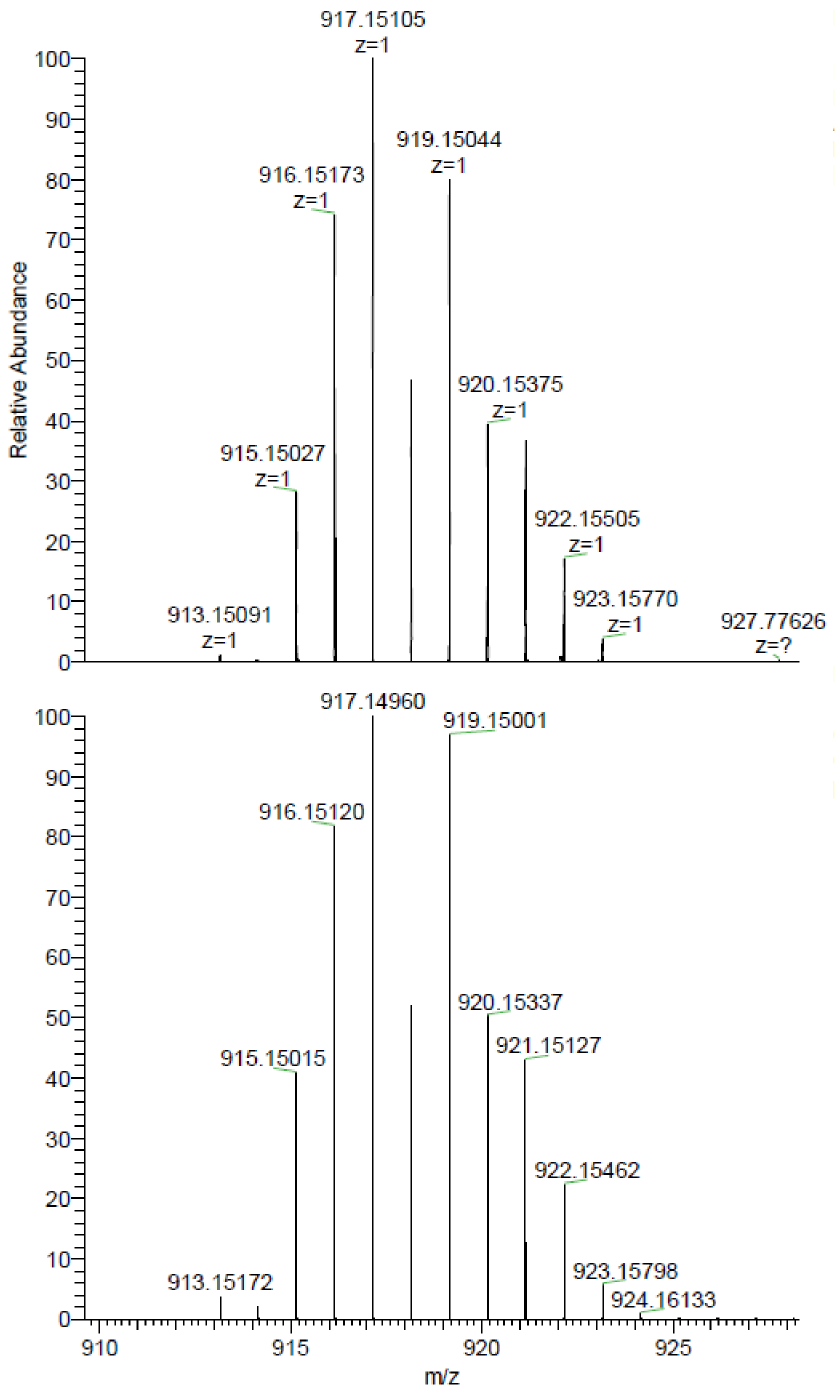

Supplementary Figure 20. Top: Experimental HRMS-ESI spectrum of [**1**·Na]$^+$. Bottom: Calculated HRMS isotope pattern for [**1**·Na]$^+$.

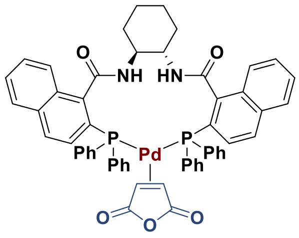

^{NAP}DACH-Pd-MAH

(*S,S*)-^{NAP}DACH-Pd-MAH (2) – The entire procedure was performed in a glovebox under a dry, oxygen-free atmosphere of nitrogen gas. A 4-dram vial was charged with of ^{DMP}DAB-Pd-MAH (100.1 mg, 0.21 mmol), (*S,S*)-DACH-naphthyl Trost ligand (168.7 mg, 0.21 mmol), and 5 mL of THF. The solution was stirred for one hour. THF was evaporated under vacuum followed by trituration/decantation with a 1:1 hexane/Et_2O solution until the washings were colourless (6 times). Residual solvents from the trituration/decantation were removed under vacuum, and the solid was dried under vacuum overnight to give a pale-yellow powder (169.6 mg, 80%). The product was recrystallized from pentane/THF.

NMR signals of **2** were generally smaller than **1**, due to the rapid conformer exchange. Some peaks are assigned as the analogue of **1**.

^{1}H NMR (500 MHz, THF-d_8) δ 8.22 (br s, 2H), 7.91-6.76 (m, 32H), 4.57 (br s), 4.39 (s, 1H), 3.95 (sept, 1H), 3.57 (s, 1H), 2.92 (br s, 1H), 2.18 (br s, 1H), 1.84-1.75 (m, 2H), 1.57-1.28 (m, 4H).

^{13}C NMR (125 MHz, THF-d_8) δ 171.0, 170.6, 168.0, 136.6, 136.5, 136.3, 136.0, 135.7, 135.5, 135.2, 135.1, 135.0, 134.9, 134.8, 134.6, 134.5, 133.6 (d, *J* = 4.3 Hz), 133.4 (d, *J* = 3.5 Hz), 132.3, 132.2, 132.1_3, 132.0_7, 130.6, 130.3, 130.0 (d, *J* = 1.7 Hz), 129.7 (d, *J* = 4.4 Hz), 129.6, 129.1, 129.0, 128.9, 128.8, 128.3, 128.2 (d, *J* = 2.6 Hz), 128.1, 127.9, 127.8, 127.7, 126.7, 126.5, 59.4, 57.0, 54.3, 54.1, 32.6, 32.2, 25.8, 24.9.

^{31}P NMR (200 MHz, THF-d_8) δ 23.2, 22.8.

HRMS (ESI): m/z calcd for $C_{56}H_{46}N_2O_5P_2Pd$, $[M + Na]^+$ (major isotopomer): 1017.1809, found: 1017.1827.

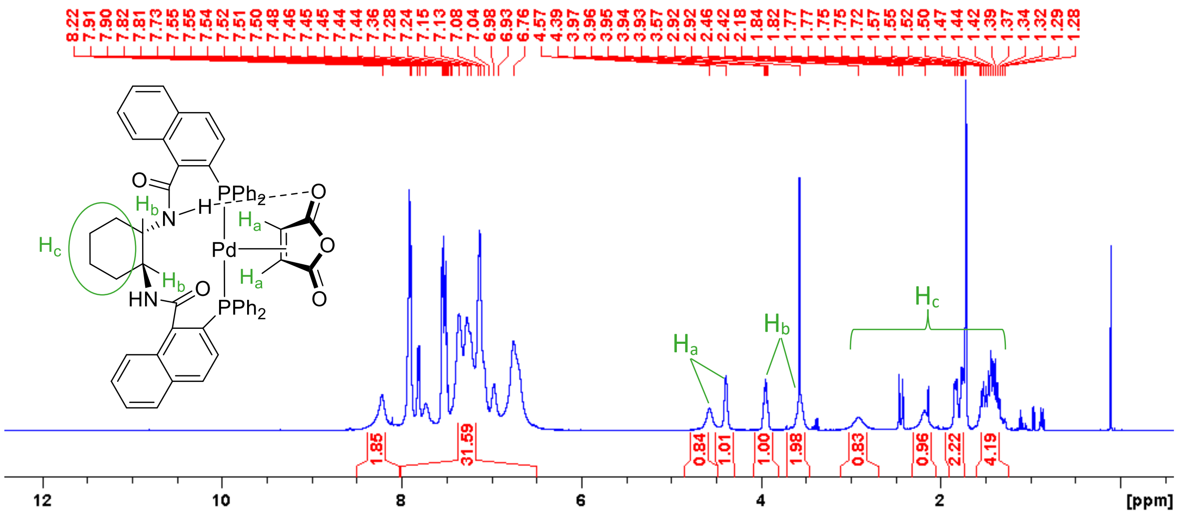

Supplementary Figure 21. ^{1}H NMR spectrum (500 MHz, THF-d_8) of **2**. Key proton signals are assigned. Singlets at 1.72 and 3.57 ppm are deuterated THF residual signals. Singlets at 2.42 ppm and 2.46 ppm are HDO and H_2O.

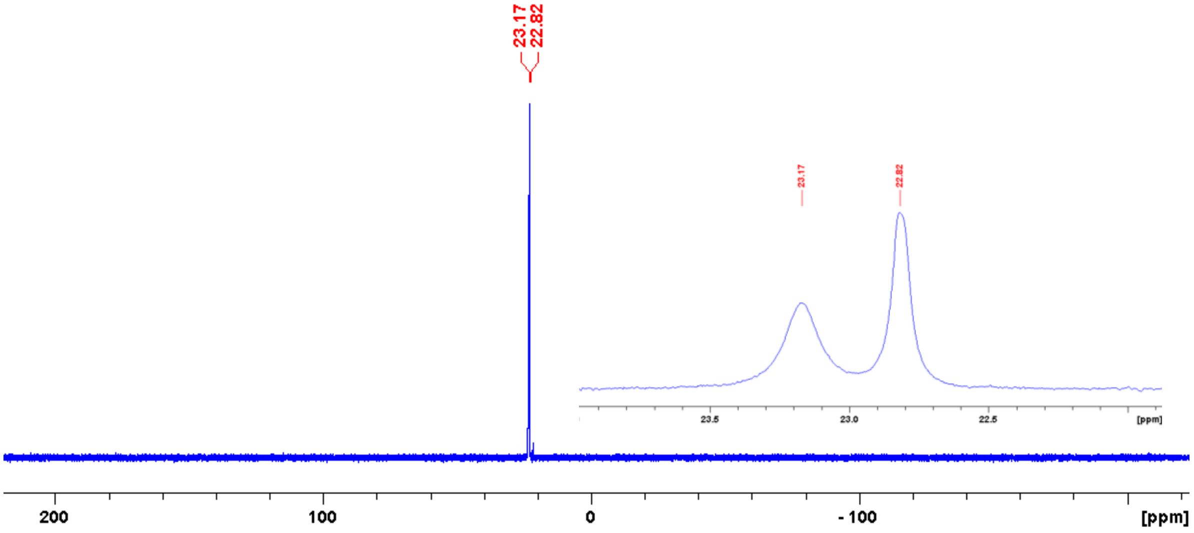

Supplementary Figure 22. $^{31}P\{^{1}H\}$ NMR spectrum (200 MHz, THF-d_8) of **2**.

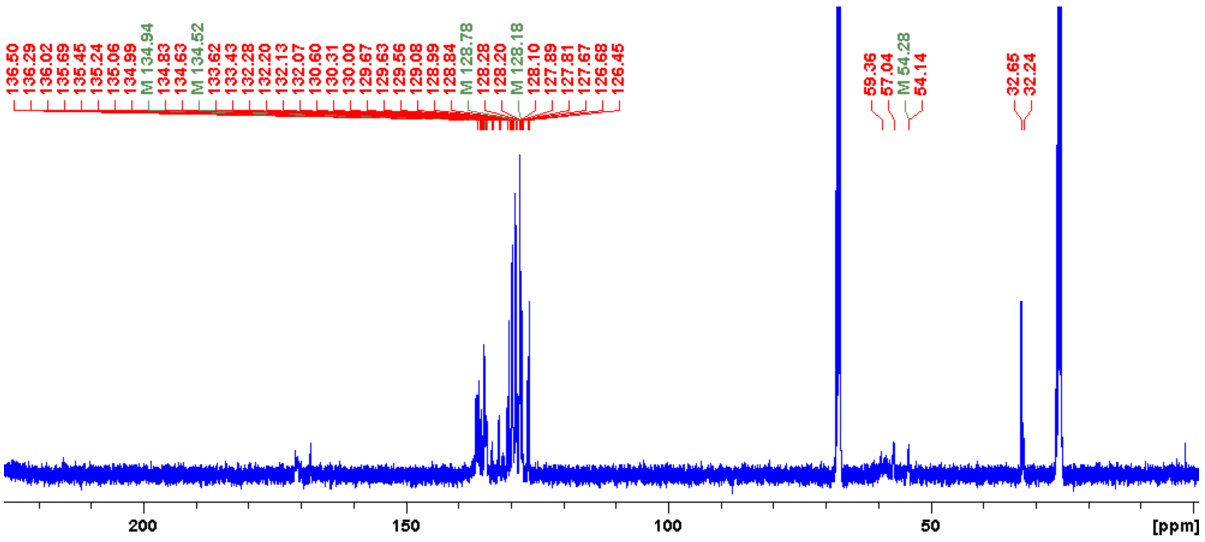

Supplementary Figure 23. $^{13}C\{^{1}H\}$ NMR spectrum (125 MHz, THF-d$_8$) of **2**.

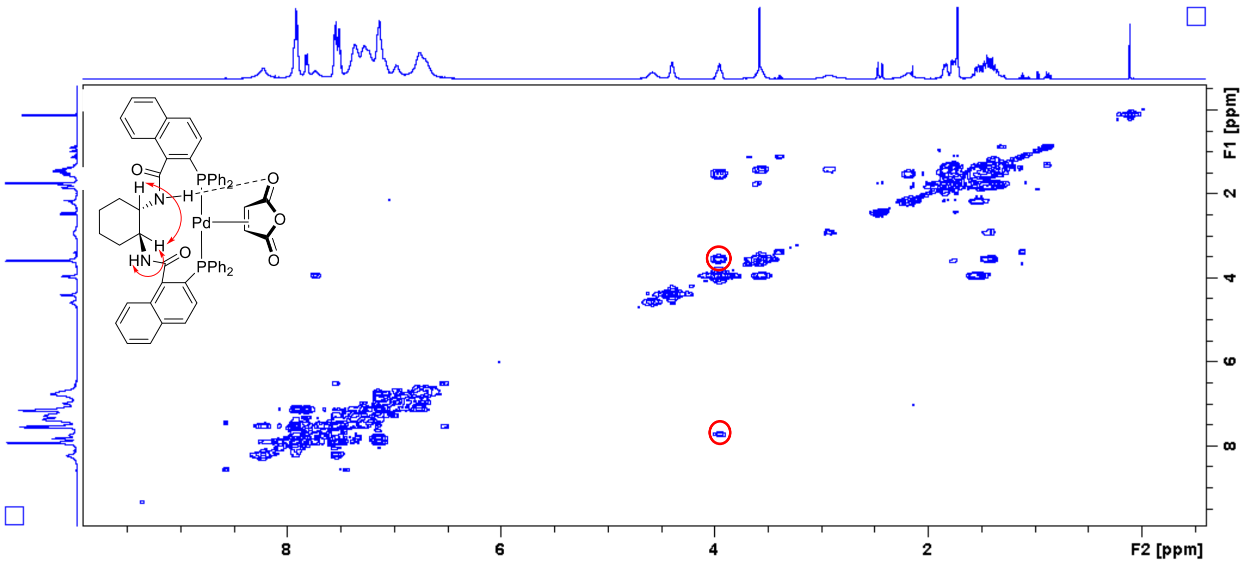

Supplementary Figure 24. ^{1}H-^{1}H COSY NMR spectrum of **2**. Correlation between two CH protons indicates one CH overlaps with the peak of THF. The correlation between CH and NH also indicates that the NH protons are in the aliphatic region.

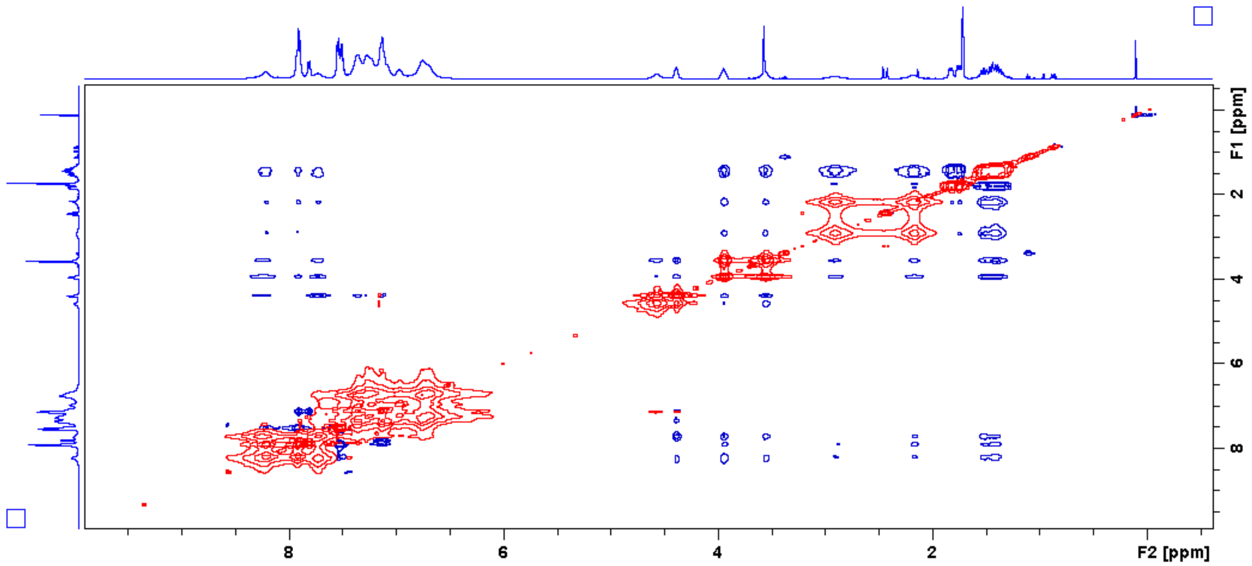

Supplementary Figure 25. ^{1}H-^{1}H NOESY NMR spectrum of **2**. Proton exchanges are observed between the two conformers.

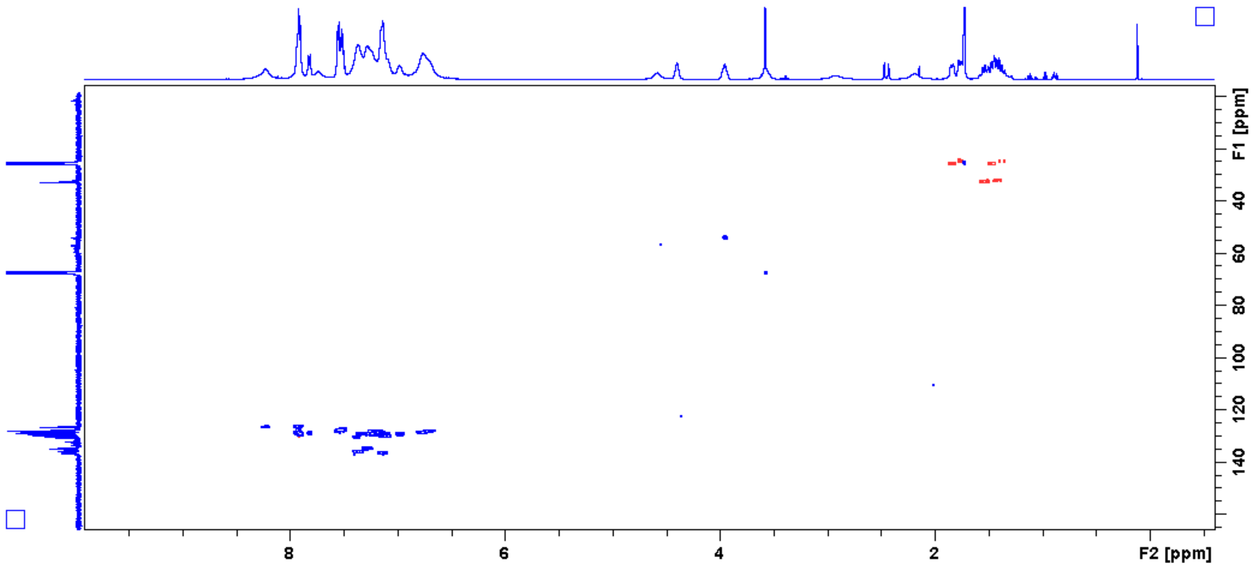

Supplementary Figure 26. ^{1}H-^{13}C HSQC NMR spectrum of **2**.

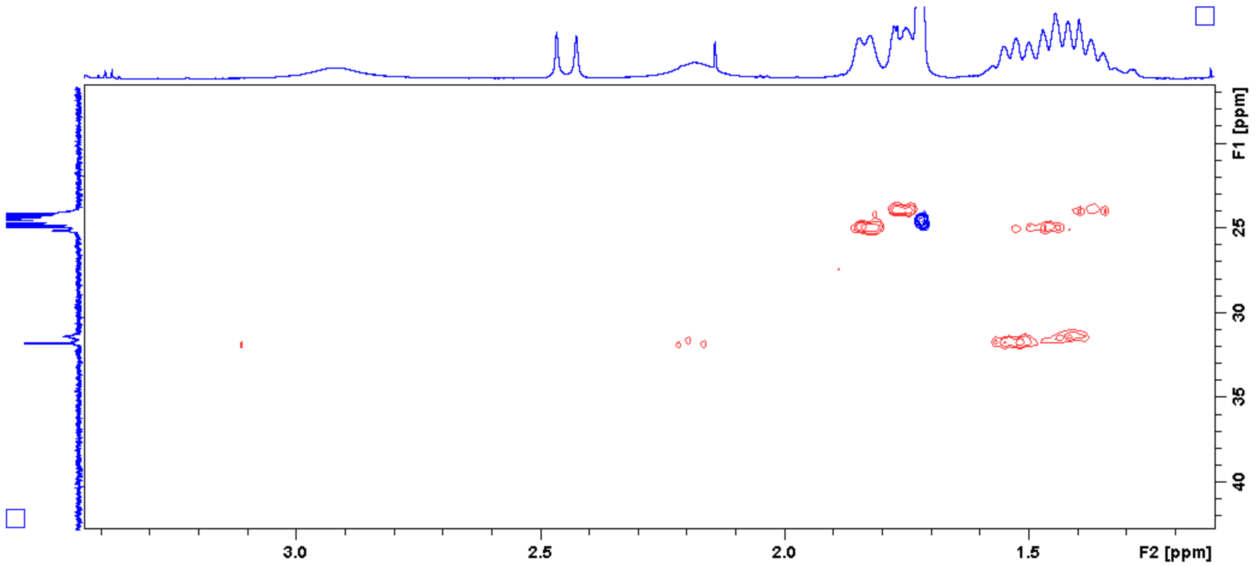

Supplementary Figure 27. ^{1}H-^{13}C HSQC NMR spectrum of **2**. Cyclohexyl region expansion. Two cyclohexyl carbons overlap with the solvent peaks.

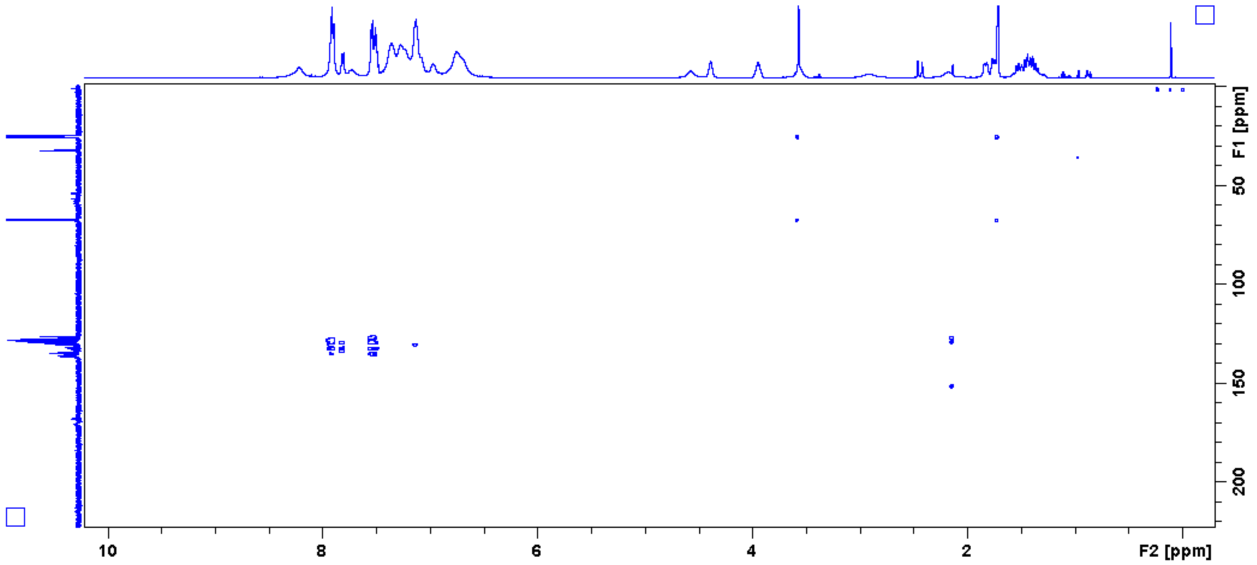

Supplementary Figure 28. ^{1}H-^{13}C HMBC NMR spectrum of **2**.

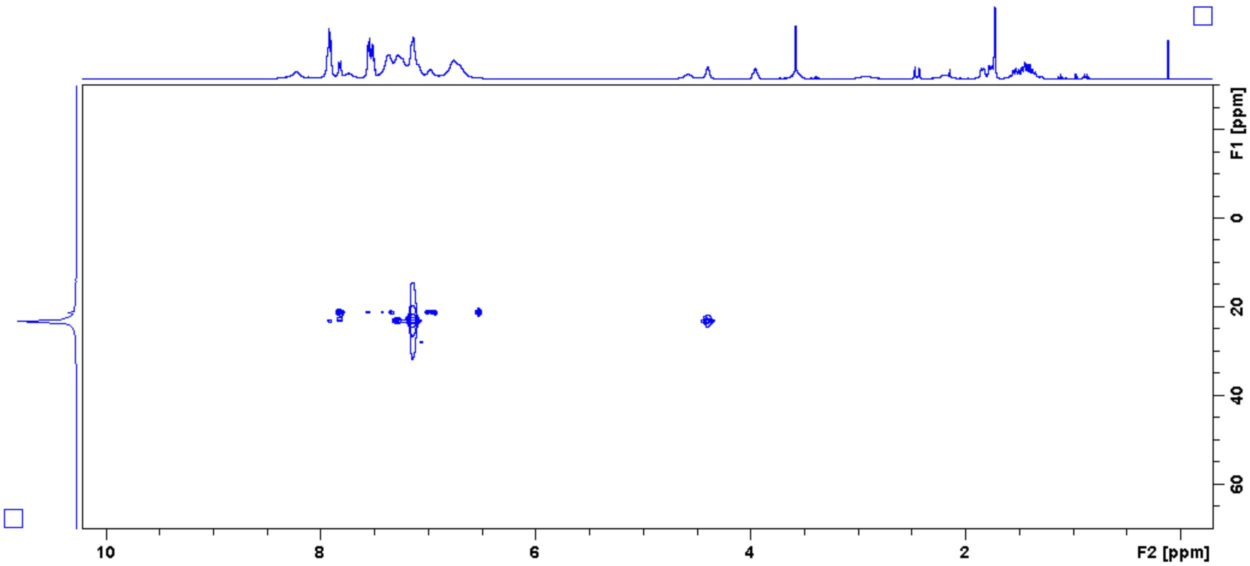

Supplementary Figure 29. ^{1}H-^{31}P HMBC NMR spectrum of **2**.

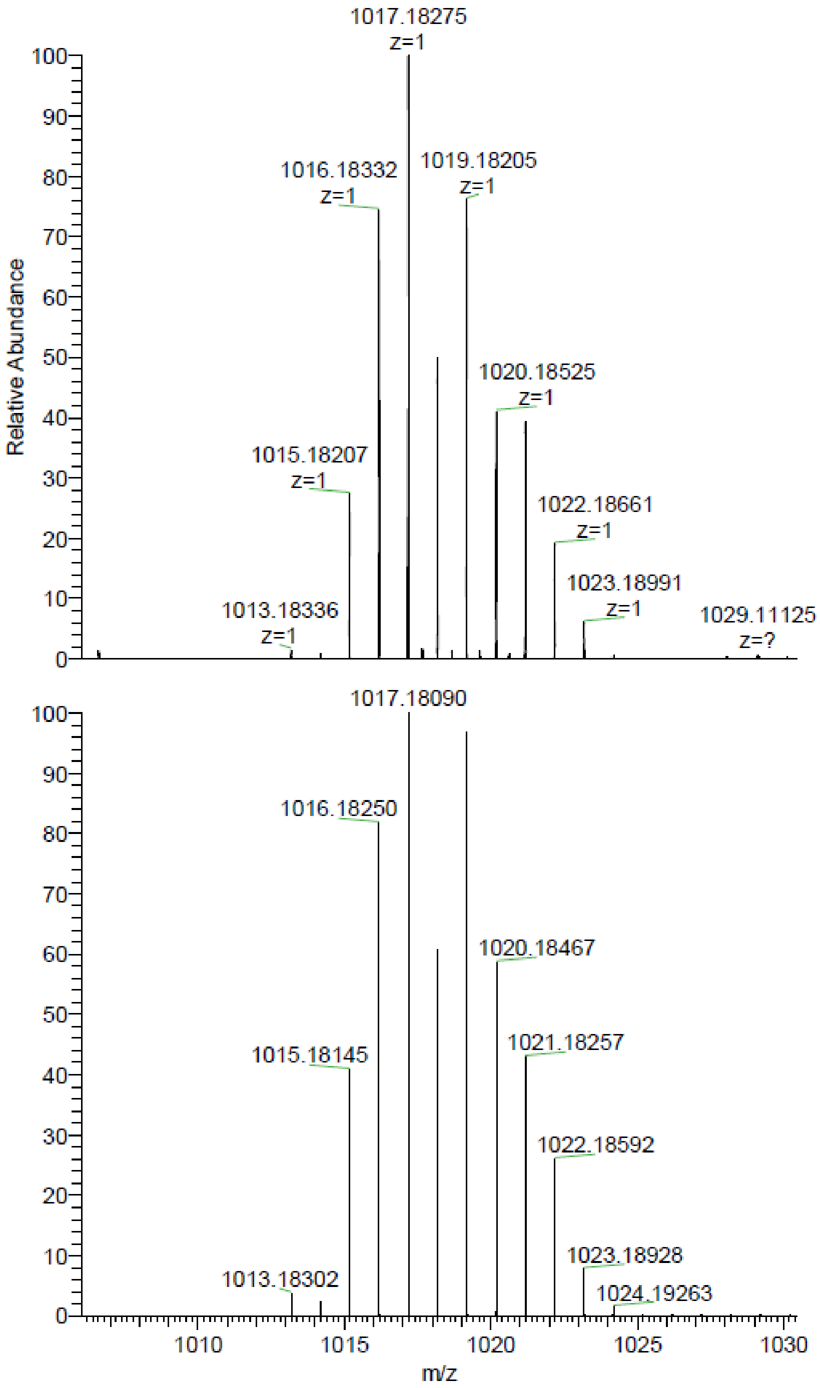

Supplementary Figure 30. Top: Experimental HRMS-ESI spectrum of [**2**·Na]$^{+}$. Bottom: Calculated HRMS isotope pattern for [**2**·Na]$^{+}$.

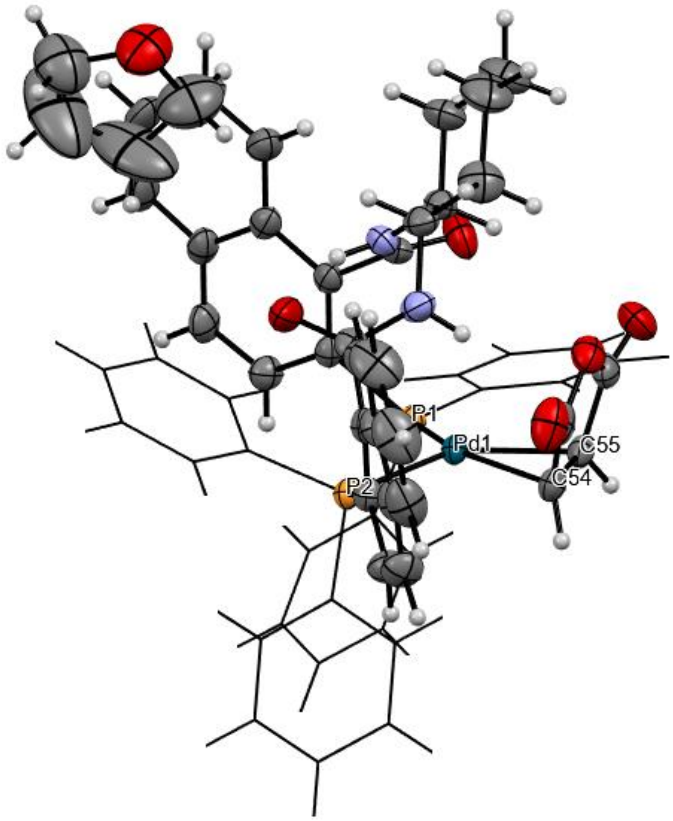

Supplementary Figure 31. Solid-state molecular structure of complex **2**, including THF solvate. Thermal ellipsoids plotted at 50% probability for non-H atoms; H-atoms shown as uniformly-sized white spheres and phenyl rings shown as wireframes for clarity. X-ray diffraction data collected at 100.15 K. Selected bond lengths (Å) and angles (°): Pd1–P1: 2.3877(15); Pd1–P2: 2.3776(12); Pd1–C54: 2.166(3); Pd1–C55: 2.152(3); C54–C55: 1.435(4); P1–Pd1–P2: 110.5(5); C54–Pd1–C55: 38.82(11); C55–Pd1–P1: 108.41(9); C54–Pd1–P2: 100.52(10).

VT NMR Spectroscopy of 2

Procedure: VT NMR experiments were conducted at elevated temperatures: **2** (8 mg, 0.0089 mmol) was dissolved in 0.6 mL toluene-d$_8$. A series of ^{31}P NMR & ^{1}H NMR spectra were obtained at 26 °C, 32 °C, 40 °C, 50 °C, 60 °C.

A second set of VT experiments was performed at low temperatures: **2** (10 mg, 0.011 mmol) was dissolved in 0.6 mL toluene-d$_8$. A series of ^{31}P NMR and ^{1}H NMR spectra were obtained at 25 °C, 20 °C, 10 °C, 0 °C, –10 °C, –20 °C, –30 °C, –40 °C, –50 °C, –60 °C, –70 °C, –80 °C.

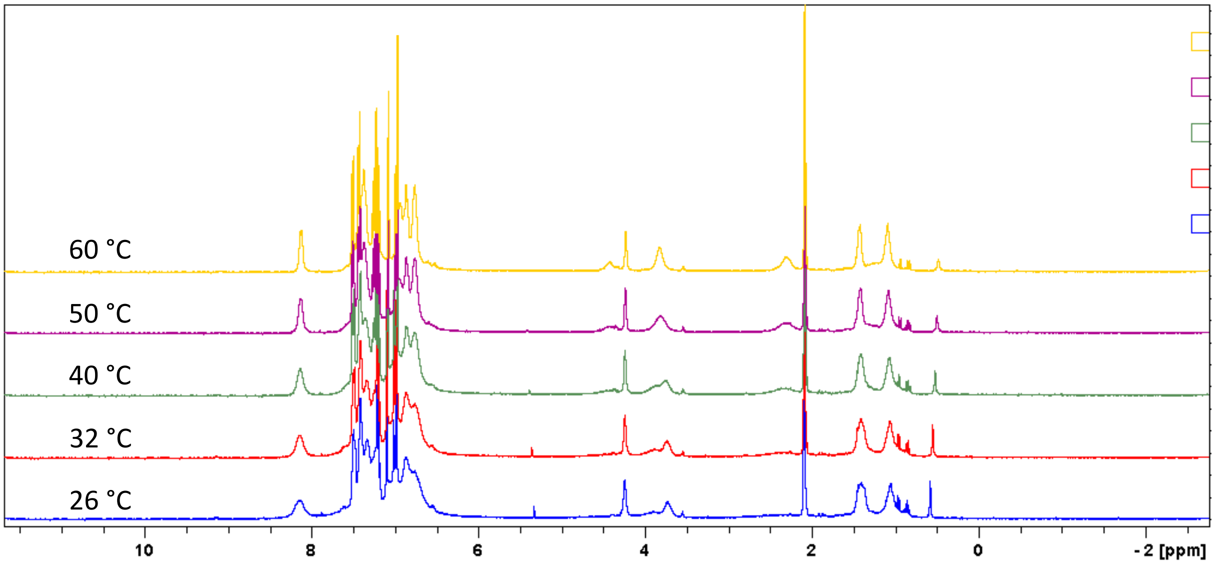

Supplementary Figure 32. ^{1}H NMR spectra (360 MHz, toluene-d$_8$) at high temperatures of **2**. Temperatures of the spectra increase from the bottom, 26 °C, to the top, 60 °C.

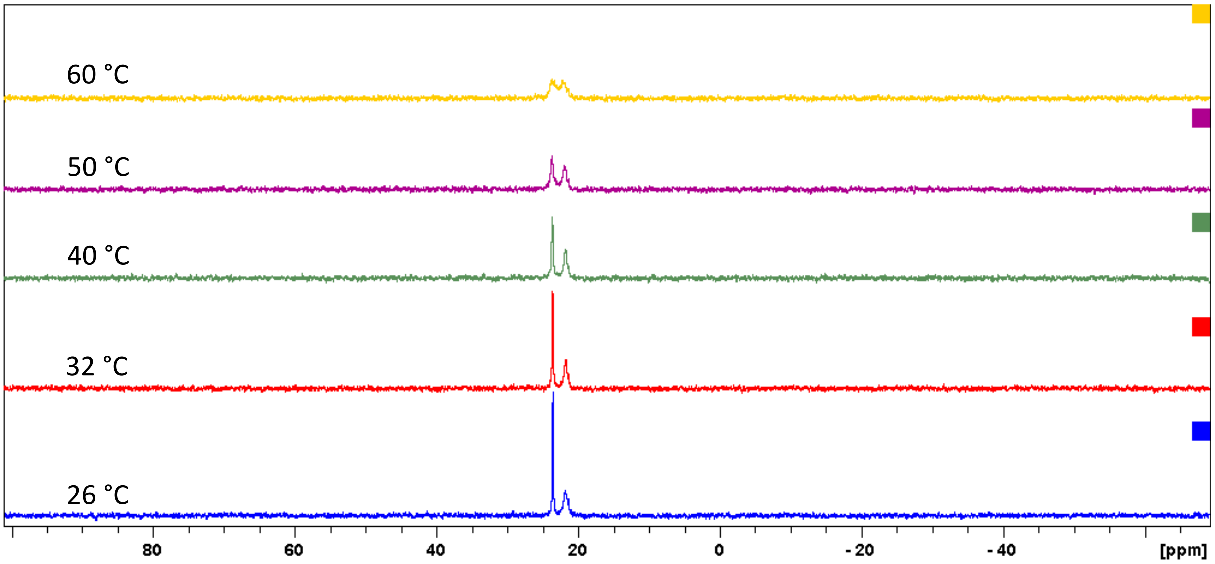

Supplementary Figure 33. ^{31}P{^{1}H} NMR spectra (145 MHz, toluene-d$_8$) at high temperatures of **2**. Temperatures of the spectra increase from the bottom, 26 °C, to the top, 60 °C.

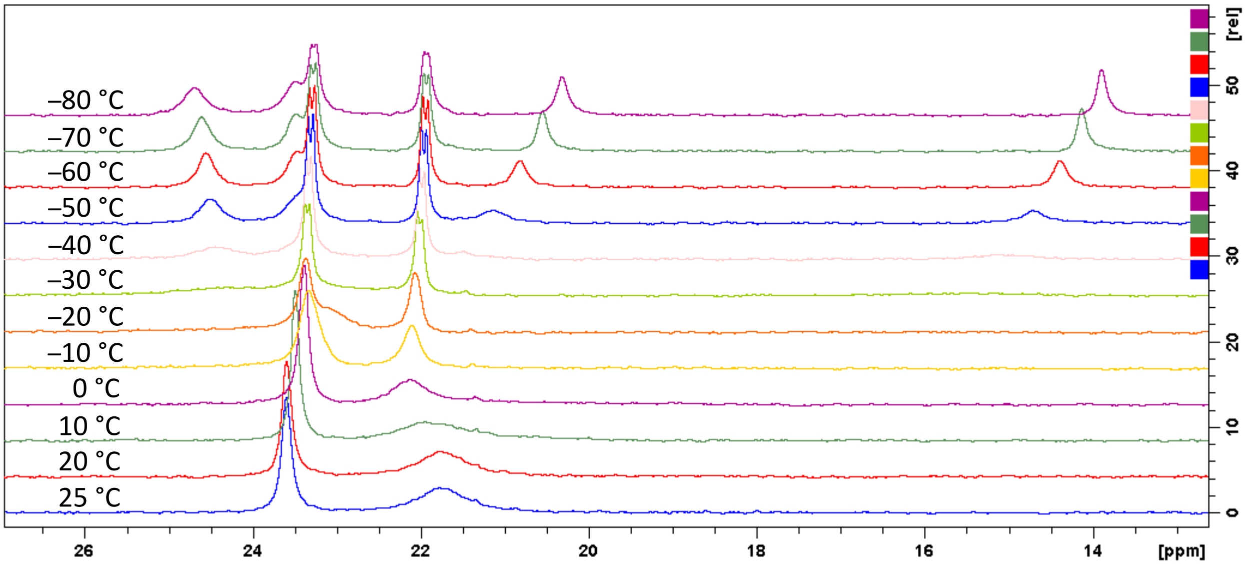

Supplementary Figure 34. $^{31}P\{^{1}H\}$ NMR spectra (145 MHz, toluene-d8) at high temperatures of **2**. Expansion of product peaks. Temperatures of the spectra decrease from bottom, 25 °C, to top, –80 °C. No signal detected beyond this range.

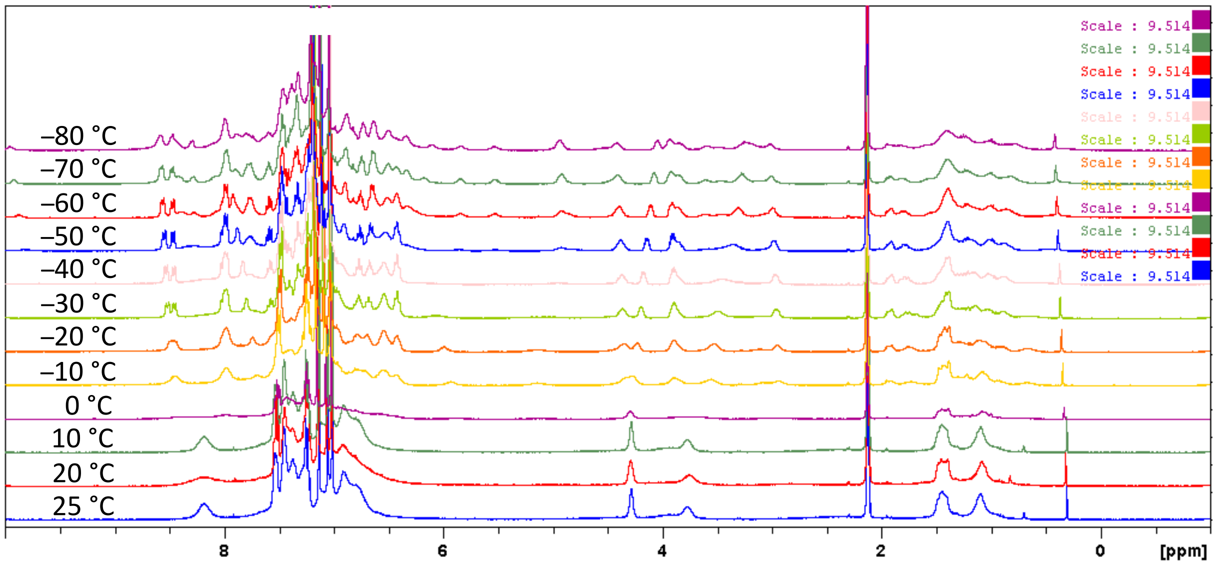

Supplementary Figure 35. ^{1}H NMR spectra (360 MHz, toluene-d8) at high temperatures of **2**. Temperatures of the spectra decrease from bottom, 25 °C, to top, –80 °C.

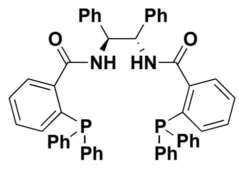

(*S*,*S*)-^{Ph}STIL (L3)

(*S,S*)-^{Ph}STIL (L3) – To a CH_2Cl_2 (42 mL) solution of DMAP (68 mg, 0.56 mmol), 2-(diphenylphosphanyl)benzoic acid (3.81 g, 12.4 mmol) and EDAC (2.1 g, 13.6 mmol) was added (1*S*,2*S*)-(−)-1,2-diphenylethylenediamine (1.2 g, 5.7 mmol), and the mixture stirred at rt overnight. Et_2O (80 mL) was added, and the organic phase was washed sequentially with 10% HCl (3 × 80 mL), water (1 × 80 mL), saturated aqueous $NaHCO_3$ (2 × 80 mL), 1 M NaOH (1 × 80 mL) and a saturated aqueous solution of brine (1 × 80 mL). The organic layer was dried over anhydrous $MgSO_4$, filtered, and the solvent removed under reduced pressure. The residue was purified by flash column chromatography over silica gel eluting with a hexanes/EtOAc gradient. The resulting white solid was re-dissolved in THF and dried with CaH_2 under N_2. The mixture was filtered, and the solvent removed under reduced pressure to recover the title compound (895 mg, 20%) as a white solid. This synthetic method is adapted from a reported procedure, and the NMR data are consistent with the reported values.[2]

^{1}H NMR (300 MHz, $CDCl_3$) δ 7.53-7.49 (m, 2H), 7.21-6.92 (m, 32H), 6.80-6.76 (m, 6H), 5.24 (dd, 2H).

^{31}P NMR (121 MHz, $CDCl_3$) δ –10.15.

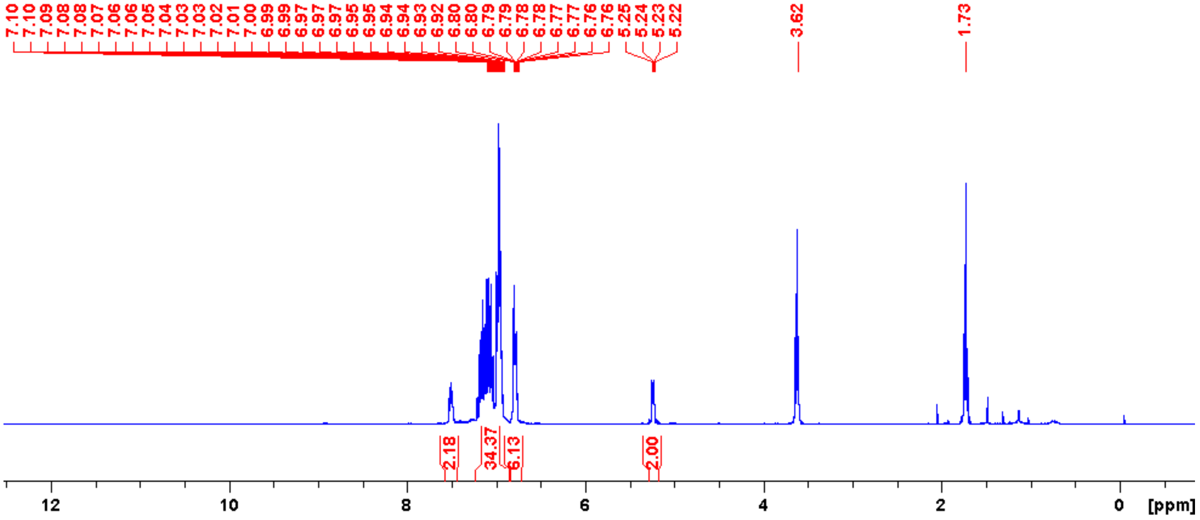

Supplementary Figure 36. ^{1}H NMR spectrum (300 MHz, $CDCl_3$) of (*S*,*S*)-^{Ph}STIL (**L3**).
Multiplets at 1.73 and 3.62 are regular THF residues.

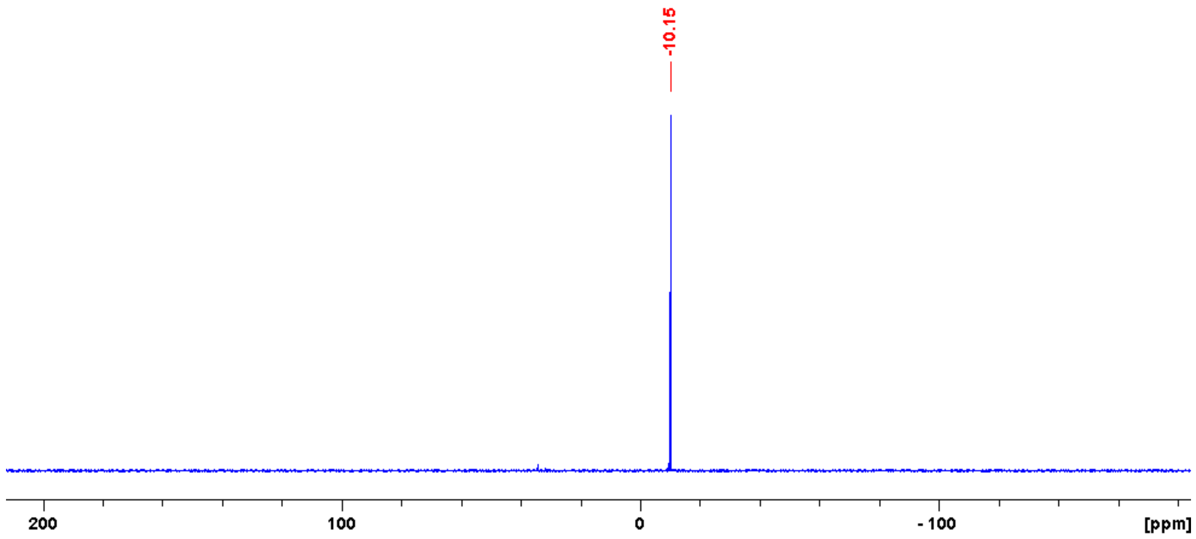

Supplementary Figure 37. $^{31}P\{^{1}H\}$ NMR spectrum (121 MHz, $CDCl_3$) of (*S*,*S*)-^{Ph}STIL (**L3**).

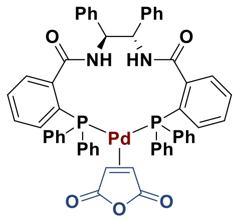

^{Ph}STIL-Pd-MAH

(*S,S*)-^{Ph}STIL-Pd-MAH (3) – The entire procedure was performed under an inert atmosphere. A 4-dram vial was charged with of ^{DMP}DAB-Pd-MAH (100 mg, 0.21 mmol), (*S,S*)-diphenyl Trost ligand (168.5 mg, 0.21 mmol) and 6 mL of anhydrous, inhibitor-free THF. The reaction mixtures were stirred for 3 h at rt. The solution was filtered through a pad of Celite© and the solvent evaporated under reduced pressure. The crude residue was then triturated/decanted six times with hexane until the washing was colourless. Residual solvents from the trituration/decantation were removed under vacuum and the desired product was precipitated from THF/hexane (1:4, 5 mL). The product was finally dried under vacuum to give a tan solid (146.2 mg, 68%).

Two conformers were observed when **3** was dissolved in THF.

^{1}H NMR (500 MHz, THF-d$_8$) δ *Minor conformer:* 8.16 (d, 1H), 7.66-6.71 (m, 38H), 5.55 (m, 1H), 5.11 (m, 1H), 4.52 (m, 1H), 4.08 (m, 1H). *Major conformer:* 8.66 (d, 1H), 8.06 (d, 1H), 7.66-6.71 (m, 38H), 5.28 (m, 1H), 5.13 (m, 1H), 4.60 (m, 1H), 4.27 (m, 1H).

^{13}C NMR (125 MHz, THF-d$_8$) δ 170.8 (d, $^3J_{C\text{-}P}$ = 2.7 Hz), 170.2 (d, $^3J_{C\text{-}P}$ = 3.5 Hz), 169.1 (d, $^3J_{C\text{-}P}$ = 4.4 Hz), 167.8 (d, $^3J_{C\text{-}P}$ = 3.5 Hz), 143.3, 143.1, 141.5, 141.2, 139.2, 139.1, 139.0, 137.8, 137.7, 137.6, 137.4, 137.3, 136.2, 136.1, 136.0, 135.8$_3$, 135.8$_0$, 135.6, 135.5, 135.3, 135.2, 135.1$_4$, 135.0$_8$, 135.0$_4$, 135.0$_1$, 134.9, 134.8$_3$, 134.7$_9$, 134.6, 134.4, 134.2, 134.0, 133.9, 133.8, 133.7, 133.6, 133.5, 133.2, 133.1, 132.6, 130.8 (d, *J* = 1.8 Hz), 129.7$_2$, 129.6$_8$, 129.6, 129.5$_3$, 129.4$_6$, 129.4, 129.3 (d, *J* = 2.7 Hz), 129.2$_2$, 129.1$_7$, 128.9$_1$, 128.8$_7$, 128.7$_4$, 128.6$_9$, 128.5$_2$, 128.5$_0$, 128.3, 128.2, 128.1$_3$, 128.0$_9$, 128.0$_0$, 127.9$_7$, 127.9, 127.7, 127.6, 127.5$_1$, 127.4$_8$, 127.4$_4$, 127.3$_9$, 127.3, 127.1, 127.0, 126.9, 126.8, 126.4, 62.0, 61.1, 59.9, 59.5, 57.7 (dd, $^2J_{C\text{-}P1}$ = 4.4 Hz, $^2J_{C\text{-}P2}$ = 24.7 Hz), 57.2 (dd, $^2J_{C\text{-}P1}$ = 3.5 Hz, $^2J_{C\text{-}P2}$ = 24.7 Hz), 57.1 (dd, $^2J_{C\text{-}P1}$ = 3.5 Hz, $^2J_{C\text{-}P2}$ = 24.7 Hz), 54.6 (dd, $^2J_{C\text{-}P}$ = 26.4 Hz, the other coupling constant could not be determined due to low signal-to-noise ratio), 53.8 (dd, $^2J_{C\text{-}P}$ = 24.7 Hz, C=C, another coupling constant was not able to be determined due to low signal-to-noise ratio).

^{31}P NMR (200 MHz, THF-d$_8$) δ 26.7 (d, $^2J_{P\text{-}P}$ = 9.9 Hz, *minor conformer*), 24.6 (d, $^2J_{P\text{-}P}$ = 5.4 Hz, *major conformer*), 24.0 (d, $^2J_{P\text{-}P}$ = 5.3 Hz, *major conformer*), 23.3 (d, $^2J_{P\text{-}P}$ = 10.1 Hz, *minor conformer*).

HRMS (ESI): m/z calcd for $C_{56}H_{44}N_2O_5P_2Pd$, [M + Na]$^+$ (major isotopomer): 1015.1653, found: 1015.1671.

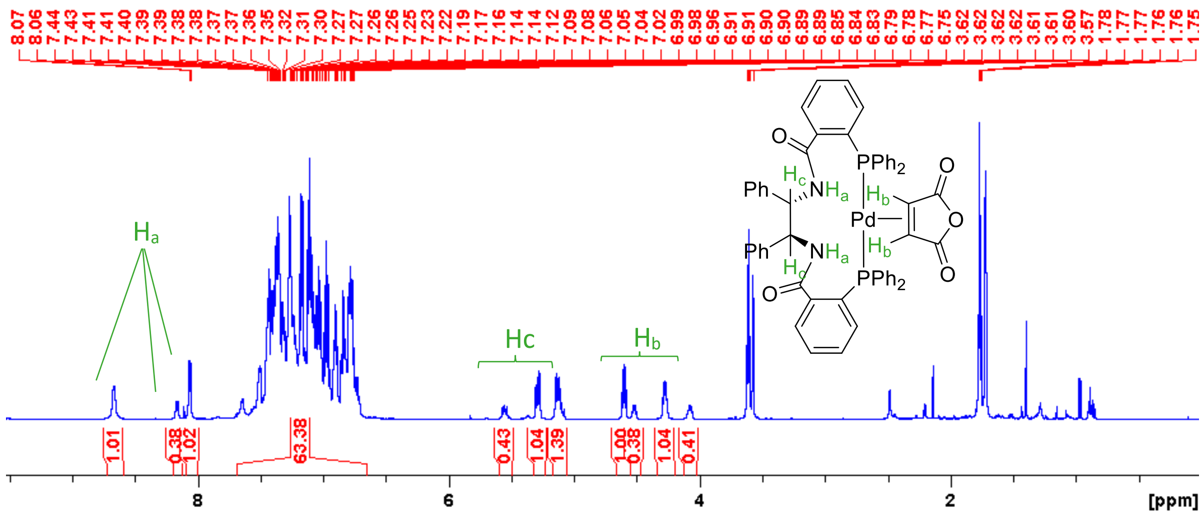

Supplementary Figure 38. ^{1}H NMR spectrum (500 MHz, THF-d$_8$) of **3**. Key proton signals are assigned. Singlets at 1.72 and 3.57 ppm are deuterated THF residual signals. Multiplets at 1.77 and 3.61 ppm are protio THF. Singlet at 2.48 ppm is water. Multiplets at 0.89 and 1.28 ppm are hexanes.

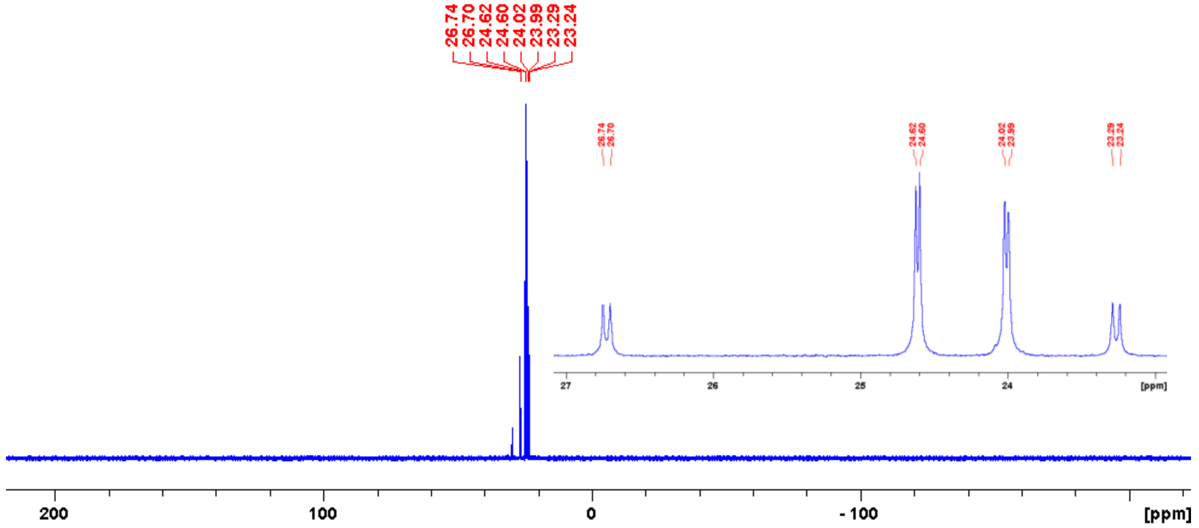

Supplementary Figure 39. $^{31}P\{^{1}H\}$ NMR spectrum (200 MHz, THF-d$_8$) of **3**.

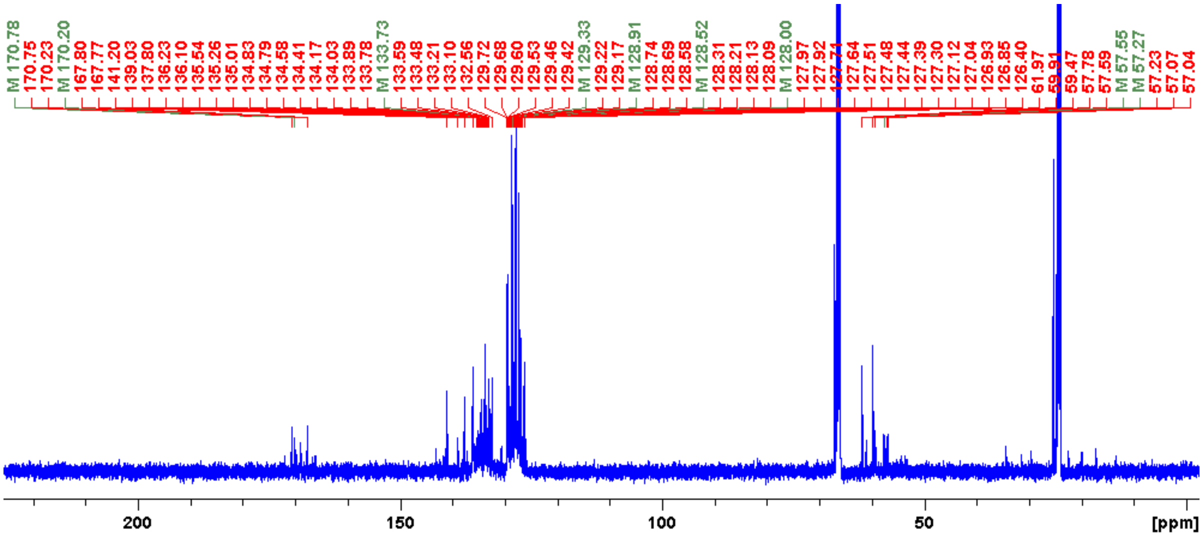

Supplementary Figure 40. $^{13}C\{^{1}H\}$ NMR spectrum (125 MHz, THF-d_8) of **3** (Green labels are manually-picked peaks in multiplets).

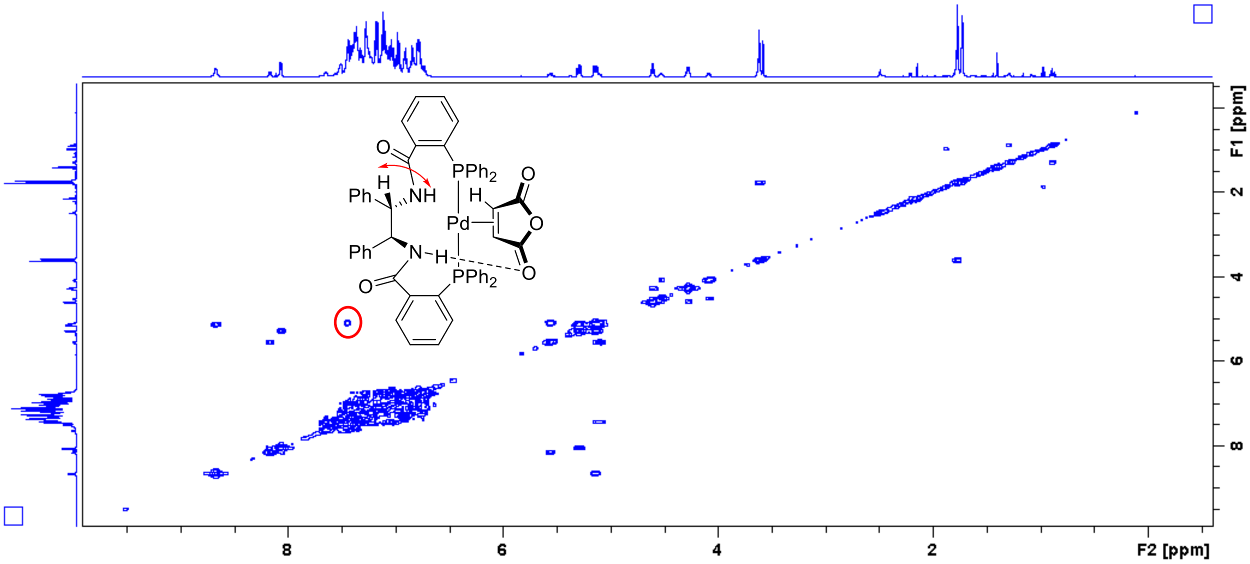

Supplementary Figure 41. ^{1}H-^{1}H COSY NMR spectrum of **3**. One NH of the minor conformer (at 7.44 ppm) hides in the aliphatic region, indicated by the correlation between NH and CH.

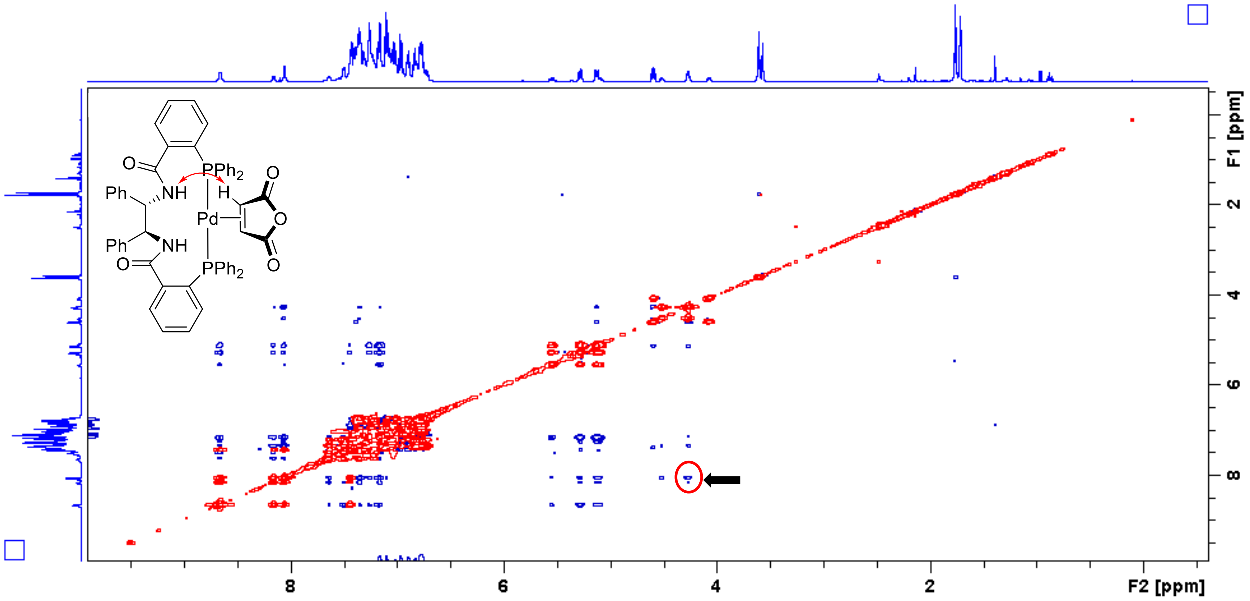

Supplementary Figure 42. ^{1}H-^{1}H NOESY NMR spectrum of **3**. The correlation between NH and MAH-H of the major conformer indicates the conformational structure without hydrogen bonding.

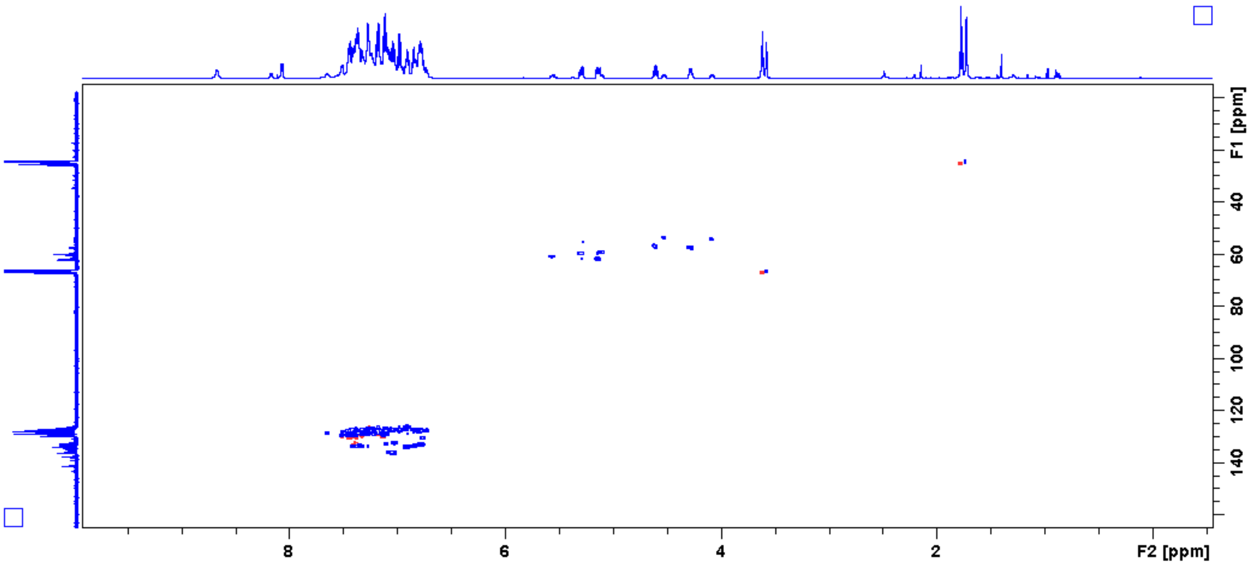

Supplementary Figure 43. ^{1}H-^{13}C HSQC NMR spectrum of **3**.

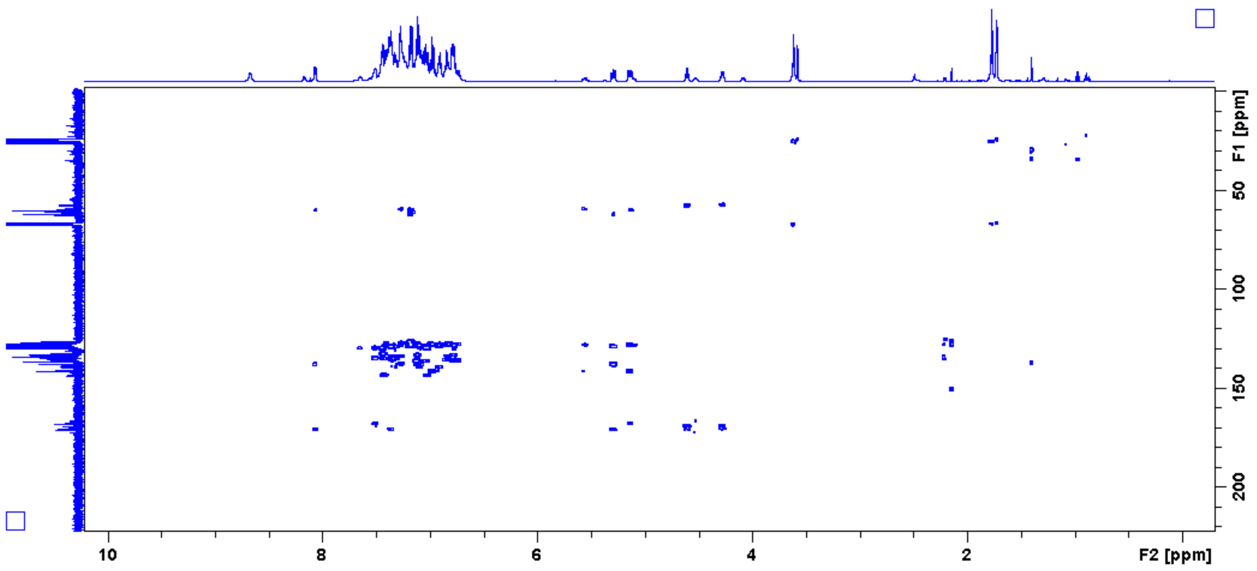

Supplementary Figure 44. ^{1}H-^{13}C HMBC NMR spectrum of **3**.

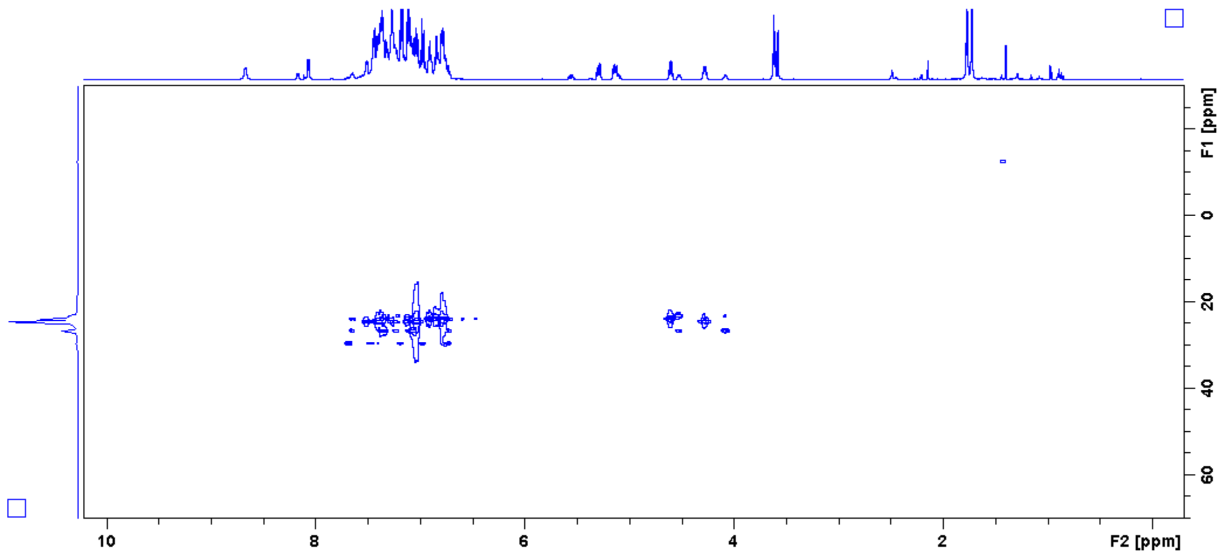

Supplementary Figure 45. ^{1}H-^{31}P HMBC NMR spectrum of **3**.

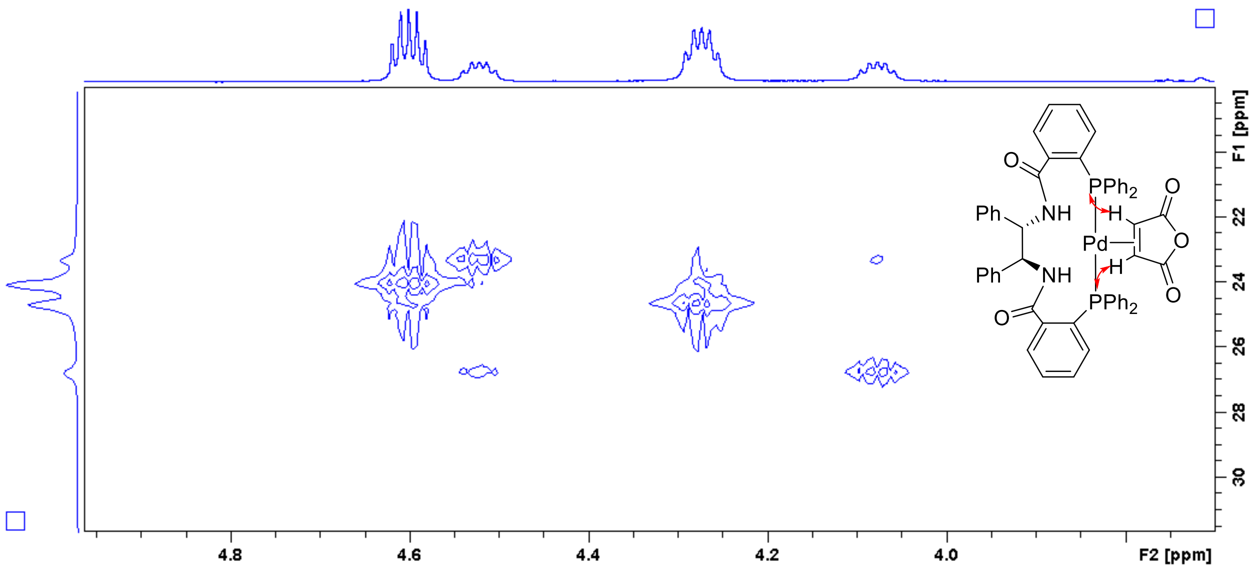

Supplementary Figure 46. ^{1}H-^{31}P HMBC NMR spectrum expansion of **3**. The correlation between P and MAH-H.

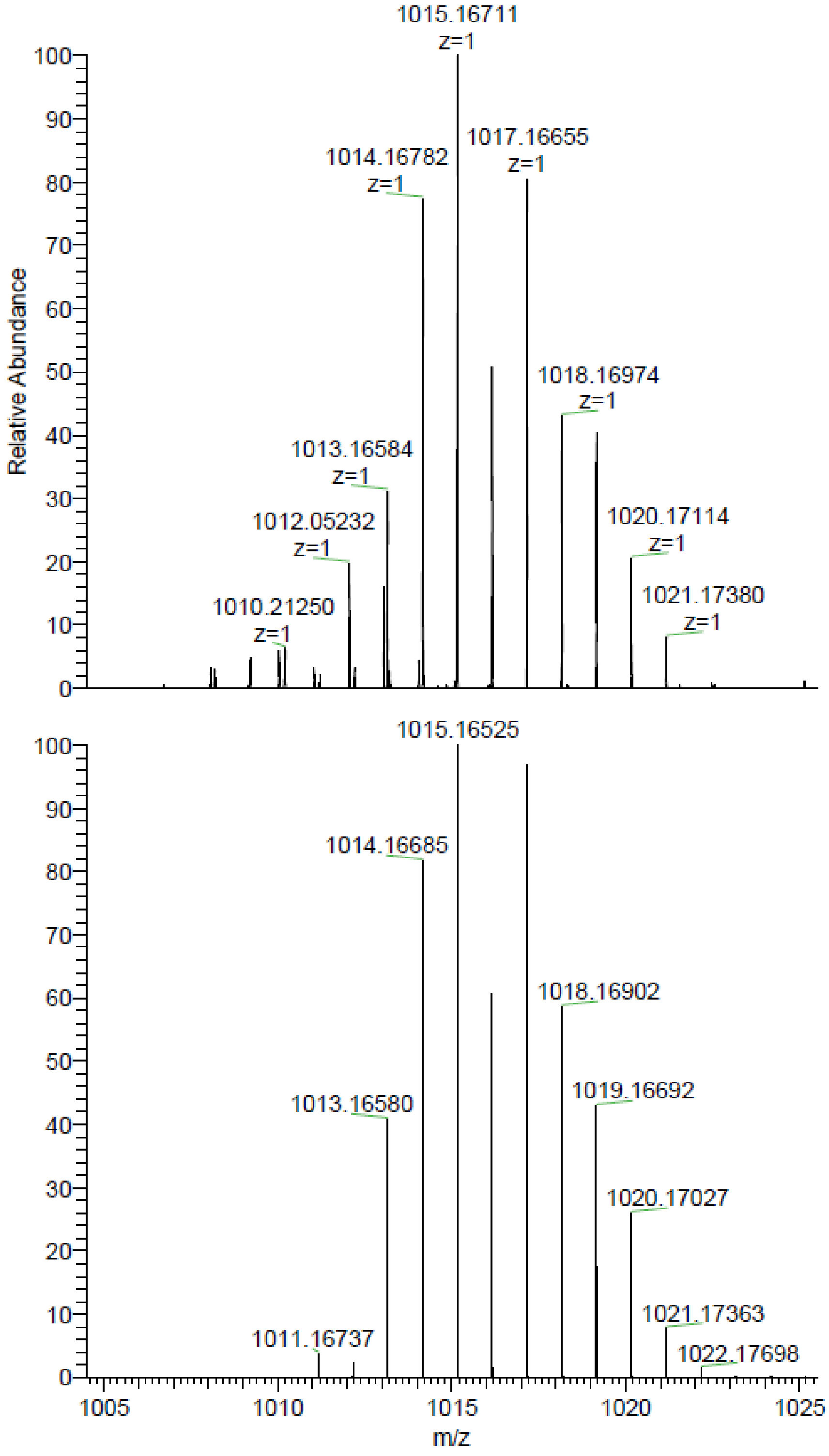

Supplementary Figure 47. Top: Experimental HRMS-ESI spectrum of [**3**·Na]$^+$. Bottom: Calculated HRMS isotope pattern for [**3**·Na]$^+$.

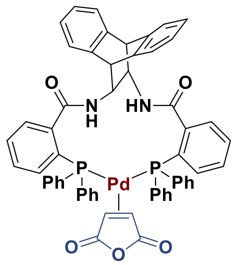

PhANDEN-Pd-MAH

(*S,S*)-^{Ph}ANDEN-Pd-MAH (4) – The entire procedure was performed under an inert atmosphere. A 4-dram vial was charged with ^{DMP}DAB-Pd-MAH (70 mg, 0.18 mmol), (*S,S*)-ANDEN Trost ligand (121 mg, 0.15 mmol) and 9 mL of anhydrous THF. The solution was stirred for 1 h at rt. THF was evaporated under reduced pressure and the crude residue was triturated/decanted with hexane/Et_2O (1:1) until the washings were colourless (6 times). The product was dried under vacuum to give a yellow solid (116.0 mg, 50%). The product was recrystallized from TBME/THF.

The product was dried under vacuum to give a light green solid (128.0 mg, 66%). The product was recrystallized from TBME/THF.

^{1}H NMR (500 MHz, THF-d_8) δ 7.80 (dd, *J* = 7.6 Hz, *J* = 2.5 Hz, 1H), 7.57 (dd, *J* = 7.4 Hz, *J* = 1.9 Hz, 1H), 7.48-6.70 (m, 36H), 4.48 (m, 1H), 4.28 (dd, 2H), 3.80 (m, 1H), 3.70 (m, 1H), 3.04 (m, 1H).

^{13}C NMR (125 MHz, THF-d_8) δ 170.3 (d, $^{3}J_{C\text{-}P}$ = 3.5 Hz), 169.0, 168.3 (d, $^{3}J_{C\text{-}P}$ = 3.5 Hz), 143.0, 142.7, 140.0, 138.4, 136.7, 136.6, 136.5, 136.3_3, 136.3_1 (d, *J* = 3.5 Hz), 135.7, 135.6, 134.4, 133.2, 133.1, 133.0, 132.2, 132.1, 129.7 (d, *J* = 5.3 Hz), 129.6, 129.1 (d, *J* = 3.51 Hz), 128.5, 128.3_1, 128.2_5, 128.2, 127.0 (d, *J* = 3.5 Hz), 127.9 (d, *J* = 3.5 Hz), 127.8, 127.5, 127.4, 127.3, 127.2, 127.1, 126.4, 126.0, 125.9, 125.8, 125.7, 125.6, 124.0, 123.3, 60.0, 60.0, 57.0, 56.7, 56.6, 50.0, 48.4.

^{31}P NMR (200 MHz, THF-d_8) δ 27.6 (m).

HRMS (ESI): m/z calcd for $C_{58}H_{44}N_2O_5P_2Pd$, $[M + Na]^+$ (major isotopomer): 1039.1653, found: 1039.1672.

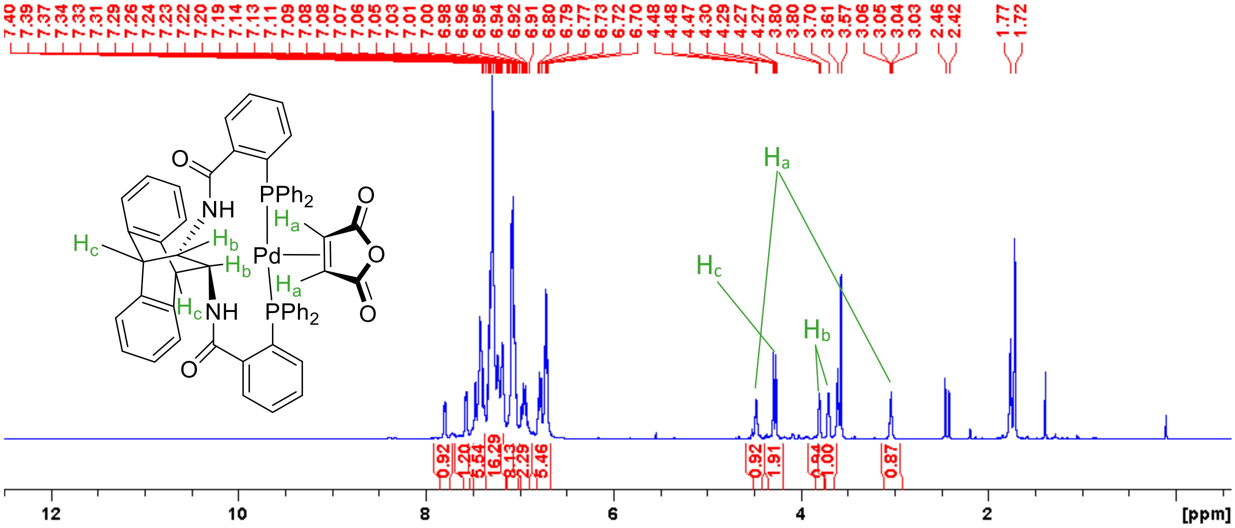

Supplementary Figure 48. ^1H NMR spectrum (500 MHz, THF-d$_8$) of **4**. Multiplets at 1.77 and 3.61 ppm are regular THF.
Singlets at 2.46 ppm and 2.42 ppm are H_2O and HDO.

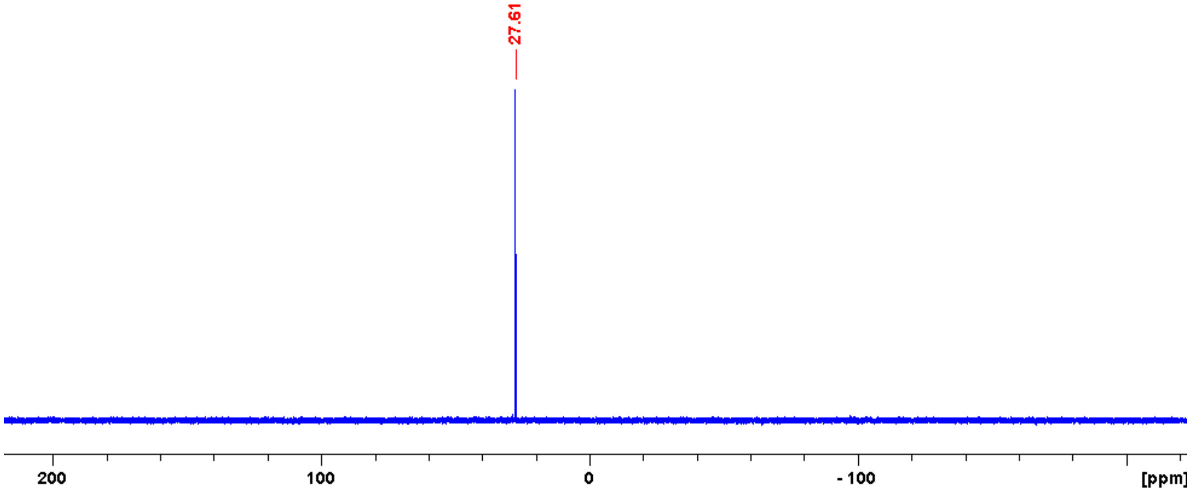

Supplementary Figure 49. $^{31}P\{^1H\}$ NMR spectrum (200 MHz, THF-d$_8$) of **4**.

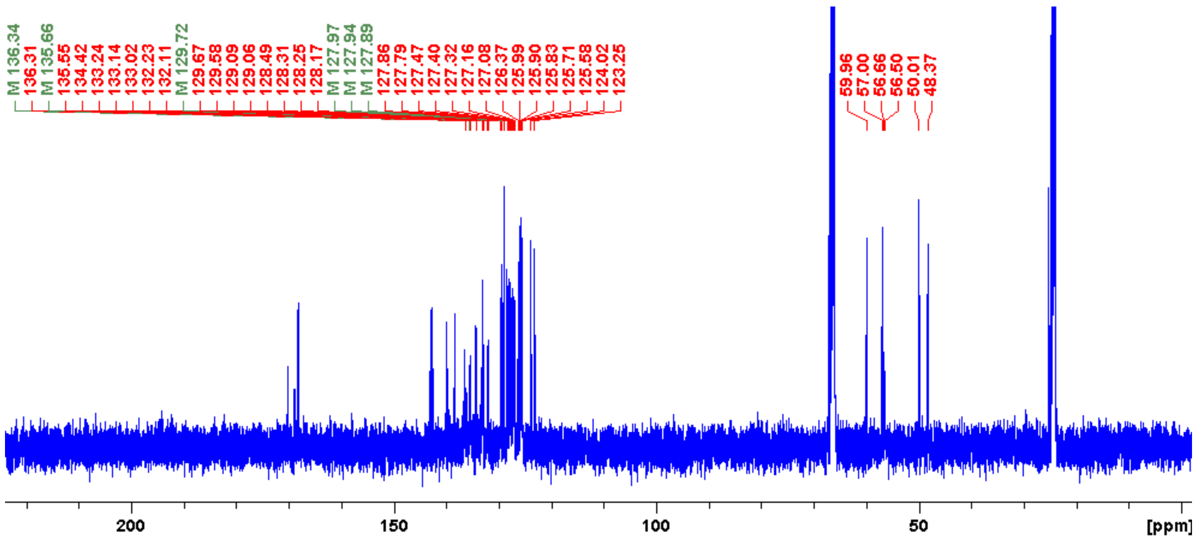

Supplementary Figure 50. $^{13}C\{^{1}H\}$ NMR spectrum (125 MHz, THF-d$_8$) of **4** (Green labels are the manually-picked peaks in multiplets).

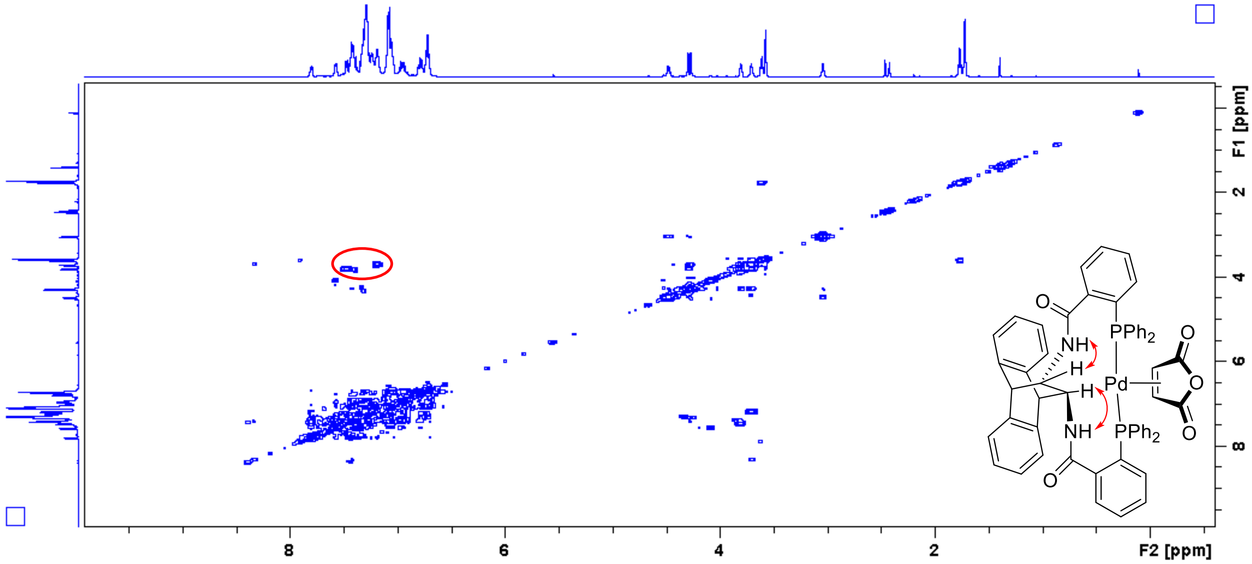

Supplementary Figure 51. ^{1}H-^{1}H COSY NMR spectrum of **4**. Determination of two NH protons (at 7.19 ppm and 7.47 ppm) according to the correlations between CH and NH.

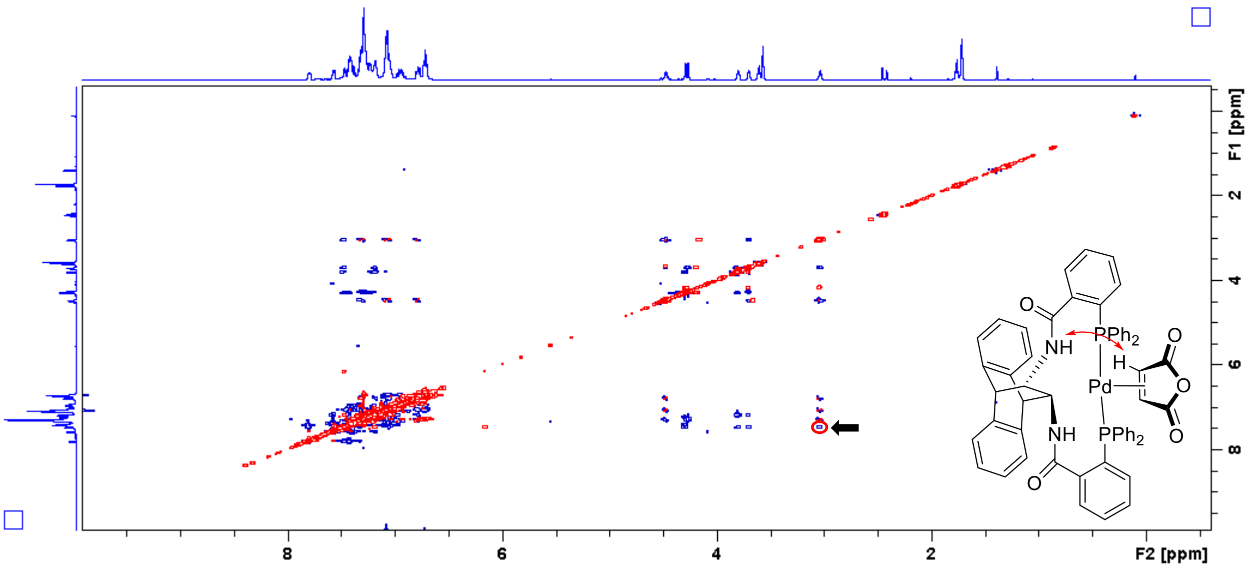

Supplementary Figure 52. ^{1}H-^{1}H NOESY NMR spectrum of **4**. The correlation between one NH and one MAH-H indicates it is the conformer without hydrogen bonding.

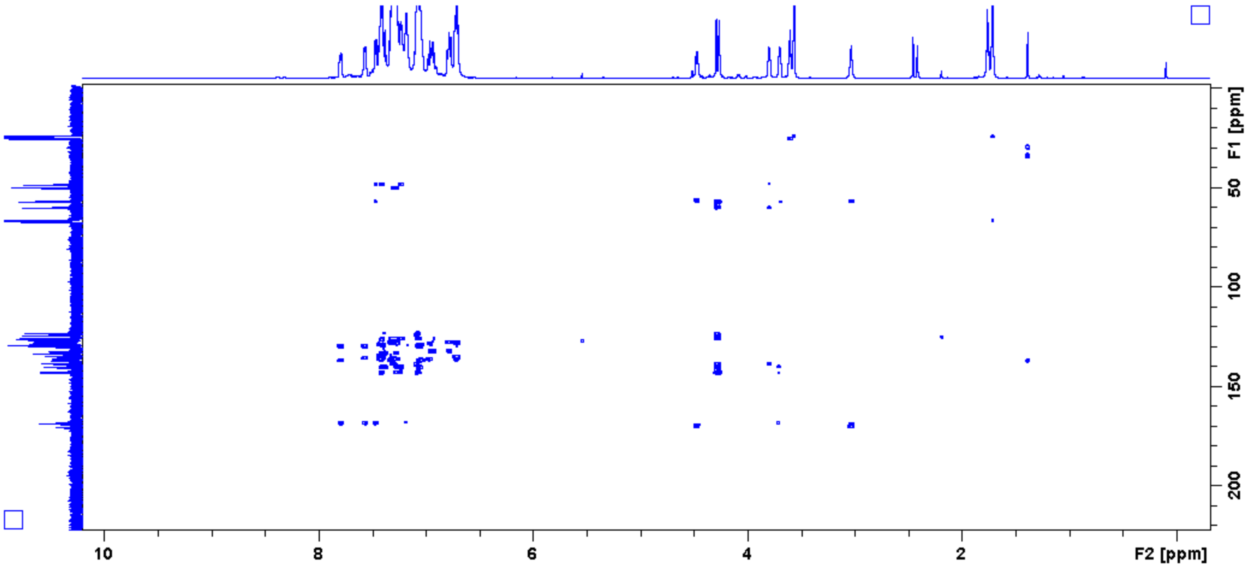

Supplementary Figure 53. ^{1}H-^{13}C HMBC NMR spectrum of **4**.

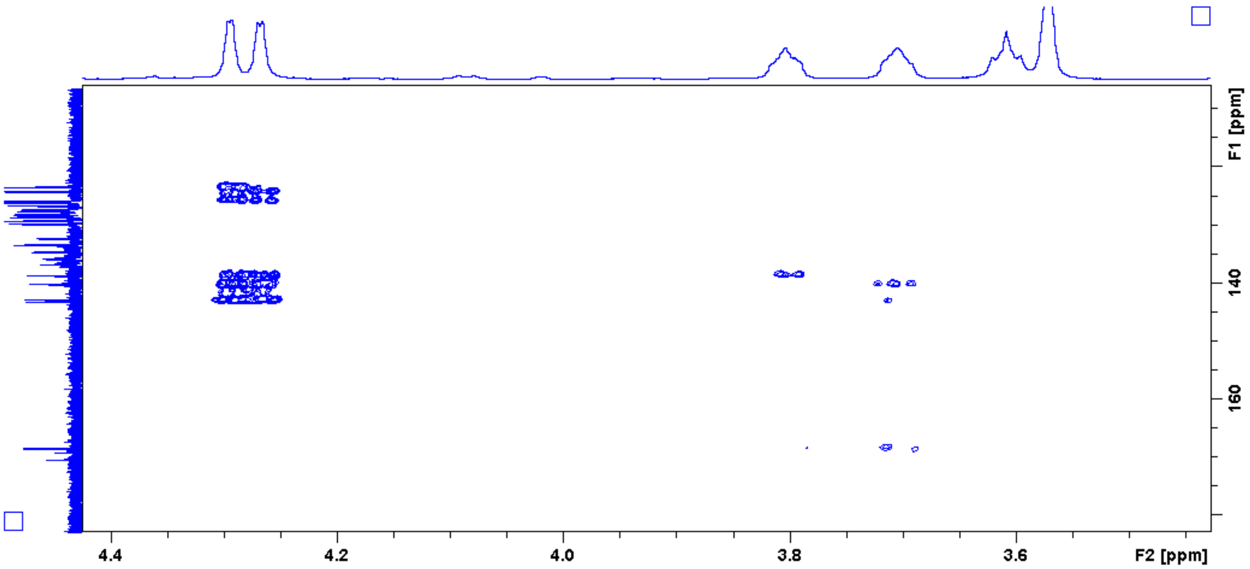

Supplementary Figure 54. ^{1}H-^{13}C HMBC NMR spectrum expansion of **4**. Differentiation of 4 × CH (C**H**-NH has weaker correlations with Ar-H but stronger correlations with C=O than the other two CH).

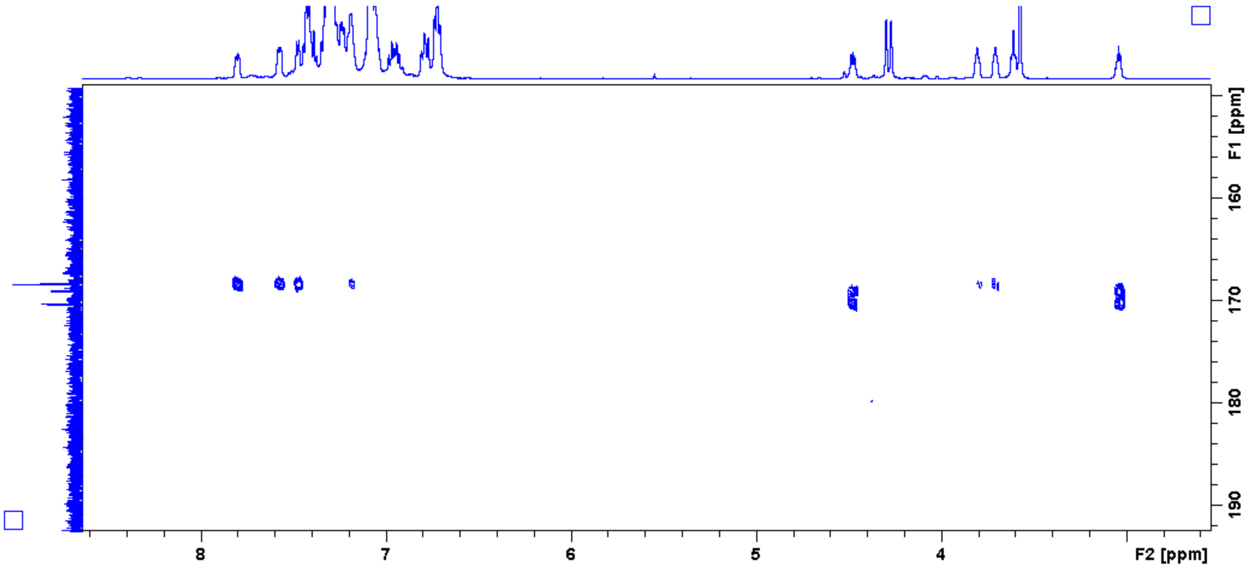

Supplementary Figure 55. ^{1}H-^{13}C HMBC NMR spectrum expansion of **4**. Differentiation between NH-CO and C=O of MAH.

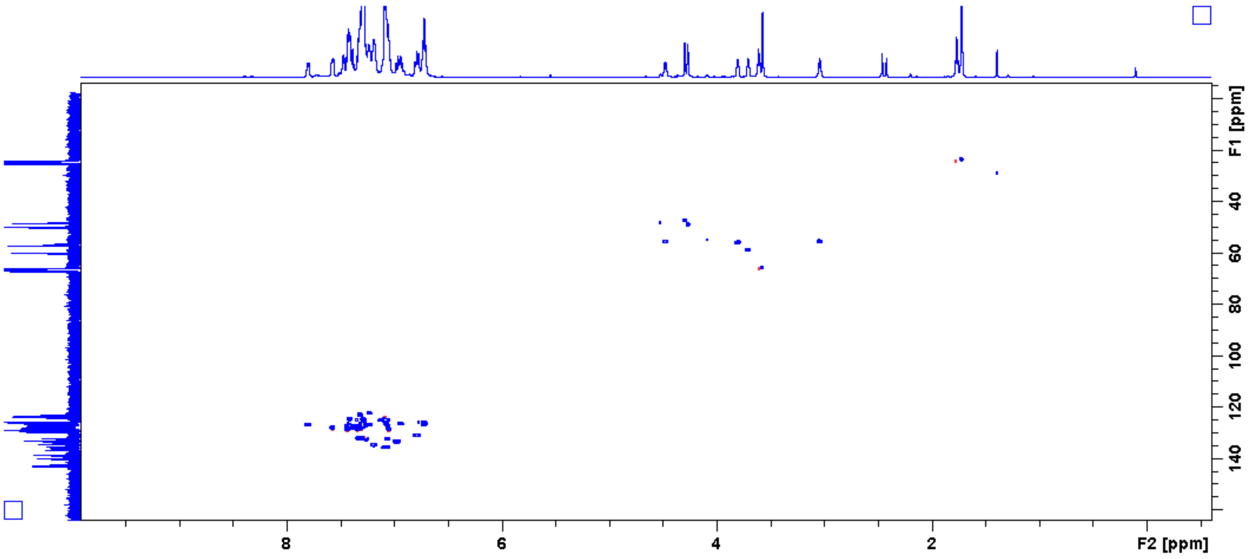

Supplementary Figure 56. ^{1}H-^{13}C HSQC NMR spectrum of **4**.

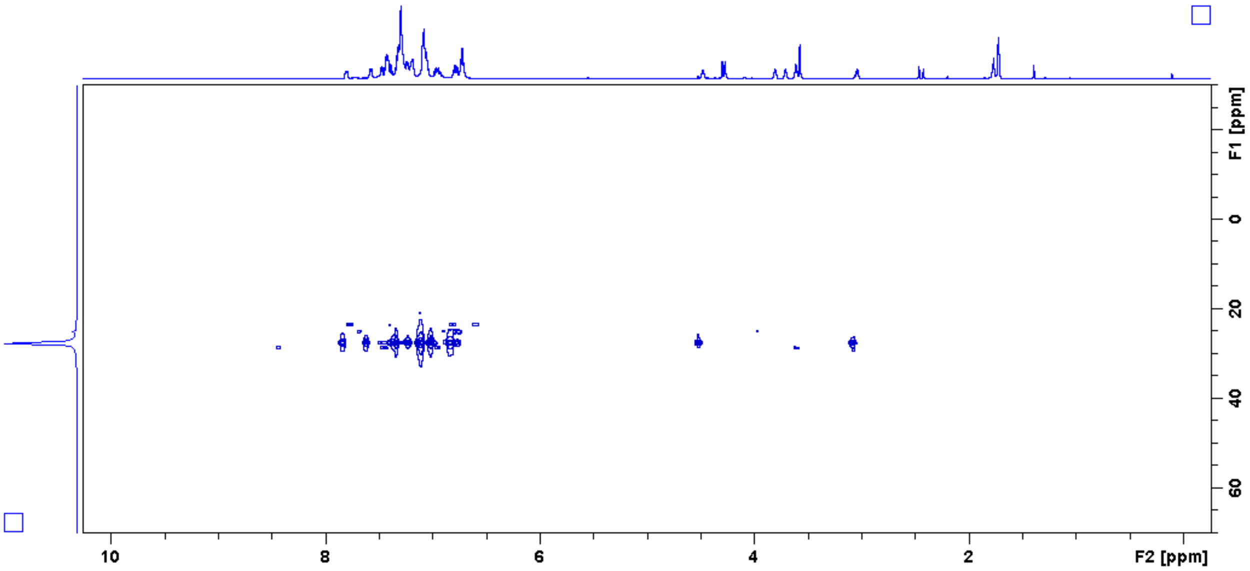

Supplementary Figure 57. ^{1}H-^{31}P HMBC NMR spectrum of **4**.

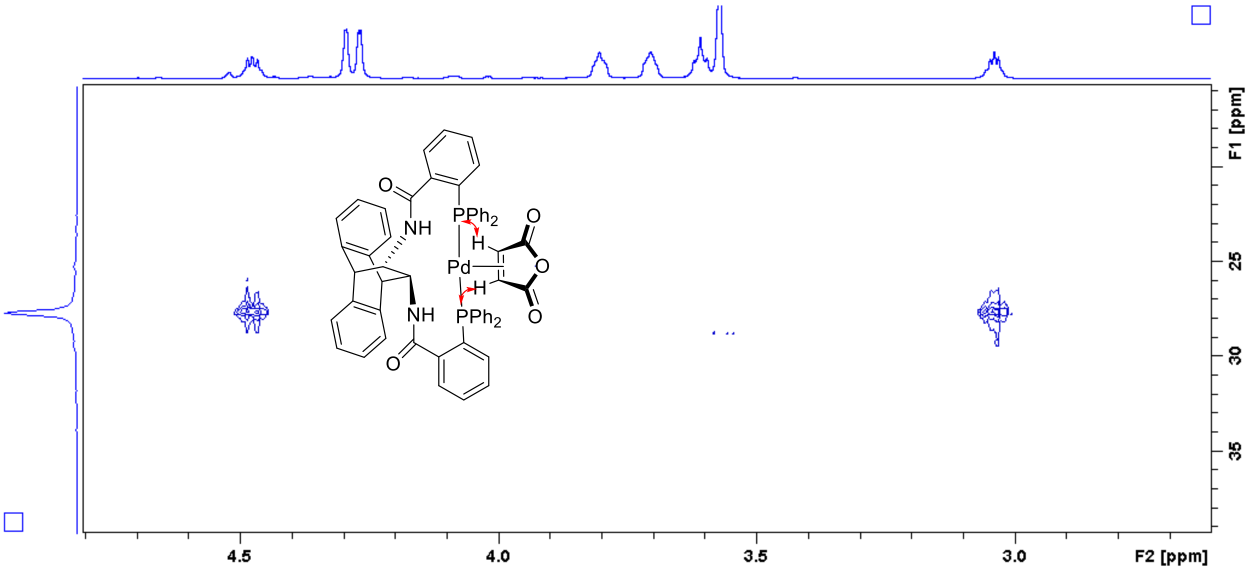

Supplementary Figure 58. ^{1}H-^{31}P HMBC NMR spectrum expansion of **4**. Identification of MAH-H.

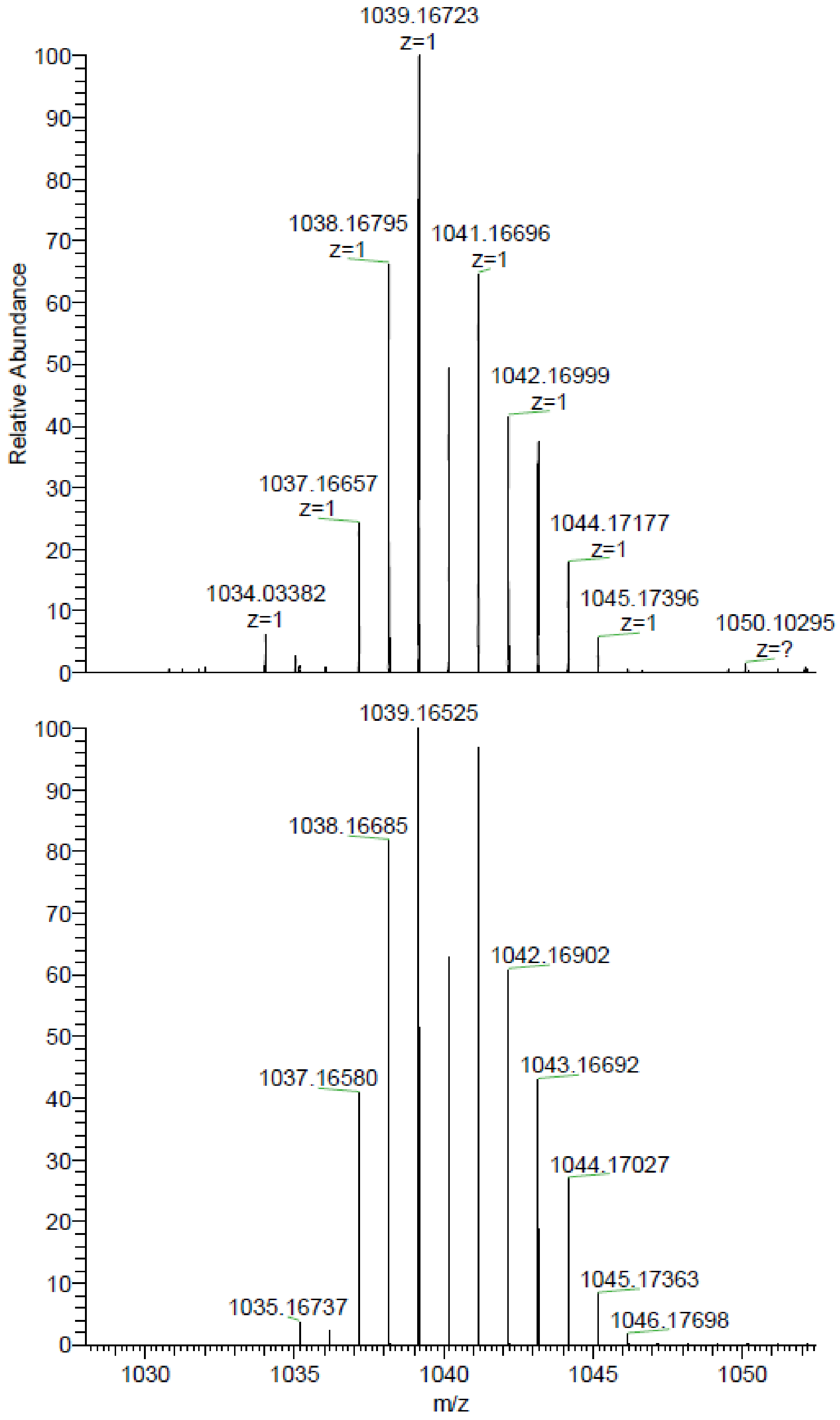

Supplementary Figure 59. Top: Experimental HRMS-ESI spectrum of [**4**·Na]$^+$. Bottom: Calculated HRMS isotope pattern for [**4**·Na]$^+$.

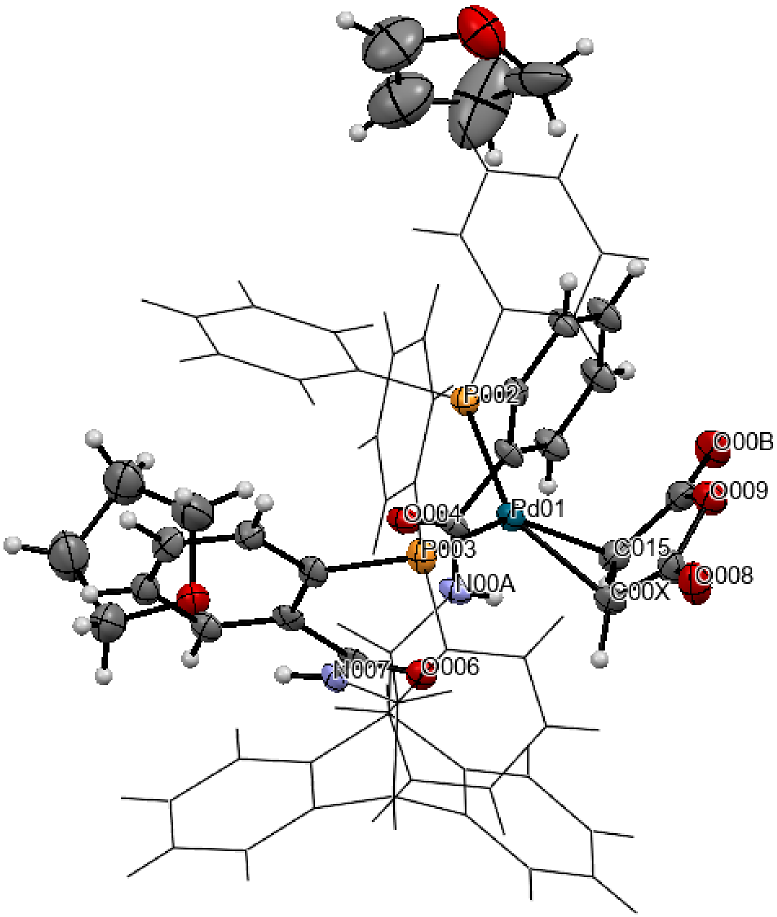

Supplementary Figure 60. Solid-state molecular structure of complex **4**, including 2 THF solvate. Thermal ellipsoids plotted at 50% probability for non-H atoms; H-atoms shown as uniformly-sized white spheres for clarity; phenyl rings and 9,10-dihyro-9,10-ethanoanthracene shown as wireframe for clarity. X-ray diffraction data collected at 100 K. Selected bond lengths (Å) and angles (°): Pd01–P002: 2.3368(18); Pd01–P003: 2.3453(17); Pd1–C015: 2.124(7); Pd1–C00X: 2.107(7); C00X–C015: 1.439(9); P002–Pd1–P003: 113.41(6); C00X–Pd01–C015: 39.8(2); C015–Pd01–P002: 102.97(19); C00X–Pd01–P003: 104.64(18).

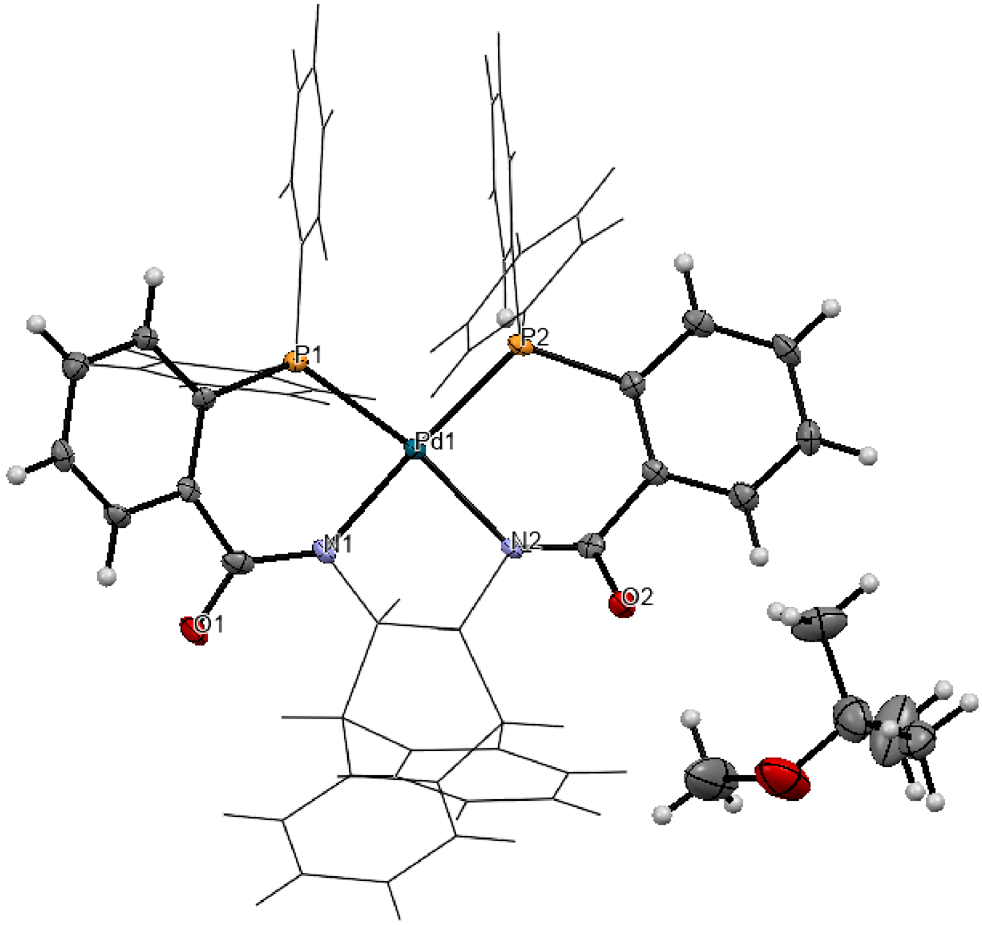

Supplementary Figure 61. Solid-state molecular structure of oxidized complex **4**, including TBME solvate. Thermal ellipsoids plotted at 50% probability for non-H atoms; H-atoms shown as uniformly-sized white spheres for clarity; phenyl rings and 9,10-dihyro-9,10-ethanoanthracene shown as wireframe for clarity. X-ray diffraction data collected at 99.93 K. Selected bond lengths (Å) and angles (°): Pd1–P1: 2.2621(16); Pd1–P2: 2.2719(18); Pd1–N1: 2.092(3);
Pd1–N2: 2.111(3); P1–Pd1–P2: 100.10(5); N1–Pd1–N2: 85.58(13); N1–Pd1–P1: 84.26(10); N2–Pd1–P2: 90.56(9).

(*S*)-^{tBu}PHOX (L5) was synthesized in three steps using adaptations to the reported procedure.[4] This includes the synthesis of 2-diphenylphosphino-benzonitrile, (+)-{(4*S*)-4-*tert*-butyl-4,5-dihydro-2-[2'-(diphenylphosphino)phenyl]oxazole}zinc(II) dichloride, and the desired (*S*)-^{tBu}PHOX ligand.

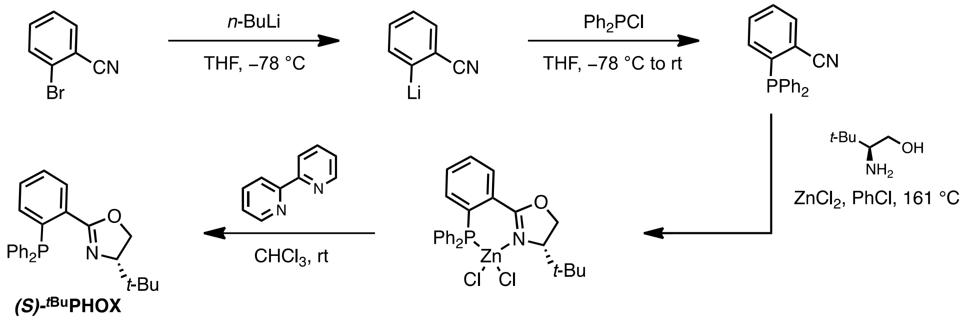

To a solution of 2-bromobenzonitrile (2.73 g, 15 mmol) in THF (30 mL) at –78 °C, was added *n*-BuLi (2.5 M in hexanes, 6 mL, 15 mmol), and the reaction stirred for 1 h. A solution of chlorodiphenylphosphine (2.83 mL, 15 mmol) in THF (5 mL) was then added, and the reaction was stirred for a further 1 h at –78 °C, before being allowed to warm to rt and stirred at that temperature for 1 h. Et_2O (80 mL) was added, and the organic layer was washed with water (70 mL) and a saturated aqueous solution of brine (70 mL). The organic layer was dried over $MgSO_4$, filtered and the solvent removed under reduced pressure. The residue was purified by recrystallization from hot methanol to recover 2-diphenylphosphino-benzonitrile (1.39 g, 32%) as a white solid.

To a solution of 2-diphenylphosphino-benzonitrile (820 mg, 2.85 mmol) in chlorobenzene (12 mL) under a N_2 atmospher was added $ZnCl_2$ (499 mg, 3.66 mmol) and L-*tert*-leucinol (423 mg, 3.61 mmol), and the reaction heated at 160 °C for 4.5 days. The reaction was passed through a pad of silica and washed with EtOAc (600 mL) and the solvent removed under reduced pressure. The residue was purified by recrystallisation from chloroform/TBME to recover (+)-{(4*S*)-4-*tert*-butyl-4,5-dihydro-2-[2'-(diphenylphosphino)-phenyl]oxazole}zinc(II) (317 mg, 21%) as a white solid.

To a solution of (+)-{(4*S*)-4-*tert*-butyl-4,5-dihydro-2-[2'-(diphenylphosphino)phenyl]oxazole}zinc(II) (221 mg, 0.42 mmol) in $CHCl_3$ (4 mL) was added 2,2’-bypiridine (65 mg, 0.42 mmol) and the reaction was stirred at rt for 4 h. The crude reaction mixture was passed through a pad of silica and washed with $CHCl_3$ (50 mL), and the solvent was removed under reduced pressure to recover the (*S*)-^{tBu}PHOX (**L5**) (140 mg, 86%) as a colorless and oily product. The NMR data are consistent with the literature.[4]

^{1}H NMR (300 MHz, CD_2Cl_2) δ 7.90 (m, 1H), 7.38-7.17 (m, 12H), 6.90 (m, 1H), 4.13 (dd, 1H), 4.02 (t, 1H), 3.85 (dd, 1H), 0.76 (s, 9H).

^{31}P NMR (121 MHz, CD_2Cl_2) δ –7.03

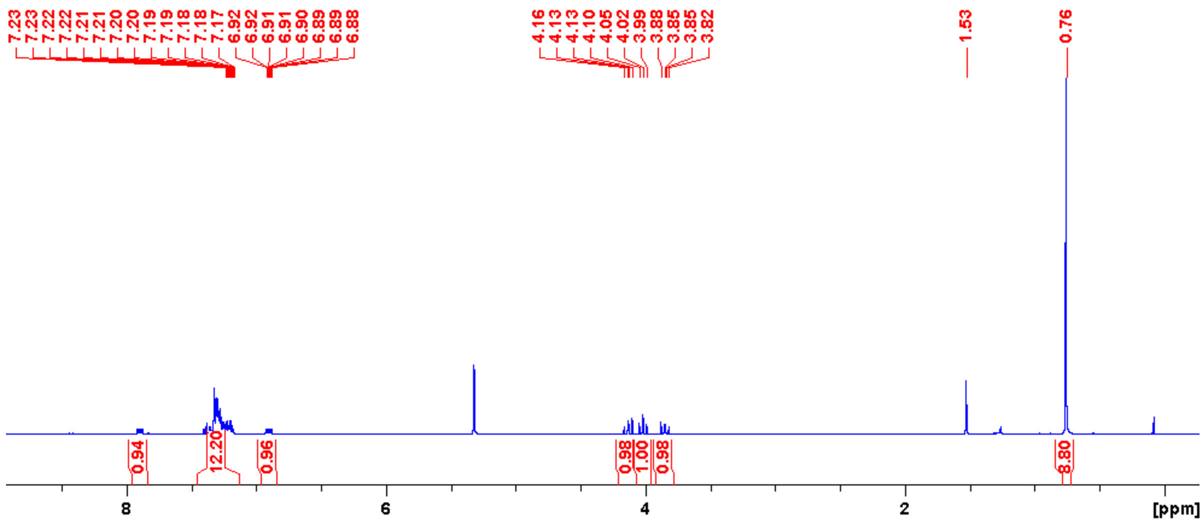

Supplementary Figure 62. ^{1}H NMR spectrum (300 MHz, CD_2Cl_2) of (*S*)-^{tBu}PHOX (**L5**).

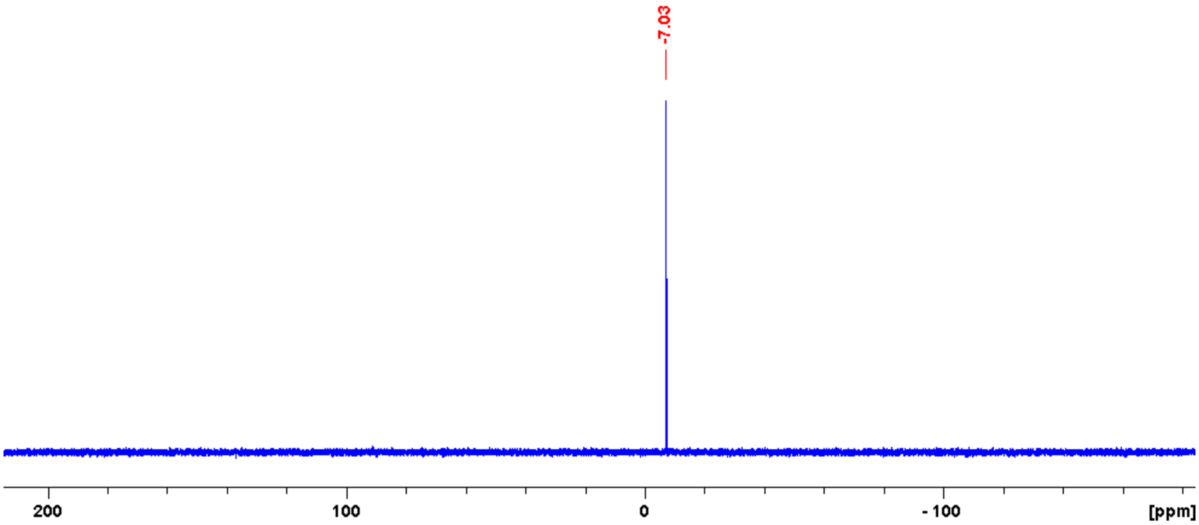

Supplementary Figure 63. $^{31}P\{^{1}H\}$ NMR spectrum (121 MHz, CD_2Cl_2) of (*S*)-^{tBu}PHOX (**L5**).

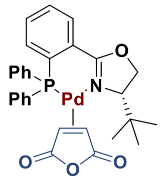

^{tBu}PHOX-Pd-MAH

(*S*)-^{tBu}PHOX-Pd-MAH (5) – The entire procedure was performed open to air. A 4-dram vial was charged with ^{DMP}DAB-Pd-MAH (167 mg, 0.36 mmol), (*S*)-^{tBu}PHOX (140 mg, 0.36 mmol), and 8 mL of THF. The solution was stirred at rt for 3 h. The reaction mixture was then filtered through a pad of Celite© followed by solvent evaporation. The crude product then underwent trituration/ decantation with hexane until the washing was colorless (8 times). The product was filtered and dried overnight to give a light brown solid (192 mg, 90%).

^{1}H NMR (500 MHz, CD_2Cl_2): *Minor conformer* **δ** 8.18-8.15 (m, 1H), 7.56-7.17 (m, 12H), 6.98-6.92 (m, 1H), 4.44-4.36 (m, 2H), 4.17-4.12 (m, 1H), 4.07 (dd, *J* = 9.3, 4.3 Hz, 1H), 3.78-3.81 (m, 1H), 0.62 (s, 9H). *Major conformer* **δ** 8.18-8.15 (m, 1H), 7.56-7.17 (m, 12H), 6.98-6.92 (m, 1H), 4.44-4.36 (m, 2H), 4.23 (t, 1H), 4.17-4.12 (m, 1H), 3.81-3.78 (m, 1H), 0.66 (s, 9H).

^{13}C NMR (125 MHz, CD_2Cl_2) δ 173.0, 172.9, 171.8 (d, $^3J_{C\text{-}P}$ = 5.3 Hz), 171.4 (d, $^3J_{C\text{-}P}$ = 5.3 Hz), 164.6, 164.0, 134.6, 134.5, 134.4, 134.3, 134.1, 133.8, 133.7, 133.6, 133.5, 132.7, 132.6_3, 132.5_6 (d, *J* = 1.8 Hz), 132.4_8 (d, *J* = 3.5 Hz), 132.4_3, 132.4_0, $132,3_7$, 130.9 (d, *J* = 1.8 Hz), 130.8 (d, *J* = 1.8 Hz), 130.6 (d, *J* = 1.8 Hz), 130.5, 130.4_9 (d, *J* = 2.6 Hz), 130.4, 129.1, 129.0 (m), 128.9 (d, *J* = 2.6 Hz), 128.8, 128.7, 80.2 (d, $^3J_{C\text{-}P}$ = 1.8 Hz), 79.9 (d, $^3J_{C\text{-}P}$ = 1.7 Hz), 68.9, 68.8, 48.5 (d, $^2J_{C\text{-}P}$ = 32.6 Hz), 47.2, 46.6_9 (d, $^2J_{C\text{-}P}$ = 33.5 Hz), 45.7_3, 34.4, 25.3, 24.5.

^{31}P NMR (200 MHz, CD_2Cl_2) δ 22.29 (major conformer), 23.12 (minor conformer).

HRMS (ESI): m/z calcd for $C_{29}H_{29}NO_4PPd$, $[M + H]^+$ (major isotopomer): 592.0864, found: 592.0863.

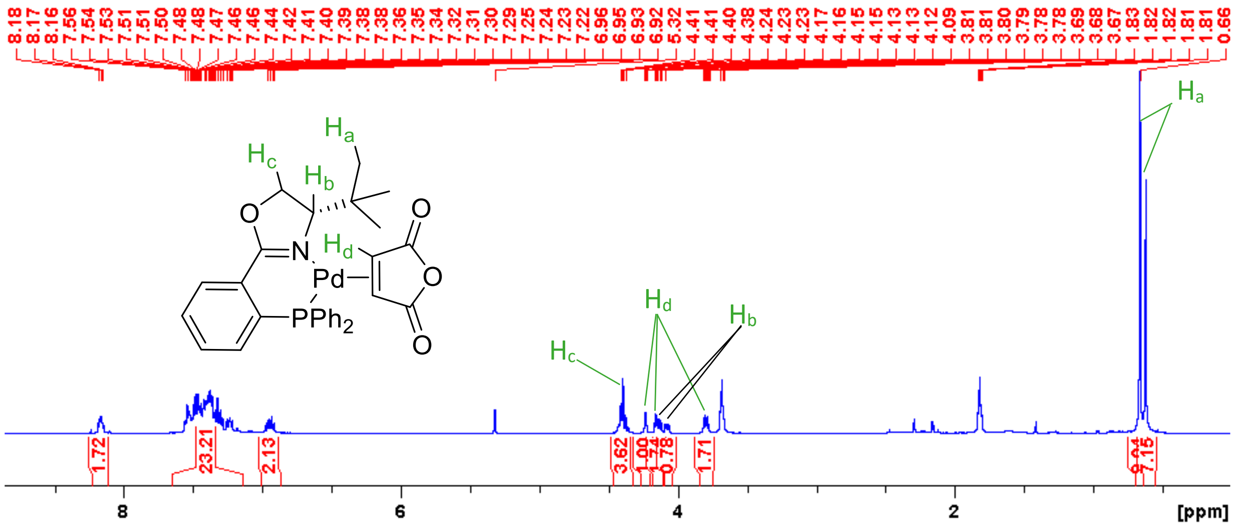

Supplementary Figure 64. ^{1}H NMR spectrum (500 MHz, CD_2Cl_2) of **5**. Two conformers (MAH up and down) are observed, and key proton signals are assigned. Singlet at 5.32 ppm is CD_2Cl_2 residual signal. Multiplets at 1.82 and 3.68 ppm are THF.

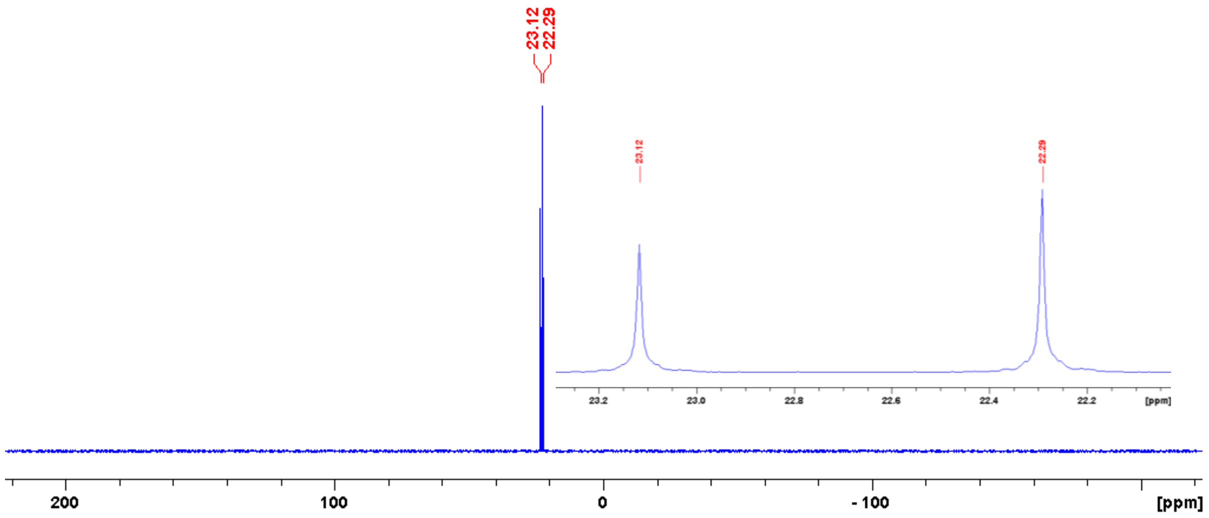

Supplementary Figure 65. $^{31}P\{^{1}H\}$ NMR spectrum (200 MHz, CD_2Cl_2) of **5**. Two conformers are observed.

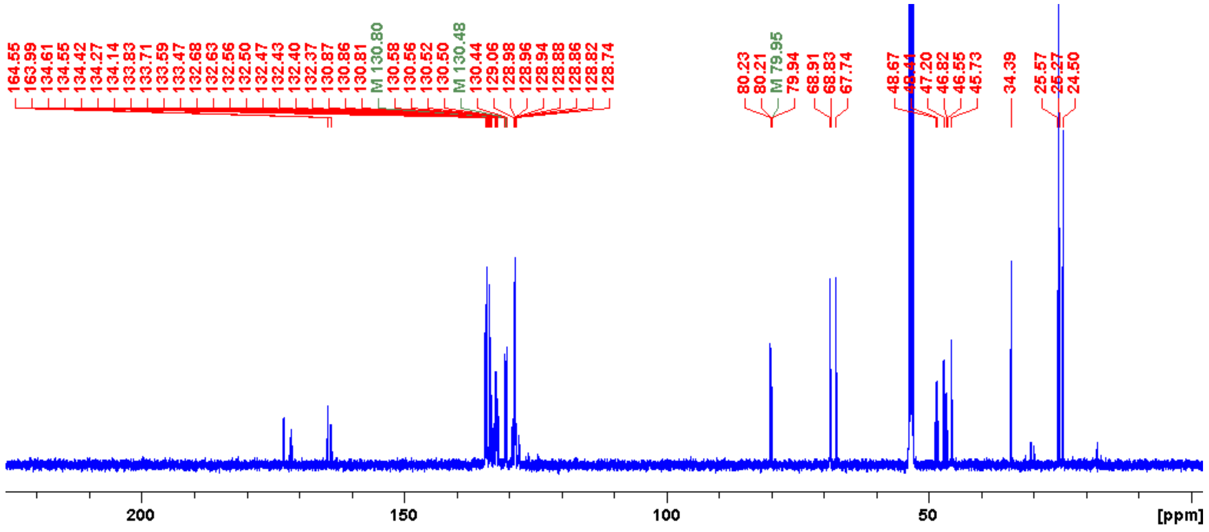

Supplementary Figure 66. $^{13}C\{^{1}H\}$ NMR spectrum (125 MHz, CD_2Cl_2) of **5** (Green labels are the manually-picked peaks).

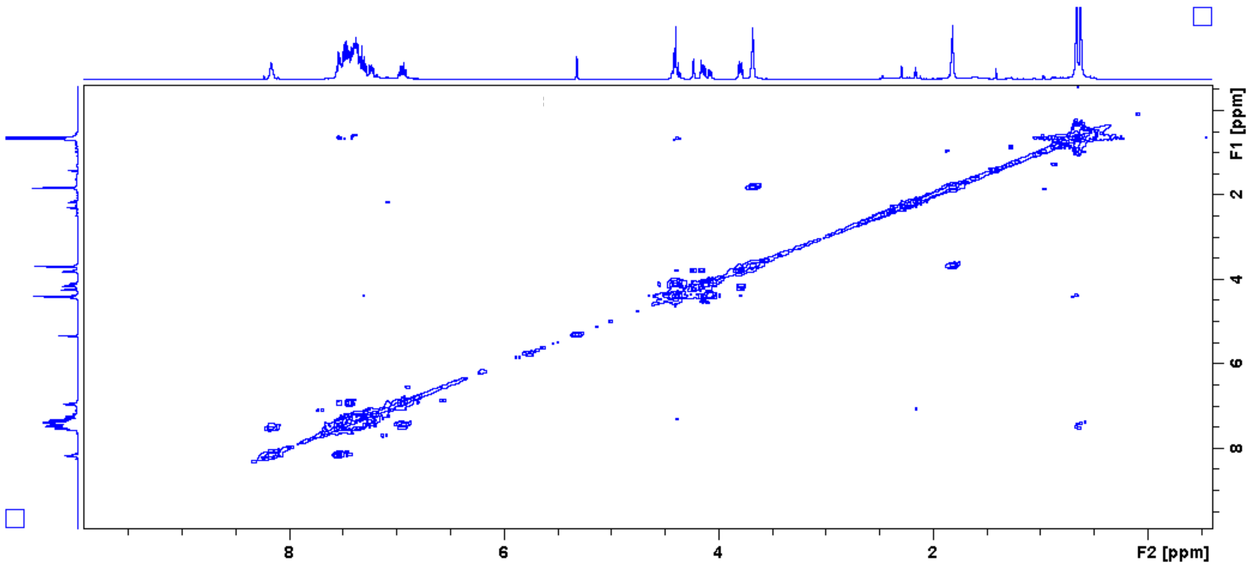

Supplementary Figure 67. ^{1}H-^{1}H COSY NMR spectrum of **5**.

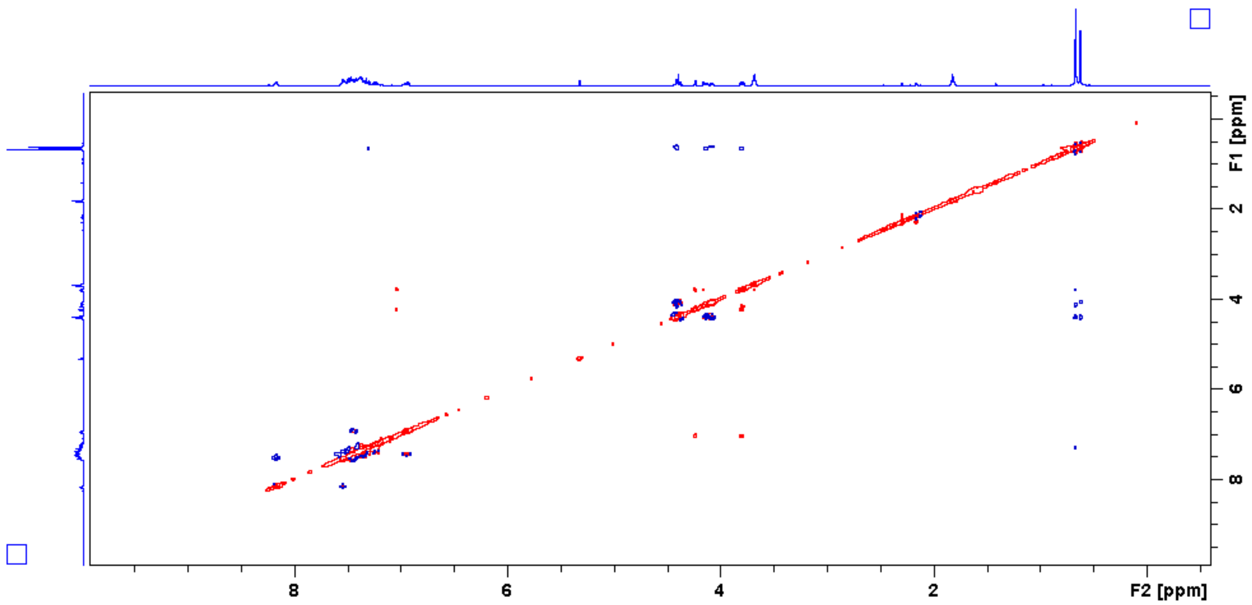

Supplementary Figure 68. ^1H-^1H NOESY NMR spectrum of **5**.

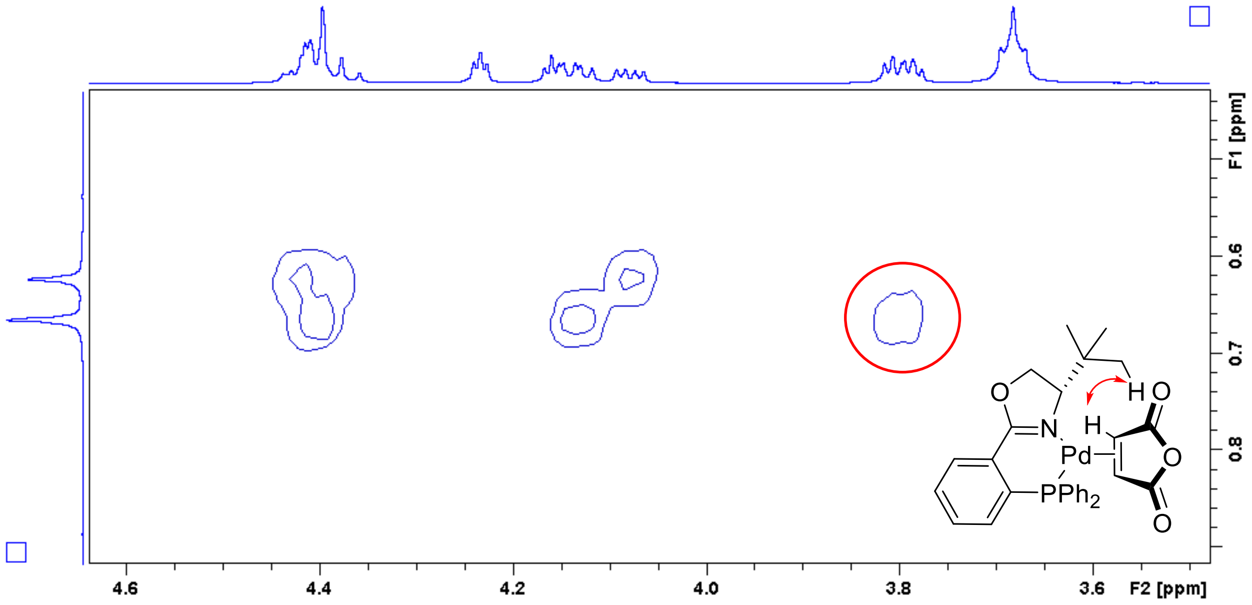

Supplementary Figure 69. ^{1}H-^{1}H NOESY NMR spectrum of **5**. The correlation between MAH-H and CH_3 indicates that the major conformer has MAH and *tert*-butyl group *exo* to one another.

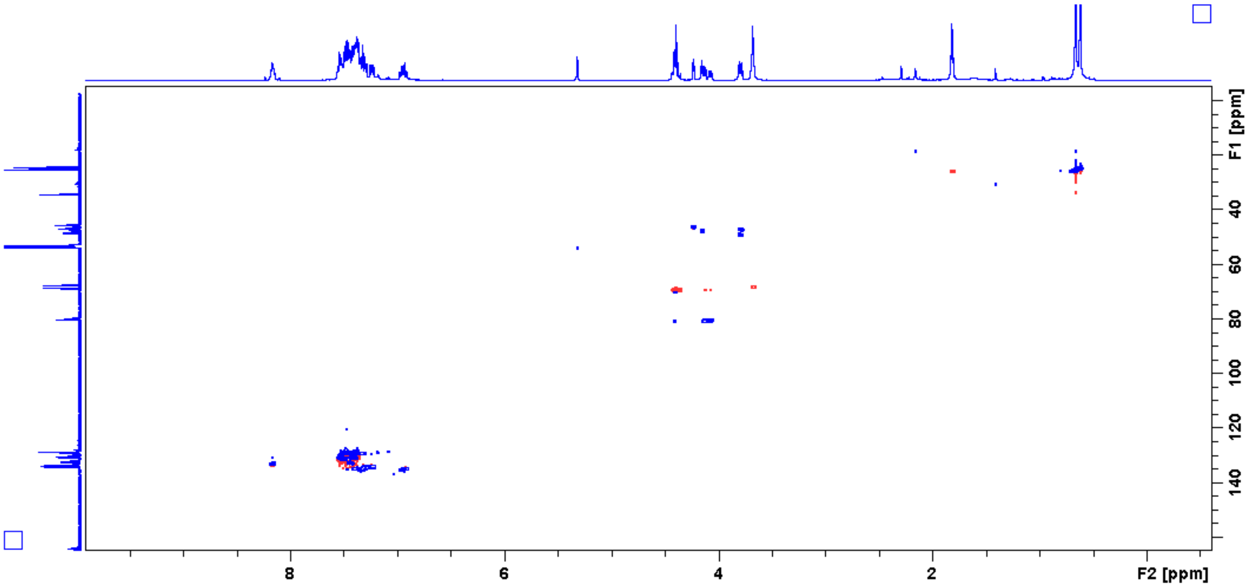

Supplementary Figure 70. ^{1}H-^{13}C HSQC NMR spectrum of **5**.

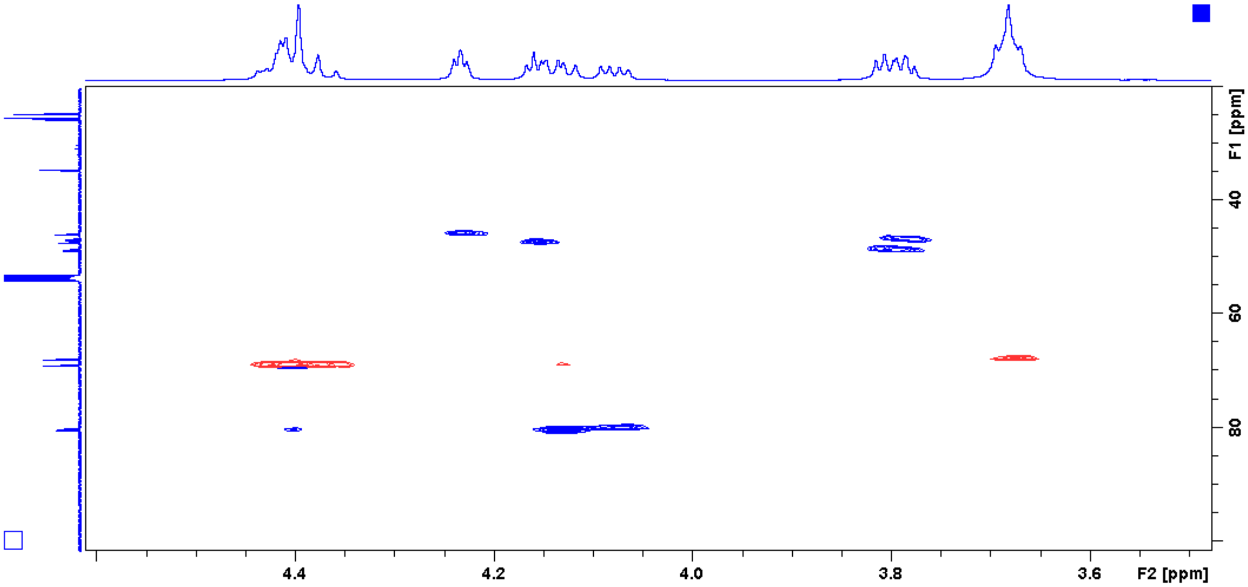

Supplementary Figure 71. ^{1}H-^{13}C HSQC NMR spectrum expansion of **5**. Differentiation of CH_2 and CH.

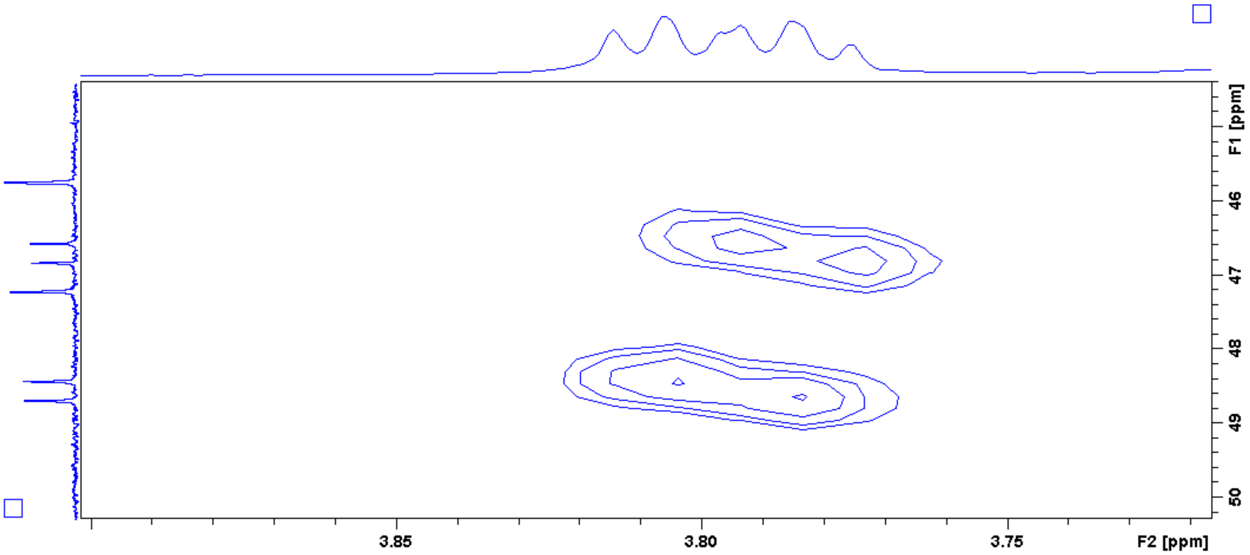

Supplementary Figure 72. ^{1}H-^{13}C HSQC NMR spectrum expansion of **5**. C-H correlations of the two alkene carbons of MAH.

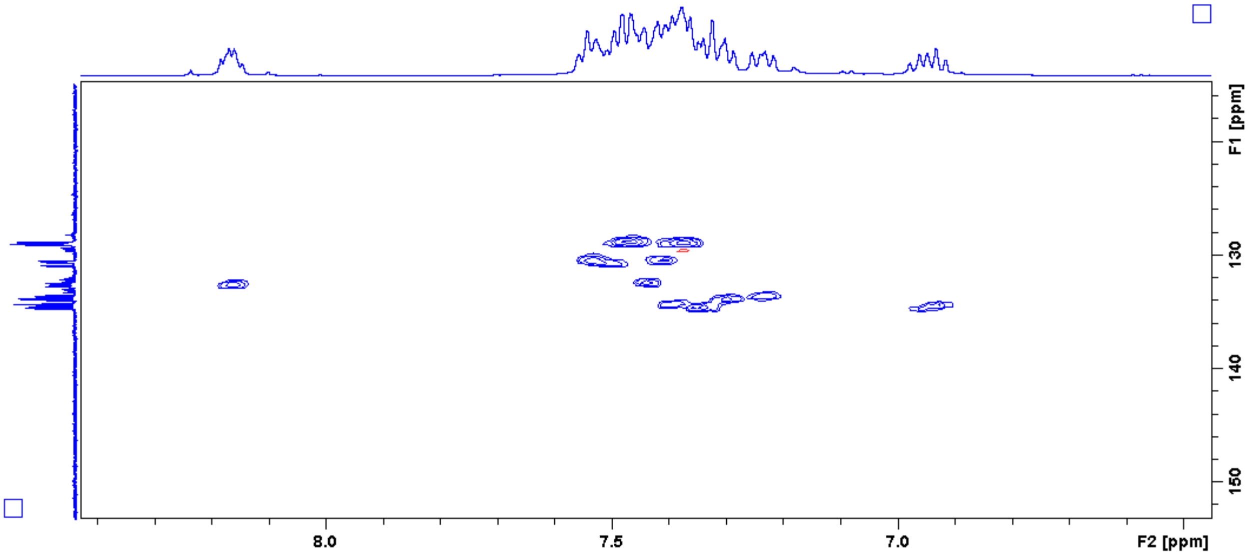

Supplementary Figure 73. ^{1}H-^{13}C HSQC NMR spectrum of **5**. Aliphatic region expansion.

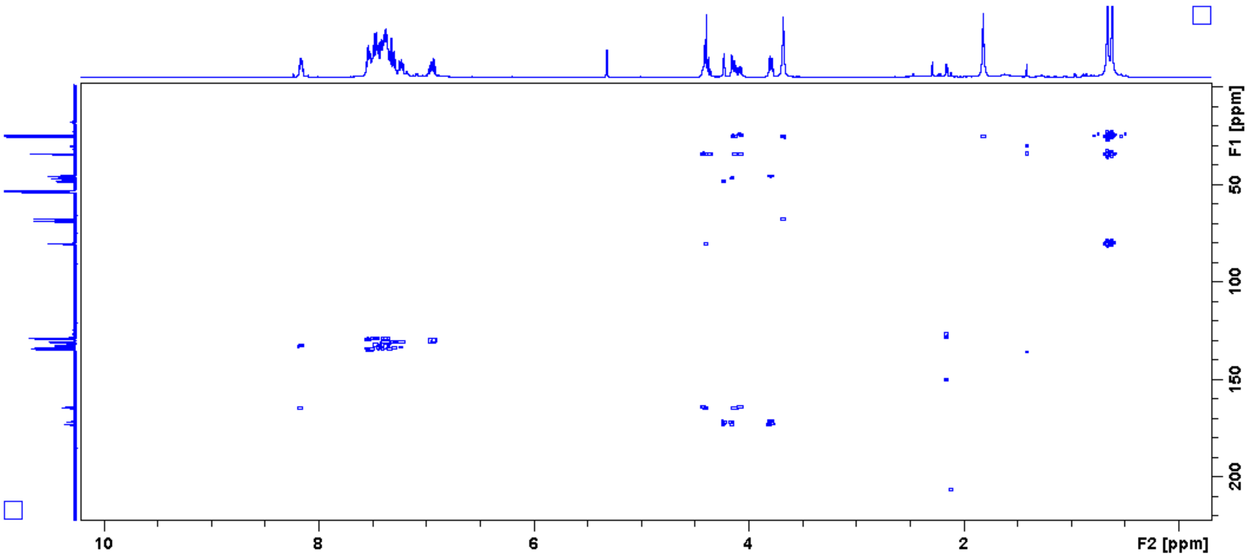

Supplementary Figure 74. ^{1}H-^{13}C HMBC NMR spectrum of **5**.

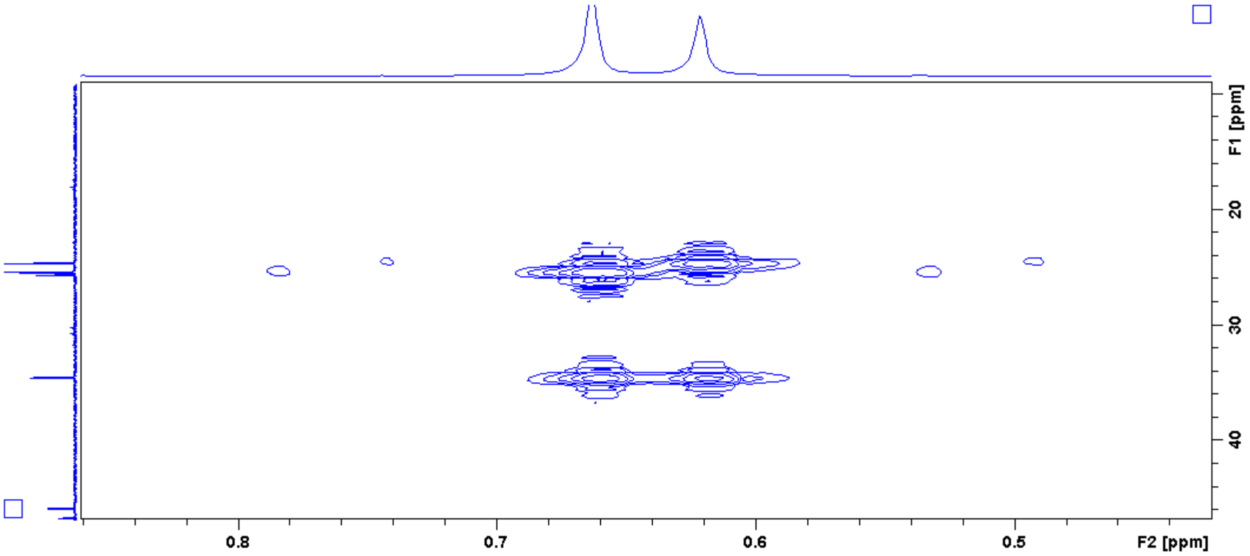

Supplementary Figure 75. ^{1}H-^{13}C HMBC NMR spectrum expansion of **5**. Assignment of C(CH$_3$)$_3$.

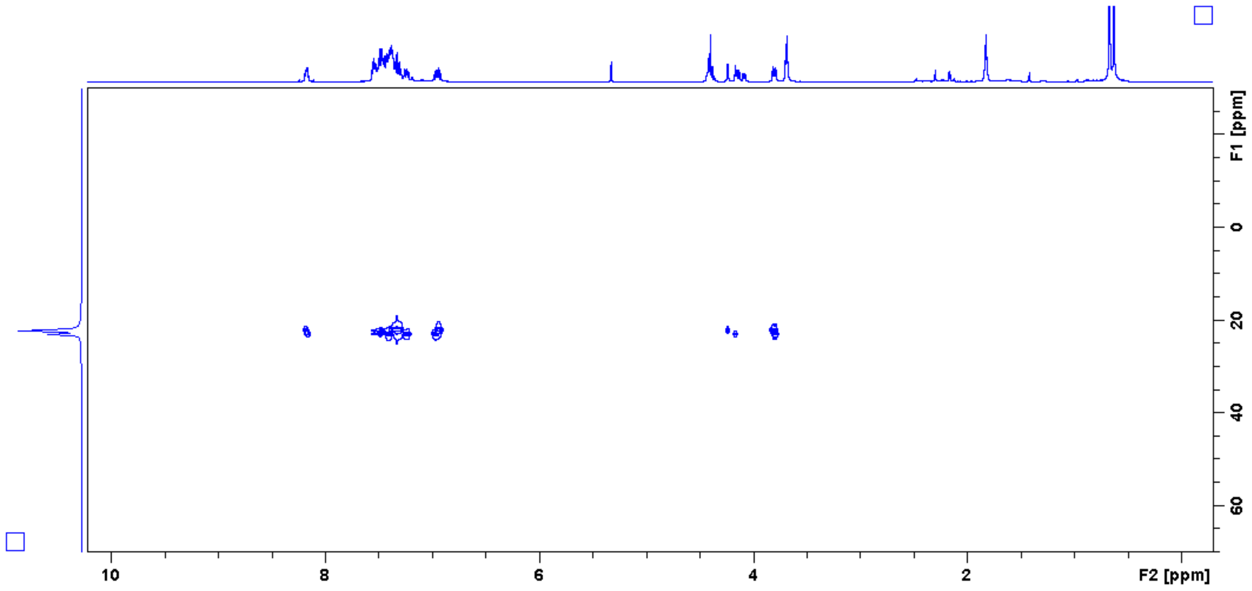

Supplementary Figure 76. ^{1}H-^{31}P HMBC NMR spectrum expansion of **5**.

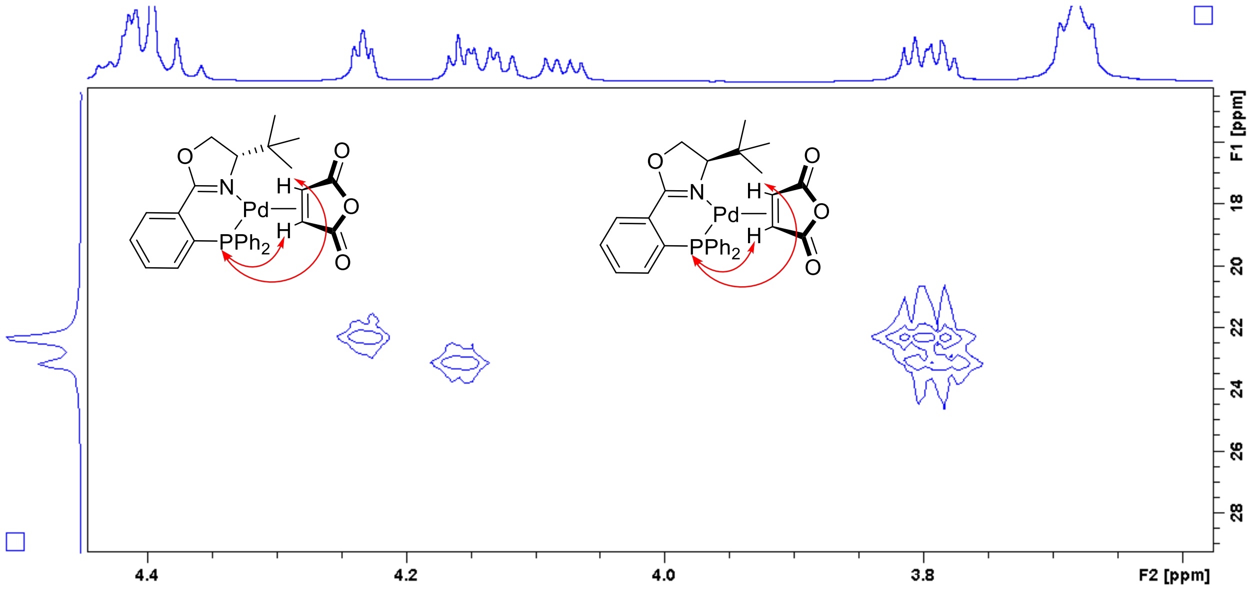

Supplementary Figure 77. ^{1}H-^{31}P HMBC NMR spectrum expansion of **5**. The correlations between P and MAH-H are observed.

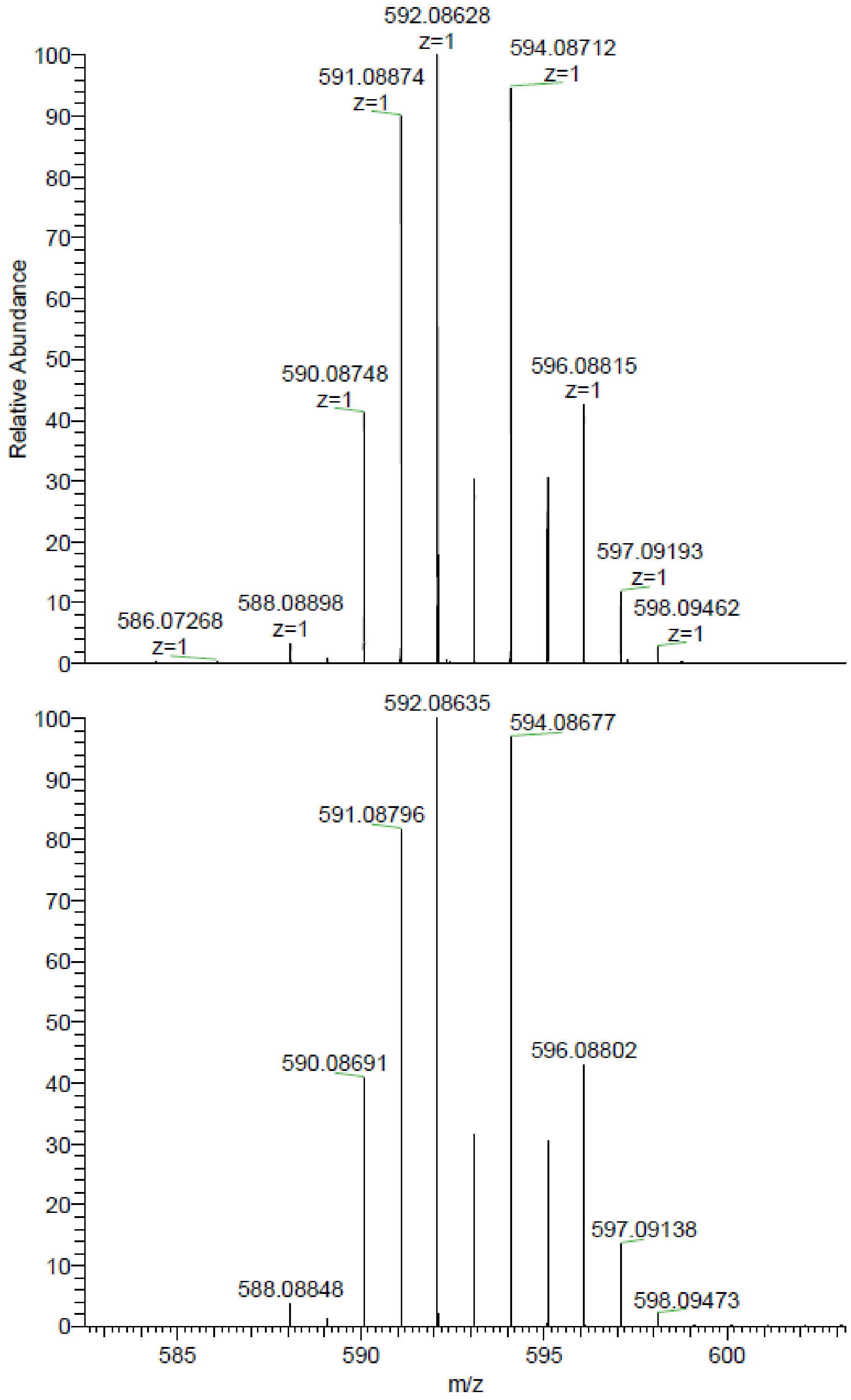

Supplementary Figure 78. Top: Experimental HRMS-ESI spectrum of [**5**·H]$^+$. Bottom: Calculated HRMS isotope pattern for [**5**·H]$^+$.

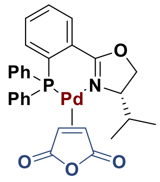

^{iPr}PHOX-Pd-MAH

(*S*)-^{iPr}PHOX-Pd-MAH (6) – The entire synthetic procedure was performed in a glovebox under a dry, oxygen-free atmosphere of nitrogen. A 4-dram vial was charged with of ^{DMP}DAB-Pd-MAH (125 mg, 0.27 mmol), (*S*)-*i*PrPHOX ligand (100 mg, 0.27 mmol), and 5 mL of THF. The reaction mixture was stirred at rt for 1 h and then filtered through a pad of Celite©. The THF was evaporated and the crude residue was triturated/decanted with hexane/Et_2O (1:1) until the washings were colourless (8 times). Residual solvents from the trituration/decantation were removed under vacuum and the product was dried overnight to give a light green solid (113 mg, 73%). The product was recrystallized from pentane/THF. Two conformers were observed in DCM.

^{1}H NMR (500 MHz, CD_2Cl_2): *minor conformer* **δ** 8.08-8.13 (m, 1H), 7.56-7.23 (m, 12H), 7.05-7.01 (m, 1H), 4.40-4.27 (m, 3H), 4.11 (m, 1H), 3.82 (m, 1H), 2.09 (m, 1H), 0.83 (d, *J* = 6.8 Hz, 3H), 0.21 (d, *J* = 6.8 Hz, 3H). *Major conformer* **δ** 8.13-8.08 (m, 1H), 7.56-7.23 (m, 12H), 7.05-7.01 (m, 1H), 4.40-4.27 (m, 3H), 4.15 (m, 1H), 3.91 (m, 1H), 2.31 (m, 1H), 0.87 (m, 3H), 0.38 (d, *J* = 6.8 Hz, 3H).

^{13}C NMR (125 MHz, CD_2Cl_2): major **δ** 172.8, 172.6, 171.9 (d, $^3J_{C\text{-}P}$ = 5.3 Hz), 171.4 (d, $^3J_{C\text{-}P}$ = 6.2 Hz), 163.7, 163.3, 134.5, 134.1 (d, $J_{C\text{-}P}$ = 5.3 Hz), 134.0 (d, $J_{C\text{-}P}$ = 3.5 Hz), 133.9, 133.8, 133.6, 133.5, 132.3, 132.2$_3$, 132.2$_2$, 132.2$_0$, 132.0$_9$, 132.0$_6$, 132.0$_4$, 132.0$_1$, 130.8 (d, $J_{C\text{-}P}$ = 1.7 Hz), 130.7 (d, $J_{C\text{-}P}$ = 1.7 Hz), 130.6 (d, $J_{C\text{-}P}$ = 1.8 Hz), 130.5$_3$ (d, $J_{C\text{-}P}$ = 1.8 Hz), 130.4$_9$, 129.1, 129.0, 128.9$_3$ (d, $J_{C\text{-}P}$ = 3.6 Hz), 128.8$_7$ (d, $J_{C\text{-}P}$ = 1.8 Hz), 128.8$_5$, 128.8$_0$, 75.6 (d, $^4J_{C\text{-}P}$ = 1.8 Hz), 75.5, 67.7$_6$, 67.7$_5$, 47.1, 46.8 (d, $^2J_{C\text{-}P}$ = 4.3 Hz), 46.3, 46.0 (d, $^2J_{C\text{-}P}$ = 5.4 Hz), 31.0, 30.8, 18.4, 13.9, 13.3.

^{31}P NMR (200 MHz, CD_2Cl_2) δ 21.68 (major conformer), 22.29 (minor conformer).

HRMS (ESI): m/z calcd for $C_{28}H_{26}NO_4PPd$, $[M + Na]^+$ (major isotopomer): 600.0526, found: 600.0525.

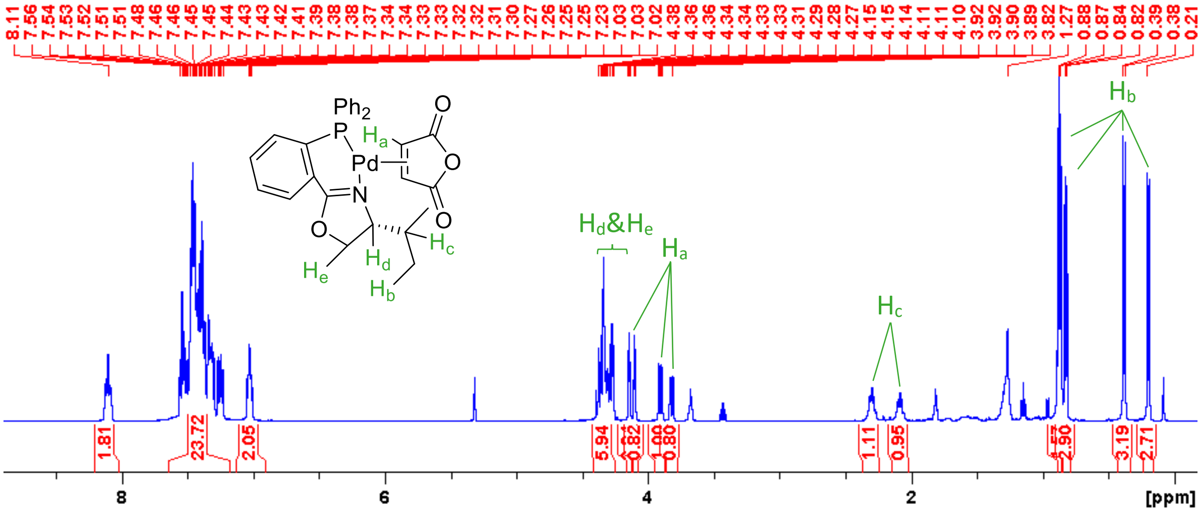

Supplementary Figure 79. ^{1}H NMR spectrum (500 MHz, CD_2Cl_2) of **6**. Two conformers (MAH *endo* and *exo*) are observed, and key proton signals are assigned. Triplet at 1.15 ppm and quartet at 3.43 ppm are diethyl ether. Multiplets at 1.82 and 3.68 ppm are THF. Multiplets at 0.88 (overlapped with one CH_3 peak) and 1.27 are hexanes.

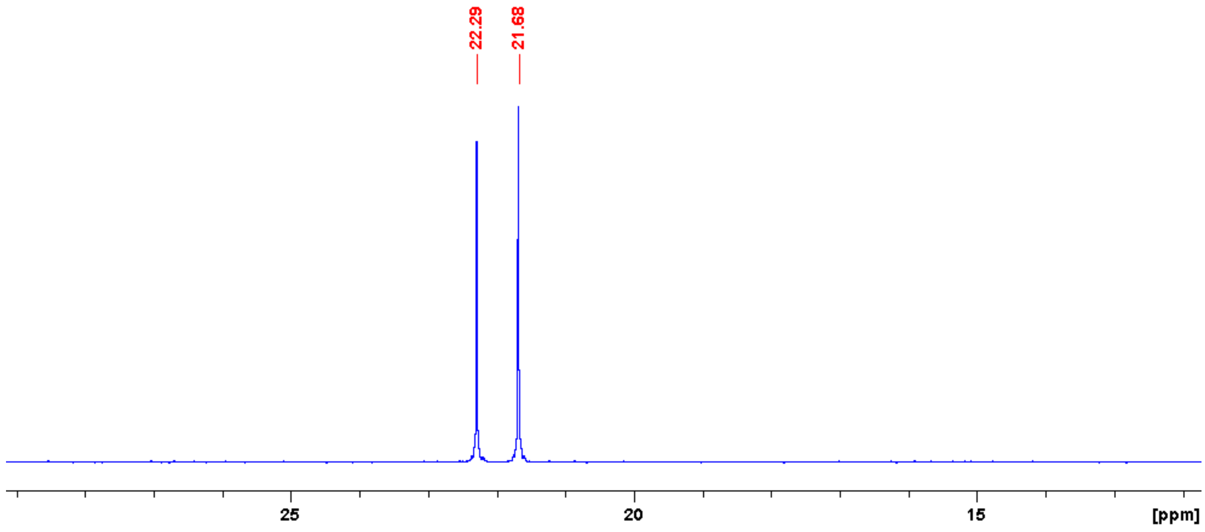

Supplementary Figure 80. $^{31}P\{^{1}H\}$ NMR spectrum (200 MHz, CD_2Cl_2) of **6**. Two conformers are observed.

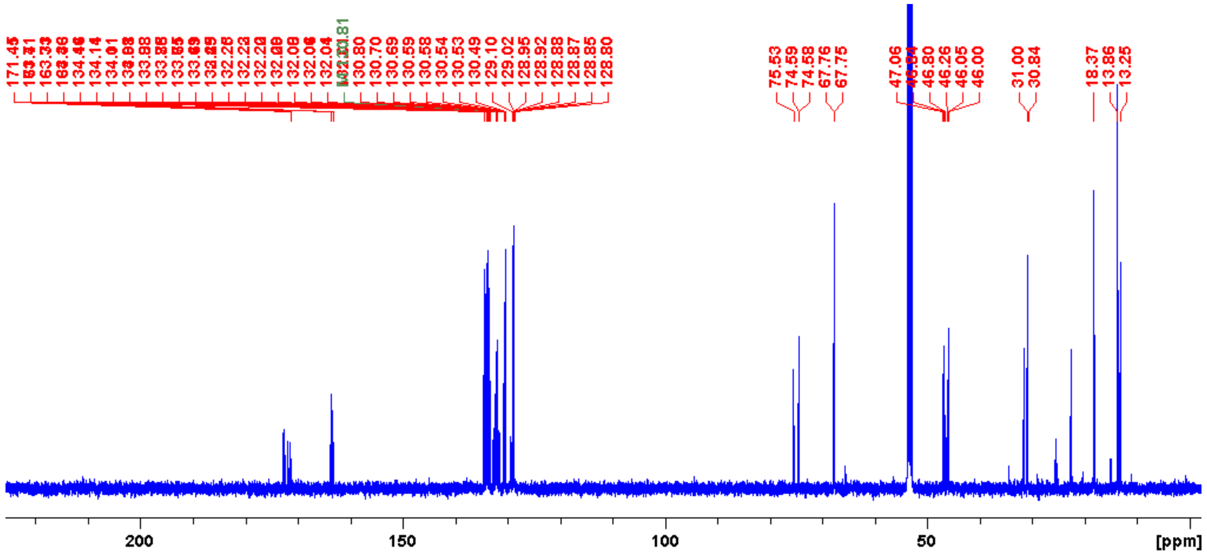

Supplementary Figure 81. $^{13}C\{^{1}H\}$ NMR spectrum (125 MHz, CD_2Cl_2) of **6** (Green label is the manually-picked peak in a doublet).

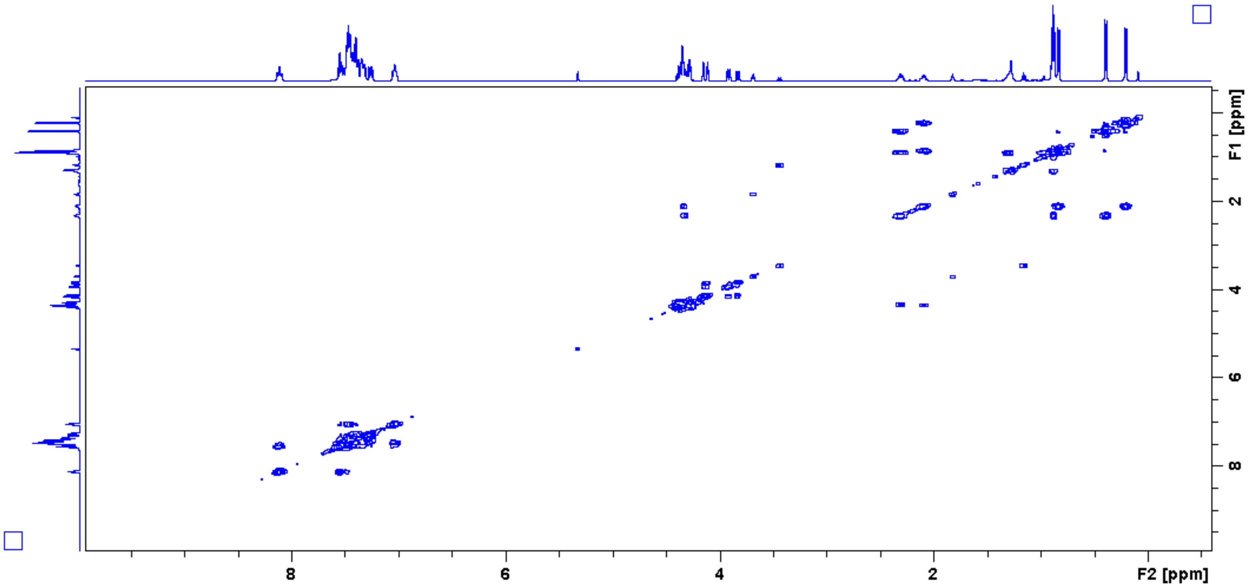

Supplementary Figure 82. ^{1}H-^{1}H COSY NMR spectrum of **6**.

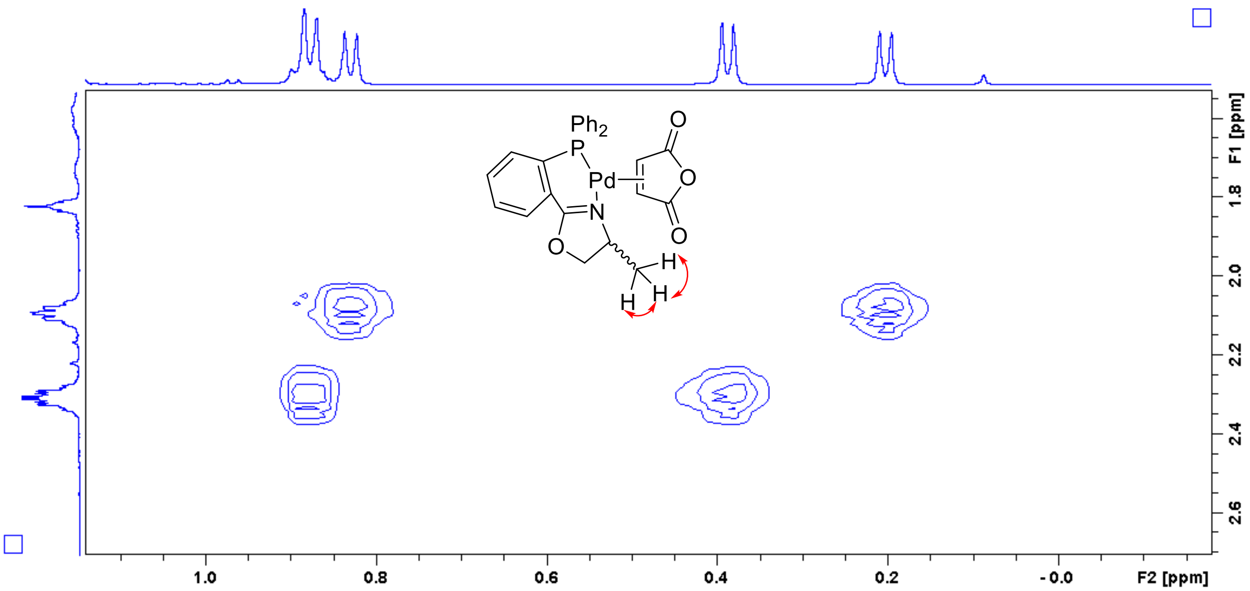

Supplementary Figure 83. ^{1}H-^{1}H COSY NMR spectrum expansion of **6**. CH_3 assignment for corresponding conformers.

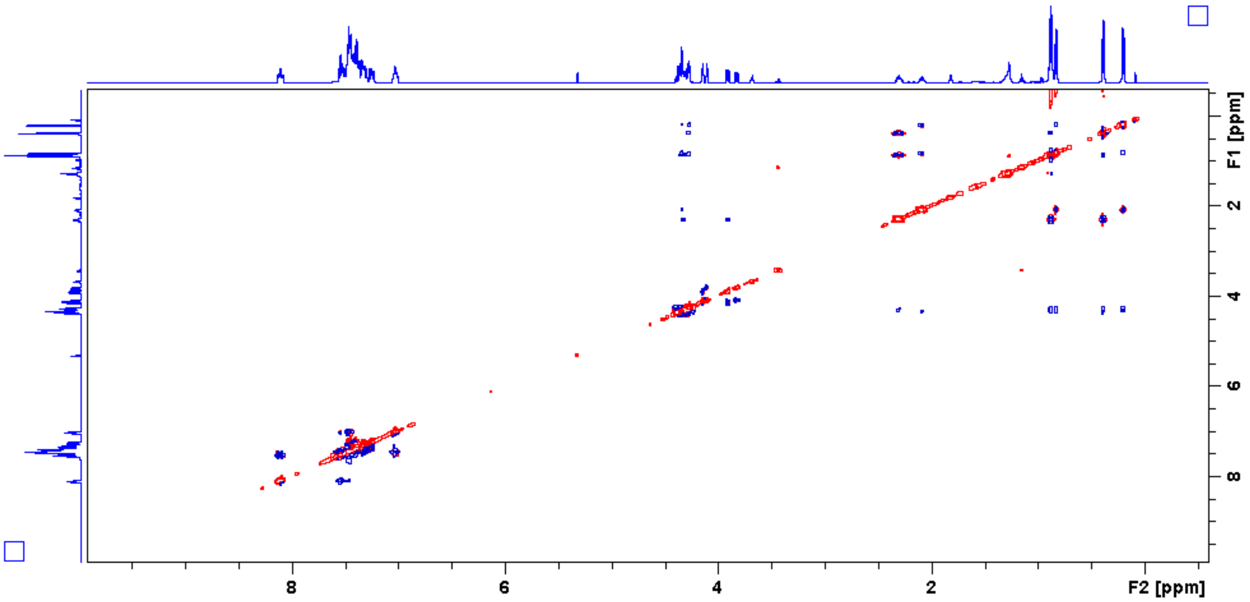

Supplementary Figure 84. ^{1}H-^{1}H NOESY NMR spectrum of **6**.

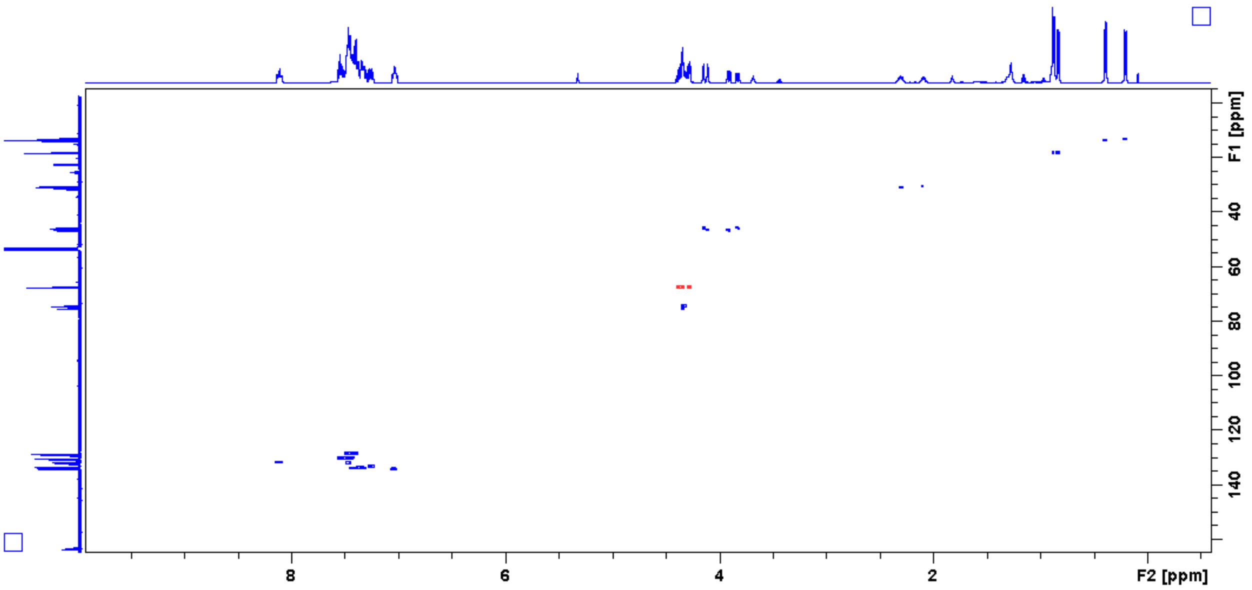

Supplementary Figure 85. ^{1}H-^{13}C HSQC NMR spectrum of **6**.

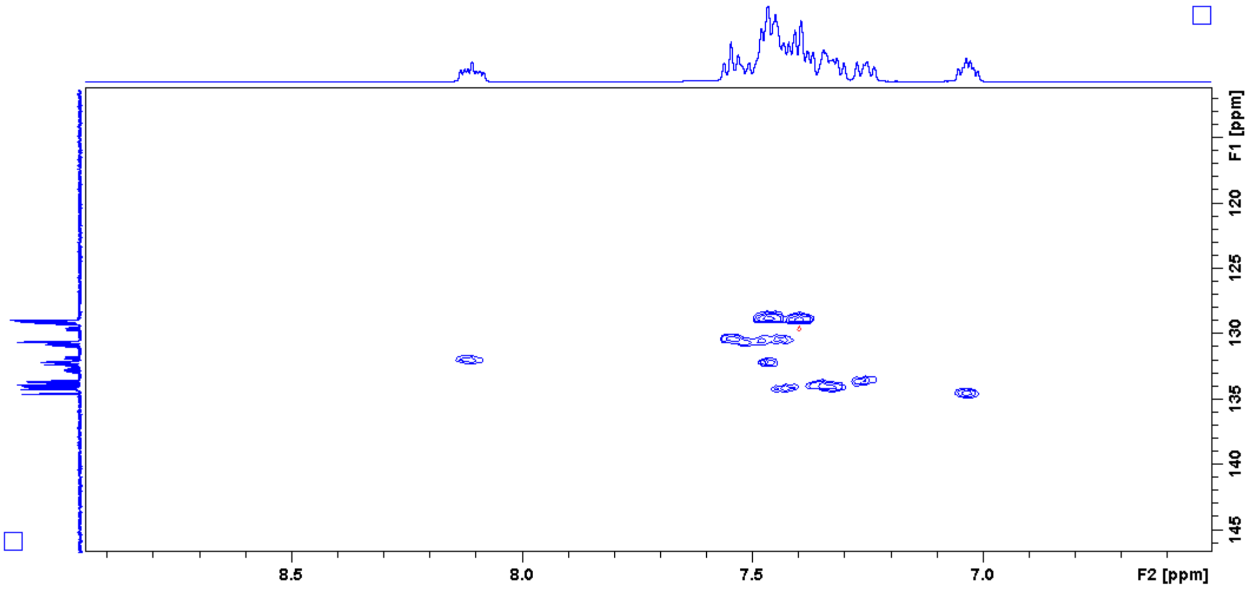

Supplementary Figure 86. ^{1}H-^{13}C HSQC NMR spectrum of **6**. Expansion of the aliphatic region.

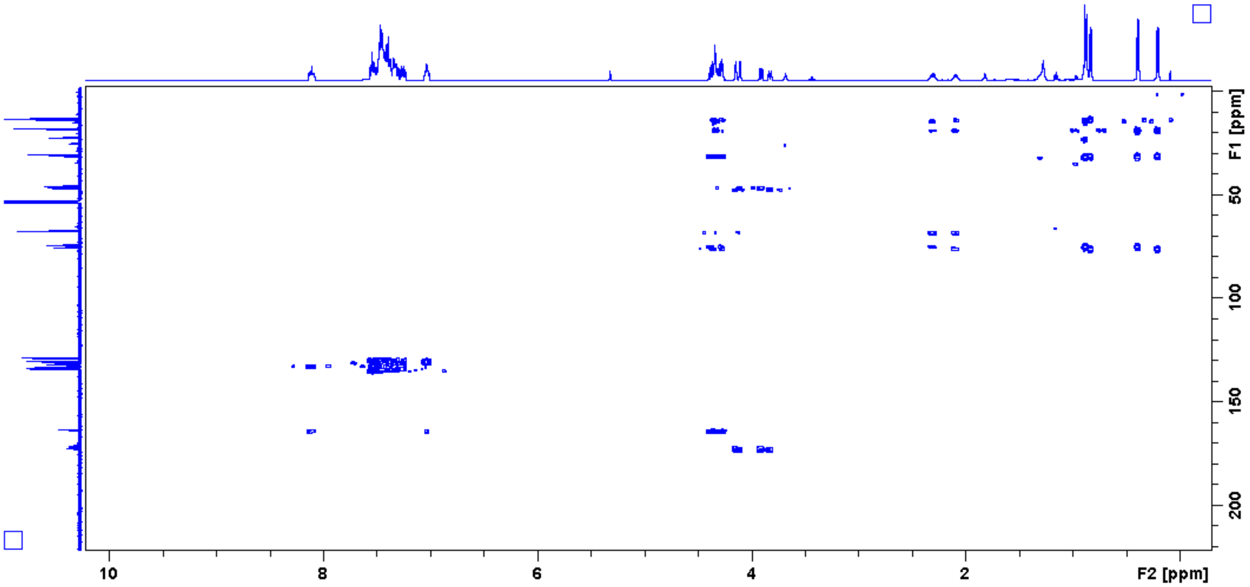

Supplementary Figure 87. ^{1}H-^{13}C HMBC NMR spectrum of **6**.

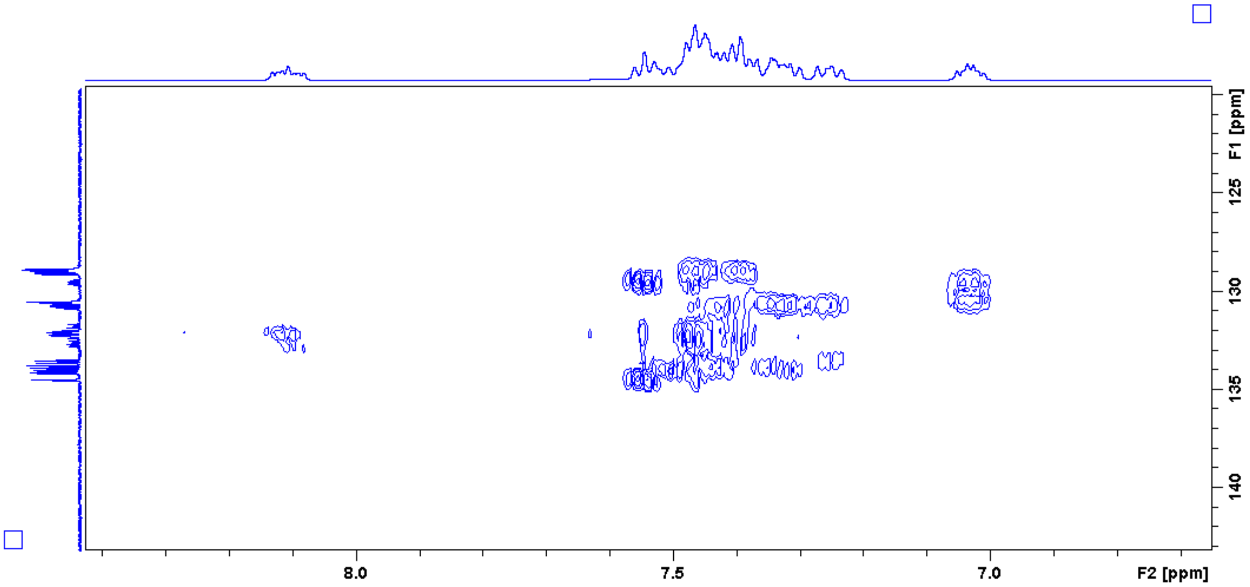

Supplementary Figure 88. ^{1}H-^{13}C HMBC NMR spectrum of **6**. Expansion of aliphatic region.

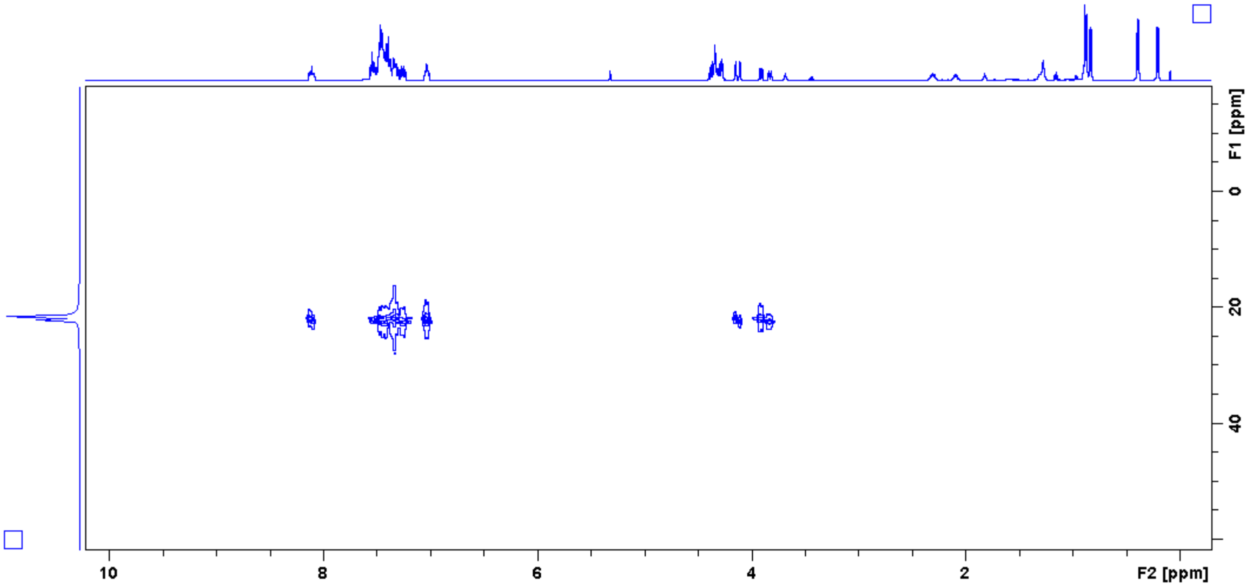

Supplementary Figure 89. ^{1}H-^{31}P HMBC NMR spectrum of **6**.

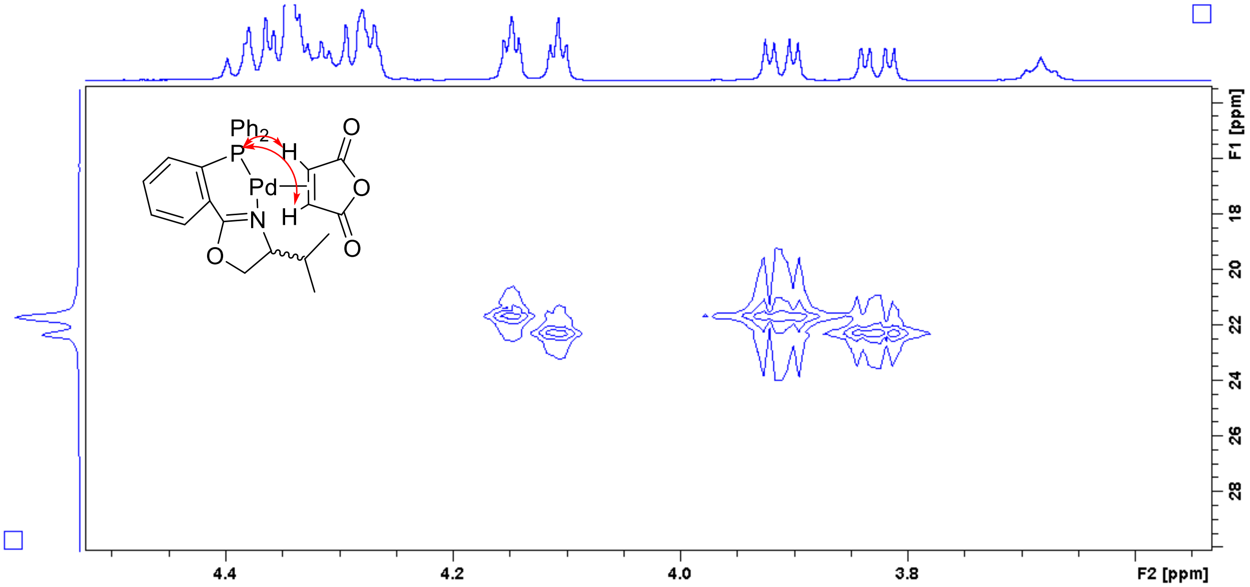

Supplementary Figure 90. ^{1}H-^{31}P HMBC NMR spectrum expansion of **6**. The correlations between P and MAH-H.

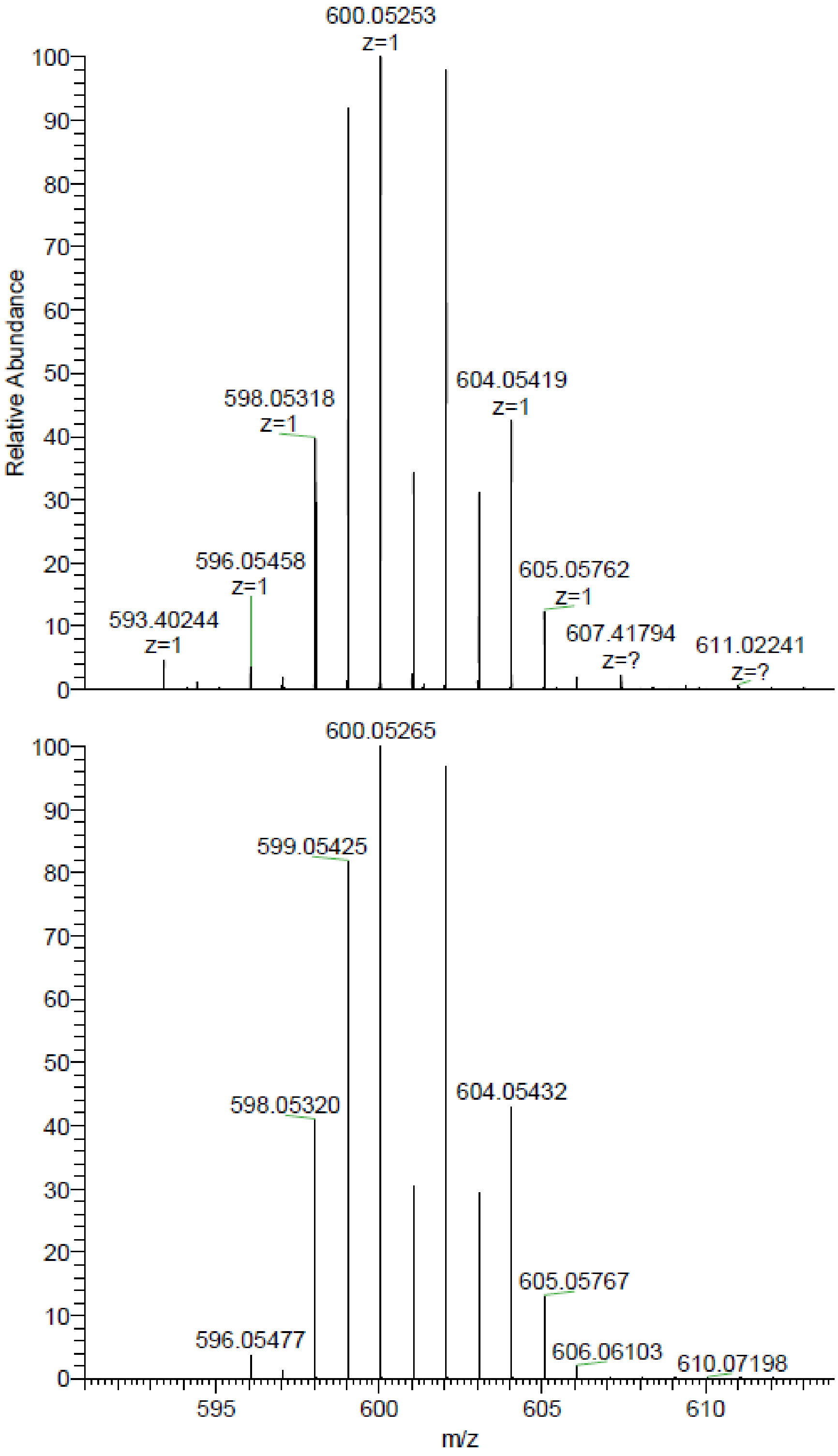

Supplementary Figure 91. Top: Experimental HRMS-ESI spectrum of [**6**·Na]$^+$. Bottom: Calculated HRMS isotope pattern for [**6**·Na]$^+$.

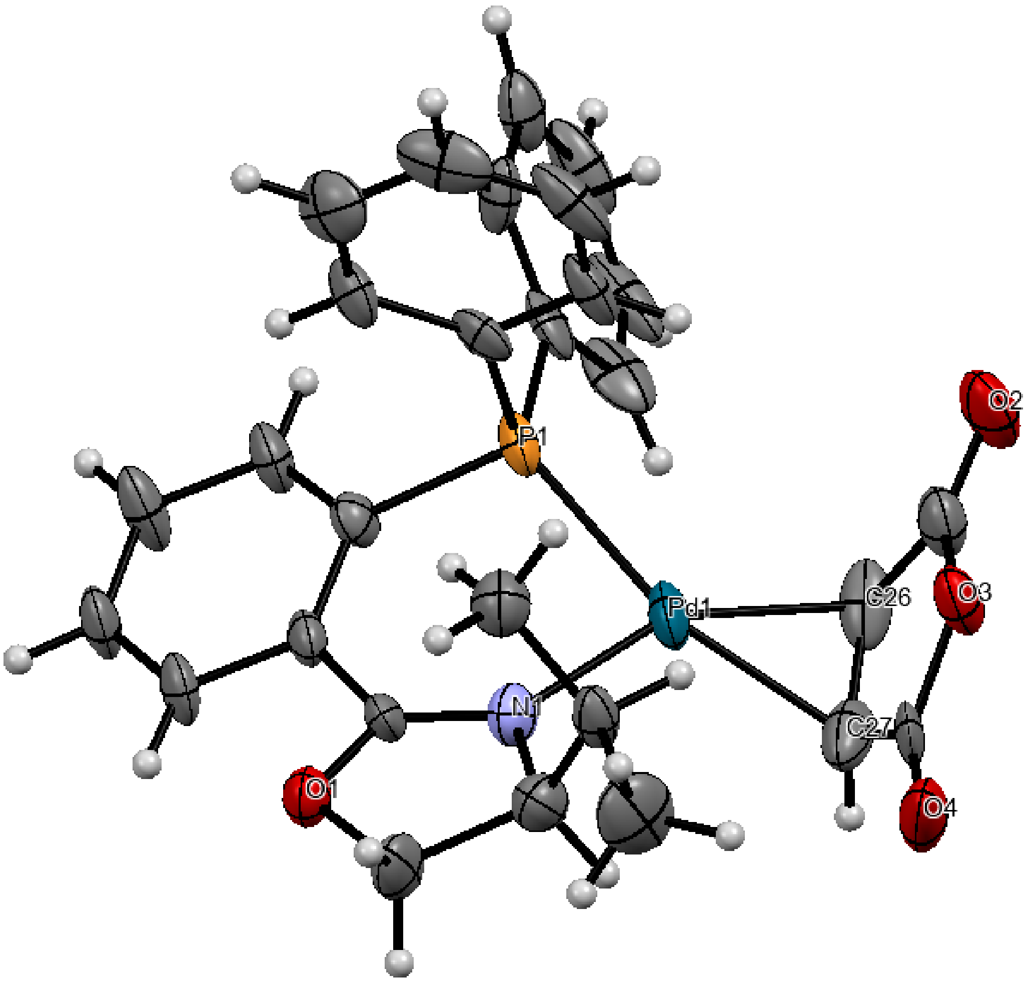

Supplementary Figure 92. Solid-state molecular structure of **6**. Thermal ellipsoids plotted at 50% probability for non-H atoms; H-atoms shown as uniformly-sized white spheres for clarity. X-ray diffraction data collected at 100.02 K. Selected bond lengths (Å) and angles (°): Pd1–P1: 2.289(3); Pd1–N1: 2.095(14); Pd1–C26: 2.072(16); Pd1–C27: 2.123(15); C26–C27: 1.41(3); P1–Pd1–N1: 87.7(3); C26–Pd1–C27: 39.4(7); C26–Pd1–P1: 120.2(5); C27–Pd1–N1: 112.8(7).

In Situ Metalation Analysis

General procedure, with **L1** as representative: the sample preparation was performed in a glovebox under a dry, oxygen-free atmosphere of nitrogen gas following the procedure shown below. A sealed NMR tube was charged with a reference solution of ^{DMP}DAB-Pd-MAH (6.3 mg, 0.0134 mmol), 1,3,5-trimethoxybenzene (2.3 mg, 0.0134 mmol) in 0.5 mL THF-d_8. Another 1-dram vial was charged with (*S,S*)-^{Ph}DACH (**L1**) (11.1 mg, 0.0161 mmol) and 0.2 mL THF-d_8 to prepare a stock solution. The initial ^{1}H NMR spectrum (500 MHz, THF-d_8) was obtained for the freshly prepared reference solution. Subsequent ^{1}H NMR and ^{31}P{^{1}H} NMR spectra were obtained immediately after mixing with the **L1** stock solution.

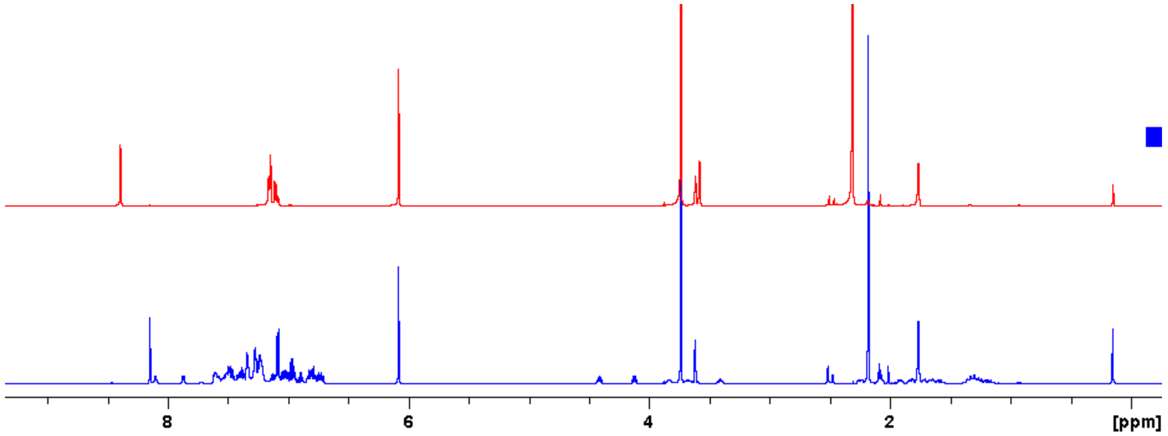

Supplementary Figure 93. ^{1}H NMR spectra (500 MHz, THF-d_8) of the metalation test for **1**. Singlets at 3.74 and 6.08 ppm are 1,3,5-trimethoxybenzene. Red: initial spectrum of ^{DMP}DAB-Pd-MAH. Blue: spectrum obtained after mixing with **L1** stock solution, indicating complete consumption of ^{DMP}DAB-Pd-MAH.

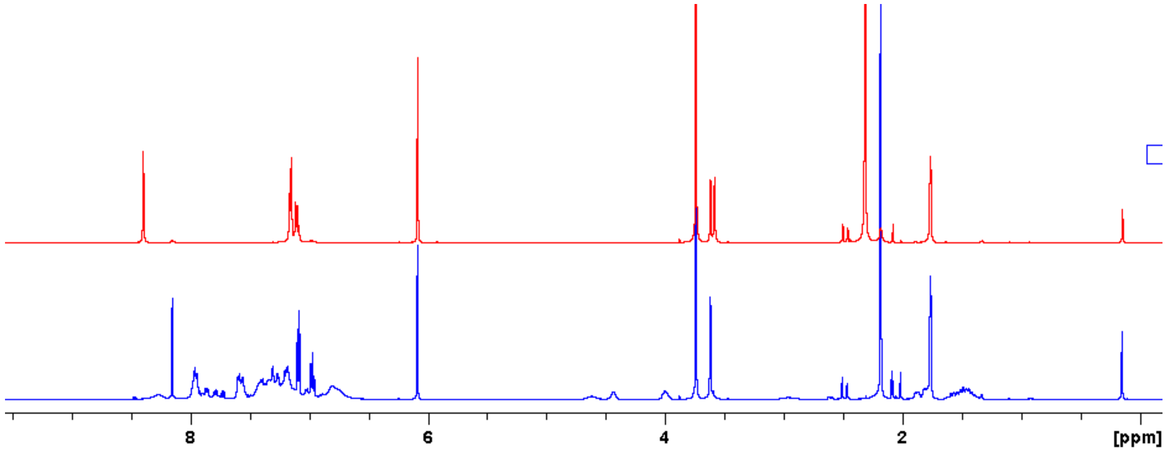

Supplementary Figure 94. ^{1}H NMR spectra (500 MHz, THF-d_8) of the metalation test for **2**. Singlets at 3.74 and 6.08 ppm are 1,3,5-trimethoxybenzene. Red: initial spectrum consisting of ^{DMP}DAB-Pd-MAH. Blue: spectrum obtained after mixing with **L2** stock solution.

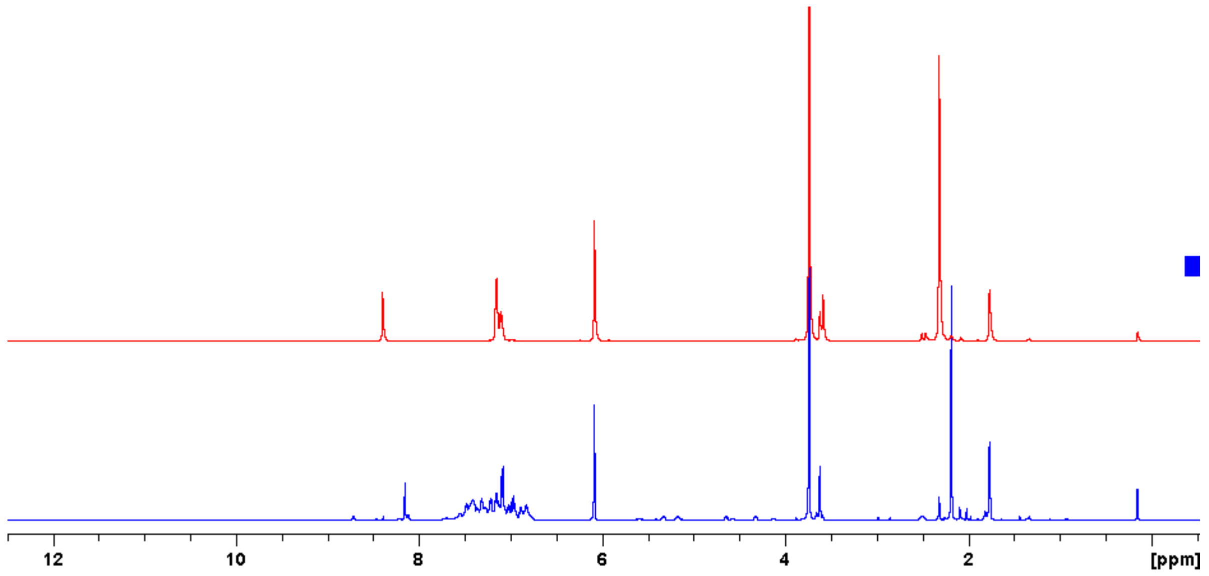

Supplementary Figure 95. ^{1}H NMR spectra (500 MHz, THF-d_8) of the metalation test for **3**. Singlets at 3.74 and 6.08 ppm are 1,3,5-trimethoxybenzene. Red: initial spectrum consisting of ^{DMP}DAB-Pd-MAH and 1,3,5-trimethoxybenzene. Blue: spectrum obtained after mixing with **L3** stock solution.

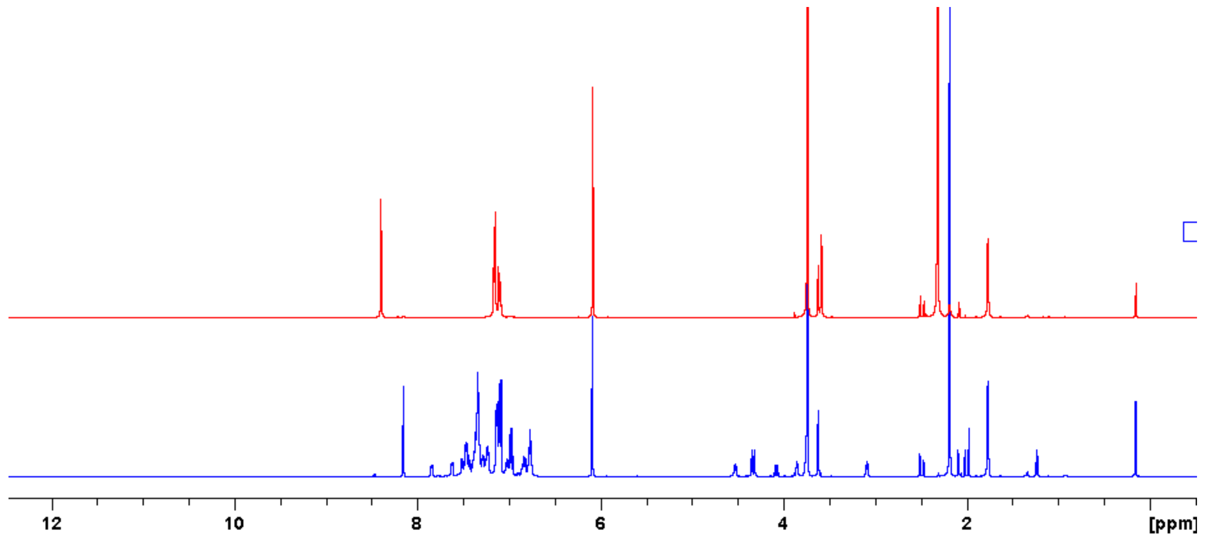

Supplementary Figure 96. ^{1}H NMR spectra (500 MHz, THF-d_8) of the metalation test for **4**. Singlets at 3.74 and 6.08 ppm are 1,3,5-trimethoxybenzene. Red: initial spectrum consisting of ^{DMP}DAB-Pd-MAH and 1,3,5-trimethoxybenzene. Blue: spectrum obtained after mixing with **L4** stock solution.

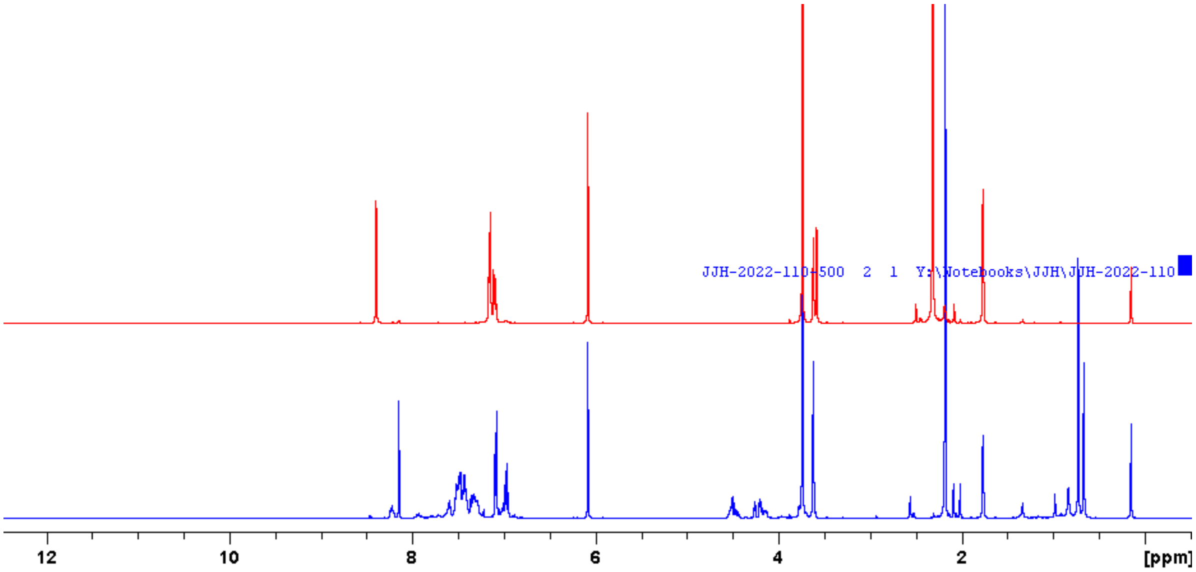

Supplementary Figure 97. ^{1}H NMR spectra (500 MHz, THF-d_8) of the metalation test for **5**. Singlets at 3.74 and 6.08 ppm are 1,3,5-trimethoxybenzene. Red: initial spectrum consisting of ^{DMP}DAB-Pd-MAH and 1,3,5-trimethoxybenzene. Blue: spectrum obtained after mixing with **L5** stock solution.

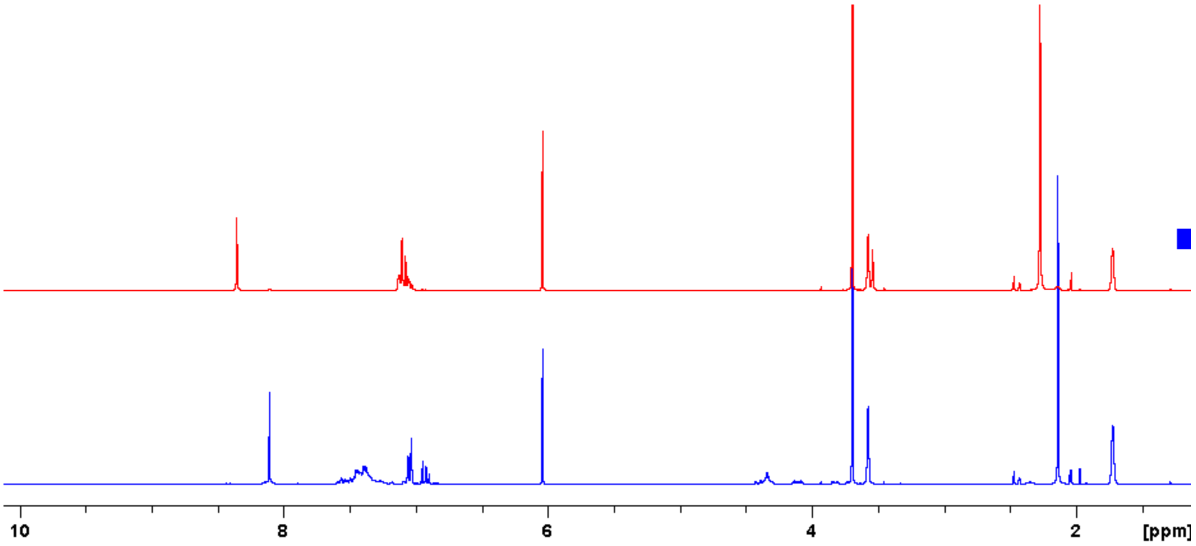

Supplementary Figure 98. ^{1}H NMR spectra (500 MHz, THF-d_8) of the metalation test for **6**. Singlets at 3.74 and 6.08 ppm are 1,3,5-trimethoxybenzene. Red: initial spectrum consisting of ^{DMP}DAB-Pd-MAH and 1,3,5-trimethoxybenzene. Blue: spectrum obtained after mixing with **L6** stock solution.

Solution Stability of Precatalysts

General procedure, with complex **1** as representative: an NMR sample was prepared from a solution of complex **1** (17.3 mg, 0.019 mmol) in 0.7 mL anhydrous THF. A sealed glass capillary containing PPh_3 in C_6D_6 was used as an internal standard. The initial ^{31}P NMR spectrum was obtained after 30 min (500 MHz). Subsequent ^{31}P NMR spectra were obtained at 2, 6, 18, 24, 30, 38, and 48 h.

Supplementary Table 1. Solution stability of six chiral Pd complexes over 48 h. The concentrations of complexes in THF over 48 h are normalized against the t = 30 min concentration to assess the stability.

			**[complex]/[complex]$_{30min}$**			
**Time/h**	**1**	**2**	**3**	**4**	**5**	**6**
0.5	1.00	1.00	1.00	1.00	1.00	1.00
2	0.99	0.98	0.98	0.98	1.01	0.99
6	0.99	0.97	1	1.01	1	0.99
18	1	1	0.99	0.99	1	0.98
24	0.99	0.98	0.99	0.99	1	0.99
30	1	1	0.99	1	0.99	0.99
38	0.99	0.99	0.99	0.98	0.99	0.98
48	0.99	0.99	0.99	0.99	0.99	1

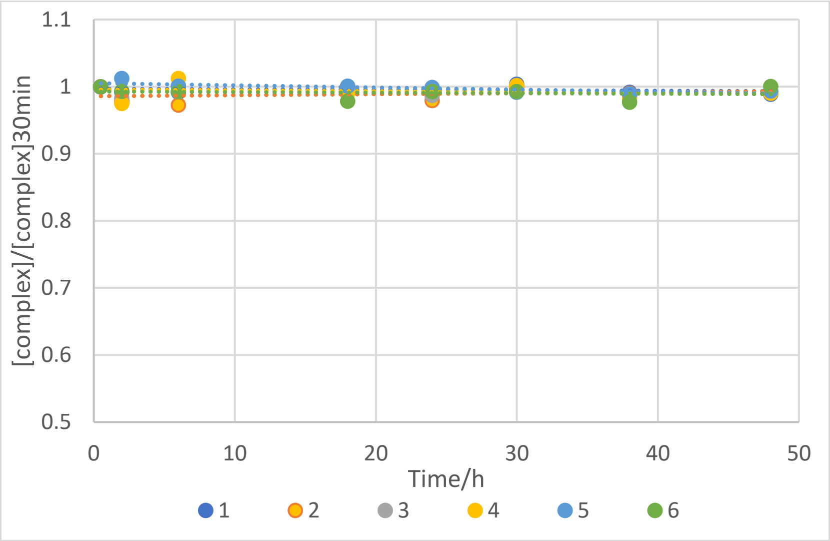

Supplementary Figure 99. Plot of normalized [L*-Pd-MAH] in THF over 48 h at rt.

(*S,S*)-^{Ph}DACH-Pd-MAH (1) (17.3 mg in 0.7 mL THF)

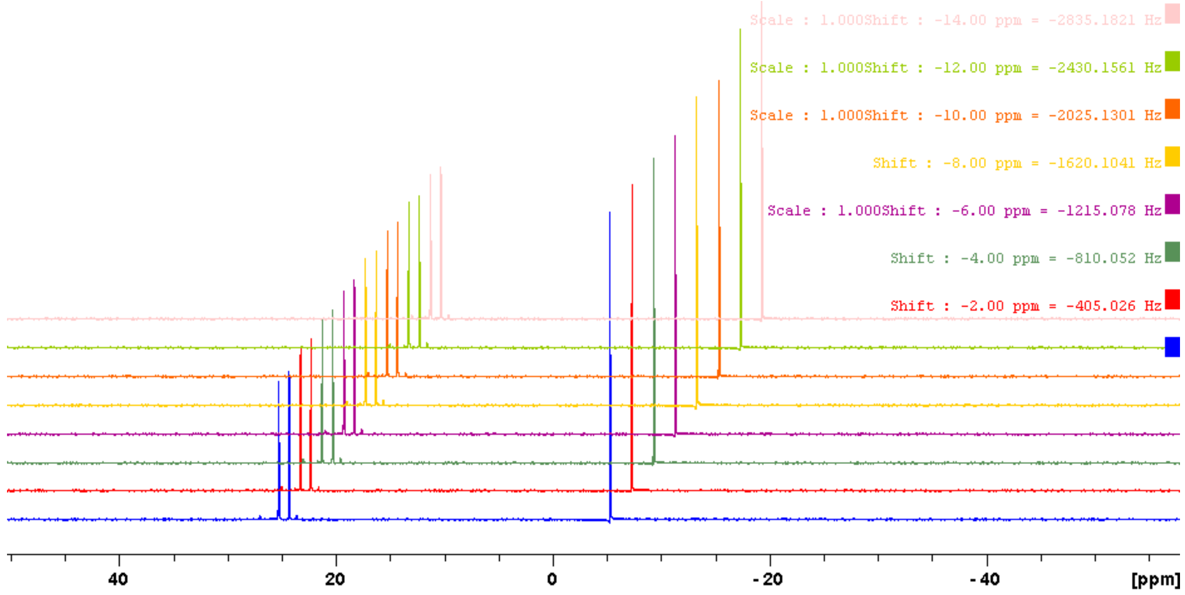

Supplementary Figure 100. $^{31}P\{^{1}H\}$ NMR spectra (200 MHz) stack plots for solution stability of **1** with time increasing from the front (30 min) to the back (48 h). Key signals are the peaks of the major conformer (24.24 ppm and 25.20 ppm) and PPh_3 internal standard (–5.36 ppm).

(*S,S*)-^{NAP}DACH -Pd-MAH (2) (20.6 mg in 0.7 mL THF)

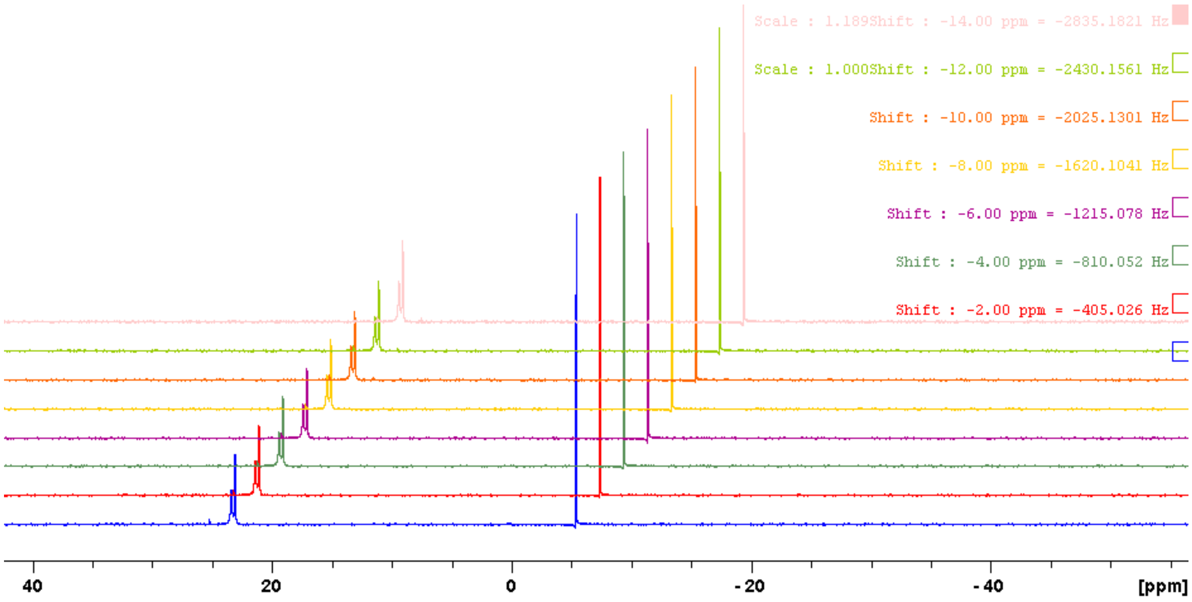

Supplementary Figure 101. $^{31}P\{^{1}H\}$ NMR spectra (200 MHz) stack plots for solution stability of **2** with time increasing from the front (30 min) to the back (48 h). Key signals are the product peaks (23.12 ppm and 23.43 ppm) and the peak of PPh_3 internal standard (–5.36 ppm).

(*S,S*)-^{Ph}STIL-Pd-MAH (3) (19.6 mg, 0.7 mL THF)

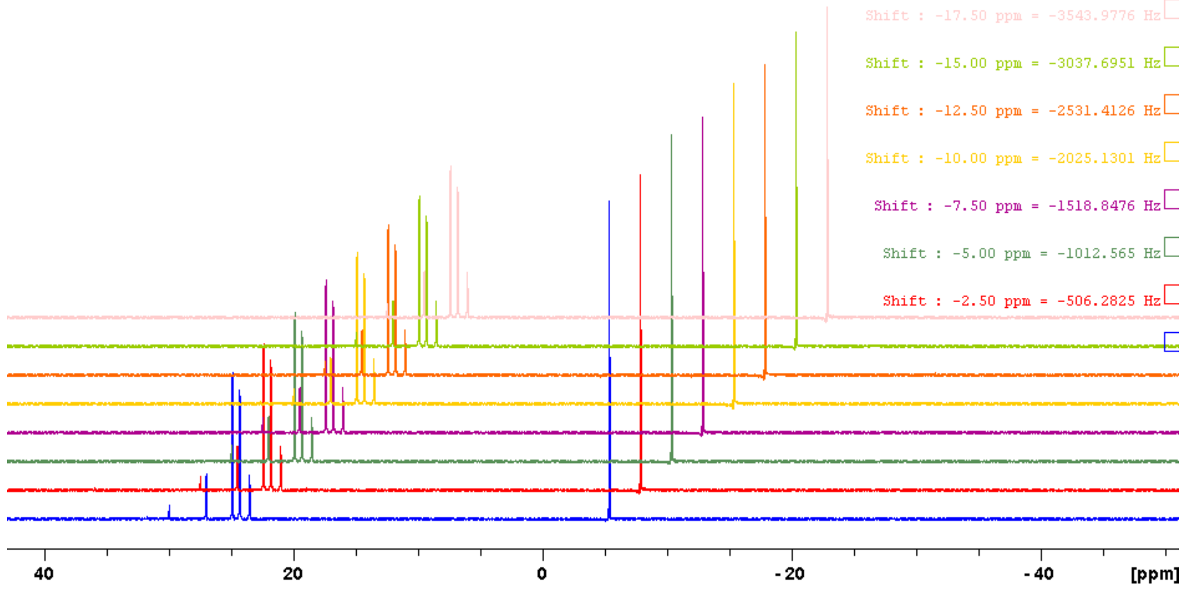

Supplementary Figure 102. $^{31}P\{^{1}H\}$ NMR spectra (200 MHz) stack plots for solution stability of **3** with time increasing from the front (30 min) to the back (48 h). Key signals are the peaks of minor conformer (23.52 ppm and 27.00 ppm), major conformer (24.29 ppm and 24.89 ppm) and PPh_3 internal standard (–5.36 ppm).

(*S,S*)-^{Ph}ANDEN-Pd-MAH (4) (12.6 mg in 0.7 mL THF)

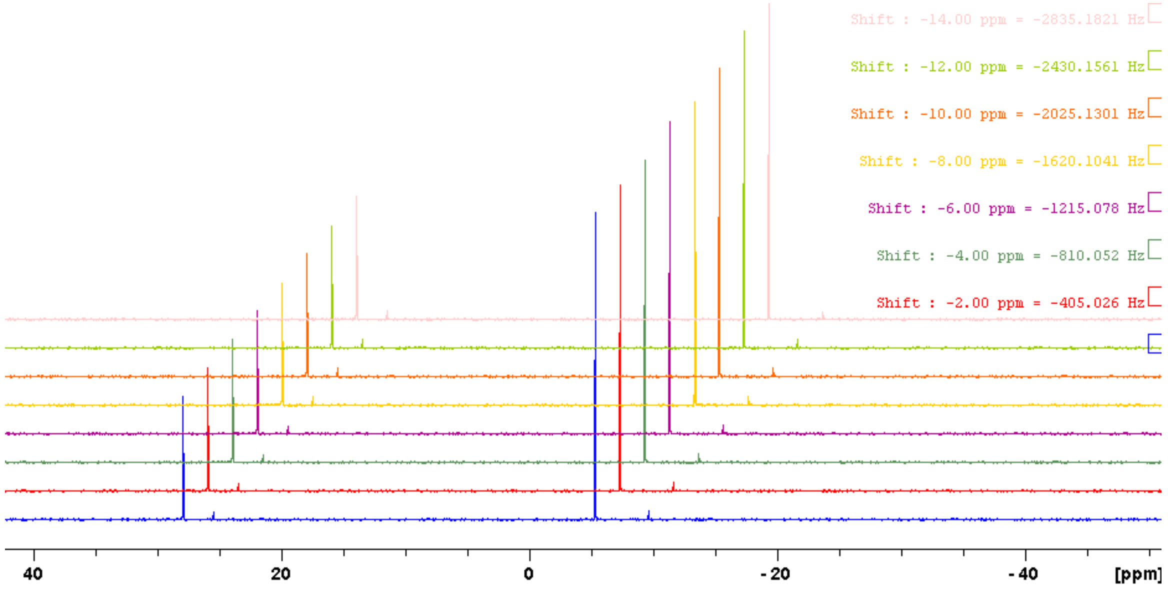

Supplementary Figure 103. $^{31}P\{^{1}H\}$ NMR spectra (200 MHz) stack plots for solution stability of **4** with time increasing from the front (30 min) to the back (48 h). Key signals are the product peak (27.93 ppm) and the peak of PPh_3 internal standard (–5.36 ppm).

(S)-^{tBu}PHOX-Pd-MAH (5) (33.2 mg, 0.6 mL THF)

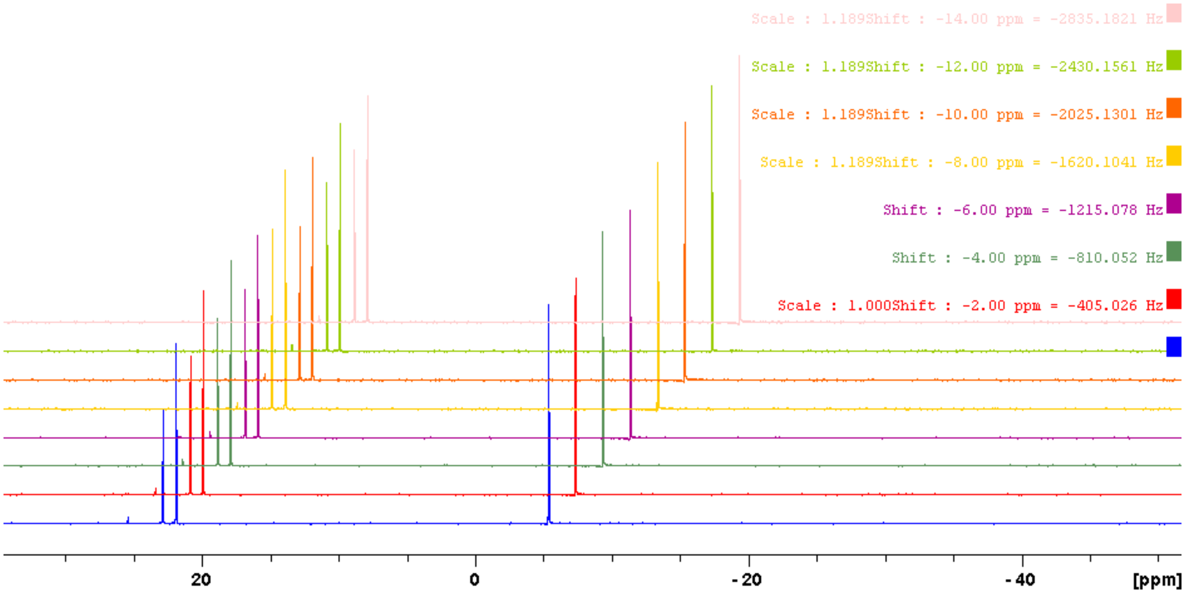

Supplementary Figure 104. $^{31}P\{^1H\}$ NMR spectra (200 MHz) stack plots for solution stability of **5** with time increasing from the front (30 min) to the back (48 h). Key signals are the peaks of minor conformer (22.83 ppm), major conformer (21.88 ppm) and PPh_3 internal standard (–5.36 ppm).

(S)-^{iPr}PHOX-Pd-MAH (6) (11.8 mg, 0.6 mL THF)

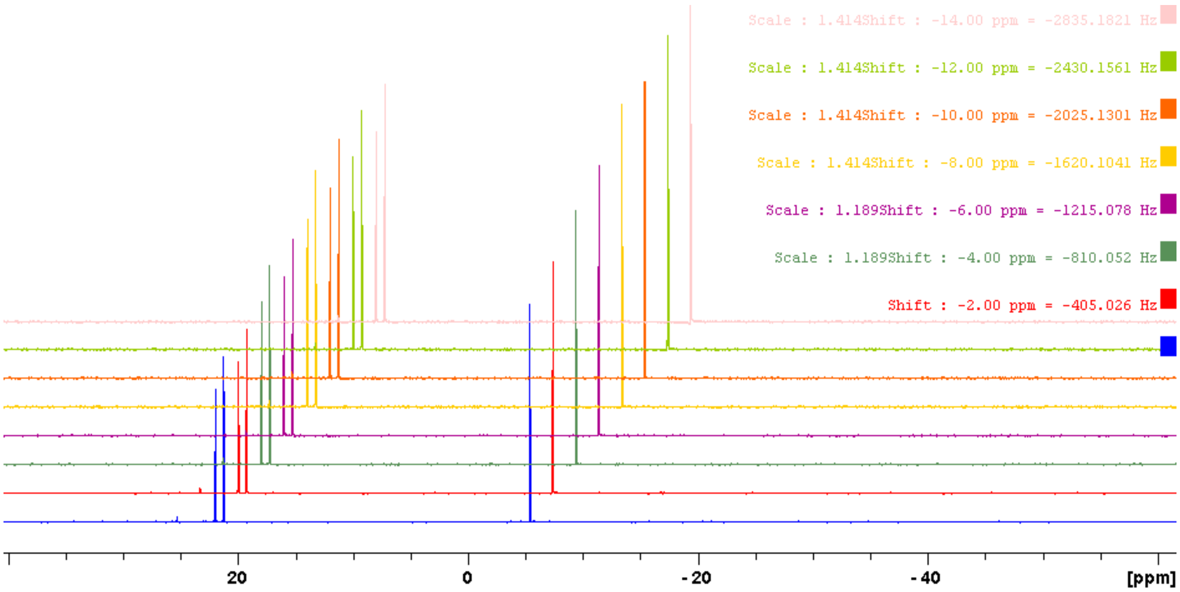

Supplementary Figure 105. $^{31}P\{^1H\}$ NMR spectra (200 MHz) stack plots for solution stability of **6** with time increasing from the front (30 min) to the back (48 h). Key signals are the peaks of minor conformer (21.99 ppm), major conformer (21.25 ppm) and PPh_3 internal standard (–5.36 ppm).

Stability of (*S,S*)-^{Ph}DACH-Pd-MAH (1) in air

An NMR sample was prepared from a solution of (*S,S*)-^{Ph}DACH-Pd-MAH (**1**) (14.9 mg, 0.017 mmol) in 0.6 mL THF. A sealed glass capillary containing PPh_3 in C_6D_6 was used as an internal standard. The initial ^{31}P NMR spectrum was obtained after 30 min. The NMR sample was exposed to the air once the initial ^{31}P NMR spectrum was obtained. Subsequent ^{31}P NMR spectra were recorded at 2, 18, 24, 30, 43, and 48 h.

Supplementary Table 2. Solution stability of **1** after exposure to air.
Normalized [**1**] and by-product formation in THF over 48 h to assess the stability.

**Time/h**	**[1]/[1]$_{30min}$**	**[(PNNP)Pd]/[1]$_{30min}$**
0.5	1.00	0.016
2	0.99	0.026
18	0.90	0.058
24	0.89	0.066
30	0.88	0.077
43	0.86	0.092
48	0.86	0.11

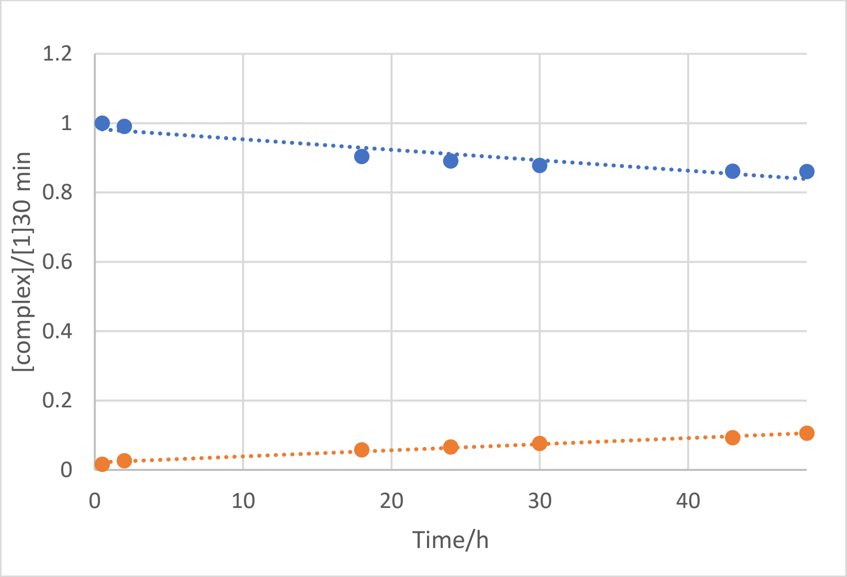

Supplementary Figure 106. Plot of normalized [**1**] (blue) and [(PNNP)Pd] (orange) after exposure to air over 48 h at rt.

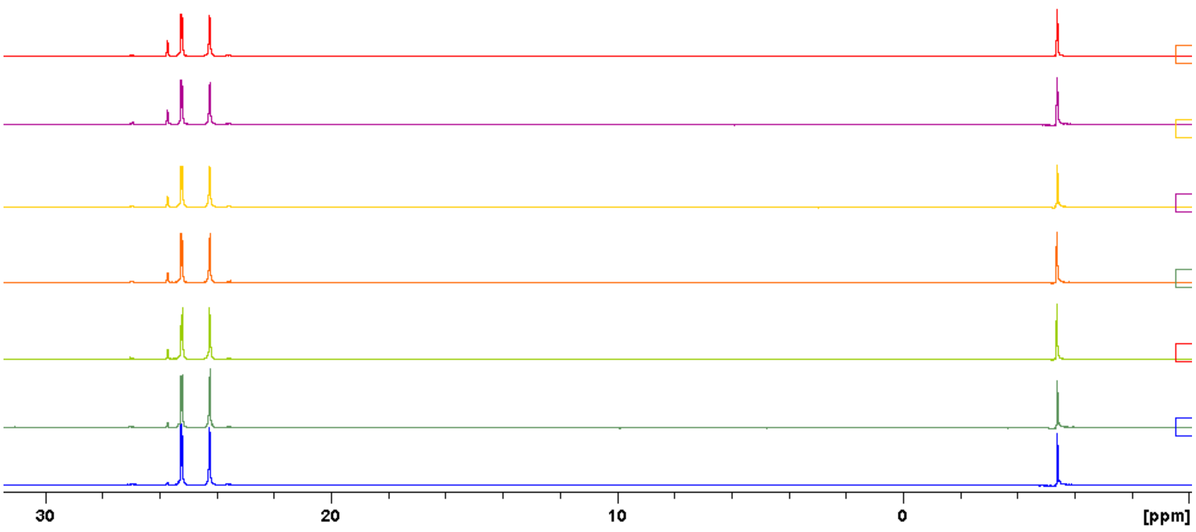

Supplementary Figure 107. $^{31}P\{^1H\}$ NMR spectra (200 MHz) stack plots for solution stability of **1** in the air with time increasing from the front (30 min) to the back (48 h). Key signals are the peaks of major conformer (24.26 ppm and 25.23 ppm), oxidized by-product (25.73 ppm) and PPh_3 internal standard (–5.36 ppm).

Stability of (*S*,*S*)-^{Ph}DACH-Pd-dba in N_2 and air

$Pd_2dba_3CHCl_3$ (12.9 mg, 0.0125 mmol), (*S*,*S*)-^{Ph}DACH (**L1**) (16.8 mg, 0.0243 mmol), and 1.2 mL THF were added to a 1-dram vial under N_2 atmosphere. The mixtures were stirred at rt for one hour. The solution was evenly split into two portions and transferred to two different sealed NMR tubes with sealed glass capillaries containing PPh_3 in C_6D_6 as internal standards. The initial ^{31}P NMR spectrum was obtained for each solution after 30 min (200 MHz). One solution was exposed to the air after the initial ^{31}P NMR spectrum was obtained. Subsequent ^{31}P NMR spectra were obtained at 2, 6, 18, 24, 30, 43, and 48 h.

Supplementary Table 3. Solution stability of phenyl Trost ligand-Pd-dba in the air and N_2. Normalized [**(*S*,*S*)-^{Ph}DACH-Pd-dba**] and [PNNP]Pd^{II} in THF over 48 h.

Time/h	(*S*,*S*)-^{Ph}DACH-Pd-dba (N_2)	[PNNP]Pd^{II} (N_2)	(*S*,*S*)-^{Ph}DACH-Pd-dba (air)	[PNNP]Pd^{II} (air)
0.5	1.00	0.00	0.47	0.71
2	1.01	0.00	0.36	0.89
6	0.96	0.00	0.25	1.15
18	0.97	0.00	0.00	0.48
24	0.96	0.00	0.00	0.31
30	0.96	0.016	0.00	0.26
43	0.95	0.038	0.00	0.15
48	0.94	0.039	0.00	0.14

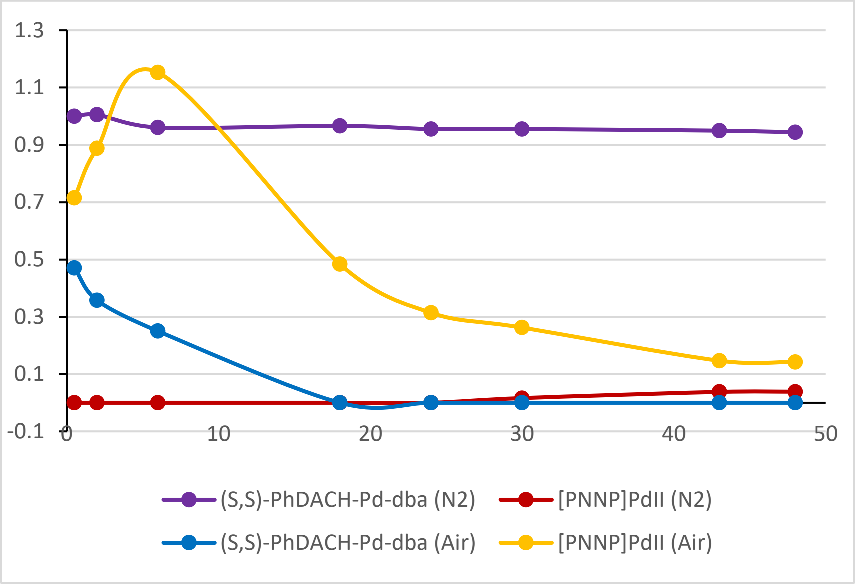

Supplementary Figure 108. Plot of normalized [**(*S,S*)-^Ph^DACH-Pd-dba**] and [[PNNP]Pd^{II}] in THF over 48 h at rt in the air and N_2. The decrease in [PNNP]Pd^{II} over time is due to precipitation of this poorly soluble complex.

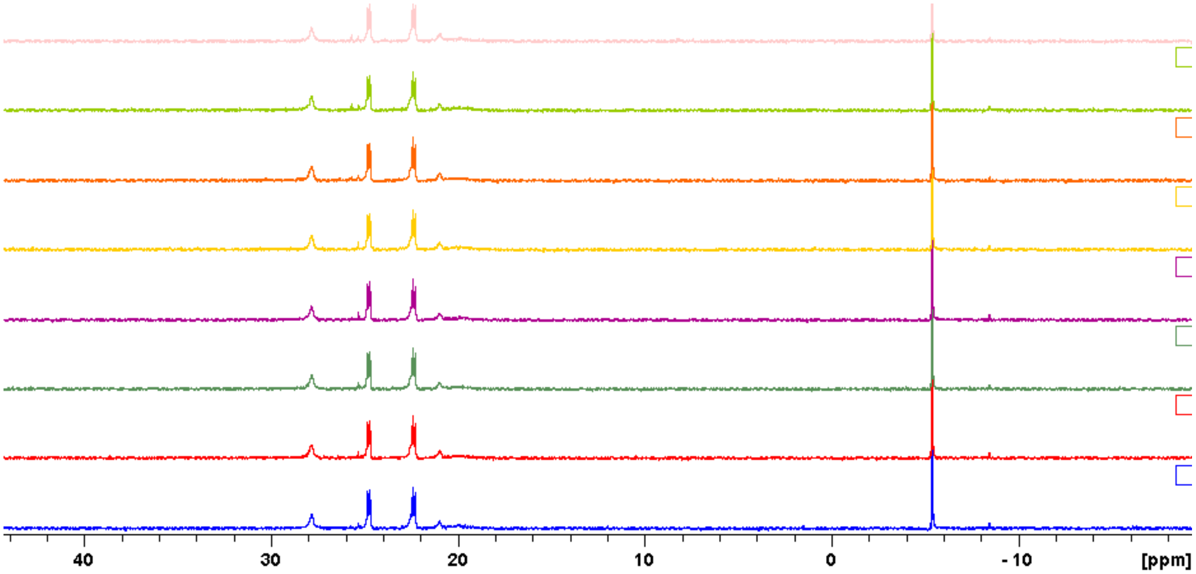

Supplementary Figure 109. $^{31}P\{^1H\}$ NMR spectra (121 MHz) stack plots for solution stability of **(*S,S*)-^Ph^DACH-Pd-dba** in N_2 with time increasing from the front (30 min) to the back (48 h). Key signals are the major product peaks (24.77 ppm and 22.35 ppm), [PNNP]Pd^{II} peak (25.69 ppm), and the peak of PPh_3 internal standard (–5.40 ppm).

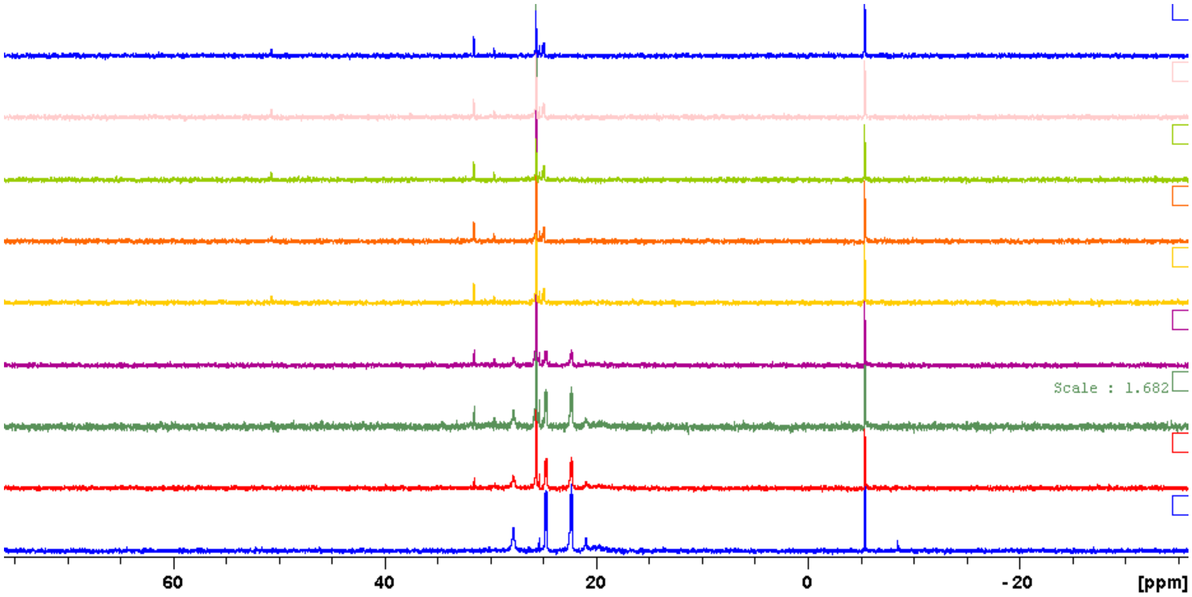

Supplementary Figure 110. $^{31}P\{^1H\}$ NMR spectra (121 MHz) stack plots for solution stability of **(*S,S*)-^{Ph}DACH-Pd-dba** in the air with time increasing from the front (30 min) to the back (48 h). Key signals are the major product peaks (24.75 ppm and 22.32 ppm), [PNNP]Pd^{II} peak (25.68 ppm), and the peak of PPh_3 internal standard (–5.36 ppm).

Investigation of Complex 1 Conformer Ratios

Spectroscopic Studies

Two species are observed in the NMR spectra of (*S,S*)-^{Ph}DACH-Pd-MAH (**1**), with a closer ratio between the two species in DCM-d_2 than in THF-d_8. To establish these two species as conformers, as proposed in the main text, a series of studies was performed.

Concentration effects: We have ruled out a monomer/dimer equilibrium as the source of these two components by observing no change to the peak area ratio of major to minor species at different initial concentrations of **1** (Supplementary Table 4).

A 1-dram vial was charged with (*S,S*)-^{Ph}DACH-Pd-MAH (**L1**) (19 mg, 0.021 mmol) and 1.2 mL DCM under N_2. Half of the solution was transferred to a sealed NMR tube, and a capillary consisting of PPh_3 in C_6D_6 was used as an internal standard. An initial ^{31}P spectrum was obtained. The other half of the solution was diluted with 0.6 mL DCM. The analysis/dilution process was repeated to give the results shown in Supplementary Table 4, revealing no change to the ratio of the two species in solution (ruling out a potential monomer/dimer equilibrium).

Supplementary Table 4. Relative peak areas for two conformers in $^{31}P\{^1H\}$ NMR spectra at different concentrations.

[1] (mM)	major conformer	minor conformer	major/minor ratio
17.5	2.007	1.541	1.303
8.8	2.000	1.627	1.229
4.4	1.933	1.419	1.362
2.2	2.075	1.434	1.447

Solvent composition effects: We have also established the interconversion of these two species by analyzing their molar ratio as a function of solvent composition in a DCM/THF mixture (Supplementary Figure 111). As the volume fraction of THF increases, the amount of the minor conformer decreases exponentially, converging to the 14:1 ratio observed in 100% THF.

An NMR sample was prepared from a solution of (*S,S*)-^{Ph}DACH-Pd-MAH (**L1**) (19.9 mg, 0.022 mmol) in 0.3 mL THF and 0.3 mL DCM under N_2 atmosphere. A sealed glass capillary containing PPh_3 in C_6D_6 was used as an internal standard. After the initial ^{31}P NMR spectrum was obtained, different amounts of THF were

subsequently added to adjust the DCM/THF ratio to 3:4, 3:7, 1:5, 1:7, and 1:9. ^{31}P NMR spectra were obtained at each ratio.

A second experiment was performed in a similar way, with different amounts of DCM added to adjust the DCM/THF ratio to 4:3, 7:3, 5:1, and 7:1. ^{31}P NMR spectra were obtained at each ratio.

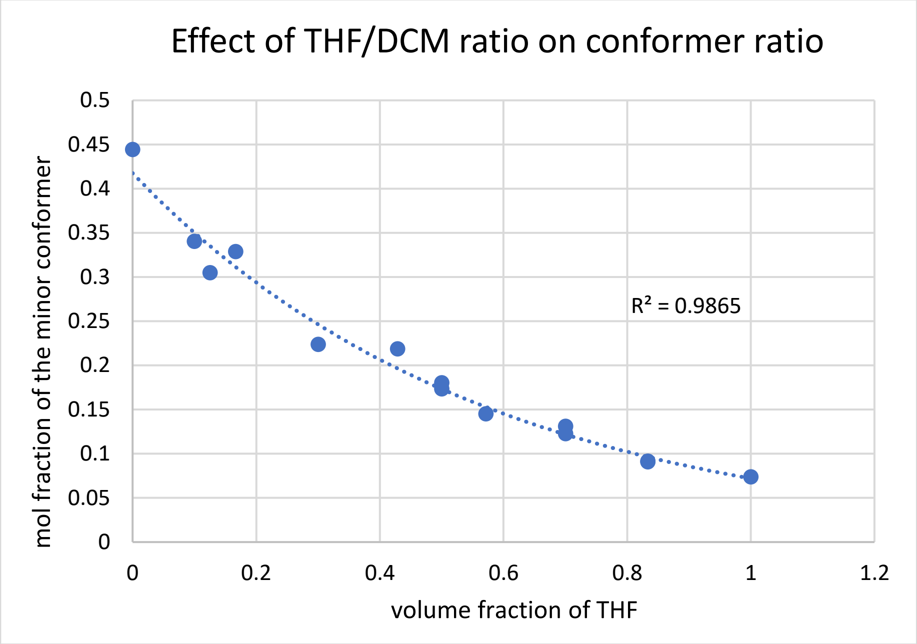

Supplementary Figure 111. Plot of mol fraction of minor conformer at different THF/DCM ratios. Mol fraction of minor conformer is determined by the peak area ratio between its ^{31}P NMR signal and the total peak area for both conformer signals.

Co-solvent identity effects: To test the hypothesis of an intramolecular hydrogen bond stabilizing the **1-*endo*** conformer, we examined the conformer ratio in 10:1 mixtures of DCM and other hydrogen-bond-accepting solvents (Supplementary Figure 112). While a 10:1 DCM/THF mixture gives a 65:35 ratio of **1-*exo*** to **1-*endo***, 10:1 DCM/DMF and 10:1 DCM/MeOH mixtures give an 80:20 **1-*exo*** to **1-*endo*** ratio. Addition of weakly hydrogen-bond-accepting solvents such as cyclopentylmethyl ether (CPME) and NEt_3 results in no change to the conformer ratio relative to that observed in DCM.

An NMR sample was prepared from a solution of (*S,S*)-^{Ph}DACH-Pd-MAH (**L1**) (10 mg, 0.011 mmol) in 0.9 mL of DCM and 0.1 mL THF. A sealed glass capillary containing PPh_3 in C_6D_6 was used as an internal standard. Similarly, other samples were prepared using 0.1 mL MeOH, DMF, CPME, and NEt_3 instead of THF, with 0.9 mL of DCM. ^{31}P NMR spectra were obtained for each solution.

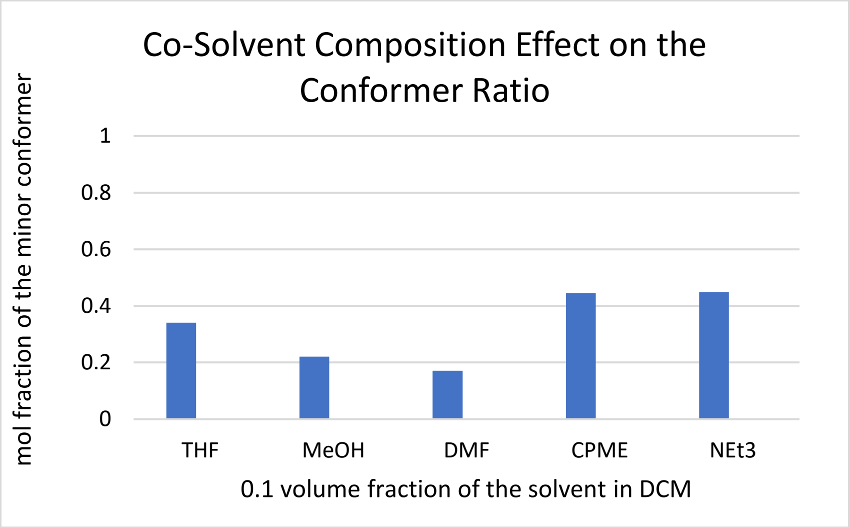

Supplementary Figure 112. Conformer ratio changes of **1** in DCM mixed with five different co-solvents.

Computational Details

The electronic energies of the two ^{Ph}DACH-Pd-MAH (**1**) conformers were calculated *in vacuo*, and in solvents THF and CH_2Cl_2. All calculations were performed using ORCA version 4.0.1.2.[5] The initial conformer geometries were edited in Avogadro version 4.1 using the molecular structure of ^{NAP}DACH-Pd-MAH (**2**) determined by X-ray crystallography as a starting point.

Geometry optimization and frequency calculations for the two ^{Ph}DACH-Pd-MAH conformers were performed at an RI-BP86 def2-SVP def2/J level with D3BJ dispersion for all atoms except for Pd, for which a def2-TZVP basis set was used. The optimized geometries were confirmed by frequency calculations that no negative frequencies were found. The single point energy (electronic energy) was calculated at an RI-B2PLYP D3 def2-TZVP def2-TZVP/C level with D3BJ dispersion for all atoms.

For the major and minor conformers in CH_2Cl_2 and the minor conformer in THF, an implicit solvation model (CPCM)[6] was used in both geometry optimization and single point energy calculations. For the major conformer in THF, both the implicit (CPCM) solvation model and an explicit THF molecule were included to account for the intramolecular hydrogen bonding between the solvent and the conformer.

Coordinate files (xyz format) for the six calculated structures are included as additional supplementary files.

Catalytic Asymmetric Allylic Alkylation Reactions

DYKAT malonation of cyclohex-2-en-1-yl methyl carbonate (7)

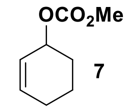

7

Cyclohex-2-en-1-yl methyl carbonate (7). Prepared according to an adapted literature procedure.[7] To a solution of cyclohex-2-ol (501 mg, 0.5 mL, 5.1 mmol, 1 equiv.) and pyridine (2.37 g, 2.42 mL, 30 mmol, 6 equiv.) in CH_2Cl_2 (15 mL) at 0 °C was added methyl chloroformate (1.21 g, 991 µL, 12.8 mmol, 2.5 equiv.). The reaction was then stirred at rt for 18 h. The reaction was quenched with 1 M HCl (15 mL) and the layers separated. The organic phase was then washed with saturated aqueous $NaHCO_3$, and brine, dried over $MgSO_4$, filtered and the solvent removed under reduced pressure. The residue was purified by flash column chromatography over silica gel eluting with hexane and EtOAc to yield the title compound (755 mg, 4.8 mmol, 95%) as a colourless oil. Spectroscopic data was in accordance with literature values.[7]

^{1}H NMR (400 MHz, CDCl$_3$) δ 6.00-5.95 (m, 1H), 5.77 (m, 1H), 5.12 (m, 1H), 3.77 (s, 3H), 2.15-1.58 (m, 6H).

^{13}C NMR (100 MHz, CDCl$_3$) δ 155.7, 133.5, 125.1, 77.5, 77.2, 76.8, 72.1, 54.7, 28.4, 25.0, 18.7.

General procedure for the malonation of cyclohex-2-en-1-yl methyl carbonate. In a microwave vial, NaH

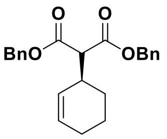

(60% in mineral oil, 25 mg, 0.62 mmol, 2.5 equiv.) was triturated with hexane before being suspended in CH_2Cl_2 (2 mL). Dibenzyl malonate[8] (142 mg, 125 µL, 0.5 mmol, 2 equiv.) was then added and the mixture was stirred at rt for 5 min. Tetrahexylammonium bromide (THAB, 325 mg, 0.75 mmol, 3 equiv.) in CH_2Cl_2 (0.5 mL) was then added and a suspension formed. In a separate microwave vial, either Pd source and **L1** (in respective mol%) or precatalyst **1** (11 mg, 0.0125 mmol, 5 mol%) was/were dissolved in 2.5 mL CH_2Cl_2 and stirred for 30 min at rt. Then, cyclohex-2-en-1-yl methyl carbonate (39 mg, 0.25 mmol, 1 equiv.) was added. The suspension of dibenzyl malonate was then added by syringe dropwise at rt and the reaction mixture stirred for 24 h. The reaction was quenched with H_2O (10 mL) and the layers separated. The aqueous phase was extracted with CH_2Cl_2 (3 x 10 mL), and the combined organics were dried over anhydrous Mg_2SO_4, filtered and the solvent removed under reduced pressure. The residue was purified by flash column chromatography over silica gel eluting with hexane and EtOAc (100:0 to 95:5) to yield the title compound. Spectroscopic data was in accordance with the literature values.[9]

^{1}H NMR (400 MHz, CDCl$_3$) δ 7.37-7.28 (m, 10H), 5.75 (m, 1H), 5.55 (m, 1H), 5.20-5.12 (m, 4H), 3.40 (d, *J* = 9.1 Hz, 1H), 2.96 (m, 1H), 2.01-1.93 (m, 2H), 1.82-1.65 (m, 2H), 1.61-1.49 (m, 1H), 1.39 (m, 1H).

^{13}C NMR (101 MHz, CDCl$_3$) δ 168.4, 168.3, 135.6, 135.5, 129.8, 128.7, 128.4, 128.4, 128.3, 128.3, 127.5, 77.5, 77.2, 76.8, 67.1, 57.2, 35.6, 26.7, 25.1, 21.1.

HPLC: Daicel CHIRALPAK IA, *n*-hexane/*i*-PrOH: 90/10, flow rate = 1 mL/min, T = 35 °C, λ = 210 nm, t_R = 17.47 min (minor), t_R = 18.94 min (major).

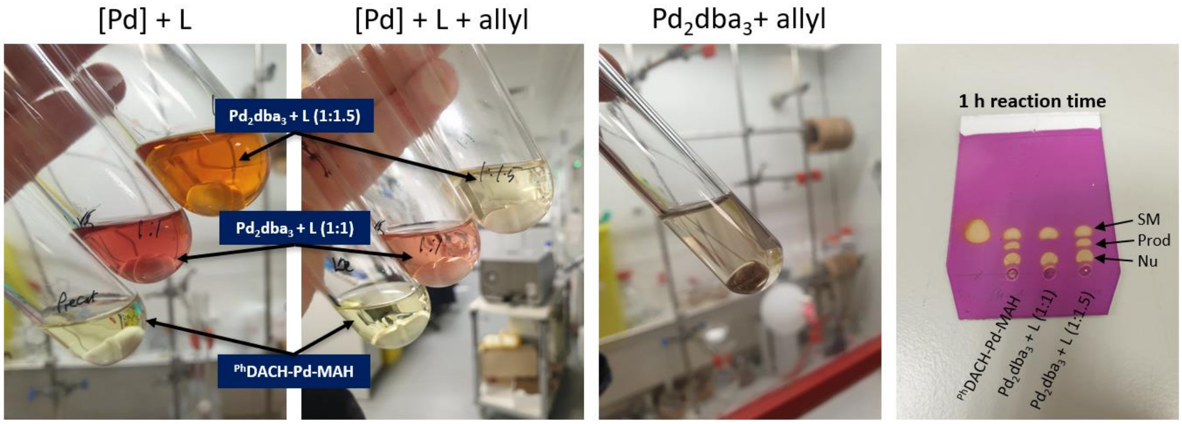

Supplementary Figure 113. (**A**) Indicated catalyst in solution after 30 min. (**B**) Indicated catalyst plus allyl carbonate **7**.
(**C**) Pd_2dba_3 with no additional ligand plus allyl carbonate **7**. (**D**) TLC after 30 min reaction time.

Supplementary Table 5. Catalyst system comparison for the synthesis of **8**

Malonation of cyclic allyl carbonate

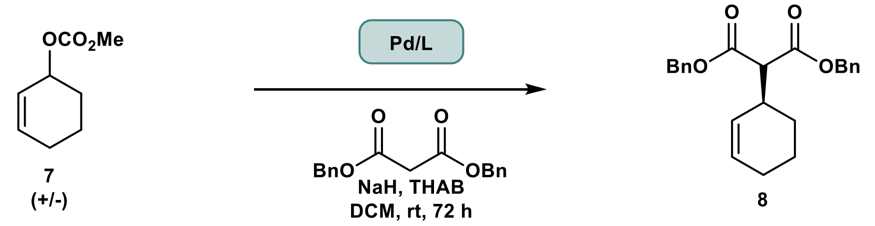

Entry	Pd source	Pd (mol%)	L (mol%)	Pd:L	Yield %	ee %
1	[PdCl(allyl)]$_2$	5	7.5	1:1.5	86	95
2	Pd_2dba_3	5	7.5	1:1.5	96	95
3	^{DMP}DAB-Pd-MAH	5	7.5	1:1.5	86	95
4	Pd_2dba_3	5	5	1:1	0	-
5	Pd_2dba_3	5	-	1:0	0	-
6	^{Ph}DACH-Pd-MAH (1)	5	-	1:1	86	96

L = (*S*,*S*)-^{Ph}DACH (L1). Reactions conducted on a 0.25 mmol scale, with 2 equivalents of dibenzyl malonate, 2.5 equivalents of NaH, and 3 equivalents of THAB in CH_2Cl_2 [0.05 M].

Desymmetrisation of *meso bis*(acetate) 9.

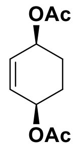

9

***cis*-Cyclohex-2-ene-1,4-diyl diacetate (9)** was prepared following an adapted literature procedure.[10] To a vial charged with $Pd(OAc)_2$ (70 mg, 0.3 mmol), LiCl (52 mg, 0.1 mmol), LiOAc 7.82 mmol), was added acetic acid (5 mL). A solution of cyclohexa-1,3-diene (500 mg, 6.2 mmol) in hexane (10 mL) was then added and the mixture vigorously stirred at rt for 24 h. The mixture was filtered through a pad of Celite©, and a saturated aqueous solution of brine (30 mL) was added to the filtrate. The mixture was extracted with hexane (3 x 30 mL), and the combined organics dried over anhydrous $MgSO_4$, filtered, and the solvent removed under reduced pressure. The residue was purified by flash column chromatography over silica gel eluting with hexane and EtOAc (100:0 to 90:10) to yield the title compound (921 mg, 4.65 mmol, 75%) as a colourless oil. Spectroscopic data was in accordance with the literature values.[10]

^{1}H NMR (400 MHz, $CDCl_3$) δ 5.90 (2 H, s), 5.22 (2 H, brs), 2.06 (6 H, s), 1.95-1.81 (4 H, m).

^{13}C NMR (100 MHz, $CDCl_3$) δ 170.7, 130.4, 67.4, 25.0, 21.4.

General procedure for synthesis of dibenzyl 2-((1*S*,4*R*)-4-acetoxycyclohex-2-en-1-yl)malonate (10) (Supplementary Table 6).[11]

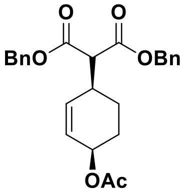

A solution of Pd source (indicated mol%) and **L1** (if required, in indicated mol%) in THF (0.5 ml) was stirred at rt for 30 min. To this solution was added *cis*-2-cyclohexenyl-1,4-diacetate (**9**) (0.3 mmol, 60 mg, 1 equiv.). In a separate vessel, NaH (60% in mineral oil and used directly, 11 mg, 0.45 mmol, 1.5 equiv.) was suspended in THF (1 mL) followed by addition of dibenzyl malonate (105 mg, 0.36 mmol, 1.2 equiv.). The mixture was stirred at rt for 5 min. The solution of catalyst was cooled to 0 °C before addition of the malonate solution *via* syringe. The reaction mixture was stirred at 0 °C for 24 h before diluting with water (10 mL) and extracting with CH_2Cl_2. The combined organics were dried over anhydrous Mg_2SO_4, filtered and the solvent removed under reduced preasure. The residue was purified by flash column chromatography over silica gel eluting with hexane and EtOAc (100:0 to 95:5) to yield the title compound. Spectroscopic data was in accordance with the literature values.[11]

^{1}H NMR (400 MHz, $CDCl_3$) δ 7.36-7.27 (m, 10H), 5.86-5.77 (m, 2H), 5.20-5.12 (m, 5H), 3.43 (d, J = 9.1 Hz, 2H), 2.90 (m, 2H), 2.02 (s, 3H), 1.88-1.66 (m, 3H), 1.58-1.48 (m, 3H).

^{13}C NMR (101 MHz, $CDCl_3$) δ 170.7, 168.0, 167.9, 135.4, 135.4, 133.5, 128.7, 128.5, 128.5, 128.3, 127.1, 67.4, 66.4, 56.4, 35.6, 27.1, 22.4, 21.4.

HPLC: Daicel CHIRALPAK IC, *n*-hexane/*i*-PrOH: 90/10, flow rate = 1 mL/min, T = 35 °C, λ = 210 nm, t_R = 25.93 min (major), t_R = 27.901 min (minor).

Supplementary Table 6. Catalyst system comparison for the synthesis of **10**

Desymetrisation of meso bis-acetate

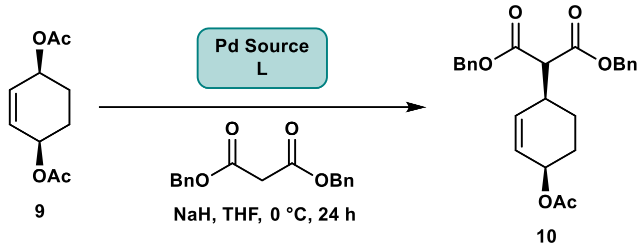

Entry	Pd source	Pd (mol %)	L (mol %)	Pd:L	Yield %	ee %
1	$[PdCl(allyl)]_2$	5	7.5	1:1.5	56	95
2	Pd_2dba_3	5	7.5	1:1.5	35	96
3	^{DMP}DAB-Pd-MAH	5	7.5	1:1.5	65	98
4	$[PdCl(allyl)]_2$	2	2	1:1	46	>99
5	Pd_2dba_3	2	3	1:1.5	24	98
6	Pd_2dba_3	2	2	1:1	15	92
7	^{DMP}DAB-Pd-MAH	2	2	1:1	30	92
8	^{Ph}DACH-Pd-MAH (1)	2	-	1:1	68	98

L = (*S*,*S*)-^{Ph}DACH (L1). Reactions run on a 0.3 mmol scale with 1.2 equivalents of malonate, 1.5 equivalents of NaH in THF [0.1 M].

Allylic amination of butadiene monoxide (12) with phthalimide (11)

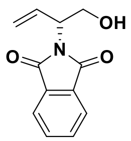

General procedure for the synthesis of 2-(1-hydroxybut-3-en-2-yl)isoindoline-1,3-dione (13) (Supplementary Table 7): The desired Pd source (indicated mol%), **L2** (if required, indicated mol%), and Na_2CO_3 (5.3 mg, 0.05 mmol, 5 mol%) were dissolved/suspended in CH_2Cl_2 (8 mL) and the mixture stirred at rt for 30 min. Then, phthalimide (**11**) (154 mg, 1.05 mmol, 1.05 equiv.) and 3,4-epoxy-1-butene (**12**) (70 mg, 81 µL, 1 mmol, 1 equiv.) were added. The reaction mixture was stirred at rt overnight. The solvent was then removed under reduced pressure, and the residue purified by flash column chromatography over silica gel eluting with hexane and EtOAc to yield the title compound as a colourless solid. Spectroscopic data was in accordance with the literature values.[12,13]

^{1}H NMR (400 MHz, $CDCl_3$) δ 7.88-7.82 (m, 2H), 7.76-7.70 (m, 2H), 6.21-6.11 (m, 1H), 5.33-5.24 (m, 2H), 4.97-4.89 (m, 1H), 4.14 (dt, *J* = 11.6, 8.1 Hz, 1H), 3.97 (dt, *J* = 11.7, 3.9 Hz, 1H), 2.65 (dd, *J* = 8.5, 3.8 Hz, 1H).

^{13}C NMR (101 MHz, $CDCl_3$) δ 168.6, 134.2, 132.0, 131.8, 123.5, 118.9, 63.0, 56.0.

HPLC: Daicel CHIRALPAK IB, *n*-hexane/*i*-PrOH: 90/10, flow rate = 1 mL/min, T = 35 °C, λ = 230 nm, t_R = 12.10 min (minor), t_R = 13.08 min (major).

Supplementary Table 7. Catalyst System Comparison for Synthesis of **13**

Allylic amination

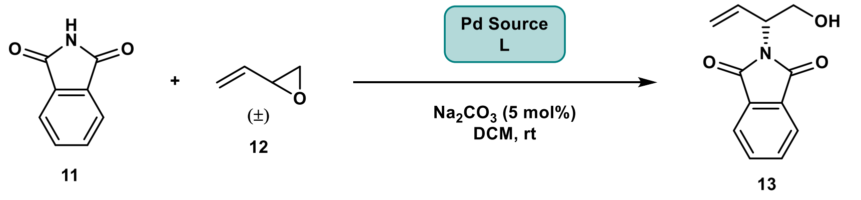

Entry	Pd source	Pd (mol %)	L (mol %)	Pd:L	Yield %	ee %
1	$[Pd(allyl)Cl]_2$	0.8	1.2	1:1.5	99	72 (96)[1]
2	Pd_2dba_3	0.8	1.2	1:1.5	82	64
3	^{DMP}DAB-Pd-MAH	0.8	1.2	1:1.5	84	82
4	^{NAP}DACH-Pd-MAH (2)	0.8	-	(1:1)	99	81

L = (*S*,*S*)-^{NAP}DACH. Reactions run on a 1 mmol scale wrt the epoxide, with 1.05 equivalents of phthalimide, and 0.05 equivalents Na_2CO_3 in CH_2Cl_2 [0.125 M].

Decarboxylative allylation of enol carboxylate 14

2-Phenylcyclohexan-1-one: Synthesised following an adapted literature procedure.[14] Cyclohexanone

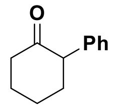

(1.38 ml, 13.2 mmol, 1.1 equiv.) and bromobenzene (1.35 ml, 12 mmol, 1 equiv.) were added to a solution of KO^{t}Bu (2 g, 18 mmol, 1.5 equiv.) in THF (10 mL). In a separate vessel, $PdOAc_2$ (54 mg, 0.24 mmol, 2 mol%) was dissolved in THF (1 mM) and P^{t}Bu$_3$ (72 mg, 0.36 mmol, 2.5 mol%) was added. The resulting red solution was added to the starting materials and the reaction heated at 60 °C for 3 h. The reaction mixture was cooled to rt and filtered through a short pad of Celite© washing with Et_2O (50 mL). The organics were then washed with water, saturated $NaHCO_3$, and brine, dried over anhydrous $MgSO_4$, filtered and the solvent removed under reduced pressure. The crude material was purified by flash column chromatography over silica gel eluting with hexane and Et_2O (95:5) to recover the title compound (1.2 g, 0.69 mmol, 58%) as a white solid. The product can be further purified if desired by dissolving in the minimum volume of CH_2Cl_2 and adding dropwise to hexane at 0 °C. The precipitate is then filtered to obtain the title compound as a white solid. Spectroscopic data was in accordance with the literature values.[14]

^{1}H NMR (400 MHz, $CDCl_3$) δ 7.34 (m, 1H), 7.28-7.23 (m, 1H), 7.16-7.12 (m, 2H), 3.61 (dd, *J* = 12.1, 5.4 Hz, 1H), 2.58-2.41 (m, 2H), 2.32-2.23 (m, 1H), 2.20-1.97 (m, 3H), 1.90-1.76 (m, 2H).

^{13}C NMR (101 MHz, $CDCl_3$) δ 210.4, 138.9, 128.7, 128.5, 127.0, 57.5, 42.3, 35.2, 28.0, 25.5.

Allyl (3,4,5,6-tetrahydro-[1,1'-biphenyl]-2-yl) carbonate (14): To a solution of 2-phenylcyclohexan-1-one

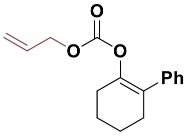

(560 mg, 3.2 mmol, 1 equiv.) in THF (1.6 mL) was added NaH (141 mg, 3.5 mmol, 1.1 equiv., triturated with hexane prior to use to remove the mineral oil), and TMEDA (0.48 mL). The mixture was heated at reflux for 1 h before being cooled to 0 °C. Allyl chloroformate (0.35 mL, 3.2 mmol, 1.1 equiv.) in THF (1.6 mL) was cooled to 0 °C and added to the 2-phenylcyclohexan-1-one solution *via* cannula at 0 °C. The reaction was stirred at the same temperature for 15 min before being quenched with a saturated aqueous solution of NH_4Cl (20 mL) and extracted with Et_2O (3 x 20 mL). The organics were dried over anhydrous $MgSO_4$, filtered, and the solvent removed under reduced pressure. The residue was purified by flash column chromatography over silica gel eluting with hexane and Et_2O (95:5) to yield the title compound (405 mg, 1.6 mmol, 49%) as a colourless oil. [**Note:** the product co-eluted on the column with diallylcarbonate, an impurity that was removed under reduced pressure at 50 °C. Spectroscopic data was in accordance with the literature values.[15]

^{1}H NMR (400 MHz, $CDCl_3$) δ 7.33-7.28 (m, 2H), 7.26-7.19 (m, 3H), 5.79 (ddt, *J* = 17.2, 10.5, 5.6 Hz, 1H), 5.22-5.15 (m, 2H), 4.51 (dt, *J* = 5.6, 1.4 Hz, 2H), 2.37 (m, 4H), 1.88-1.73 (m, 4H).

^{13}**C NMR (101 MHz, CDCl$_3$) δ** 153.0, 143.5, 138.9, 131.5, 128.3, 127.8, 127.0, 126.0, 118.7, 68.6, 30.3, 27.2, 23.0, 22.7.

General procedure for synthesis of (*R*)-2-allyl-2-phenylcyclohexan-1-one (15) (Supplementary Table 8):[16] The Pd source (indicated mol%) and **L4** (if required, indicated mol%) were dissolved in toluene (1 mL) and stirred at rt for 30 min before being cooled to –78 °C. Separately, a solution of **14** (52 mg, 0.2 mmol) in toluene (1 mL) was cooled to –78 °C. This solution was then added to the catalyst mixture. The reaction mixture was stirred at –78 °C for 10 min before being removed from the cooling bath and allowed to warm to rt over ~5 min. Once the starting material was consumed (reaction monitored by TLC), the reaction was diluted with Et_2O (20 mL) and washed with a saturated aqueous solution of brine. The organic layer was dried over anhydrous $MgSO_4$, filtered, and the solvent removed under reduced pressure. The residue was purified by flash column chromatography over silica gel eluting with hexane and Et_2O (95:5) to yield the title compound as a colourless oil. Spectroscopic data was in accordance with the literature values.[16]

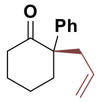

^{1}**H NMR (400 MHz, CDCl$_3$) δ** 7.34 (m, 2H), 7.26-7.22 (m, 1H), 7.15 (m, 2H), 5.45 (dddd, *J* = 17.0, 10.2, 7.9, 6.8 Hz, 1H), 4.89 (m, 2H), 2.71-2.63 (m, 1H), 2.54-2.39 (m, 2H), 2.39-2.24 (m, 2H), 1.98-1.89 (m, 1H), 1.82-1.61 (m, 4H).

^{13}**C NMR (101 MHz, CDCl$_3$) δ** 213.2, 140.7, 134.5, 128.9, 127.0, 126.9, 117.8, 76.8, 76.8, 57.0, 45.1, 40.3, 34.8, 28.4, 21.7.

HPLC: Daicel CHIRALPAK IB, *n*-hexane/*i*-PrOH: 99.9/0.1, flow rate = 0.5 mL/min, T = 35 °C, λ = 230 nm, t_R = 12.26 min (minor), t_R = 16.76 min (major).

Supplementary Table 8. Catalyst system comparison for the synthesis of **15**

Pd-DAAA

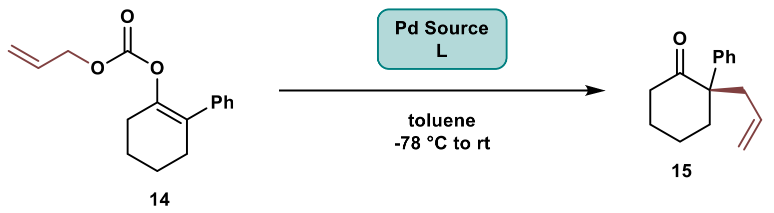

Entry	Pd source	Pd (mol %)	L (mol %)	Pd:L	t (h)	Yield %	ee %
1	$[Pd(allyl)Cl]_2$	5	7.5	1:1.5	24[a]	21	81
2	Pd_2dba_3	5	7.5	1:1.5	0.5	86	83
3	^{DMP}DAB-Pd-MAH	5	7.5	1:1.5	0.5	84	81
4	Pd_2dba_3	2	3	1:1.5	0.5	77	83
5	Pd_2dba_3	2	2	1:1	24[a]	39	82
6	^{DMP}DAB-Pd-MAH	2	3	1:1.5	1	86	82
7	^{DMP}DAB-Pd-MAH	2	2	1:1	24[a]	43	81
8	^{Ph}ANDEN-Pd-MAH	2	-	1:1	1	87	81

L = (*S*,*S*)-^{Ph}ANDEN. Reactions run on a 0.2 mmol scale in toluene [0.1 M].
[a] Incomplete conversion after 24 h

PHOX-Pd catalyzed allylation of dimethyl malonate using allylic acetate 16

Condition A: Pd source (2 mol%) and PHOX ligand (2.5 mol% if required) in degassed CH_2Cl_2 (0.3 mL) stirred at rt for 2 h, or was heated at 50 °C for 2 h, or added as a solid as indicated. The catalyst solution or solid was then added to a mixture of (±)-(*trans*)-1,3-diphenylallyl acetate (76 mg, 0.3 mmol), dimethyl malonate (118 mg, 102 µL, 0.9 mmol), *N,O*-bis(trimethylsilyl)acetamide (BSA, 183 mg, 220 µL, 0.9 mmol), and KOAc (0.6 mg, 0.006 mmol, 2 mol%) in degassed CH_2Cl_2 (0.7 mL). The reaction was stirred at rt until complete (reaction monitored by TLC). The reaction was then diluted with Et_2O (20 mL) and washed with a saturated aqueous solution of NH_4Cl. The organic layer was dried over anhydrous Mg_2SO_4, filtered and the solvent removed under reduced preasure. The residue was purified by flash column chromatography over silica gel eluting with hexane and EtOAc (95:5) to recover the title compound.

Condition B: In a microwave vial, KH (30% in mineral oil, 120 mg, 0.9 mmol, 3 equiv.) was triturated with hexane before being suspended in THF (2 mL). Dimethyl malonate (118 mg, 102 µL, 0.9 mmol, 3 equiv.) was then added dropwise and the reaction stirred for 20 min at rt. (±)-(*trans*)-1,3-diphenylallyl acetate (76 mg, 0.3 mmol) was then added. Separately the Pd source (2 mol%) and PHOX ligand (2.5 mol% if required) in degassed THF (1 mL) was heated at 50 °C for 2 h prior to addition to the reaction mixture as a solution, or added as a solid to the reaction mixture along with 1 mL of additional THF as indicated. The reaction mixture was stirred at rt for the indicated time. H_2O (20 mL) was then added and the mixture extracted with CH_2Cl_2. The combined organics were dried over anhydrous $MgSO_4$, filtered, and the solvent removed under reduced pressure. The residue was purified by flash column chromatography over silica gel eluting with hexane and Et_2O (95:5) to recover the title compound.

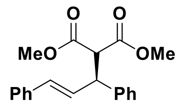

Dimethyl (*S,E*)-2-(1,3-diphenylallyl)malonate (17): Spectroscopic data was in accordance with literature values.[17]

^{1}H NMR (400 MHz, CDCl$_3$) δ 7.34-7.18 (m, 10H), 6.49 (d, *J* = 15.8 Hz, 1H), 6.34 (dd, *J* = 15.7, 8.6 Hz, 1H), 4.28 (dd, *J* = 10.7, 8.7 Hz, 1H), 3.96 (d, *J* = 10.9 Hz, 1H), 3.71 (s, 3H), 3.52 (s, 3H).

^{13}C NMR (101 MHz, CDCl$_3$) δ 168.3, 167.9, 140.3, 137.0, 132.0, 129.3, 128.9, 128.6, 128.0, 127.7, 127.3, 126.5, 57.8, 52.7, 52.6, 49.3.

HPLC: Daicel CHIRALPAK IB, *n*-hexane/*i*-PrOH: 90/10, flow rate = 1 mL/min, T = 35 °C, λ = 210 nm, t_R = 7.36 min (major), t_R = 8.85 min (minor).

Supplementary Table 9. Catalyst System Comparison for Synthesis of **17**

PHOX catalysts

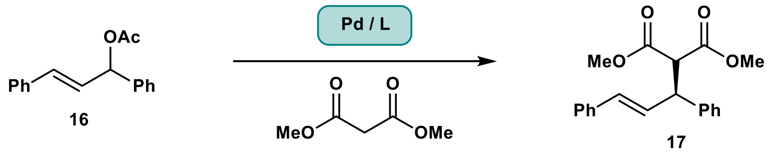

Conditions:	A	B
	BSA (3 equiv), KOAc (2 mol%) CH_2Cl_2 [0.3] M	KH (3 equiv) THF [0.1] M

Entry	Pd source	Pd mol %	L (mol%)	Pd:L	Cond^n	t (h)	Yield %	*ee* %
1	$[Pd(allyl)Cl]_2$	2[a]	(*R*)-^{iPr}PHOX (2.5)	1:1.25	A	1	93	95
2	Pd_2dba_3	2[a]	(*R*)-^{iPr}PHOX (2.5)	1:1.25	A	1	80	95
3	^{DMP}DAB-Pd-MAH	2[a]	(*R*)-^{iPr}PHOX (2.5)	1:1.25	A	24	81	97
4	$[Pd(allyl)Cl]_2$	2[b]	(*R*)-^{iPr}PHOX (2.5)	1:1.25	A	24	67	94
5	Pd_2dba_3	2[b]	(*R*)-^{iPr}PHOX (2.5)	1:1.25	A	24	59	97
6	^{DMP}DAB-Pd-MAH	2[b]	(*R*)-^{iPr}PHOX (2.5)	1:1.25	A	24	89	97
7	(*S*)-^{iPr}PHOX-Pd-MAH	2[c]	-	1:1	A	24	84	-96
8	$[Pd(allyl)Cl]_2$	2[a]	(*R*)-^{tBu}PHOX (2.5)	1:1.25	A	1	89	96
9	^{DMP}DAB-Pd-MAH	2[a]	(*R*)-^{tBu}PHOX (2.5)	1:1.25	A	24	72	85
10	(*S*)-^{tBu}PHOX-Pd-MAH	2[c]	-	1:1	A	24	80	-82
11	$[Pd(allyl)Cl]_2$	2[a]	(*R*)-^{iPr}PHOX (2.5)	1:1.25	B	1	94	96
12	(*S*)-^{iPr}PHOX-Pd-MAH	2[c]	-	1:1	B	1	89	-96
13	(*S*)-^{tBu}PHOX-Pd-MAH	2[c]	-	1:1	B	1	90	-93

[a]Pd and L heated in reaction solvent at 50 °C for 2 h prior to addition of SM.
[b]Pd and L stirred in reaction solvent at rt for 2 h prior to addition of SM.
[c]Pre-catalyst added as a solid to SM at rt.

Improved Pd-AAA of furanone derivatives

3-Bromo-2,5-dihydrofuran-2-one: To a solution of 2(5*H*)-furanone (2.0 mL, 28.2 mmol, 1.0 equiv.) in

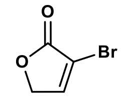

benzene (30 mL), was added bromine (1.70 mL, 31.0 mmol, 1.1 equiv.). The solution was stirred for 24 h at rt and was then cooled to 0 °C. Pyridine (6.80 mL, 84.6 mmol, 3.0 equiv.) was added dropwise and the mixture was stirred at 0 °C for 2 h and then at rt for another 2 h. The reaction mixture was cooled to 0 °C, filtered through a pad of silica gel to remove the pyridine salts, and the pad was rinsed with cold Et_2O. The filtrate was concentrated under reduced pressure and the crude was purified by flash column chromatography over silica gel to yield the title compound (3.76 g, 23.1 mmol, 82%) as a white/yellowish solid. The spectroscopic data of the product were identical with those reported in the literature.[18]

^{1}H NMR (400 MHz, $CDCl_3$) δ 7.64 (t, *J* = 1.9 Hz, 1H), 4.86 (d, *J* = 1.9 Hz, 2H).

^{13}C NMR (101 MHz, CDCl3) δ 169.0, 149.4, 113.1, 71.7.

3-Phenyl-2,5-dihydrofuran-2-one (18): Synthesised following an adapted literature procedure.[19]

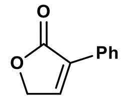

To a solution of 3-bromo-2,5-dihydrofuran-2-one (163 mg, 1.0 mmol, 1.0 equiv.) in benzene (3 mL) was added phenylboronic acid (182 mg, 1.5 mmol, 1.5 equiv.), $Pd(PPh_3)_4$ (57 mg, 5 mol%) and a solution of Na_2CO_3 (2.0 equiv.) in distilled H_2O (1.0 mL). The reaction mixture was heated in a sealed vessel at 100 °C for 30 min under microwave irradiation (300 W). A saturated aqueous solution of brine was then added and the aqueous phase was extracted with EtOAc. The combined organic phases were dried over anhydrous $MgSO_4$, filtered, and the solvent removed under reduced pressure. The residue was purified by flash column chromatography over silica gel (hexane/EtOAc, 75:25) to yield the title compound (88 mg, 0.55 mmol, 55% yield) as a slightly yellow solid. The spectroscopic data of the product were identical with those reported in the literature.[19]

^{1}H NMR (400 MHz, $CDCl_3$) δ 7.86 (m, 1H), 7.84 (m, 1H), 7.65 (t, *J* = 2.0 Hz, 1H), 7.46-7.36 (m, 3H), 4.92 (d, *J* = 2.0 Hz, 2H).

^{13}C NMR (101 MHz, CDCl3) δ 172.3, 144.5, 131.6, 129.5, 129.3, 128.7, 127.0, 69.6.

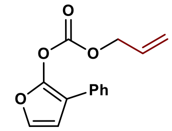

3-Phenylfuran-2-yl prop-2-en-1-ylcarbonate (20): Synthesised following a previously reported procedure.[19]

^{1}H NMR (400 MHz, CDCl$_3$) δ 7.52-7.48 (m, 2H), 7.42-7.37 (m, 2H), 7.28 (m, 1H), 7.18 (d, *J* = 2.3 Hz, 1H), 6.68 (d, *J* = 2.3 Hz, 1H), 5.97 (ddt, *J* = 17.3, 10.5, 5.8 Hz, 1H), 5.43 (dq$_{app}$, *J* = 17.3, 1.3 Hz, 1H), 5.35 (dq$_{app}$, *J* = 10.5, 1.1 Hz, 1H), 4.77 (dt$_{app}$, *J* = 1.3 Hz, 2H).

^{13}C NMR (101 MHz, CDCl$_3$) δ 151.7, 146.3, 136.5, 130.7, 130.4, 128.8, 127.0, 126.4, 120.1, 111.0, 108.1, 70.2.

***tert*-Butyldimethyl((3-phenylfuran-2-yl)oxy)silane (21):** To a solution of 3-phenyl-2,5-dihydrofuran-2-one (0.25 mmol, 1.0 equiv.) in anhydrous CH_2Cl_2 (2.50 mL), was added triethylamine (3.0 equiv.) and TBSOTf (2.0 equiv.). The reaction mixture was heated at reflux overnight. After cooling to rt, the solvent was removed under reduced pressure. The crude residue was purified by suction filtration over silica gel (hexane/triethylamine, 99:1). After evaporation, an internal standard (1,2-dimethoxyethane, DME) was used to quantify the purity of the desired protected product. The crude product was engaged directly in the next step without further purification due to its instability.

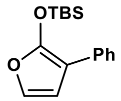

(3*S*)-3-Phenyl-3-(prop-2-en-1-yl)-2,3-dihydrofuran-2-one (19): Prepared using the various methods outlined below. The spectroscopic data of the product were identical with those reported in the literature.[20]

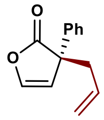

^{1}H NMR (400 MHz, CDCl3) δ 7.51-7.45 (m, 2H), 7.40-7.36 (m, 2H), 7.31 (3m, 1H), 6.93 (d, *J* = 3.5 Hz, 1H), 5.89 (d, *J* = 3.5 Hz, 1H), 5.61 (m, 1H), 5.15 (2m, 1H), 5.12 (2m, 1H), 2.88-2.76 (m, 2H).

^{13}C NMR (101 MHz, CDCl3) δ 178.5, 141.8, 137.9, 131.8, 128.8, 127.9, 126.4, 119.8, 113.2, 54.5, 43.3.

HPLC: Chiralpak IB column, *n*-hexane/*i*-PrOH = 98:2, flow rate = 1 mL/min, T = 30 °C, λ = 230 nm, t_R = 7.15 min (minor) and t_R = 8.00 min (major).

- **Direct approach**

Supplementary Table 10. Catalyst system comparison for the direct allylation of **18** to generate **19**

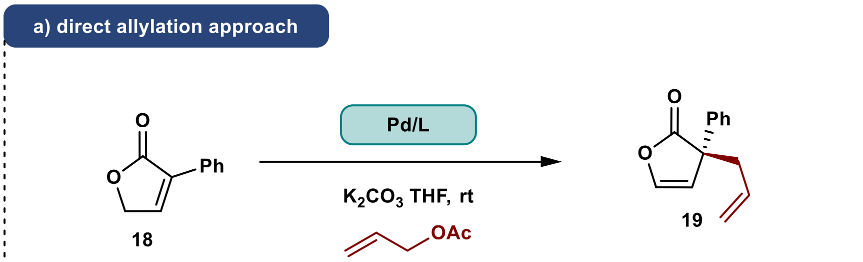

entry	Pd source	Pd (mol%)	L (mol%)	Pd:L	Time	Yield%	ee%
1	Pd_2dba_3	10	1 (12)	1:1.2	24 h	86%	83%
2	Pd_2dba_3	2	1 (2.5)	1:1.25	3d	12%	78%
3	Pd_2dba_3	2	1 (2)	1:1	3d	10%	75%
4	^{Ph}DACH-Pd-MAH	2	-	(1:1)	3d	65%	82%

The catalyst solution was first prepared either by mixing Pd_2dba_3 (indicated mol%) and **L1** (indicated mol%) in THF (0.5 mL) for 30 min at rt or by simply adding ^{Ph}DACH-Pd-MAH (**1**) (3.6 mg, 0.004 mmol, 2 mol%) in a solution THF (0.5 mL). The catalyst solution is then added to a mixture of **18** (32 mg, 0.2 mmol) and K_2CO_3 (41.4 mg, 0.3 mmol, 1.5 equiv.) in THF (1.5 mL), followed by allyl acetate (30 mg, 32 µL, 0.3 mmol, 1.5 equiv.) and the was stirred at rt for the indicated time. The reaction mixture is then filtered through a pad of Celite© and the solvent removed under reduced pressure. The residue is purified by flash column chromatography over silica gel (*n*-hexane/Et_2O, 70:30) to give **19** (indicated yield) as a colourless oil.

- **Pd-DAAA approach**

Supplementary Table 11. Catalyst system comparison for the decarboxylative allylation of **20** to give **19**

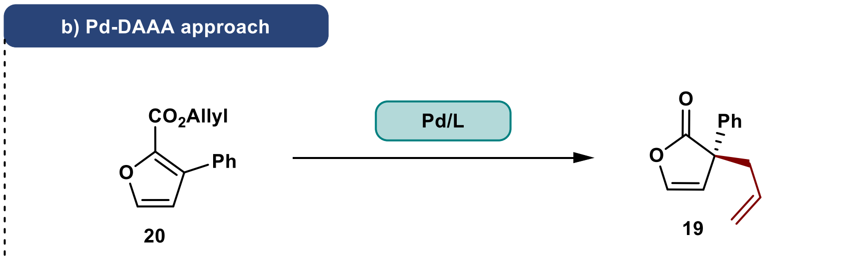

entry	Pd source	Pd (mol%)	L (mol%)	Pd:L	Time	Yield%	ee%
1	Pd_2dba_3	10	1 (12)	1:1.2	1 h	80%	82%
2	Pd_2dba_3	2	1 (2.5)	1:1.25	3 d	<5%	-
3	Pd_2dba_3	2	1 (2)	1:1	3 d	<5%	-
4	^{Ph}DACH-Pd-MAH	2	-	(1:1)	4 h	75%	82%

The catalyst solution was first prepared either by mixing Pd_2dba_3 (indicated mol%) and **L1** (indicated mol%) in THF (0.5 mL) for 30 min at rt or by simply adding ^{Ph}DACH-Pd-MAH (**1**) (2.7 mg, 0.0030 mmol, 2 mol%) in a solution THF (0.5 mL). The catalyst solution was then added to a mixture of **20** (35 mg, 0.15 mmol) in THF (1.5 mL) and the reaction was stirred at rt for the indicated time. The reaction mixture was filtered through a pad of Celite© and the solvent removed under reduced pressure. The residue was purified by flash column chromatography over silica gel (*n*-hexane/Et_2O, 70:30) to give **19** (indicated yield) as a colourless oil.

- **Enol silane nucleophile approach**

Supplementary Table 12. Catalyst system comparison for the allylation of enol silane **21** to give **19**

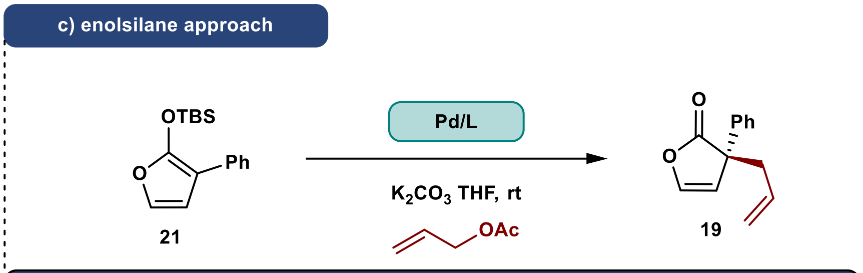

entry	Pd source	Pd (mol%)	L (mol%)	Pd:L	Time	Yield%	ee%
1	Pd_2dba_3	10	1 (12)	1:1.2	1 h	85%	75%
2	Pd_2dba_3	2	1 (2.5)	1:1.25	15 min	70%	78%
3	Pd_2dba_3	2	1 (2)	1:1	2 d	<5%	-
4	^{Ph}DACH-Pd-MAH	2	-	(1:1)	15 min	67%	75%

The catalyst solution was first prepared either by mixing Pd_2dba_3 (indicated mol%) and **L1** (indicated mol%) in THF (0.5 mL) for 30 min at rt or by simply adding ^{Ph}DACH-Pd-MAH (**1**) (3.6 mg, 0.0040 mmol, 2 mol%) in a solution THF (0.5 mL). The catalyst solution was then added to a mixture of **21** (55 mg, 0.2 mmol) and K_2CO_3 (41.4 mg, 0.3 mmol, 1.5 equiv.) in THF (1.5 mL), followed by allyl acetate (30 mg, 32 μL, 0.3 mmol, 1.5 equiv.), and the reaction was stirred at rt for the indicated time. The reaction mixture was filtered through a pad of Celite© and the solvent removed under reduced pressure. The residue was purified by flash column chromatography over silica gel (*n*-hexane/Et_2O, 70:30) to give **19** (indicated yield) as a colourless oil.

Allylation of hydantoin 22

1-Benzyl-3-(*tert*-butyl)-5-phenylimidazolidine-2,4-dione (22). To a solution of methyl 2-benzamido-2-phenylacetate (3.5 g, 13.7 mmol) in THF (30 mL) was added NEt_3 (3.1 ml, 22 mmol, 1.6 equiv.) and *t*BuNCO (2.03 g, 2.3 mL, 20.5 mmol, 1.5 equiv.). The solution was heated at 65 °C for 18 h before being allowed to cool to rt. The solvent was removed under reduced pressure. The residue was re-dissolved in THF (45 mL), and *t*-BuOK (2.3 g, 20.5 mmol, 1.5 equiv.) was added. The reaction mixture was stirred for 1 h at rt. Water (100 mL) was added, and the mixture extracted with CH_2Cl_2 (3 x 100 mL). The combined organic layer was dried over anhydrous $MgSO_4$, filtered, and the solvent removed under reduced pressure. The residue was purified by flash column chromatography over silica gel eluting with *n*-hexane/EtOAc (9:1) to yield the title compound (2.5 g, 7.75 mmol, 57%) as a colourless solid, which could be further purified by recrystallisation from *n*-hexane (~5 mL/g). Spectroscopic data was in accordance with the literature values.[21]

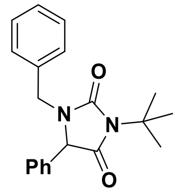

m.p 88-90 °C.

R_f: 0.5 (EtOAc/hexane, 3:7, UV active).

^{1}H NMR (400 MHz, $CDCl_3$) δ 7.44-7.37 (m, 3H), 7.34-7.28 (m, 3H), 7.19-7.14 (m, 2H), 7.14-7.09 (m, 2H), 5.12 (d, *J* = 14.9 Hz, 1H), 4.49 (s, 1H), 3.69 (d, *J* = 14.9 Hz, 1H), 1.65 (s, 9H).

^{13}C NMR (101 MHz, $CDCl_3$) δ 172.2, 157.5, 135.8, 133.6, 129.4, 129.3, 129.0, 128.6, 128.1, 127.6, 62.5, 58.4, 44.5, 28.9.

HRMS (ESI): m/z calcd for $C_{20}H_{22}N_2O_2$, $[M + H]^+$: 323.1760, found: 323.1728.

Parallel screening experiments

All screening experiments were performed in a Radleys GreenHouse Parallel Synthesiser under N_2 (Supplementary Figure 115)

- **Round 1: Chiral ligand identification.**

The following stock solutions were prepared in THF and handled under air:

^{DMP}**DAB-Pd-MAH**: 5.0 mg/mL (0.010 M)

L1: 21 mg/mL (0.030 M)

L2: 24 mg/mL (0.030 M)

L3: 24 mg/mL (0.030 M)

L4: 24 mg/mL (0.030 M)

L5: 11 mg/mL (0.030 M)

L6: 11 mg/mL (0.030 M)

1-Benzyl-3-(*tert*-butyl)-5-phenylimidazolidine-2,4-dione (**22**): 64.5 mg/mL (0.200 M)

Allyl acetate: 400 mg/mL (4.00 M)

The ^{DMP}DAB-Pd-MAH solution (200 µL, 1 mg, 2 mol%) was dispensed followed by the appropriate ligand solution (100 µL, 3 mol%) and allowed to stir for ~5 min before the addition of **22** (500 µL, 0.1 mmol). NaHMDS (150 µL, 1 M, 1.5 equiv. commercial solution in THF) was then added, and the mixture stirred for 1 h. Finally, the solution of allyl acetate (50 µL, 2 equiv.) was added, and the reaction mixtures were stirred for 18 h at rt. MeOH (1 mL) was added, and the solvent removed by blow down. Yields were obtained by ^{1}H NMR spectroscopy using 1,2-dimethoxyethane (DME) as an internal standard. Successful reactions (>10% yield) were purified by a short preparative TLC plate and enantiopurity was determined by chiral HPLC (Supplementary Figure 114).

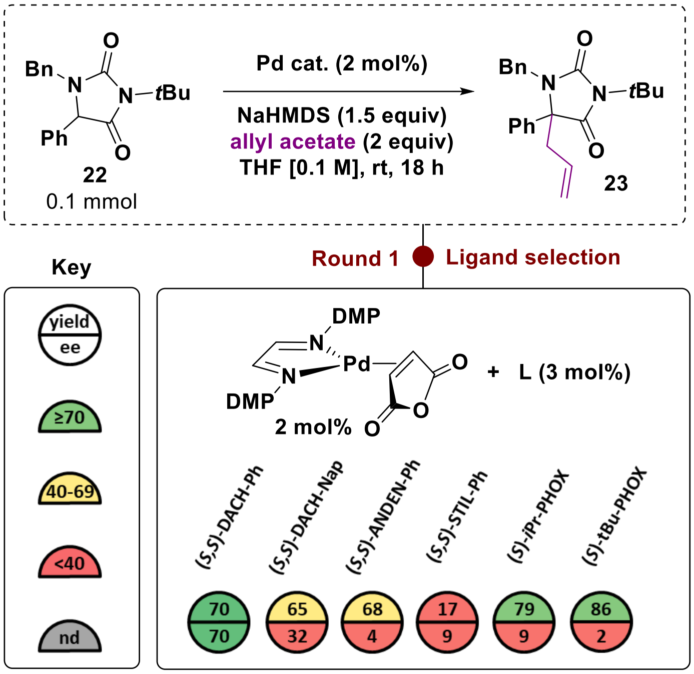

Supplementary Figure 114: Chiral ligand identification using ^{DMP}DAB-Pd-MAH as a Pd(0) precursor.

Round 2: Solvent/base array.

The following stock solutions were prepared in THF, CH_2Cl_2, toluene, and Et_2O and handled under air:

^{Ph}**DACH-Pd-MAH** (**1**): 4.5 mg/mL (0.0050 M)

Allyl acetate: 134 mg/mL (1.33 M)

1-Benzyl-3-(*tert*-butyl)-5-phenylimidazolidine-2,4-dione (**22**): 107.2 mg/mL (0.333 M)

Bases were used as either commercial 1 M solutions in THF (LHMDS, NaHMDS, KHMDS), or dissolved in the appropriate solvent to a 1 M concentration (*t*BuONa, DBU). If the base proved to be insoluble in the required solvent, then a 1 M solution was made in a different solvent, the required amount added to a reaction vial, and the solvent removed by a stream of nitrogen followed by reduced pressure.

Representative procedure: 300 µL of SM stock solution was added to a well followed by 150 µL of base solution (1 M) and stirred at rt for 1 h. ^{Ph}DACH-Pd-MAH (**L1**) stock solution (400 µL) was added followed by allyl acetate stock solution (150 µL). The reactions were stirred for 18 h at rt. MeOH (1 mL) was added, and the solvent removed by blow down. Yields were obtained by H^1 NMR using DME as an internal standard. Successful reactions (>10% yield) were purified by a short preparative TLC plate and enantiopurity was determined by chiral HPLC.

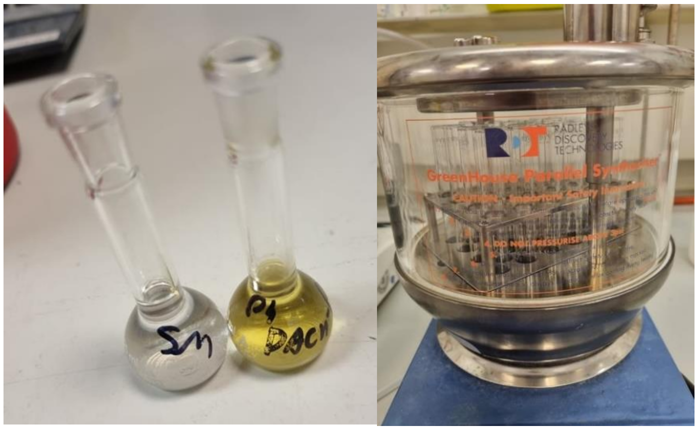

A B

Supplementary Figure 115: (**A**) Stock solutions of SM and ^{Ph}DACH-Pd-MAH handled in air. (**B**) Experimental set up in Radleys GreenHouse Parallel Synthesiser.

Optimization at low catalyst loading

Representative procedure for 0.2 mol% Pd, 0.2 M concentration (Supplementary Table 13, entry 5): To a solution of **22** (64 mg, 0.2 mmol) in THF (340 µL) at 0 °C, was added NaHMDS (1 M in THF, 300 µL, 1.5 equiv.) followed by stirring for 30 min. A solution of ^{Ph}DACH-Pd-MAH (**1**) (1 mg/mL in THF, 360 µL, 0.4 µmol, 0.2 mol%) was then added, followed by allyl acetate (43 µL, 0.4 mmol, 2 equiv.). The reaction mixture was stirred at 0 °C for 24 h. H_2O (10 mL) was added and then the mixture was extracted with CH_2Cl_2 (3 x 10 mL). The combined organic layers were dried over $MgSO_4$, filtered, and the solvent removed under reduced pressure. The residue was purified by flash column chromatography over silica gel eluting with Hexane and EtOAc (9:1) to recover the title compound (65 mg, 0.18 mmol, 90%) as a colourless oil. Enantiopurity was determined by chiral HPLC analysis.

Supplementary Table 13. Catalyst loading optimization for the allylation of hydantoin **22** to give **23**

Round 3

Entry	Pd (mol %)	Time (h)	T (°C)	Yield %	ee %
1	1	1h	0	91	76
2	1	18	-40	94	75
3	0.2	24	0	90	88
4	0.1	48	0	31	82
5	0.2[a]	24	0	90	89

reaction conditions: SM 0.2 mmol, 1.5 equiv NaHMDS (1 M in THF), 2 equiv allyl acetate, and idicated catalyst loading, in THF (0.1 M). [a] reaction at (0.2 M)

Furthermore, the conditions from Entry 1 were repeated a total of five times by two different experimentalists, with yields (91%, 91%, 90%, 89%, 92%) and *ee*'s (76%, 78%, 78%, 77%, 79%) that are highly consistent. Yield = 90.5 ± 1.5%; *ee* = 77.5 ± 1.5%.

Gram scale synthesis of 23

5-Allyl-1-benzyl-3-(*tert*-butyl)-5-phenylimidazolidine-2,4-dione (23): To a solution of **22** (1.29 g, 4 mmol, 1 equiv.) in THF (14 mL) at 0 °C was added NaHMDS (1 M in THF, 6 mL, 6 mmol, 1.5 equiv.) and the solution was stirred for 1 h. ^{Ph}DACH-Pd-MAH (**1**) (7.2 mg, 0.008 mmol, 0.2 mol%) was then added as a solid followed by allyl acetate (860 µL, 8 mmol, 2 equiv.). The reaction mixture was stirred at 0 °C for 24 h, at which time the reaction was deemed complete by TLC analysis. H_2O (50 mL) was added and the mixture extracted with CH_2Cl_2 (3 x 50 mL). The combined organic layers were then washed with 10 % w/w aqueous citric acid (2 x 50 mL) and a saturated aqueous solution of brine (50 mL). The organic phase was dried over anhydrous $MgSO_4$, filtered, and the solvent removed under reduced pressure. The residue was purified by flash column chromatography over silica gel eluting with hexane and EtOAc (100:0 to 80:20) to recover the title compound (1.35g, 3.7 mmol, 93 %) as a colourless oil. Spectroscopic data was in accordance with the literature values.[21]

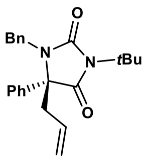

R_f: 0.55 (EtOAc:Hex, 3:7, UV active)

^{1}H NMR (400 MHz, CDCl$_3$) δ 7.30-7.24 (m, 3H), 7.16-7.09 (m, 7H), 5.27-5.15 (m, 1H), 4.97-4.81 (m, 2H), 4.60 (d, *J* = 15.1 Hz, 1H), 3.82 (d, *J* = 15.1 Hz, 1H), 2.96 (dd, *J* = 13.7, 7.7 Hz, 1H), 2.59 (dd, *J* = 13.7, 6.4 Hz, 1H), 1.56 (s, 9H).

^{13}C NMR (101 MHz, CDCl$_3$) δ 174.7, 158.1, 137.4, 137.0, 130.2, 129.4, 129.2, 128.8, 128.5, 127.6, 126.6, 121.0, 69.6, 58.4, 44.7, 37.1, 28.9.

HRMS (ESI): m/z calcd for $C_{23}H_{27}N_2O_2$, $[M + H]^+$: 363.2072, found 363.2080.

HPLC: Daicel CHIRALPAK IC, *n*-hexane/*i*-PrOH: 90/10, flow rate = 1 mL/min, T = 35 °C, λ = 254 nm, t_R = 4.91 min (major), t_R = 5.71 min (minor).

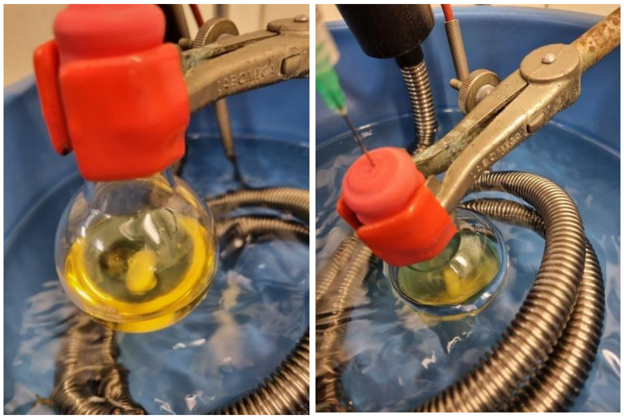

Supplementary Figure 116: Experimental setup for gram scale alkylation

Representative HPLC chromatograms for enantiopurity determination

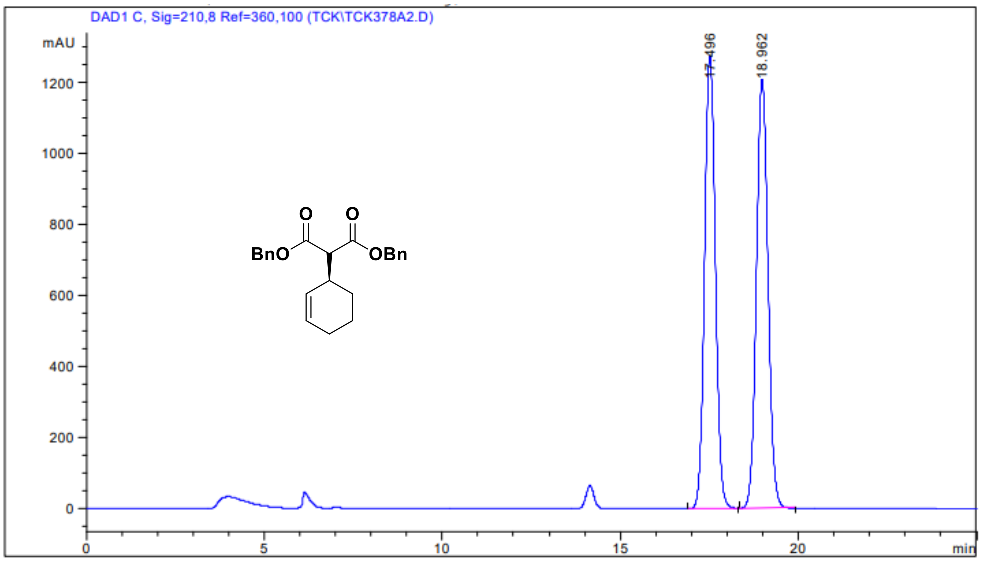

Signal 1: DAD1 C, Sig=210,8 Ref=360,100

Peak #	RetTime [min]	Type	Width [min]	Area [mAU*s]	Height [mAU]	Area %
1	17.496	BB	0.3305	2.69347e4	1275.05286	49.6039
2	18.962	BP	0.3506	2.73649e4	1206.39246	50.3961

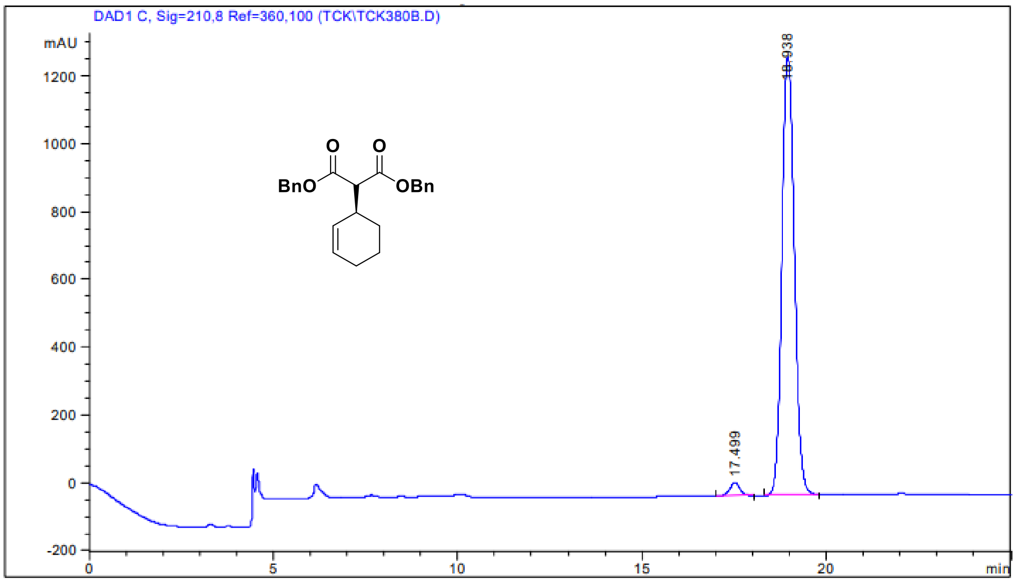

Signal 1: DAD1 C, Sig=210,8 Ref=360,100

Peak #	RetTime [min]	Type	Width [min]	Area [mAU*s]	Height [mAU]	Area %
1	17.499	PB	0.2976	753.04895	38.60555	2.4018
2	18.938	BB	0.3718	3.06004e4	1294.34900	97.5982

Supplementary Figure 117: *Top:* HPLC UV/Vis absorbance trace (210 nm) for racemic **8**. *Bottom:* Representative HPLC trace for **8** produced using asymmetric allylic allylation with 95% *ee* (Supplementary Table 5, entry 1).

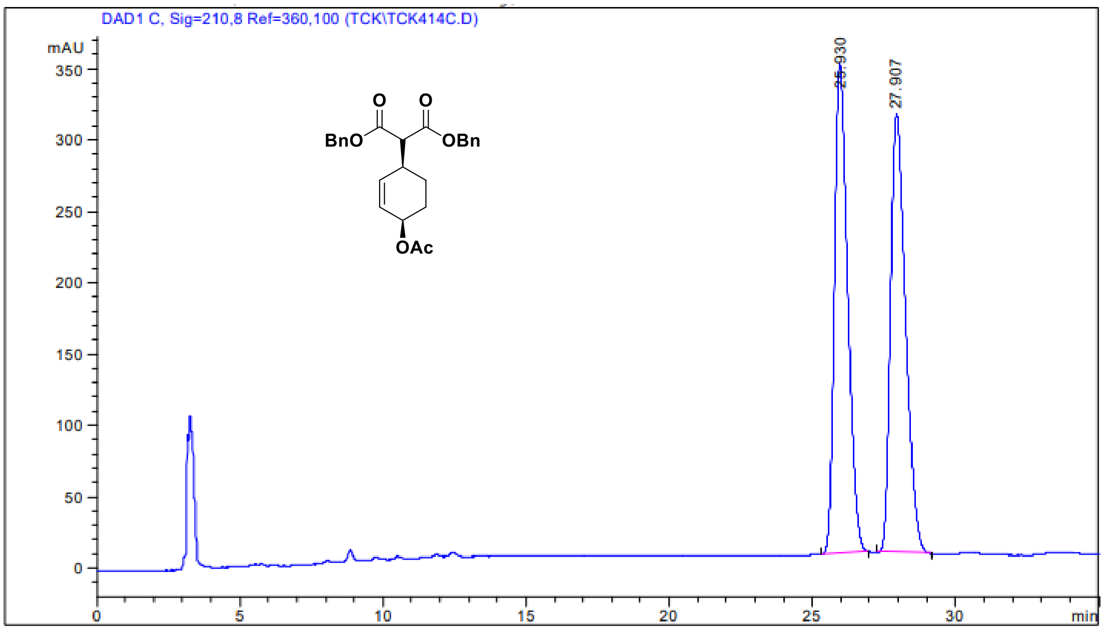

Signal 1: DAD1 C, Sig=210,8 Ref=360,100

Peak #	RetTime [min]	Type	Width [min]	Area [mAU*s]	Height [mAU]	Area %
1	25.930	BB	0.5012	1.11827e4	344.04471	49.8697
2	27.907	PB	0.5648	1.12411e4	307.27789	50.1303

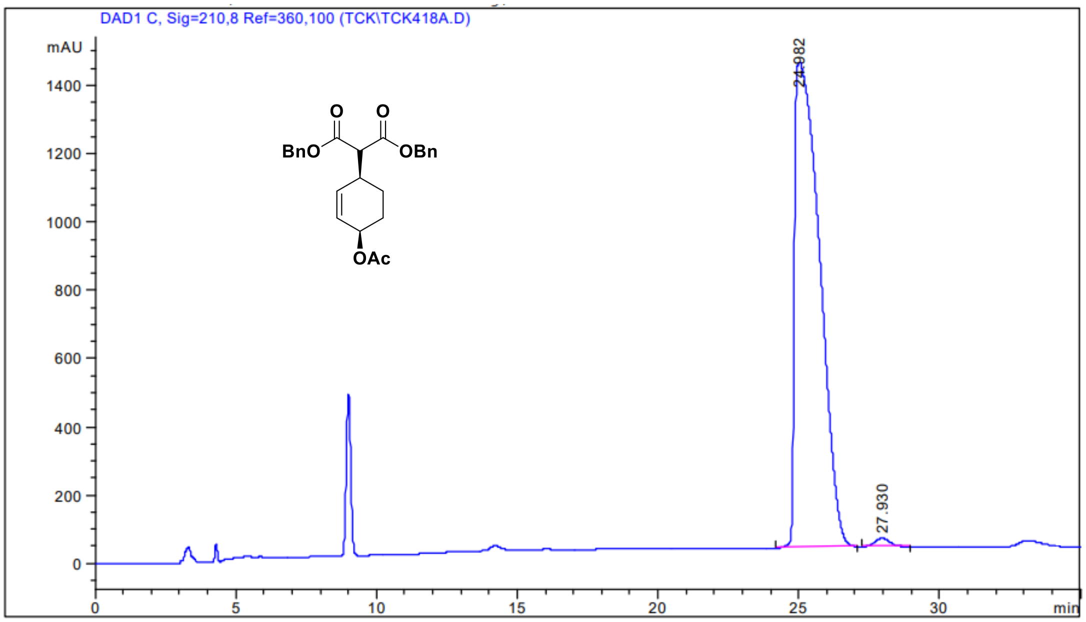

Signal 1: DAD1 C, Sig=210,8 Ref=360,100

Peak #	RetTime [min]	Type	Width [min]	Area [mAU*s]	Height [mAU]	Area %
1	24.982	PB	0.7774	8.77486e4	1423.07446	98.9052
2	27.930	BB	0.5149	971.28748	25.24310	1.0948

Supplementary Figure 118: *Top:* HPLC UV/Vis absorbance trace (210 nm) for racemic **10**. *Bottom:* Representative HPLC trace for **10** produced using asymmetric allylic allylation with 95% *ee* (Supplementary Table 6, entry 8).

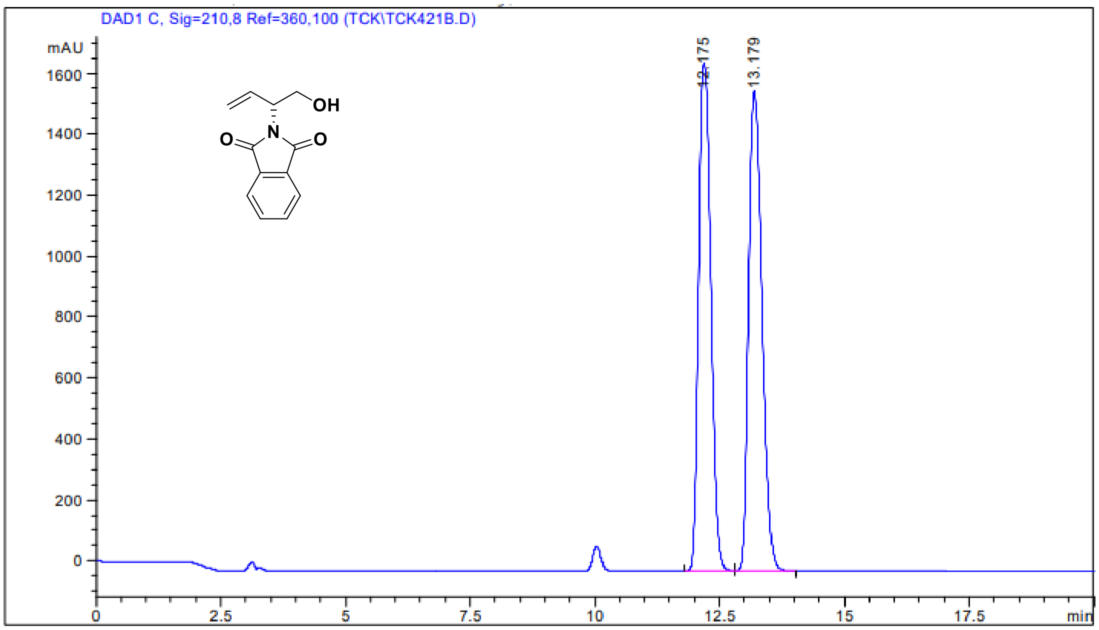

Signal 1: DAD1 C, Sig=210,8 Ref=360,100

Peak #	RetTime [min]	Type	Width [min]	Area [mAU*s]	Height [mAU]	Area %
1	12.175	BV	0.2725	2.84139e4	1669.89453	49.1790
2	13.179	VB	0.2934	2.93626e4	1575.64563	50.8210

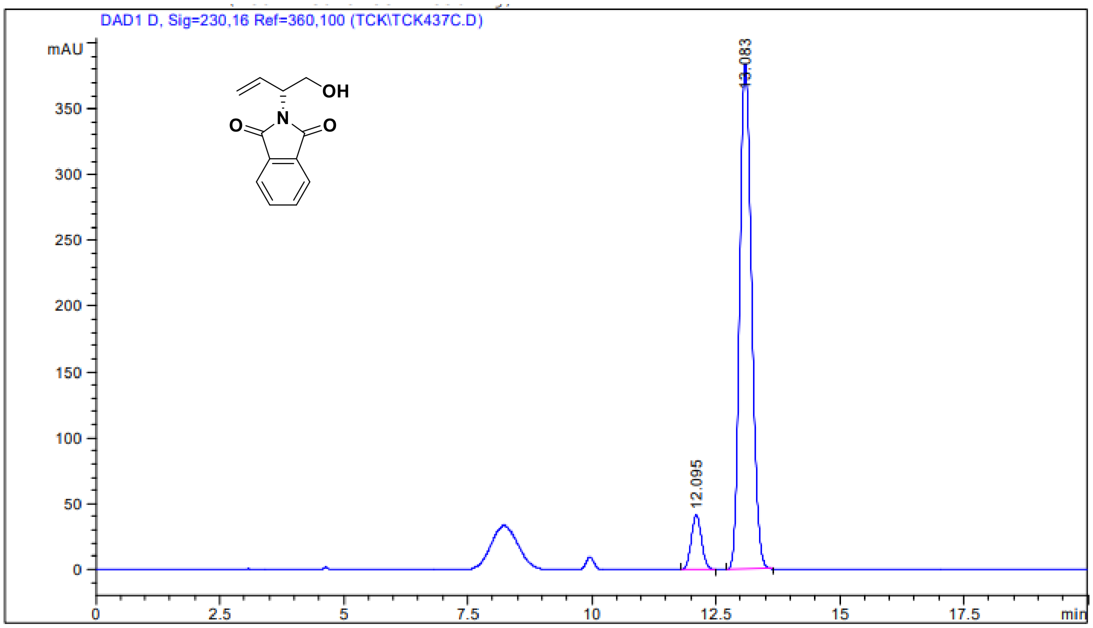

Signal 1: DAD1 D, Sig=230,16 Ref=360,100

Peak #	RetTime [min]	Type	Width [min]	Area [mAU*s]	Height [mAU]	Area %
1	12.095	BB	0.2299	614.46600	41.70309	8.8981
2	13.083	BB	0.2548	6291.10352	384.57986	91.1019

Supplementary Figure 119: *Top:* HPLC UV/Vis absorbance trace (210 nm) for racemic **13**. *Bottom:* Representative HPLC trace for **13** produced using asymmetric allylic allylation with 82% *ee* (Supplementary Table 7, entry 3).

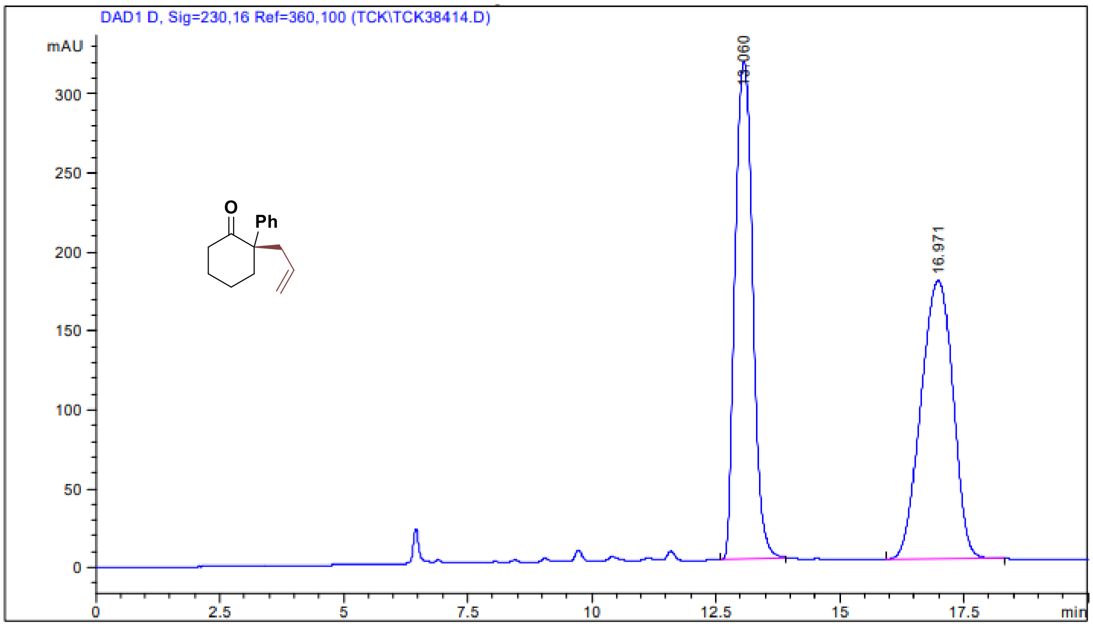

Signal 1: DAD1 D, Sig=230,16 Ref=360,100

Peak #	RetTime [min]	Type	Width [min]	Area [mAU*s]	Height [mAU]	Area %
1	13.060	BB	0.4025	7765.65430	316.27905	49.3002
2	16.971	BB	0.7308	7986.11621	176.87541	50.6998

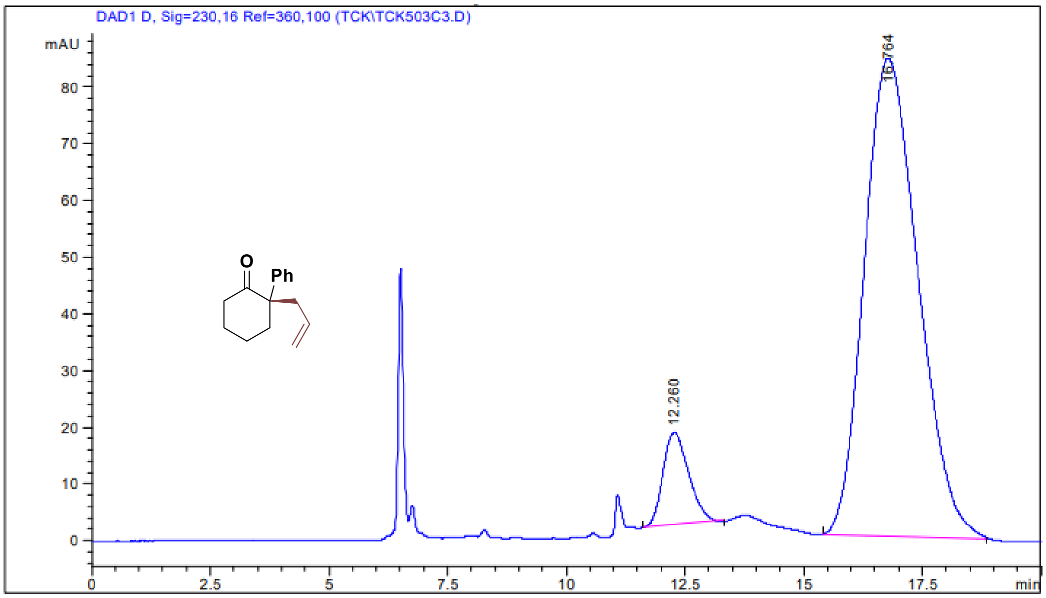

Signal 1: DAD1 D, Sig=230,16 Ref=360,100

Peak #	RetTime [min]	Type	Width [min]	Area [mAU*s]	Height [mAU]	Area %
1	12.260	PP	0.5539	620.39142	16.45427	8.5191
2	16.764	BB	1.1106	6661.98926	84.42743	91.4809

Supplementary Figure 120: *Top:* HPLC UV/Vis absorbance trace (230 nm) for racemic **15**. *Bottom:* Representative HPLC trace for **15** produced using asymmetric allylic allylation with 83% *ee* (Supplementary Table 8, entry 4).

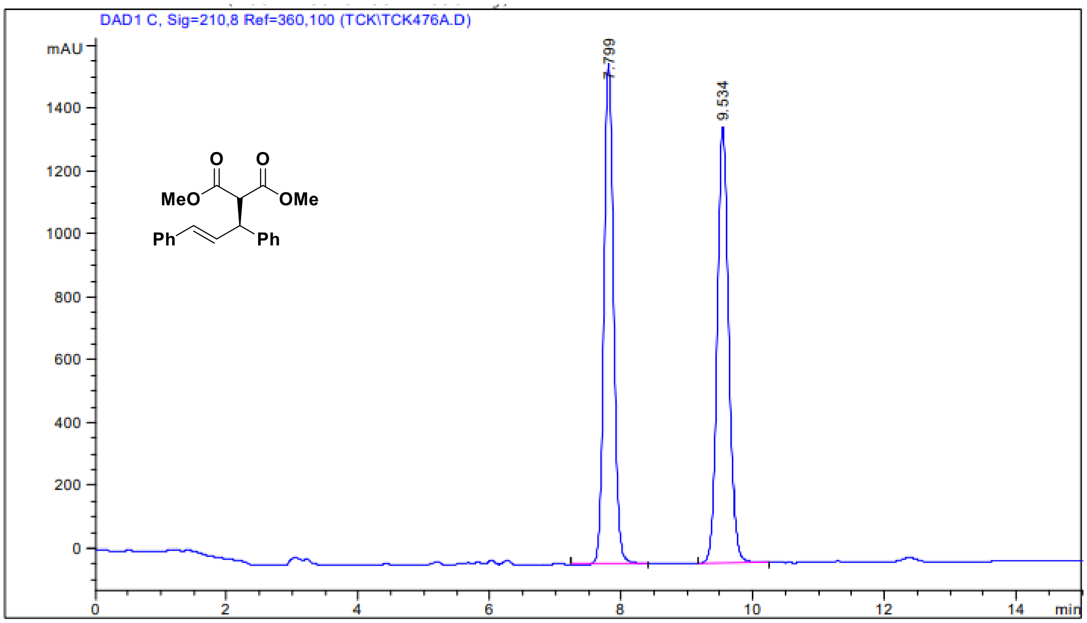

Signal 1: DAD1 C, Sig=210,8 Ref=360,100

Peak #	RetTime [min]	Type	Width [min]	Area [mAU*s]	Height [mAU]	Area %
1	7.799	VB	0.1593	1.63217e4	1595.67200	49.0262
2	9.534	PB	0.1885	1.69701e4	1389.36975	50.9738

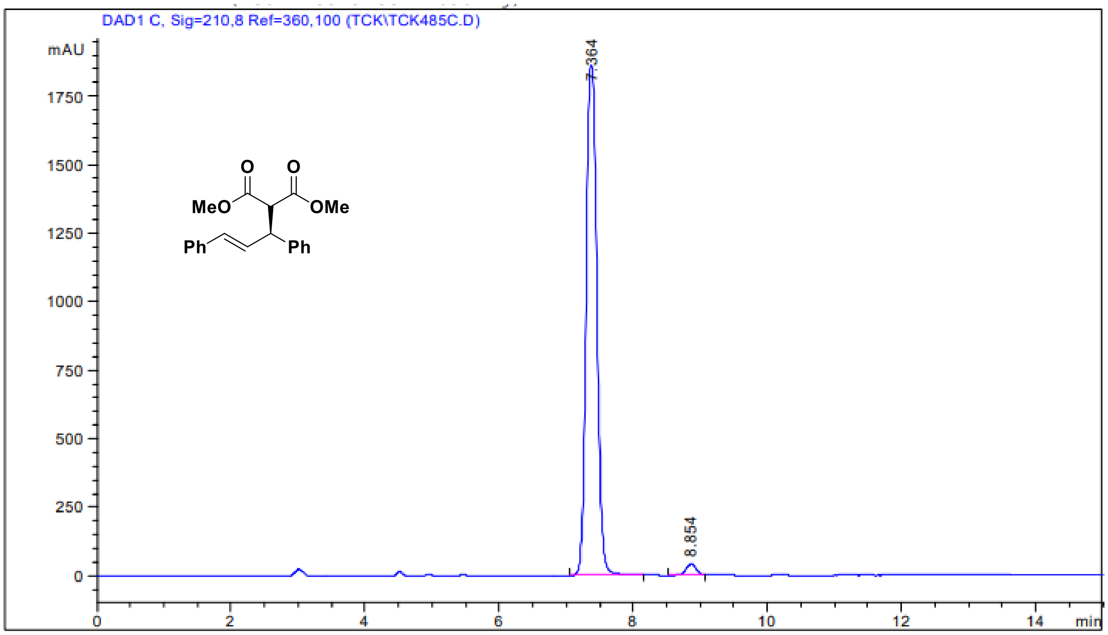

Signal 1: DAD1 C, Sig=210,8 Ref=360,100

Peak #	RetTime [min]	Type	Width [min]	Area [mAU*s]	Height [mAU]	Area %
1	7.364	BB	0.1790	2.09470e4	1865.80811	97.8628
2	8.854	PV	0.1635	457.45615	43.20699	2.1372

Supplementary Figure 121: *Top:* HPLC UV/Vis absorbance trace (210 nm) for racemic **17**. *Bottom:* Representative HPLC trace for **17** produced using asymmetric allylic allylation with 96% *ee* (Supplementary Table 9, entry 12).

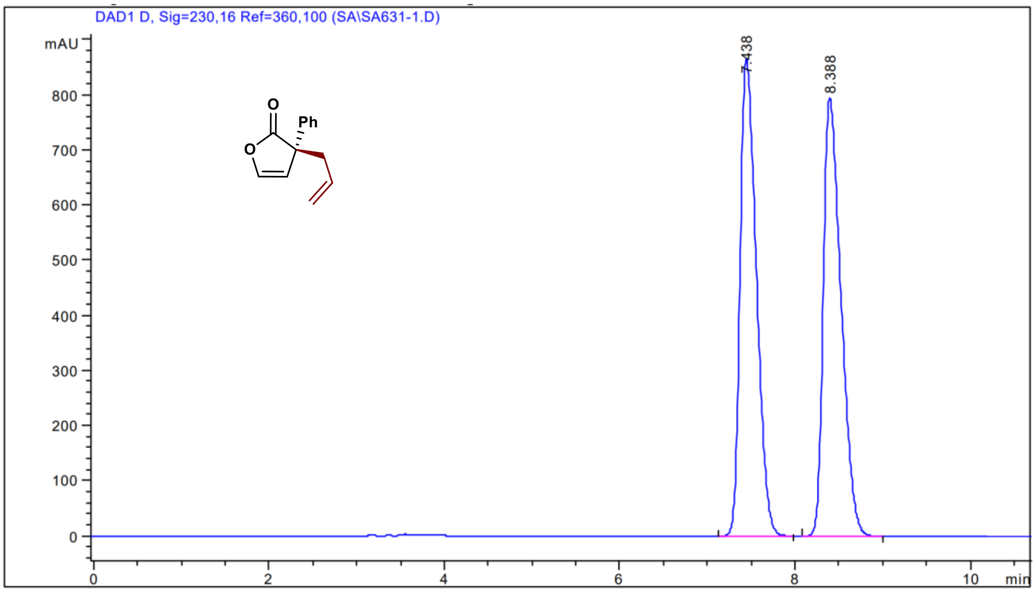

Signal 1: DAD1 D, Sig=230,16 Ref=360,100

Peak #	RetTime [min]	Type	Width [min]	Area [mAU*s]	Height [mAU]	Area %
1	7.438	BB	0.2005	1.11912e4	867.63470	49.9613
2	8.388	BB	0.2206	1.12085e4	794.51630	50.0387

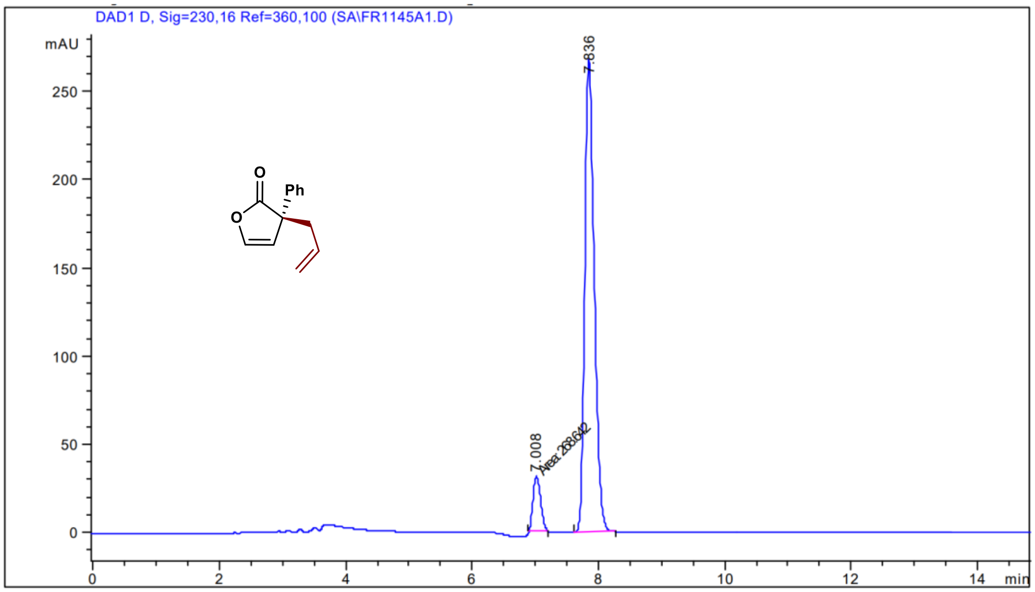

Signal 1: DAD1 D, Sig=230,16 Ref=360,100

Peak #	RetTime [min]	Type	Width [min]	Area [mAU*s]	Height [mAU]	Area %
1	7.008	MM	0.1422	268.64197	31.48771	8.7152
2	7.836	BB	0.1602	2813.80005	268.62350	91.2848

Supplementary Figure 122: *Top:* HPLC UV/Vis absorbance trace (230 nm) for racemic **19**. *Bottom:* Representative HPLC trace for **19** produced using asymmetric allylic allylation with 85% *ee* (Supplementary Table 10, entry 1).

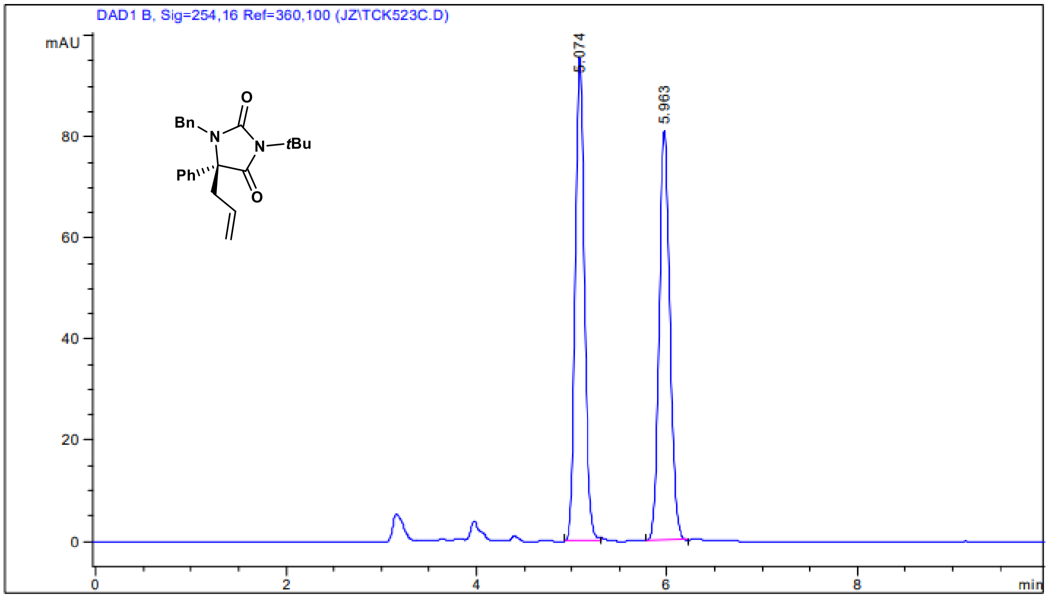

Signal 1: DAD1 B, Sig=254,16 Ref=360,100

Peak #	RetTime [min]	Type	Width [min]	Area [mAU*s]	Height [mAU]	Area %
1	5.074	PB	0.1015	636.92737	95.88192	49.9556
2	5.963	BP	0.1218	638.05951	81.07191	50.0444

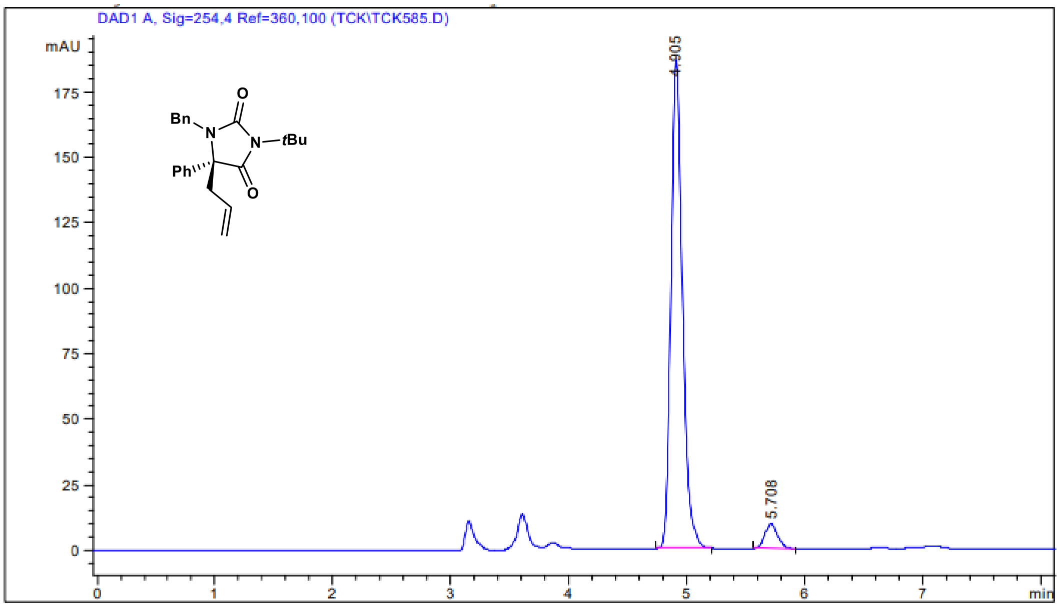

Signal 1: DAD1 A, Sig=254,4 Ref=360,100

Peak #	RetTime [min]	Type	Width [min]	Area [mAU*s]	Height [mAU]	Area %
1	4.905	BB	0.1025	1256.95691	186.85724	94.5005
2	5.708	BB	0.1208	73.14969	9.40305	5.4995

Supplementary Figure 123: *Top:* HPLC UV/Vis absorbance trace (254 nm) for racemic **23**. *Bottom:* HPLC trace for **23** produced on gram scale using asymmetric allylic allylation with 89% *ee* (Supplementary Table 13, entry 5).

X-ray Crystallographic Details

(*S,S*)-^{NAP}DACH-Pd-MAH (2). Single crystals of $C_{60}H_{54}N_2O_6P_2Pd$ (**2**) were selected using a MitEGen loop and paratone oil. A suitable crystal was selected and run on a Bruker APEX-II CCD diffractometer. The crystal was kept at 100.15 K during data collection. Using Olex2[22], the structure was solved with the olex2.solve[23] structure solution program using Charge Flipping and refined with the XL[24] refinement package using Least Squares minimisation.

Crystal structure determination of (2)

Crystal Data for $C_{60}H_{54}N_2O_6P_2Pd$ (M =1067.39 g/mol): orthorhombic, space group $P2_12_12_1$ (no. 19), a = 13.443(10) Å, b = 16.517(11) Å, c = 23.465(17) Å, V = 5210(6) Å^3, Z = 4, T = 100.15 K, μ(MoKα) = 0.471 mm^{-1}, $Dcalc$ = 1.361 g/cm^3, 83698 reflections measured (3.016° ≤ 2Θ ≤ 54.84°), 11652 unique (R_{int} = 0.0456, R_{sigma} = 0.0325) which were used in all calculations. The final R_1 was 0.0252 ($I > 2\sigma(I)$) and wR_2 was 0.0569 (all data).

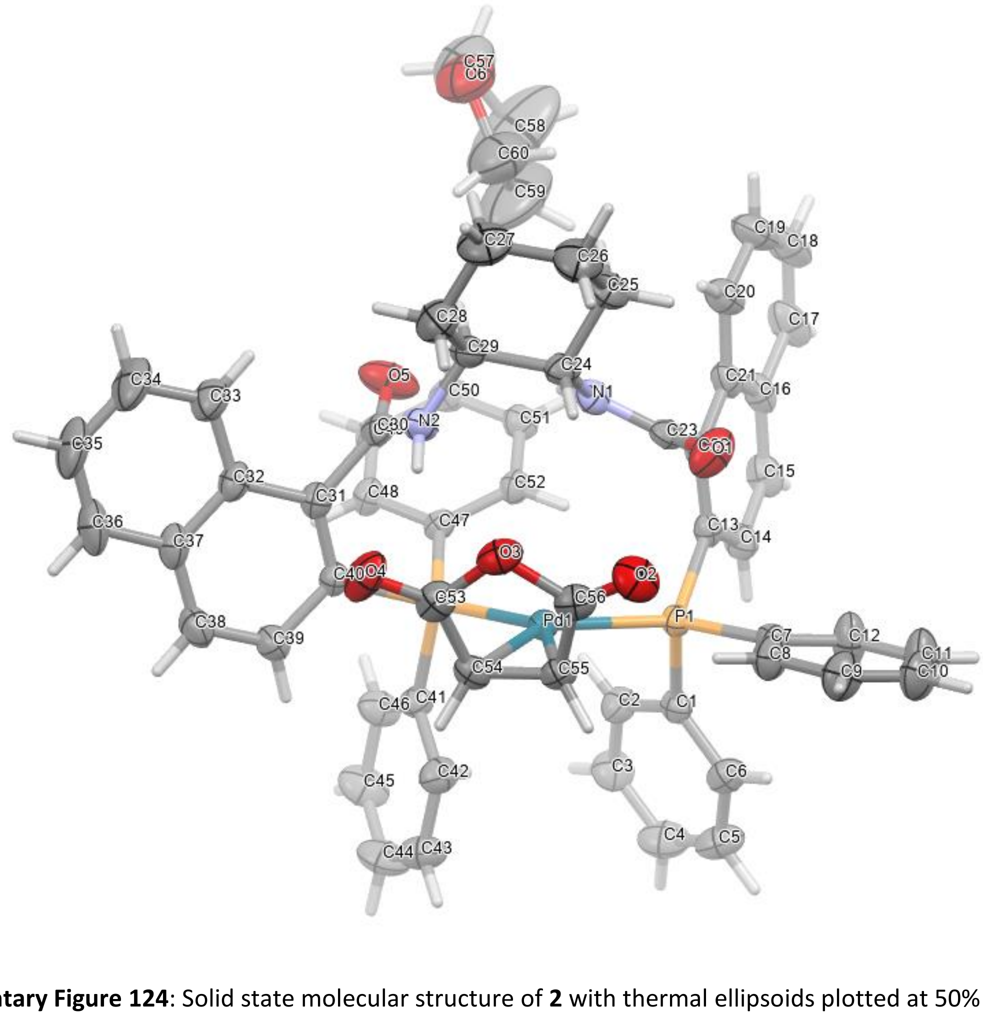

Supplementary Figure 124: Solid state molecular structure of **2** with thermal ellipsoids plotted at 50% probability. Hydrogen atoms are shown as capped sticks. Asymmetric unit contains one molecule of THF solvent. Image generated using Mercury 2021.3.0. CCDC deposition number 2258958.

Supplementary Table 14. Crystal data and structure refinement for 2.

Identification code	**2**
Empirical formula	$C_{60}H_{54}N_2O_6P_2Pd$
Formula weight	1067.39
Temperature/K	100.15
Crystal system	orthorhombic
Space group	$P2_12_12_1$
a/Å	13.443(10)
b/Å	16.517(11)
c/Å	23.465(17)
α/°	90
β/°	90
γ/°	90
Volume/Å^3	5210(6)
Z	4
ρ_{calc}g/cm^3	1.361
μ/mm^{-1}	0.471
F(000)	2208.0
Crystal size/mm^3	0.05 × 0.03 × 0.02
Radiation	MoKα (λ = 0.71073)
2Θ range for data collection/°	3.016 to 54.84
Index ranges	-17 ≤ h ≤ 17, -19 ≤ k ≤ 21, -30 ≤ l ≤ 30
Reflections collected	83698
Independent reflections	11652 [R_{int} = 0.0456, R_{sigma} = 0.0325]
Data/restraints/parameters	11652/0/641
Goodness-of-fit on F^2	1.025
Final R indexes [I>=2σ (I)]	R_1 = 0.0252, wR_2 = 0.0545
Final R indexes [all data]	R_1 = 0.0322, wR_2 = 0.0569
Largest diff. peak/hole / e Å^{-3}	0.23/-0.26
Flack parameter	-0.021(16)

Supplementary Table 15. Fractional Atomic Coordinates (×10^4) and Equivalent Isotropic Displacement Parameters (Å^2×10^3) for **2**. U_{eq} is defined as 1/3 of of the trace of the orthogonalised U_{IJ} tensor.

Atom	*x*	*y*	*z*	U(eq)
C_1	4584(2)	4489.6(17)	3591.2(13)	29.1(6)
C_2	5290(2)	5060(2)	3401.8(14)	36.9(7)
C_3	6136(3)	5234(2)	3722.6(16)	45.5(8)
C_4	6302(3)	4835(2)	4238.5(17)	53.6(10)
C_5	5623(3)	4273(2)	4425.2(16)	49.8(9)
C_6	4762(3)	4100.9(18)	4107.6(13)	36.8(7)
C_7	2708(2)	3634.9(19)	3598.7(12)	32.9(6)
C_8	2495(3)	2828(2)	3485.5(15)	42.9(8)
C_9	1920(3)	2364(2)	3859.4(17)	51.8(9)
C_{10}	1548(3)	2713(2)	4352.8(18)	58.6(10)
C_{11}	1748(3)	3513(3)	4476.2(19)	66.8(12)

Supplementary Table 15. Fractional Atomic Coordinates (×10[4]) and Equivalent Isotropic Displacement Parameters (Å^2×10^3) for **2**. U_{eq} is defined as 1/3 of of the trace of the orthogonalised U_{IJ} tensor.

Atom	*x*	*y*	*z*	U(eq)
C_{12}	2326(3)	3966(2)	4102.0(16)	57.0(11)
C_{13}	2809(2)	5203.6(16)	3087.9(13)	27.9(6)
C_{14}	3036(2)	5847.6(18)	3475.9(13)	35.4(7)
C_{15}	2525(2)	6571.1(18)	3458.8(15)	40.8(8)
C_{16}	1731(2)	6689.4(18)	3066.0(15)	38.6(8)
C_{17}	1140(3)	7412(2)	3064.2(19)	53.1(10)
C_{18}	354(3)	7502(2)	2705(2)	60.9(11)
C_{19}	118(2)	6880(2)	2317(2)	56.2(10)
C_{20}	669(2)	6172.5(18)	2294.6(16)	44.5(7)
C_{21}	1491(2)	6052.9(16)	2673.0(15)	34.4(7)
C_{22}	2063.1(19)	5311.8(15)	2685.0(14)	29.5(6)
C_{23}	1760.9(19)	4626.6(16)	2277.8(15)	31.2(6)
C_{24}	1573(2)	4155.3(19)	1282.3(13)	33.6(7)
C_{25}	445(2)	4282(2)	1189.1(16)	46.5(9)
C_{26}	34(3)	3693(3)	744.1(17)	63.8(11)
C_{27}	585(3)	3783(3)	174.5(18)	68.8(12)
C_{28}	1708(3)	3676(3)	259.4(15)	55.2(9)
C_{29}	2123(2)	4259(2)	711.6(14)	37.8(7)
C_{30}	3880(2)	4730.9(18)	698.0(12)	32.1(7)
C_{31}	4945(2)	4430.7(17)	625.5(13)	29.7(6)
C_{32}	5241(2)	4249.8(19)	45.8(13)	34.6(7)
C_{33}	4624(3)	4425(2)	-435.0(14)	49.4(9)
C_{34}	4936(4)	4255(3)	-977.6(16)	66.3(12)
C_{35}	5872(4)	3900(2)	-1080.3(16)	66.1(12)
C_{36}	6487(3)	3721(2)	-631.1(16)	54.0(9)
C_{37}	6200(2)	3900.4(19)	-57.7(14)	39.7(8)
C_{38}	6832(2)	3748(2)	413.1(15)	45.6(8)
C_{39}	6557(2)	3961(2)	958.7(15)	39.8(8)
C_{40}	5605(2)	4314.1(16)	1071.9(12)	28.5(6)
C_{41}	6492(2)	4600.2(17)	2175.2(12)	29.7(6)
C_{42}	6760(2)	3908.7(19)	2486.0(14)	39.8(8)
C_{43}	7688(3)	3846(2)	2748.7(17)	51.0(9)
C_{44}	8361(2)	4483(2)	2708(2)	57.5(10)
C_{45}	8098(3)	5179(2)	2421.5(17)	53.6(10)
C_{46}	7173(2)	5240.1(19)	2150.7(15)	41.2(8)
C_{47}	4974(2)	5688.2(16)	1708.6(12)	26.7(6)
C_{48}	5445(2)	6171.8(17)	1296.7(13)	33.6(7)
C_{49}	5137(3)	6966.8(19)	1206.5(14)	37.6(7)
C_{50}	4339(3)	7287.2(18)	1512.8(14)	38.3(7)
C_{51}	3863(2)	6811.9(18)	1923.9(14)	35.9(7)
C_{52}	4185(2)	6019.3(16)	2017.1(12)	30.6(6)
C_{54}	4674(2)	2545.1(16)	1858.8(14)	33.0(7)
C_{53}	4149(3)	2509.6(16)	1316.1(14)	40.0(7)
C_{56}	2999(2)	2236.7(17)	2018.2(14)	35.6(7)
C_{55}	3964.0(19)	2367.3(15)	2297.8(14)	29.7(6)

Supplementary Table 15. Fractional Atomic Coordinates (×10^4) and Equivalent Isotropic Displacement Parameters ($Å^2$×10^3) for **2**. U_{eq} is defined as 1/3 of of the trace of the orthogonalised U_{IJ} tensor.

Atom	*x*	*y*	*z*	U(eq)
N_1	1954.9(18)	4727.2(14)	1716.6(11)	33.0(6)
N_2	3211.9(18)	4132.8(16)	762.5(12)	38.9(6)
O_1	1326(2)	4031.0(14)	2470.5(10)	53.3(7)
O_2	2196.1(17)	2033.5(14)	2192.4(12)	54.0(7)
O_3	3131.4(18)	2381.7(13)	1422.1(10)	43.2(6)
O_4	4429(2)	2573.2(14)	824.1(10)	57.0(7)
O_5	3642.3(19)	5450.2(14)	685.3(13)	60.1(7)
P_1	3518.8(5)	4232.9(4)	3120.2(3)	25.96(15)
P_2	5270.8(5)	4604.6(4)	1813.2(3)	25.00(15)
Pd_1	4224.6(2)	3652.6(2)	2280.5(2)	23.60(5)
C_{57}	1110(5)	8159(4)	233(2)	101.3(19)
C_{58}	1443(8)	8172(6)	819(3)	187(5)
C_{59}	1728(6)	7335(6)	942(3)	159(4)
C_{60}	1156(5)	6824(4)	548(3)	109(2)
O_6	804(3)	7340(2)	107.5(13)	85.2(9)

Supplementary Table 16. Anisotropic Displacement Parameters ($Å^2$×10^3) for 2. The Anisotropic displacement factor exponent takes the form: $-2\pi^2[h^2a^{*2}U_{11}+2hka^*b^*U_{12}+\ldots]$.

Atom	U_{11}	U_{22}	U_{33}	U_{23}	U_{13}	U_{12}
C_1	31.3(15)	27.9(14)	28.1(16)	-7.1(12)	1.0(12)	3.8(11)
C_2	39.5(18)	39.4(17)	31.8(18)	1.7(13)	-1.1(15)	-3.6(14)
C_3	40.5(18)	48(2)	48(2)	-4.8(16)	-4.1(16)	-9.0(15)
C_4	52(2)	52(2)	57(2)	-8.2(18)	-22.3(19)	1.3(18)
C_5	63(3)	44.1(19)	42(2)	2.5(15)	-18.5(19)	5.0(18)
C_6	48.9(19)	27.0(16)	34.4(18)	-0.5(13)	-3.6(15)	2.1(14)
C_7	31.2(14)	32.6(14)	35.0(16)	3.2(14)	4.0(12)	4.6(13)
C_8	47(2)	40.3(18)	41(2)	-1.0(15)	7.0(16)	-8.4(15)
C_9	56(2)	44(2)	55(2)	4.5(17)	7.3(19)	-17.1(17)
C_{10}	54(2)	63(2)	58(3)	19(2)	19(2)	-9.0(19)
C_{11}	78(3)	61(3)	62(3)	1(2)	37(2)	-7(2)
C_{12}	76(3)	41.0(19)	54(2)	-4.9(17)	34(2)	-4.1(18)
C_{13}	29.0(14)	25.2(14)	29.6(16)	-1.4(11)	7.2(13)	0.6(11)
C_{14}	36.0(17)	36.3(17)	33.9(17)	-6.8(13)	0.6(14)	3.1(13)
C_{15}	46.0(19)	29.1(16)	47(2)	-11.3(13)	6.7(16)	1.1(13)
C_{16}	33.7(16)	28.8(15)	53(2)	1.2(14)	14.1(15)	2.4(12)
C_{17}	49(2)	31.6(18)	78(3)	-2.9(18)	15(2)	8.0(15)
C_{18}	46(2)	40.7(19)	96(3)	9(2)	12(3)	18.1(15)
C_{19}	33.0(17)	52(2)	84(3)	15(2)	0(2)	10.3(14)
C_{20}	33.3(15)	41.0(17)	59(2)	4.4(16)	1.6(18)	5.3(13)
C_{21}	28.0(13)	31.8(14)	43.3(18)	6.0(14)	10.6(15)	1.6(11)
C_{22}	25.3(13)	27.3(13)	36.0(16)	3.1(13)	6.2(14)	-2.1(10)
C_{23}	21.4(12)	33.7(14)	38.6(16)	1.2(15)	-3.3(14)	0.0(11)
C_{24}	28.9(15)	36.5(17)	35.4(18)	3.5(13)	-4.6(13)	-2.7(13)
C_{25}	27.9(16)	58(2)	53(2)	0.1(17)	-3.6(15)	-4.3(15)
C_{26}	39(2)	88(3)	65(3)	-3(3)	-14.0(18)	-12(2)
C_{27}	61(3)	88(3)	57(3)	-8(2)	-26(2)	-6(2)

Supplementary Table 16. Anisotropic Displacement Parameters (Å^2×10^3) for 2. The Anisotropic displacement factor exponent takes the form: $-2\pi^2[h^2a^{*2}U_{11}+2hka^*b^*U_{12}+\ldots]$.

Atom	U_{11}	U_{22}	U_{33}	U_{23}	U_{13}	U_{12}
C_{28}	61(2)	66(2)	39(2)	-5(2)	-5.4(17)	0(2)
C_{29}	30.3(16)	41.9(18)	41.2(19)	7.9(14)	-4.3(14)	2.7(14)
C_{30}	39.8(17)	35.5(16)	21.0(15)	-4.3(12)	-5.8(12)	1.1(13)
C_{31}	35.6(16)	24.6(14)	28.9(16)	-2.7(12)	3.8(13)	-2.3(12)
C_{32}	43.8(18)	34.6(16)	25.5(16)	-4.6(12)	2.0(14)	-6.6(14)
C_{33}	54(2)	64(2)	30.1(19)	-4.2(16)	-2.6(16)	-3.7(18)
C_{34}	78(3)	95(3)	26(2)	-10(2)	-1(2)	-15(3)
C_{35}	85(3)	82(3)	31(2)	-17.9(18)	19(2)	-19(3)
C_{36}	59(2)	56(2)	47(2)	-18.1(19)	24.2(18)	-10.2(19)
C_{37}	46.1(19)	39.5(18)	33.5(18)	-7.8(13)	11.1(15)	-6.8(14)
C_{38}	36.8(17)	53(2)	47(2)	-7.9(17)	9.4(15)	7.0(16)
C_{39}	33.9(17)	47.4(19)	38.1(19)	-1.7(14)	2.2(14)	6.8(14)
C_{40}	32.9(17)	26.4(14)	26.2(15)	-0.1(11)	5.1(12)	-0.2(12)
C_{41}	31.0(14)	30.2(14)	27.9(17)	-0.5(12)	-1.7(13)	0.7(11)
C_{42}	40.1(17)	39.6(18)	39.8(18)	5.0(13)	-5.7(14)	-4.2(13)
C_{43}	51.4(19)	49(2)	53(2)	3.1(18)	-17.6(19)	9.3(15)
C_{44}	36.6(18)	63(2)	73(3)	-8(2)	-21(2)	4.6(16)
C_{45}	37.2(18)	47(2)	77(3)	-6.5(18)	-10.1(18)	-9.3(15)
C_{46}	33.4(16)	35.3(16)	55(2)	4.2(14)	-4.6(15)	-3.3(13)
C_{47}	31.4(15)	24.2(13)	24.6(15)	1.5(11)	-1.2(12)	-0.7(11)
C_{48}	41.7(17)	31.5(16)	27.8(16)	1.4(11)	5.5(13)	-2.4(12)
C_{49}	49(2)	32.0(16)	32.2(17)	8.2(13)	1.9(15)	-4.8(14)
C_{50}	46(2)	30.1(15)	39.0(18)	3.5(13)	-4.5(16)	3.6(14)
C_{51}	34.1(16)	33.7(16)	39.8(19)	0.1(14)	1.4(14)	2.7(12)
C_{52}	31.0(14)	32.4(14)	28.5(14)	2.2(11)	3.1(14)	-2.7(13)
C_{54}	36.2(16)	23.2(14)	39.6(18)	-4.7(12)	0.4(15)	3.7(12)
C_{53}	62(2)	21.6(14)	36.2(18)	-7.3(12)	-0.2(18)	3.9(15)
C_{56}	42.2(18)	23.3(15)	41.2(19)	-2.7(13)	-6.5(15)	-2.2(13)
C_{55}	37.1(15)	20.5(12)	31.7(15)	-0.3(13)	-2.4(14)	0.7(10)
N_1	27.9(13)	31.6(13)	39.6(16)	-0.1(11)	0.6(11)	-4.2(10)
N_2	31.3(14)	38.7(15)	46.7(17)	10.2(12)	1.6(12)	5.1(11)
O_1	69.9(16)	44.8(14)	45.1(15)	5.0(10)	-5.2(12)	-26.5(12)
O_2	41.2(13)	51.5(14)	69.2(19)	-3.3(13)	-3.9(14)	-15.5(11)
O_3	54.3(15)	36.0(12)	39.3(14)	-3.5(10)	-15.1(11)	-2.9(10)
O_4	97(2)	41.7(13)	31.8(13)	-6.3(10)	6.0(14)	3.4(13)
O_5	47.3(15)	35.1(13)	98(2)	-13.0(13)	-23.2(15)	8.0(11)
P_1	27.5(4)	24.2(3)	26.2(4)	-1.3(3)	1.7(3)	0.9(3)
P_2	27.7(4)	24.1(3)	23.2(4)	1.4(3)	0.7(3)	-0.7(3)
Pd_1	26.42(9)	20.87(9)	23.51(10)	-1.18(9)	0.05(9)	-0.86(8)
C_{57}	121(5)	107(4)	76(4)	-4(3)	-15(3)	-19(4)
C_{58}	287(13)	185(9)	87(6)	5(5)	-69(7)	-114(9)
C_{59}	131(6)	264(11)	83(5)	39(6)	-54(5)	-67(7)
C_{60}	116(5)	135(5)	75(4)	10(4)	-22(4)	20(4)
O_6	102(3)	92(2)	61(2)	-9.6(16)	-24(2)	11(2)

Supplementary Table 17. Bond Lengths for 2.

Atom	Atom	Length/Å	Atom	Atom	Length/Å
C_1	C_2	1.409(4)	C_{31}	C_{40}	1.386(4)
C_1	C_6	1.392(4)	C_{32}	C_{33}	1.430(5)
C_1	P_1	1.858(3)	C_{32}	C_{37}	1.433(5)
C_2	C_3	1.393(4)	C_{33}	C_{34}	1.369(5)
C_3	C_4	1.396(5)	C_{34}	C_{35}	1.409(6)
C_4	C_5	1.373(5)	C_{35}	C_{36}	1.371(6)
C_5	C_6	1.406(5)	C_{36}	C_{37}	1.431(5)
C_7	C_8	1.389(5)	C_{37}	C_{38}	1.416(5)
C_7	C_{12}	1.399(4)	C_{38}	C_{39}	1.378(5)
C_7	P_1	1.850(3)	C_{39}	C_{40}	1.431(4)
C_8	C_9	1.397(5)	C_{40}	P_2	1.859(3)
C_9	C_{10}	1.386(6)	C_{41}	C_{42}	1.402(4)
C_{10}	C_{11}	1.380(6)	C_{41}	C_{46}	1.400(4)
C_{11}	C_{12}	1.390(5)	C_{41}	P_2	1.849(3)
C_{13}	C_{14}	1.433(4)	C_{42}	C_{43}	1.395(5)
C_{13}	C_{22}	1.390(4)	C_{43}	C_{44}	1.392(5)
C_{13}	P_1	1.867(3)	C_{44}	C_{45}	1.377(5)
C_{14}	C_{15}	1.379(4)	C_{45}	C_{46}	1.400(5)
C_{15}	C_{16}	1.424(5)	C_{47}	C_{48}	1.405(4)
C_{16}	C_{17}	1.433(4)	C_{47}	C_{52}	1.396(4)
C_{16}	C_{21}	1.435(4)	C_{47}	P_2	1.850(3)
C_{17}	C_{18}	1.361(6)	C_{48}	C_{49}	1.393(4)
C_{18}	C_{19}	1.408(6)	C_{49}	C_{50}	1.396(5)
C_{19}	C_{20}	1.384(4)	C_{50}	C_{51}	1.399(4)
C_{20}	C_{21}	1.432(5)	C_{51}	C_{52}	1.396(4)
C_{21}	C_{22}	1.446(4)	C_{54}	C_{53}	1.457(5)
C_{22}	C_{23}	1.536(4)	C_{54}	C_{55}	1.435(4)
C_{23}	N_1	1.352(4)	C_{54}	Pd_1	2.166(3)
C_{23}	O_1	1.230(4)	C_{53}	O_3	1.406(5)
C_{24}	C_{25}	1.547(4)	C_{53}	O_4	1.219(4)
C_{24}	C_{29}	1.539(5)	C_{56}	C_{55}	1.469(4)
C_{24}	N_1	1.482(4)	C_{56}	O_2	1.202(4)
C_{25}	C_{26}	1.530(5)	C_{56}	O_3	1.430(4)
C_{26}	C_{27}	1.535(6)	C_{55}	Pd_1	2.152(3)
C_{27}	C_{28}	1.534(6)	P_1	Pd_1	2.3877(15)
C_{28}	C_{29}	1.538(5)	P_2	Pd_1	2.3776(12)
C_{29}	N_2	1.484(4)	C_{57}	C_{58}	1.447(8)
C_{30}	C_{31}	1.525(4)	C_{57}	O_6	1.444(6)
C_{30}	N_2	1.343(4)	C_{58}	C_{59}	1.463(12)
C_{30}	O_5	1.231(4)	C_{59}	C_{60}	1.470(9)
C_{31}	C_{32}	1.449(4)	C_{60}	O_6	1.420(6)

Supplementary Table 18. Bond Angles for 2.

Atom	Atom	Atom	Angle/˚	Atom	Atom	Atom	Angle/˚
C_2	C_1	P_1	119.0(2)	C_{38}	C_{37}	C_{32}	118.6(3)
C_6	C_1	C_2	117.8(3)	C_{38}	C_{37}	C_{36}	122.4(3)
C_6	C_1	P_1	123.0(2)	C_{39}	C_{38}	C_{37}	121.2(3)
C_3	C_2	C_1	121.1(3)	C_{38}	C_{39}	C_{40}	121.1(3)
C_2	C_3	C_4	120.1(3)	C_{31}	C_{40}	C_{39}	119.2(3)
C_5	C_4	C_3	119.3(3)	C_{31}	C_{40}	P_2	121.1(2)
C_4	C_5	C_6	121.0(3)	C_{39}	C_{40}	P_2	119.7(2)
C_1	C_6	C_5	120.6(3)	C_{42}	C_{41}	P_2	118.1(2)
C_8	C_7	C_{12}	117.4(3)	C_{46}	C_{41}	C_{42}	117.9(3)
C_8	C_7	P_1	121.2(2)	C_{46}	C_{41}	P_2	124.0(2)
C_{12}	C_7	P_1	121.3(3)	C_{43}	C_{42}	C_{41}	121.4(3)
C_7	C_8	C_9	121.3(3)	C_{44}	C_{43}	C_{42}	119.6(3)
C_{10}	C_9	C_8	119.8(4)	C_{45}	C_{44}	C_{43}	119.9(3)
C_{11}	C_{10}	C_9	120.1(3)	C_{44}	C_{45}	C_{46}	120.6(3)
C_{10}	C_{11}	C_{12}	119.5(4)	C_{41}	C_{46}	C_{45}	120.5(3)
C_{11}	C_{12}	C_7	121.9(4)	C_{48}	C_{47}	P_2	123.0(2)
C_{14}	C_{13}	P_1	120.2(2)	C_{52}	C_{47}	C_{48}	118.5(3)
C_{22}	C_{13}	C_{14}	119.3(3)	C_{52}	C_{47}	P_2	118.3(2)
C_{22}	C_{13}	P_1	120.5(2)	C_{49}	C_{48}	C_{47}	120.4(3)
C_{15}	C_{14}	C_{13}	121.3(3)	C_{48}	C_{49}	C_{50}	120.5(3)
C_{14}	C_{15}	C_{16}	120.7(3)	C_{49}	C_{50}	C_{51}	119.6(3)
C_{15}	C_{16}	C_{17}	122.1(3)	C_{52}	C_{51}	C_{50}	119.5(3)
C_{15}	C_{16}	C_{21}	118.9(3)	C_{47}	C_{52}	C_{51}	121.5(3)
C_{17}	C_{16}	C_{21}	118.9(3)	C_{53}	C_{54}	Pd_1	107.33(19)
C_{18}	C_{17}	C_{16}	121.6(4)	C_{55}	C_{54}	C_{53}	107.2(3)
C_{17}	C_{18}	C_{19}	119.7(3)	C_{55}	C_{54}	Pd_1	70.07(15)
C_{20}	C_{19}	C_{18}	121.3(4)	O_3	C_{53}	C_{54}	108.8(3)
C_{19}	C_{20}	C_{21}	120.4(3)	O_4	C_{53}	C_{54}	132.4(4)
C_{16}	C_{21}	C_{22}	119.2(3)	O_4	C_{53}	O_3	118.7(3)
C_{20}	C_{21}	C_{16}	118.1(3)	O_2	C_{56}	C_{55}	133.0(3)
C_{20}	C_{21}	C_{22}	122.7(3)	O_2	C_{56}	O_3	119.4(3)
C_{13}	C_{22}	C_{21}	120.4(3)	O_3	C_{56}	C_{55}	107.6(3)
C_{13}	C_{22}	C_{23}	121.3(2)	C_{54}	C_{55}	C_{56}	107.3(3)
C_{21}	C_{22}	C_{23}	118.1(3)	C_{54}	C_{55}	Pd_1	71.11(15)
N_1	C_{23}	C_{22}	117.7(2)	C_{56}	C_{55}	Pd_1	106.27(18)
O_1	C_{23}	C_{22}	119.1(3)	C_{23}	N_1	C_{24}	121.6(2)
O_1	C_{23}	N_1	123.2(3)	C_{30}	N_2	C_{29}	123.1(3)
C_{29}	C_{24}	C_{25}	109.4(3)	C_{53}	O_3	C_{56}	108.6(2)
N_1	C_{24}	C_{25}	110.5(3)	C_1	P_1	C_{13}	102.84(13)
N_1	C_{24}	C_{29}	111.1(2)	C_1	P_1	Pd_1	106.03(10)
C_{26}	C_{25}	C_{24}	111.3(3)	C_7	P_1	C_1	102.40(14)
C_{25}	C_{26}	C_{27}	111.0(3)	C_7	P_1	C_{13}	100.49(13)
C_{28}	C_{27}	C_{26}	110.5(3)	C_7	P_1	Pd_1	121.36(11)
C_{27}	C_{28}	C_{29}	112.0(3)	C_{13}	P_1	Pd_1	120.95(10)
C_{28}	C_{29}	C_{24}	110.9(3)	C_{40}	P_2	Pd_1	113.82(10)
N_2	C_{29}	C_{24}	112.8(3)	C_{41}	P_2	C_{40}	102.37(14)

Supplementary Table 18. Bond Angles for 2.

Atom	Atom	Atom	Angle/˚	Atom	Atom	Atom	Angle/˚
N_2	C_{29}	C_{28}	109.0(3)	C_{41}	P_2	C_{47}	104.84(13)
N_2	C_{30}	C_{31}	113.7(3)	C_{41}	P_2	Pd_1	108.11(10)
O_5	C_{30}	C_{31}	123.7(3)	C_{47}	P_2	C_{40}	100.23(13)
O_5	C_{30}	N_2	122.6(3)	C_{47}	P_2	Pd_1	124.99(10)
C_{32}	C_{31}	C_{30}	115.5(3)	C_{54}	Pd_1	P_1	145.79(9)
C_{40}	C_{31}	C_{30}	124.2(3)	C_{54}	Pd_1	P_2	100.52(10)
C_{40}	C_{31}	C_{32}	120.3(3)	C_{55}	Pd_1	C_{54}	38.82(11)
C_{33}	C_{32}	C_{31}	122.7(3)	C_{55}	Pd_1	P_1	108.41(9)
C_{33}	C_{32}	C_{37}	118.0(3)	C_{55}	Pd_1	P_2	139.23(8)
C_{37}	C_{32}	C_{31}	119.3(3)	P_2	Pd_1	P_1	110.50(5)
C_{34}	C_{33}	C_{32}	120.9(4)	O_6	C_{57}	C_{58}	107.2(5)
C_{33}	C_{34}	C_{35}	121.2(4)	C_{57}	C_{58}	C_{59}	104.7(7)
C_{36}	C_{35}	C_{34}	119.7(3)	C_{58}	C_{59}	C_{60}	106.4(6)
C_{35}	C_{36}	C_{37}	121.1(4)	O_6	C_{60}	C_{59}	106.6(6)
C_{36}	C_{37}	C_{32}	119.0(3)	C_{60}	O_6	C_{57}	108.6(4)

Supplementary Table 19. Torsion Angles for 2.

A	B	C	D	Angle/˚	A	B	C	D	Angle/˚
C_1	C_2	C_3	C_4	0.7(5)	C_{32}	C_{37}	C_{38}	C_{39}	-2.7(5)
C_2	C_1	C_6	C_5	-0.3(4)	C_{33}	C_{32}	C_{37}	C_{36}	-1.7(4)
C_2	C_1	P_1	C_7	-169.7(2)	C_{33}	C_{32}	C_{37}	C_{38}	178.0(3)
C_2	C_1	P_1	C_{13}	-65.8(3)	C_{33}	C_{34}	C_{35}	C_{36}	-0.1(7)
C_2	C_1	P_1	Pd_1	62.1(2)	C_{34}	C_{35}	C_{36}	C_{37}	-0.9(6)
C_2	C_3	C_4	C_5	-0.1(6)	C_{35}	C_{36}	C_{37}	C_{32}	1.9(5)
C_3	C_4	C_5	C_6	-0.6(6)	C_{35}	C_{36}	C_{37}	C_{38}	-177.8(4)
C_4	C_5	C_6	C_1	0.9(5)	C_{36}	C_{37}	C_{38}	C_{39}	177.0(3)
C_6	C_1	C_2	C_3	-0.5(5)	C_{37}	C_{32}	C_{33}	C_{34}	0.7(5)
C_6	C_1	P_1	C_7	15.2(3)	C_{37}	C_{38}	C_{39}	C_{40}	2.5(5)
C_6	C_1	P_1	C_{13}	119.2(3)	C_{38}	C_{39}	C_{40}	C_{31}	1.1(5)
C_6	C_1	P_1	Pd_1	-113.0(2)	C_{38}	C_{39}	C_{40}	P_2	-179.2(3)
C_7	C_8	C_9	C_{10}	0.3(6)	C_{39}	C_{40}	P_2	C_{41}	15.9(3)
C_8	C_7	C_{12}	C_{11}	-0.4(6)	C_{39}	C_{40}	P_2	C_{47}	123.7(2)
C_8	C_7	P_1	C_1	-117.1(3)	C_{39}	C_{40}	P_2	Pd_1	-100.5(2)
C_8	C_7	P_1	C_{13}	137.1(3)	C_{40}	C_{31}	C_{32}	C_{33}	-174.4(3)
C_8	C_7	P_1	Pd_1	0.6(3)	C_{40}	C_{31}	C_{32}	C_{37}	4.0(4)
C_8	C_9	C_{10}	C_{11}	-0.3(6)	C_{41}	C_{42}	C_{43}	C_{44}	0.7(6)
C_9	C_{10}	C_{11}	C_{12}	0.0(7)	C_{42}	C_{41}	C_{46}	C_{45}	1.1(5)
C_{10}	C_{11}	C_{12}	C_7	0.4(7)	C_{42}	C_{41}	P_2	C_{40}	-97.6(3)
C_{12}	C_7	C_8	C_9	0.1(5)	C_{42}	C_{41}	P_2	C_{47}	158.2(2)
C_{12}	C_7	P_1	C_1	59.8(3)	C_{42}	C_{41}	P_2	Pd_1	22.9(3)
C_{12}	C_7	P_1	C_{13}	-46.0(3)	C_{42}	C_{43}	C_{44}	C_{45}	1.5(6)
C_{12}	C_7	P_1	Pd_1	177.5(3)	C_{43}	C_{44}	C_{45}	C_{46}	-2.4(6)
C_{13}	C_{14}	C_{15}	C_{16}	2.2(5)	C_{44}	C_{45}	C_{46}	C_{41}	1.1(6)
C_{13}	C_{22}	C_{23}	N_1	-113.2(3)	C_{46}	C_{41}	C_{42}	C_{43}	-2.0(5)

Supplementary Table 19. Torsion Angles for 2.

A	B	C	D	Angle/˚	A	B	C	D	Angle/˚
C_{13}	C_{22}	C_{23}	O_1	69.4(4)	C_{46}	C_{41}	P_2	C_{40}	81.4(3)
C_{14}	C_{13}	C_{22}	C_{21}	-2.7(4)	C_{46}	C_{41}	P_2	C_{47}	-22.9(3)
C_{14}	C_{13}	C_{22}	C_{23}	-177.2(3)	C_{46}	C_{41}	P_2	Pd_1	-158.2(2)
C_{14}	C_{13}	P_1	C_1	-12.5(3)	C_{47}	C_{48}	C_{49}	C_{50}	-1.8(5)
C_{14}	C_{13}	P_1	C_7	93.0(3)	C_{48}	C_{47}	C_{52}	C_{51}	0.0(4)
C_{14}	C_{13}	P_1	Pd_1	-130.3(2)	C_{48}	C_{47}	P_2	C_{40}	-31.7(3)
C_{14}	C_{15}	C_{16}	C_{17}	175.5(3)	C_{48}	C_{47}	P_2	C_{41}	74.2(3)
C_{14}	C_{15}	C_{16}	C_{21}	-2.2(5)	C_{48}	C_{47}	P_2	Pd_1	-160.5(2)
C_{15}	C_{16}	C_{17}	C_{18}	-176.9(3)	C_{48}	C_{49}	C_{50}	C_{51}	1.7(5)
C_{15}	C_{16}	C_{21}	C_{20}	177.9(3)	C_{49}	C_{50}	C_{51}	C_{52}	-0.8(5)
C_{15}	C_{16}	C_{21}	C_{22}	-0.2(4)	C_{50}	C_{51}	C_{52}	C_{47}	0.0(5)
C_{16}	C_{17}	C_{18}	C_{19}	-1.1(6)	C_{52}	C_{47}	C_{48}	C_{49}	0.9(4)
C_{16}	C_{21}	C_{22}	C_{13}	2.7(4)	C_{52}	C_{47}	P_2	C_{40}	142.3(2)
C_{16}	C_{21}	C_{22}	C_{23}	177.3(3)	C_{52}	C_{47}	P_2	C_{41}	-111.8(2)
C_{17}	C_{16}	C_{21}	C_{20}	0.1(4)	C_{52}	C_{47}	P_2	Pd_1	13.5(3)
C_{17}	C_{16}	C_{21}	C_{22}	-178.0(3)	C_{54}	C_{53}	O_3	C_{56}	-6.8(3)
C_{17}	C_{18}	C_{19}	C_{20}	0.3(6)	C_{53}	C_{54}	C_{55}	C_{56}	-0.9(3)
C_{18}	C_{19}	C_{20}	C_{21}	0.7(6)	C_{53}	C_{54}	C_{55}	Pd_1	-102.7(2)
C_{19}	C_{20}	C_{21}	C_{16}	-0.9(5)	C_{55}	C_{54}	C_{53}	O_3	4.8(3)
C_{19}	C_{20}	C_{21}	C_{22}	177.2(3)	C_{55}	C_{54}	C_{53}	O_4	-175.7(3)
C_{20}	C_{21}	C_{22}	C_{13}	-175.4(3)	C_{55}	C_{56}	O_3	C_{53}	6.2(3)
C_{20}	C_{21}	C_{22}	C_{23}	-0.7(4)	N_1	C_{24}	C_{25}	C_{26}	-179.6(3)
C_{21}	C_{16}	C_{17}	C_{18}	0.8(5)	N_1	C_{24}	C_{29}	C_{28}	-178.9(3)
C_{21}	C_{22}	C_{23}	N_1	72.2(3)	N_1	C_{24}	C_{29}	N_2	58.6(3)
C_{21}	C_{22}	C_{23}	O_1	-105.2(3)	N_2	C_{30}	C_{31}	C_{32}	87.6(3)
C_{22}	C_{13}	C_{14}	C_{15}	0.3(4)	N_2	C_{30}	C_{31}	C_{40}	-91.1(3)
C_{22}	C_{13}	P_1	C_1	167.0(2)	O_1	C_{23}	N_1	C_{24}	5.7(4)
C_{22}	C_{13}	P_1	C_7	-87.6(3)	O_2	C_{56}	C_{55}	C_{54}	175.5(3)
C_{22}	C_{13}	P_1	Pd_1	49.2(3)	O_2	C_{56}	C_{55}	Pd_1	-109.7(4)
C_{22}	C_{23}	N_1	C_{24}	-171.6(2)	O_2	C_{56}	O_3	C_{53}	-172.7(3)
C_{24}	C_{25}	C_{26}	C_{27}	-57.5(4)	O_3	C_{56}	C_{55}	C_{54}	-3.2(3)
C_{24}	C_{29}	N_2	C_{30}	-112.0(3)	O_3	C_{56}	C_{55}	Pd_1	71.6(2)
C_{25}	C_{24}	C_{29}	C_{28}	-56.5(4)	O_4	C_{53}	O_3	C_{56}	173.6(3)
C_{25}	C_{24}	C_{29}	N_2	-179.0(3)	O_5	C_{30}	C_{31}	C_{32}	-90.7(4)
C_{25}	C_{24}	N_1	C_{23}	74.1(3)	O_5	C_{30}	C_{31}	C_{40}	90.6(4)
C_{25}	C_{26}	C_{27}	C_{28}	55.5(5)	O_5	C_{30}	N_2	C_{29}	12.3(5)
C_{26}	C_{27}	C_{28}	C_{29}	-55.1(5)	P_1	C_1	C_2	C_3	-175.8(2)
C_{27}	C_{28}	C_{29}	C_{24}	56.3(4)	P_1	C_1	C_6	C_5	174.8(2)
C_{27}	C_{28}	C_{29}	N_2	-178.9(3)	P_1	C_7	C_8	C_9	177.1(3)
C_{28}	C_{29}	N_2	C_{30}	124.4(3)	P_1	C_7	C_{12}	C_{11}	-177.4(3)
C_{29}	C_{24}	C_{25}	C_{26}	57.6(4)	P_1	C_{13}	C_{14}	C_{15}	179.7(2)
C_{29}	C_{24}	N_1	C_{23}	-164.2(3)	P_1	C_{13}	C_{22}	C_{21}	177.9(2)
C_{30}	C_{31}	C_{32}	C_{33}	6.9(4)	P_1	C_{13}	C_{22}	C_{23}	3.4(4)
C_{30}	C_{31}	C_{32}	C_{37}	-174.8(3)	P_2	C_{41}	C_{42}	C_{43}	177.0(3)
C_{30}	C_{31}	C_{40}	C_{39}	174.4(3)	P_2	C_{41}	C_{46}	C_{45}	-177.8(3)
C_{30}	C_{31}	C_{40}	P_2	-5.3(4)	P_2	C_{47}	C_{48}	C_{49}	174.9(2)

Supplementary Table 19. Torsion Angles for 2.

A	B	C	D	Angle/°	A	B	C	D	Angle/°
C_{31}	C_{30}	N_2	C_{29}	-166.0(3)	P_2	C_{47}	C_{52}	C_{51}	-174.3(2)
C_{31}	C_{32}	C_{33}	C_{34}	179.1(3)	Pd_1	C_{54}	C_{53}	O_3	-69.1(2)
C_{31}	C_{32}	C_{37}	C_{36}	179.8(3)	Pd_1	C_{54}	C_{53}	O_4	110.4(3)
C_{31}	C_{32}	C_{37}	C_{38}	-0.4(4)	Pd_1	C_{54}	C_{55}	C_{56}	101.75(19)
C_{31}	C_{40}	P_2	C_{41}	-164.4(2)	C_{57}	C_{58}	C_{59}	C_{60}	-25.1(10)
C_{31}	C_{40}	P_2	C_{47}	-56.6(3)	C_{58}	C_{57}	O_6	C_{60}	-13.6(8)
C_{31}	C_{40}	P_2	Pd_1	79.2(2)	C_{58}	C_{59}	C_{60}	O_6	17.1(9)
C_{32}	C_{31}	C_{40}	C_{39}	-4.3(4)	C_{59}	C_{60}	O_6	C_{57}	-2.3(7)
C_{32}	C_{31}	C_{40}	P_2	176.0(2)	O_6	C_{57}	C_{58}	C_{59}	23.8(10)
C_{32}	C_{33}	C_{34}	C_{35}	0.2(6)					

Supplementary Table 20. Hydrogen Atom Coordinates (Å×10^4) and Isotropic Displacement Parameters (Å^2×10^3) for 2.

Atom	*x*	*y*	*z*	U(eq)
H_2	5188.23	5330.44	3049.29	44
H_3	6600.14	5624.73	3590.02	55
H_4	6878.26	4951.68	4458.1	64
H_5	5737.65	3996.81	4774.2	60
H_6	4297.61	3715.33	4246.75	44
H_8	2745.48	2585.96	3147.25	51
H_9	1785.25	1812.6	3775.73	62
H_{10}	1153.87	2399.34	4606.5	70
H_{11}	1493.84	3752.98	4814.12	80
H_{12}	2464.56	4515.59	4190.57	68
H_{14}	3547.42	5774.67	3750.27	42
H_{15}	2704.48	6995.44	3711.92	49
H_{17}	1301.95	7837.53	3320.07	64
H_{18}	-34.25	7982.35	2715.41	73
H_{19}	-429.57	6947.81	2065.6	67
H_{20}	499.6	5763.9	2026.63	53
H_{24}	1682.9	3590.35	1421.94	40
H_{25A}	89.94	4201.79	1554.43	56
H_{25B}	323.45	4845.1	1061.44	56
H_{26A}	-683.37	3798.79	686.05	77
H_{26B}	108.4	3131	884.8	77
H_{27A}	337.98	3371.76	-98.01	83
H_{27B}	449.9	4325.15	12.15	83
H_{28A}	2052.82	3772.24	-107.29	66
H_{28B}	1846.1	3111.36	377.54	66
H_{29}	2008.55	4825.27	575.05	45
H_{33}	3988.34	4662.13	-377.29	59
H_{34}	4514.04	4378.62	-1290.29	80
H_{35}	6076.78	3785.1	-1459.31	79
H_{36}	7113.25	3474.64	-701.91	65

Supplementary Table 20. Hydrogen Atom Coordinates (Å×10[4]) and Isotropic Displacement Parameters (Å[2]×10[3]) for 2.

Atom	*x*	*y*	*z*	U(eq)
H_{38}	7457.28	3495.25	351.73	55
H_{39}	7006.19	3871.46	1264.69	48
H_{42}	6300.9	3474.22	2518.44	48
H_{43}	7859.74	3369.77	2953.83	61
H_{44}	8999.65	4439.3	2878.68	69
H_{45}	8549.38	5621.12	2407.15	64
H_{46}	7006.76	5719.84	1948.69	49
H_{48}	5977.31	5955.13	1078.24	40
H_{49}	5473.34	7293.4	934.39	45
H_{50}	4120.54	7825	1442.55	46
H_{51}	3324.4	7026.7	2138.11	43
H_{52}	3860.13	5698.21	2296.64	37
H_{54}	5374.84	2346.17	1889.88	40
H_{55}	4159.76	2038.24	2637.48	36
H_{1}	2317.05	5142.73	1606.82	40
H_{2A}	3430.85	3642.53	838.58	47
H_{57A}	546.35	8536.91	180.18	122
H_{57B}	1657.2	8324.84	-24.53	122
H_{58A}	901.77	8350.88	1075.78	224
H_{58B}	2018.38	8540.31	865.68	224
H_{59A}	1567.92	7195.57	1341.84	191
H_{59B}	2450.66	7258.2	881.26	191
H_{60A}	1584.08	6392.76	386.77	131
H_{60B}	590.6	6566.28	748.37	131

Refinement model description

Number of restraints - 0, number of constraints - unknown.

Details:

1. Twinned data refinement

Scales: 1.021(16)

-0.021(16)

2. Fixed Uiso

At 1.2 times of:

All C(H) groups, All C(H,H) groups, All N(H) groups

3.a Ternary CH refined with riding coordinates:

C24(H24), C29(H29), C54(H54), C55(H55)

3.b Secondary CH2 refined with riding coordinates:

C25(H25A,H25B), C26(H26A,H26B), C27(H27A,H27B), C28(H28A,H28B), C57(H57A,H57B), C58(H58A,H58B), C59(H59A,H59B), C60(H60A,H60B)

3.c Aromatic/amide H refined with riding coordinates:

C2(H2), C3(H3), C4(H4), C5(H5), C6(H6), C8(H8), C9(H9), C10(H10), C11(H11), C12(H12), C14(H14), C15(H15), C17(H17), C18(H18), C19(H19), C20(H20), C33(H33), C34(H34), C35(H35), C36(H36), C38(H38), C39(H39), C42(H42), C43(H43), C44(H44), C45(H45), C46(H46), C48(H48), C49(H49), C50(H50), C51(H51), C52(H52), N1(H1), N2(H2A)

(*S,S*)-^{Ph}ANDEN -Pd-MAH (4)

Single crystals of $C_{66}H_{58}N_2O_7P_2Pd$ (**3**) were selected using a MitEGen loop and paratone oil. A suitable crystal was selected and run on a Bruker APEX-II CCD diffractometer. The crystal was kept at 100 K during data collection. Using Olex2[22], the structure was solved with the olex2.solve[23] structure solution program using Charge Flipping and refined with the XL[24] refinement package using Least Squares minimisation. SQUEEZE[25] was used on four molecules of tetrahydrofuran, used as solvent, due to it being highly disordered.

Crystal structure determination of (4)

Crystal Data for $C_{66}H_{58}N_2O_7P_2Pd$ (M =1159.48 g/mol): orthorhombic, space group $P2_12_12_1$ (no. 19), a = 14.552(3) Å, b = 16.681(3) Å, c = 26.014(5) Å, V = 6314(2) Å^3, Z = 57, T = 100 K, μ(MoKα) = 0.395 mm^{-1}, $Dcalc$ = 1.220 g/cm^3, 78493 reflections measured (2.9° ≤ 2Θ ≤ 52.604°), 12630 unique (R_{int} = 0.0988, R_{sigma} = 0.0715) which were used in all calculations. The final R_1 was 0.0546 (I > 2σ(I)) and wR_2 was 0.1439 (all data).

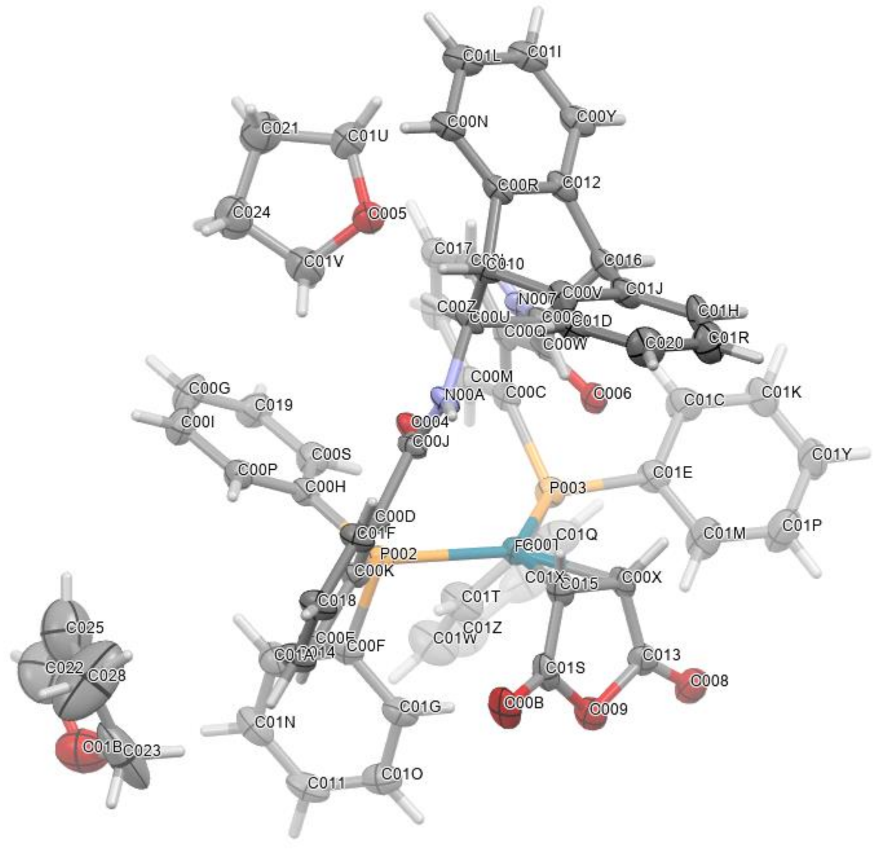

Supplementary Figure 125: Solid state molecular structure of **4** with thermal ellipsoids plotted at 50% probability. Hydrogen atoms are shown as capped sticks. Asymmetric unit contains two molecules of THF solvent. Image generated using Mercury 2021.3.0. CCDC deposition number 2258959.

Supplementary Table 21. Crystal data and structure refinement for 4.

Identification code	**4**
Empirical formula	$C_{66}H_{58}N_2O_7P_2Pd$
Formula weight	1159.48
Temperature/K	100
Crystal system	orthorhombic
Space group	$P2_12_12_1$
a/Å	14.552(3)
b/Å	16.681(3)
c/Å	26.014(5)
α/°	90
β/°	90
γ/°	90
Volume/Å^3	6314(2)
Z	57
ρ_{calc}g/cm^3	1.220
μ/mm^{-1}	0.395
F(000)	2400.0
Crystal size/mm^3	0.0176 × 0.0132 × 0.0078
Radiation	MoKα (λ = 0.71073)
2Θ range for data collection/°	2.9 to 52.604
Index ranges	-18 ≤ h ≤ 18, -20 ≤ k ≤ 20, -31 ≤ l ≤ 32
Reflections collected	78493
Independent reflections	12630 [R_{int} = 0.0988, R_{sigma} = 0.0715]
Data/restraints/parameters	12630/0/703
Goodness-of-fit on F^2	1.027
Final R indexes [I>=2σ (I)]	R_1 = 0.0546, wR_2 = 0.1325
Final R indexes [all data]	R_1 = 0.0740, wR_2 = 0.1439
Largest diff. peak/hole / e Å^{-3}	1.38/-1.93
Flack parameter	-0.017(13)

Supplementary Table 22. Fractional Atomic Coordinates (×10^4) and Equivalent Isotropic Displacement Parameters (Å^2×10^3) for 4. U_{eq} is defined as 1/3 of of the trace of the orthogonalised U_{IJ} tensor.

**Atom**	***x***	***y***	***z***	**U(eq)**
Pd_{01}	5634.5(3)	4044.2(3)	3670.5(2)	22.38(14)
P_{002}	6285.4(11)	4876.0(10)	4294.9(7)	22.1(4)
P_{003}	5508.8(12)	4655.0(11)	2861.5(6)	26.5(4)
O_{004}	7739(3)	4118(3)	3961.5(16)	25.6(10)
O_{005}	9815(3)	4677(3)	3208(2)	37.3(12)
O_{006}	6758(3)	3442(3)	2812.4(17)	26.4(10)
N_{007}	8273(4)	3581(3)	2955(2)	23.9(12)
O_{008}	3403(4)	2913(4)	3425(2)	43.4(14)
O_{009}	3970(3)	3060(3)	4222.5(19)	34.6(12)
N_{00A}	8391(4)	2966(3)	4207(2)	24.2(12)
O_{00B}	4878(4)	2950(3)	4915.3(19)	43.9(14)

Supplementary Table 22. Fractional Atomic Coordinates (×10[4]) and Equivalent Isotropic Displacement Parameters (Å[2]×10[3]) for 4. U_{eq} is defined as 1/3 of of the trace of the orthogonalised U_{IJ} tensor.

Atom	*x*	*y*	*z*	U(eq)
C00C	6566(5)	5103(4)	2599(2)	24.5(14)
C00D	7405(4)	3716(4)	4804(2)	22.9(14)
C00E	6343(5)	4432(4)	5337(3)	25.6(14)
C00F	5341(4)	5472(4)	4580(2)	25.4(15)
C00G	8399(5)	6916(5)	4062(3)	42(2)
C00H	7147(4)	5662(4)	4204(3)	24.4(14)
C00I	8445(5)	6443(4)	4495(3)	36.8(18)
C00J	7852(4)	3608(4)	4296(2)	23.4(14)
C00K	6715(4)	4303(4)	4852(2)	21.8(13)
C00L	8194(5)	5060(4)	2403(2)	26.9(15)
C00M	6565(4)	5882(4)	2408(2)	26.1(14)
C00N	11120(5)	2781(4)	3254(3)	29.2(16)
C00O	8337(5)	2837(4)	3242(3)	25.4(15)
C00P	7828(4)	5818(4)	4568(3)	29.9(16)
C00Q	7418(4)	4692(4)	2599(2)	23.2(14)
C00R	10240(5)	2477(4)	3271(3)	26.4(15)
C00S	7108(5)	6141(4)	3774(3)	30.8(17)
C00T	4702(5)	5490(5)	2842(3)	34.6(18)
C00U	8933(4)	2955(4)	3734(2)	23.1(13)
C00V	9214(4)	1487(4)	3702(3)	26.9(14)
C00W	7450(4)	3848(4)	2799(2)	21.2(14)
C00X	5046(5)	2900(4)	3572(2)	28.6(16)
C00Y	10102(5)	2590(4)	2347(3)	32.6(17)
C00Z	7335(5)	6244(4)	2212(3)	29.8(16)
C010	9683(4)	2301(4)	3751(2)	26.8(15)
C011	3838(6)	6370(5)	4920(3)	39.1(19)
C012	9730(5)	2387(4)	2815(3)	28.8(15)
C013	4084(5)	2944(4)	3682(3)	33.4(16)
C014	6625(5)	4004(5)	5759(3)	32.8(16)
C015	5530(5)	2924(4)	4054(3)	30.4(15)
C016	8743(5)	2150(4)	2912(3)	27.9(15)
C017	8157(5)	5844(4)	2215(3)	29.2(15)
C018	7297(5)	3418(4)	5710(3)	31.9(16)
C019	7728(5)	6765(4)	3703(3)	38.6(17)
C01A	5456(5)	6243(4)	4761(3)	36.7(18)
O01B	5790(5)	7130(5)	6105(3)	73(2)
C01C	5519(5)	4016(5)	1845(3)	41.2(18)
C01D	9224(5)	869(4)	4058(3)	34.0(16)
C01E	5078(4)	4054(5)	2327(3)	31.1(15)
C01F	7678(5)	3273(4)	5236(3)	28.3(15)
C01G	4458(5)	5153(4)	4571(3)	32.1(16)
C01H	8257(6)	698(4)	3141(3)	35.9(18)
C01I	10998(5)	2888(5)	2329(3)	36.6(18)
C01J	8711(5)	1411(4)	3240(3)	30.8(16)
C01K	5152(6)	3562(6)	1459(3)	50(2)

Supplementary Table 22. Fractional Atomic Coordinates (×10[4]) and Equivalent Isotropic Displacement Parameters (Å[2]×10[3]) for 4. U_{eq} is defined as 1/3 of of the trace of the orthogonalised U_{IJ} tensor.

Atom	*x*	*y*	*z*	U(eq)
C01L	11494(5)	2992(5)	2782(3)	37.4(18)
C01M	4270(6)	3642(6)	2391(3)	53(2)
C01N	4718(6)	6687(5)	4929(3)	45(2)
C01O	3711(5)	5592(5)	4732(3)	37.7(19)
C01P	3898(6)	3191(6)	1993(4)	60(3)
C01Q	4307(5)	5798(5)	2385(3)	46(2)
C01R	8283(6)	75(5)	3497(3)	43(2)
C01S	4832(6)	2976(4)	4452(3)	32.9(17)
C01T	4503(5)	5895(5)	3295(3)	41.7(18)
C01U	10553(6)	4994(5)	2908(4)	52(2)
C01V	9549(7)	5310(6)	3544(4)	63(3)
C01W	3955(7)	6573(6)	3309(4)	59(3)
C01X	3754(6)	6494(6)	2415(4)	53(3)
C01Y	4346(7)	3142(5)	1527(3)	52(2)
C01Z	3592(7)	6896(6)	2873(4)	62(3)
C020	8767(6)	166(5)	3949(3)	40.6(19)
C021	11105(7)	5519(7)	3269(4)	65(3)
C022	6674(10)	7483(8)	5911(6)	97(4)
C023	5874(12)	6309(7)	6244(4)	103(5)
C024	10404(6)	5750(6)	3675(4)	58(2)
C025	7266(11)	6837(8)	5937(6)	100(4)
C028	6966(10)	6230(9)	6274(7)	135(7)

Supplementary Table 23. Anisotropic Displacement Parameters (Å[2]×10[3]) for 4. The Anisotropic displacement factor exponent takes the form: $-2\pi^2[h^2a^{*2}U_{11}+2hka^*b^*U_{12}+\ldots]$.

Atom	U_{11}	U_{22}	U_{33}	U_{23}	U_{13}	U_{12}
Pd01	27.9(2)	25.7(2)	13.5(2)	3.2(2)	-1.6(2)	-1.8(2)
P002	26.1(8)	23.7(8)	16.4(8)	2.2(7)	-0.5(7)	1.1(7)
P003	28.9(9)	32.3(9)	18.4(9)	7.0(7)	-3.9(7)	-2.3(8)
O004	36(2)	25(2)	15(2)	2(2)	2.0(19)	6(2)
O005	41(3)	37(3)	34(3)	-3(2)	6(2)	-8(2)
O006	32(2)	30(2)	17(2)	0(2)	-3(2)	-4(2)
N007	30(3)	28(3)	14(3)	2(2)	5(2)	-5(2)
O008	42(3)	60(4)	28(3)	1(3)	-1(2)	-20(3)
O009	43(3)	40(3)	21(3)	1(2)	1(2)	-5(2)
N00A	31(3)	29(3)	13(3)	0(2)	1(2)	5(3)
O00B	71(4)	47(3)	13(3)	3(2)	-1(2)	-14(3)
C00C	33(3)	30(3)	11(3)	5(3)	-3(3)	-3(3)
C00D	26(3)	32(3)	11(3)	2(3)	2(3)	2(3)
C00E	28(3)	29(3)	20(3)	4(3)	7(3)	6(3)

Supplementary Table 23. Anisotropic Displacement Parameters (Å^2×10^3) for 4. The Anisotropic displacement factor exponent takes the form: $-2\pi^2[h^2a^{*2}U_{11}+2hka^*b^*U_{12}+…]$.

Atom	U_{11}	U_{22}	U_{33}	U_{23}	U_{13}	U_{12}
C00F	30(3)	32(4)	14(3)	7(3)	1(3)	3(3)
C00G	40(4)	33(4)	51(5)	7(4)	-4(4)	-13(4)
C00H	32(3)	19(3)	21(4)	2(3)	3(3)	1(3)
C00I	38(4)	36(4)	36(4)	1(4)	-8(3)	-8(4)
C00J	25(3)	33(4)	13(3)	-2(3)	2(3)	4(3)
C00K	25(3)	23(3)	17(3)	3(3)	3(3)	-1(3)
C00L	33(3)	32(4)	15(3)	1(3)	0(3)	-1(3)
C00M	30(3)	26(3)	23(3)	1(3)	-1(3)	0(3)
C00N	33(4)	31(4)	24(4)	0(3)	4(3)	8(3)
C00O	33(3)	28(3)	15(3)	0(3)	-1(3)	4(3)
C00P	33(3)	37(4)	20(4)	1(3)	-1(3)	-6(3)
C00Q	32(3)	30(4)	7(3)	-2(3)	-3(3)	0(3)
C00R	33(3)	27(3)	19(4)	-3(3)	2(3)	7(3)
C00S	34(3)	35(4)	23(4)	6(3)	-5(3)	0(3)
C00T	25(3)	42(4)	36(5)	21(4)	-4(3)	-4(3)
C00U	31(3)	30(3)	8(3)	-2(3)	4(3)	4(3)
C00V	29(3)	30(3)	22(3)	1(3)	-5(3)	7(3)
C00W	29(3)	30(4)	5(3)	-3(2)	0(2)	3(3)
C00X	40(4)	34(4)	11(4)	-2(3)	0(3)	-6(3)
C00Y	41(4)	36(4)	22(4)	3(3)	2(3)	9(3)
C00Z	42(4)	27(3)	20(4)	8(3)	-8(3)	-2(3)
C010	33(3)	33(4)	14(3)	3(3)	1(3)	4(3)
C011	42(4)	44(5)	31(4)	5(4)	9(4)	19(4)
C012	40(4)	30(4)	16(3)	0(3)	-1(3)	8(3)
C013	43(4)	37(4)	21(3)	1(3)	5(3)	-14(3)
C014	39(4)	45(4)	14(3)	6(3)	4(3)	5(4)
C015	41(4)	27(3)	23(3)	2(3)	-4(3)	-5(3)
C016	38(4)	31(4)	15(3)	-3(3)	-3(3)	4(3)
C017	34(3)	32(4)	21(3)	1(3)	0(3)	-8(3)
C018	37(4)	42(4)	16(4)	8(3)	1(3)	7(3)
C019	42(4)	40(4)	34(4)	10(4)	1(4)	-7(3)
C01A	39(4)	37(4)	34(4)	-6(3)	-2(3)	1(3)
O01B	74(5)	91(5)	55(4)	13(4)	8(4)	5(4)
C01C	43(4)	61(5)	20(3)	6(4)	-1(3)	-9(5)
C01D	41(4)	35(4)	26(4)	4(3)	2(3)	10(4)
C01E	34(3)	40(4)	19(3)	8(3)	-4(3)	-2(4)
C01F	30(3)	37(4)	17(4)	5(3)	0(3)	8(3)
C01G	35(4)	35(4)	27(4)	1(3)	7(3)	6(4)
C01H	59(5)	28(4)	20(4)	-5(3)	-6(3)	0(4)
C01I	43(4)	39(4)	28(4)	2(3)	9(3)	11(4)
C01J	39(4)	35(4)	18(4)	1(3)	3(3)	1(3)
C01K	51(5)	74(6)	25(5)	-12(4)	-5(4)	-7(5)

Supplementary Table 23. Anisotropic Displacement Parameters (Å^2×10^3) for 4. The Anisotropic displacement factor exponent takes the form: -2π^2[h^{2}a*^{2}U$_{11}$+2hka*b*U$_{12}$+…].

Atom	U_{11}	U_{22}	U_{33}	U_{23}	U_{13}	U_{12}
C01L	39(4)	40(4)	34(4)	4(4)	9(3)	8(4)
C01M	55(5)	83(6)	22(4)	5(4)	-11(4)	-25(5)
C01N	56(5)	42(5)	37(5)	-10(4)	3(4)	12(4)
C01O	33(4)	55(5)	25(4)	10(4)	0(3)	4(4)
C01P	61(6)	75(7)	43(5)	6(5)	-6(4)	-37(5)
C01Q	37(4)	68(6)	32(4)	25(4)	-6(3)	0(4)
C01R	62(5)	29(4)	38(5)	2(3)	1(4)	-5(4)
C01S	55(5)	25(4)	19(4)	1(3)	0(3)	-9(3)
C01T	50(4)	42(4)	33(4)	8(4)	-3(3)	16(4)
C01U	49(5)	51(5)	57(6)	-11(4)	11(5)	-16(5)
C01V	67(6)	71(6)	50(6)	-19(5)	17(5)	-16(5)
C01W	69(6)	57(6)	50(6)	6(5)	-2(5)	25(5)
C01X	39(4)	60(6)	62(7)	40(5)	-10(4)	3(4)
C01Y	62(5)	67(6)	27(4)	-11(4)	-2(4)	-25(5)
C01Z	63(6)	49(5)	75(8)	22(5)	-9(6)	13(5)
C020	57(5)	30(4)	34(4)	12(4)	0(4)	0(4)
C021	65(6)	79(7)	49(6)	1(5)	4(5)	-23(6)
C022	108(10)	63(7)	120(12)	4(8)	-10(9)	17(7)
C023	215(18)	53(6)	41(6)	-10(5)	20(8)	19(8)
C024	73(6)	67(6)	35(5)	0(5)	0(5)	-20(5)
C025	123(11)	72(8)	103(11)	-1(8)	-23(9)	9(8)
C028	99(10)	104(11)	202(19)	70(12)	-70(12)	-6(9)

Supplementary Table 24. Bond Lengths for 4.

Atom	Atom	Length/Å	Atom	Atom	Length/Å
Pd01	P002	2.3368(18)	C00R	C012	1.407(9)
Pd01	P003	2.3453(17)	C00S	C019	1.389(10)
Pd01	C00X	2.107(7)	C00T	C01Q	1.417(10)
Pd01	C015	2.124(7)	C00T	C01T	1.389(11)
P002	C00F	1.851(7)	C00U	C010	1.543(9)
P002	C00H	1.830(7)	C00V	C010	1.525(9)
P002	C00K	1.845(7)	C00V	C01D	1.385(10)
P003	C00C	1.842(7)	C00V	C01J	1.414(9)
P003	C00T	1.823(8)	C00X	C013	1.432(10)
P003	C01E	1.825(7)	C00X	C015	1.439(9)
O004	C00J	1.228(8)	C00Y	C012	1.376(10)

Supplementary Table 24. Bond Lengths for 4.

Atom	Atom	Length/Å	Atom	Atom	Length/Å
O005	C01U	1.428(9)	C00Y	C01I	1.395(11)
O005	C01V	1.425(10)	C00Z	C017	1.369(10)
O006	C00W	1.214(7)	C011	C01N	1.384(12)
N007	C00O	1.450(8)	C011	C01O	1.400(11)
N007	C00W	1.341(8)	C012	C016	1.512(10)
O008	C013	1.195(8)	C014	C018	1.389(10)
O009	C013	1.429(9)	C015	C01S	1.453(10)
O009	C01S	1.396(9)	C016	C01J	1.499(10)
N00A	C00J	1.348(8)	C018	C01F	1.373(9)
N00A	C00U	1.460(8)	C01A	C01N	1.377(11)
O00B	C01S	1.208(8)	O01B	C022	1.502(16)
C00C	C00M	1.392(9)	O01B	C023	1.422(12)
C00C	C00Q	1.416(9)	C01C	C01E	1.409(10)
C00D	C00J	1.484(9)	C01C	C01K	1.367(11)
C00D	C00K	1.408(9)	C01D	C020	1.378(11)
C00D	C01F	1.401(9)	C01E	C01M	1.372(11)
C00E	C00K	1.390(9)	C01G	C01O	1.376(10)
C00E	C014	1.373(9)	C01H	C01J	1.385(10)
C00F	C01A	1.380(10)	C01H	C01R	1.392(10)
C00F	C01G	1.390(10)	C01I	C01L	1.393(11)
C00G	C00I	1.379(11)	C01K	C01Y	1.378(12)
C00G	C019	1.375(11)	C01M	C01P	1.390(12)
C00H	C00P	1.394(9)	C01P	C01Y	1.380(12)
C00H	C00S	1.376(9)	C01Q	C01X	1.415(12)
C00I	C00P	1.388(10)	C01R	C020	1.377(11)
C00L	C00Q	1.382(9)	C01T	C01W	1.384(11)
C00L	C017	1.399(10)	C01U	C021	1.515(13)
C00M	C00Z	1.372(10)	C01V	C024	1.483(12)
C00N	C00R	1.379(9)	C01W	C01Z	1.362(13)
C00N	C01L	1.388(10)	C01X	C01Z	1.387(15)
C00O	C00U	1.559(9)	C021	C024	1.519(13)
C00O	C016	1.549(9)	C022	C025	1.381(17)
C00Q	C00W	1.502(9)	C023	C028	1.60(2)
C00R	C010	1.515(9)	C025	C028	1.409(19)

Supplementary Table 25. Bond Angles for 4.

Atom	Atom	Atom	Angle/˚	Atom	Atom	Atom	Angle/˚
P002	Pd01	P003	113.41(6)	O006	C00W	C00Q	120.6(6)
C00X	Pd01	P002	141.90(18)	N007	C00W	C00Q	116.4(5)
C00X	Pd01	P003	104.64(18)	C013	C00X	Pd01	109.1(5)
C00X	Pd01	C015	39.8(2)	C013	C00X	C015	107.7(6)
C015	Pd01	P002	102.97(19)	C015	C00X	Pd01	70.8(4)
C015	Pd01	P003	142.99(19)	C012	C00Y	C01I	118.9(7)
C00F	P002	Pd01	107.2(2)	C017	C00Z	C00M	119.8(6)

Supplementary Table 25. Bond Angles for 4.

Atom	Atom	Atom	Angle/˚	Atom	Atom	Atom	Angle/˚
C00H	P002	Pd01	127.9(2)	C00R	C010	C00U	102.6(5)
C00H	P002	C00F	100.1(3)	C00R	C010	C00V	110.1(5)
C00H	P002	C00K	103.9(3)	C00V	C010	C00U	108.1(5)
C00K	P002	Pd01	112.0(2)	C01N	C011	C01O	118.8(7)
C00K	P002	C00F	102.4(3)	C00R	C012	C016	112.8(6)
C00C	P003	Pd01	116.3(2)	C00Y	C012	C00R	120.9(7)
C00T	P003	Pd01	114.0(3)	C00Y	C012	C016	125.9(6)
C00T	P003	C00C	102.6(3)	O008	C013	O009	117.3(6)
C00T	P003	C01E	100.2(3)	O008	C013	C00X	134.2(7)
C01E	P003	Pd01	118.1(2)	O009	C013	C00X	108.4(6)
C01E	P003	C00C	103.1(3)	C00E	C014	C018	120.1(6)
C01V	O005	C01U	105.4(6)	C00X	C015	Pd01	69.5(4)
C00W	N007	C00O	119.9(6)	C00X	C015	C01S	106.3(6)
C01S	O009	C013	107.7(5)	C01S	C015	Pd01	109.4(5)
C00J	N00A	C00U	118.0(6)	C012	C016	C00O	105.1(6)
C00M	C00C	P003	120.6(5)	C01J	C016	C00O	106.4(5)
C00M	C00C	C00Q	116.9(6)	C01J	C016	C012	109.9(6)
C00Q	C00C	P003	122.4(5)	C00Z	C017	C00L	119.5(6)
C00K	C00D	C00J	118.4(6)	C01F	C018	C014	119.4(6)
C01F	C00D	C00J	121.7(6)	C00G	C019	C00S	120.5(7)
C01F	C00D	C00K	119.9(6)	C01N	C01A	C00F	121.0(7)
C014	C00E	C00K	122.0(6)	C023	O01B	C022	112.9(10)
C01A	C00F	P002	123.1(5)	C01K	C01C	C01E	120.1(7)
C01A	C00F	C01G	118.3(7)	C020	C01D	C00V	119.4(7)
C01G	C00F	P002	118.3(5)	C01C	C01E	P003	123.1(5)
C019	C00G	C00I	119.0(7)	C01M	C01E	P003	118.5(6)
C00P	C00H	P002	122.2(5)	C01M	C01E	C01C	118.4(7)
C00S	C00H	P002	119.5(5)	C018	C01F	C00D	120.8(6)
C00S	C00H	C00P	118.2(6)	C01O	C01G	C00F	121.5(7)
C00G	C00I	C00P	120.7(7)	C01J	C01H	C01R	120.3(7)
O004	C00J	N00A	120.5(6)	C01L	C01I	C00Y	120.1(7)
O004	C00J	C00D	119.2(6)	C00V	C01J	C016	113.2(6)
N00A	C00J	C00D	120.3(6)	C01H	C01J	C00V	118.8(6)
C00D	C00K	P002	122.2(5)	C01H	C01J	C016	128.0(6)
C00E	C00K	P002	120.1(5)	C01C	C01K	C01Y	121.3(8)
C00E	C00K	C00D	117.7(6)	C00N	C01L	C01I	120.9(7)
C00Q	C00L	C017	120.8(6)	C01E	C01M	C01P	121.0(8)
C00Z	C00M	C00C	122.8(6)	C01A	C01N	C011	120.7(8)
C00R	C00N	C01L	119.1(7)	C01G	C01O	C011	119.7(8)
N007	C00O	C00U	110.5(5)	C01Y	C01P	C01M	120.2(8)
N007	C00O	C016	111.9(5)	C01X	C01Q	C00T	118.8(8)
C016	C00O	C00U	109.6(5)	C020	C01R	C01H	119.9(7)
C00I	C00P	C00H	120.5(7)	O009	C01S	C015	109.2(6)
C00C	C00Q	C00W	118.7(6)	O00B	C01S	O009	118.7(7)
C00L	C00Q	C00C	120.1(6)	O00B	C01S	C015	132.0(8)
C00L	C00Q	C00W	121.2(6)	C01W	C01T	C00T	122.6(7)

Supplementary Table 25. Bond Angles for 4.

Atom	Atom	Atom	Angle/˚	Atom	Atom	Atom	Angle/˚
C00N	C00R	C010	126.5(6)	O005	C01U	C021	105.9(7)
C00N	C00R	C012	120.1(7)	O005	C01V	C024	106.3(7)
C012	C00R	C010	113.1(6)	C01Z	C01W	C01T	121.7(9)
C00H	C00S	C019	121.2(7)	C01Z	C01X	C01Q	122.8(8)
C01Q	C00T	P003	124.1(7)	C01K	C01Y	C01P	119.0(8)
C01T	C00T	P003	118.8(5)	C01W	C01Z	C01X	117.2(9)
C01T	C00T	C01Q	116.9(7)	C01R	C020	C01D	121.1(7)
N00A	C00U	C00O	113.2(5)	C01U	C021	C024	102.8(7)
N00A	C00U	C010	111.6(5)	C025	C022	O01B	102.3(11)
C010	C00U	C00O	109.1(5)	O01B	C023	C028	100.2(11)
C01D	C00V	C010	127.1(6)	C01V	C024	C021	106.1(7)
C01D	C00V	C01J	120.5(6)	C022	C025	C028	113.5(15)
C01J	C00V	C010	112.4(6)	C025	C028	C023	102.6(11)
O006	C00W	N007	123.1(6)				

Supplementary Table 26. Torsion Angles for 4.

A	B	C	D	Angle/˚	A	B	C	D	Angle/˚
Pd01	P002	C00F	C01A	-147.9(5)	C00T	P003	C00C	C00M	-5.2(6)
Pd01	P002	C00F	C01G	26.3(6)	C00T	P003	C00C	C00Q	172.2(5)
Pd01	P002	C00H	C00P	-144.5(5)	C00T	P003	C01E	C01C	104.4(7)
Pd01	P002	C00H	C00S	38.3(7)	C00T	P003	C01E	C01M	-73.1(7)
Pd01	P002	C00K	C00D	60.7(6)	C00T	C01Q	C01X	C01Z	0.8(13)
Pd01	P002	C00K	C00E	-118.1(5)	C00T	C01T	C01W	C01Z	-1.5(15)
Pd01	P003	C00C	C00M	-130.4(5)	C00U	N00A	C00J	O004	8.4(9)
Pd01	P003	C00C	C00Q	47.0(6)	C00U	N00A	C00J	C00D	-170.0(6)
Pd01	P003	C00T	C01Q	-160.7(6)	C00U	C00O	C016	C012	-55.8(7)
Pd01	P003	C00T	C01T	24.1(7)	C00U	C00O	C016	C01J	60.8(7)
Pd01	P003	C01E	C01C	-131.1(6)	C00V	C01D	C020	C01R	-1.6(11)
Pd01	P003	C01E	C01M	51.3(7)	C00W	N007	C00O	C00U	-130.7(6)
Pd01	C00X	C013	O008	108.6(9)	C00W	N007	C00O	C016	106.8(7)
Pd01	C00X	C013	O009	-70.6(6)	C00X	C015	C01S	O009	-5.5(7)
Pd01	C00X	C015	C01S	105.1(5)	C00X	C015	C01S	O00B	173.5(8)
Pd01	C015	C01S	O009	67.9(6)	C00Y	C012	C016	C00O	-113.0(8)
Pd01	C015	C01S	O00B	-113.0(8)	C00Y	C012	C016	C01J	132.9(7)
P002	C00F	C01A	C01N	174.4(6)	C00Y	C01I	C01L	C00N	2.0(11)
P002	C00F	C01G	C01O	-173.8(6)	C010	C00R	C012	C00Y	174.3(6)
P002	C00H	C00P	C00I	-177.2(6)	C010	C00R	C012	C016	0.9(8)
P002	C00H	C00S	C019	177.4(6)	C010	C00V	C01D	C020	-179.4(7)
P003	C00C	C00M	C00Z	179.6(5)	C010	C00V	C01J	C016	-1.0(8)
P003	C00C	C00Q	C00L	-179.2(5)	C010	C00V	C01J	C01H	179.2(6)
P003	C00C	C00Q	C00W	2.9(8)	C012	C00R	C010	C00U	-63.1(7)
P003	C00T	C01Q	C01X	-174.3(6)	C012	C00R	C010	C00V	51.8(7)
P003	C00T	C01T	C01W	174.9(7)	C012	C00Y	C01I	C01L	-1.4(11)
P003	C01E	C01M	C01P	178.4(8)	C012	C016	C01J	C00V	54.0(8)

Supplementary Table 26. Torsion Angles for 4.

A	B	C	D	Angle/˚	A	B	C	D	Angle/˚
O005	C01U	C021	C024	-24.7(10)	C012	C016	C01J	C01H	-126.2(8)
O005	C01V	C024	C021	18.4(11)	C013	O009	C01S	O00B	-170.8(6)
N007	C00O	C00U	N00A	106.8(6)	C013	O009	C01S	C015	8.4(7)
N007	C00O	C00U	C010	-128.4(6)	C013	C00X	C015	Pd01	-104.6(5)
N007	C00O	C016	C012	67.3(7)	C013	C00X	C015	C01S	0.5(8)
N007	C00O	C016	C01J	-176.2(6)	C014	C00E	C00K	P002	178.3(6)
N00A	C00U	C010	C00R	-171.8(5)	C014	C00E	C00K	C00D	-0.6(10)
N00A	C00U	C010	C00V	71.9(7)	C014	C018	C01F	C00D	0.7(11)
C00C	P003	C00T	C01Q	72.6(7)	C015	C00X	C013	O008	-176.2(9)
C00C	P003	C00T	C01T	-102.6(6)	C015	C00X	C013	O009	4.6(8)
C00C	P003	C01E	C01C	-1.2(7)	C016	C00O	C00U	N00A	-129.3(6)
C00C	P003	C01E	C01M	-178.8(7)	C016	C00O	C00U	C010	-4.5(7)
C00C	C00M	C00Z	C017	-2.3(11)	C017	C00L	C00Q	C00C	1.7(10)
C00C	C00Q	C00W	O006	22.1(9)	C017	C00L	C00Q	C00W	179.6(6)
C00C	C00Q	C00W	N007	-158.1(6)	C019	C00G	C00I	C00P	0.5(12)
C00E	C014	C018	C01F	0.4(11)	C01A	C00F	C01G	C01O	0.7(10)
C00F	P002	C00H	C00P	94.2(6)	O01B	C022	C025	C028	-20.4(16)
C00F	P002	C00H	C00S	-83.0(6)	O01B	C023	C028	C025	-25.9(15)
C00F	P002	C00K	C00D	175.3(5)	C01C	C01E	C01M	C01P	0.7(13)
C00F	P002	C00K	C00E	-3.5(6)	C01C	C01K	C01Y	C01P	0.6(15)
C00F	C01A	C01N	C011	-0.1(13)	C01D	C00V	C010	C00R	130.0(7)
C00F	C01G	C01O	C011	-1.6(11)	C01D	C00V	C010	C00U	-118.7(7)
C00G	C00I	C00P	C00H	-0.4(12)	C01D	C00V	C01J	C016	177.4(6)
C00H	P002	C00F	C01A	-12.8(6)	C01D	C00V	C01J	C01H	-2.4(10)
C00H	P002	C00F	C01G	161.4(5)	C01E	P003	C00C	C00M	98.6(6)
C00H	P002	C00K	C00D	-80.8(6)	C01E	P003	C00C	C00Q	-84.0(6)
C00H	P002	C00K	C00E	100.4(6)	C01E	P003	C00T	C01Q	-33.5(7)
C00H	C00S	C019	C00G	0.1(12)	C01E	P003	C00T	C01T	151.3(6)
C00I	C00G	C019	C00S	-0.4(12)	C01E	C01C	C01K	C01Y	0.9(14)
C00J	N00A	C00U	C00O	-70.8(7)	C01E	C01M	C01P	C01Y	0.8(15)
C00J	N00A	C00U	C010	165.8(6)	C01F	C00D	C00J	O004	-164.1(6)
C00J	C00D	C00K	P002	5.1(9)	C01F	C00D	C00J	N00A	14.3(10)
C00J	C00D	C00K	C00E	-176.1(6)	C01F	C00D	C00K	P002	-177.2(5)
C00J	C00D	C01F	C018	175.9(6)	C01F	C00D	C00K	C00E	1.6(10)
C00K	P002	C00F	C01A	94.0(6)	C01G	C00F	C01A	C01N	0.1(11)
C00K	P002	C00F	C01G	-91.7(6)	C01H	C01R	C020	C01D	0.5(12)
C00K	P002	C00H	C00P	-11.4(6)	C01I	C00Y	C012	C00R	0.0(10)
C00K	P002	C00H	C00S	171.3(5)	C01I	C00Y	C012	C016	172.4(7)
C00K	C00D	C00J	O004	13.5(9)	C01J	C00V	C010	C00R	-51.8(7)
C00K	C00D	C00J	N00A	-168.0(6)	C01J	C00V	C010	C00U	59.6(7)
C00K	C00D	C01F	C018	-1.7(11)	C01J	C00V	C01D	C020	2.5(10)
C00K	C00E	C014	C018	-0.4(11)	C01J	C01H	C01R	C020	-0.4(12)
C00L	C00Q	C00W	O006	-155.8(6)	C01K	C01C	C01E	P003	-179.1(7)
C00L	C00Q	C00W	N007	24.0(8)	C01K	C01C	C01E	C01M	-1.6(12)
C00M	C00C	C00Q	C00L	-1.7(9)	C01L	C00N	C00R	C010	-172.7(7)
C00M	C00C	C00Q	C00W	-179.6(6)	C01L	C00N	C00R	C012	-0.3(10)

Supplementary Table 26. Torsion Angles for 4.

A	B	C	D	Angle/˚	A	B	C	D	Angle/˚
C00M	C00Z	C017	C00L	2.1(10)	C01M	C01P	C01Y	C01K	-1.5(15)
C00N	C00R	C010	C00U	109.8(7)	C01N	C011	C01O	C01G	1.5(11)
C00N	C00R	C010	C00V	-135.3(7)	C01O	C011	C01N	C01A	-0.7(13)
C00N	C00R	C012	C00Y	0.9(10)	C01Q	C00T	C01T	C01W	-0.7(12)
C00N	C00R	C012	C016	-172.5(6)	C01Q	C01X	C01Z	C01W	-2.9(14)
C00O	N007	C00W	O006	-11.9(9)	C01R	C01H	C01J	C00V	1.3(11)
C00O	N007	C00W	C00Q	168.3(5)	C01R	C01H	C01J	C016	-178.4(7)
C00O	C00U	C010	C00R	62.5(6)	C01S	O009	C013	O008	172.6(7)
C00O	C00U	C010	C00V	-53.9(7)	C01S	O009	C013	C00X	-8.0(8)
C00O	C016	C01J	C00V	-59.3(7)	C01T	C00T	C01Q	C01X	1.0(11)
C00O	C016	C01J	C01H	120.5(8)	C01T	C01W	C01Z	C01X	3.2(15)
C00P	C00H	C00S	C019	0.0(10)	C01U	O005	C01V	C024	-34.6(9)
C00Q	C00C	C00M	C00Z	2.0(10)	C01U	C021	C024	C01V	3.8(11)
C00Q	C00L	C017	C00Z	-1.9(10)	C01V	O005	C01U	C021	37.3(9)
C00R	C00N	C01L	C01I	-1.1(11)	C022	O01B	C023	C028	15.6(13)
C00R	C012	C016	C00O	60.0(7)	C022	C025	C028	C023	30.0(18)
C00R	C012	C016	C01J	-54.1(8)	C023	O01B	C022	C025	0.8(14)
C00S	C00H	C00P	C00I	0.1(10)					

Supplementary Table 27. Hydrogen Atom Coordinates (Å×10^4) and Isotropic Displacement Parameters (Å^2×10^3) for 4.

Atom	*x*	*y*	*z*	U(eq)
H007	8761.48	3850.04	2885.77	29
H00A	8416.04	2574.79	4421.8	29
H00E	5890.29	4820.48	5376.59	31
H00G	8816.48	7331.78	4012.48	50
H00I	8893.36	6543.09	4741.82	44
H00L	8748.17	4781.63	2396.01	32
H00M	6017.41	6170.32	2413.87	31
H00N	11458.81	2843.46	3554.25	35
H00O	7716.48	2682.17	3348.67	30
H00P	7868.6	5501.8	4861.48	36
H00S	6658.81	6046.22	3527.59	37
H00U	9242.35	3474.71	3704.9	28
H00X	5266.06	2550.3	3295.64	34
H00Y	9762.4	2529.82	2046.48	39
H00Z	7298.76	6759.53	2076.64	36
H010	10057.96	2337.59	4062.63	32
H011	3341.36	6669.15	5037.68	47
H014	6366.3	4107.58	6079.46	39
H015	6078.14	2592.4	4106.73	37
H016	8402.38	2078.2	2590.56	33
H017	8685.63	6092.77	2092.5	35
H018	7486.42	3124.92	5994.76	38

Supplementary Table 27. Hydrogen Atom Coordinates (Å×10⁴) and Isotropic Displacement Parameters (Å²×10³) for 4.

Atom	*x*	*y*	*z*	U(eq)
H_{019}	7688.17	7082.26	3409.56	46
H_{01A}	6041.33	6466.36	4768.75	44
H_{01C}	6059.78	4300.4	1790.21	49
H_{01D}	9536.2	928.68	4367.49	41
H_{01F}	8122.48	2876.54	5200.8	34
H_{01G}	4370.65	4632.12	4453.76	39
H_{01H}	7934.02	634.1	2835.63	43
H_{01I}	11263.59	3016.73	2014.81	44
H_{01K}	5453.69	3535.26	1143.93	60
H_{01L}	12083.54	3206.32	2768.34	45
H_{01M}	3966.92	3665.54	2705.59	64
H_{01N}	4810.38	7205.51	5050.16	54
H_{01O}	3123.99	5373.13	4714.77	45
H_{01P}	3345.69	2920.83	2041.75	71
H_{01Q}	4408.39	5546.17	2070.99	55
H_{01R}	7974.33	-401.61	3430.71	51
H_{01T}	4747.4	5702.46	3601.67	50
H_{01B}	10317.65	5306.3	2622.76	63
H_{01E}	10931.12	4563.55	2773.28	63
H_{01J}	9264.35	5096.34	3851.61	75
H_{01S}	9115.41	5664.46	3374.77	75
H_{01W}	3831.04	6813.67	3623.42	70
H_{01X}	3489.32	6690.59	2115.12	64
H_{01Y}	4106.19	2831.33	1261.74	63
H_{01Z}	3249.49	7366.52	2881.92	75
H_{020}	8785.39	-254.08	4183.58	49
H_{02A}	11341.12	5987.93	3093.04	78
H_{02B}	11613.95	5224.49	3419.02	78
H_{022}	6793.09	8003.39	5801.32	116
H_{02E}	5614.58	5958.54	5984.57	124
H_{02F}	5587.1	6199.71	6573.36	124
H_{02C}	10618.12	5597.48	4014.76	70
H_{02D}	10296.69	6323.67	3671.24	70
H_{025}	7809.81	6805.94	5749.84	120
H_{02G}	7187.8	6321	6620.33	162
H_{02H}	7168.38	5705.29	6158.45	162

Supplementary Table 28. Solvent masks information for 4.

Number	X	Y	Z	Volume	Electron count	Content
1	-0.106	-0.024	-0.012	345.9	35.3	THF
2	0.106	0.476	0.512	345.9	35.2	THF
3	0.394	0.524	0.012	345.9	35.8	THF

Supplementary Table 28. Solvent masks information for 4.

Number	X	Y	Z	Volume	Electron count	Content
4	0.606	0.024	0.488	345.9	35.6	THF

Refinement model description

Number of restraints - 0, number of constraints - unknown.

Details:

1. Fixed Uiso

At 1.2 times of:

All C(H) groups, All C(H,H) groups, All N(H) groups

2.a Ternary CH refined with riding coordinates:

C00O(H00O), C00U(H00U), C00X(H00X), C010(H010), C015(H015), C016(H016)

2.b Secondary CH2 refined with riding coordinates:

C01U(H01B,H01E), C01V(H01J,H01S), C021(H02A,H02B), C023(H02E,H02F), C024(H02C, H02D), C028(H02G,H02H)

2.c Aromatic/amide H refined with riding coordinates:

N007(H007), N00A(H00A), C00E(H00E), C00G(H00G), C00I(H00I), C00L(H00L), C00M(H00M), C00N(H00N), C00P(H00P), C00S(H00S), C00Y(H00Y), C00Z(H00Z), C011(H011), C014(H014), C017(H017), C018(H018), C019(H019), C01A(H01A), C01C(H01C), C01D(H01D), C01F(H01F), C01G(H01G), C01H(H01H), C01I(H01I), C01K(H01K), C01L(H01L), C01M(H01M), C01N(H01N), C01O(H01O), C01P(H01P), C01Q(H01Q), C01R(H01R), C01T(H01T), C01W(H01W), C01X(H01X), C01Y(H01Y), C01Z(H01Z), C020(H020), C022(H022), C025(H025)

[PNNP(^{Ph}ANDEN)]-Pd^{II} (4[O])

Single crystals of $C_{59}H_{52}N_2O_3P_2Pd$ (**4[O]**) were selected using a MitEGen loop and paratone oil. A suitable crystal was selected and run on a Bruker APEX-II CCD diffractometer. The crystal was kept at 99.93 K during data collection. Using Olex2[22], the structure was solved with the olex2.solve[23] structure solution program using Charge Flipping and refined with the XL[24] refinement package using Least Squares minimisation.

Crystal structure determination of 4[O]

Crystal Data for $C_{59}H_{52}N_2O_3P_2Pd$ (M = 1005.42 g/mol): monoclinic, space group $P2_1/n$ (no. 14), a = 15.203(11) Å, b = 21.082(15) Å, c = 16.227(12) Å, β = 112.831(15)°, V = 4793(6) Å^3, Z = 43, T = 99.93 K, μ(MoKα) = 0.503 mm^{-1}, $Dcalc$ = 1.393 g/cm^3, 38884 reflections measured (3.118° ≤ 2Θ ≤ 52.68°), 9436 unique (R_{int} = 0.1010, R_{sigma} = 0.1003) which were used in all calculations. The final R_1 was 0.0511 (I > 2σ(I)) and wR_2 was 0.1192 (all data).

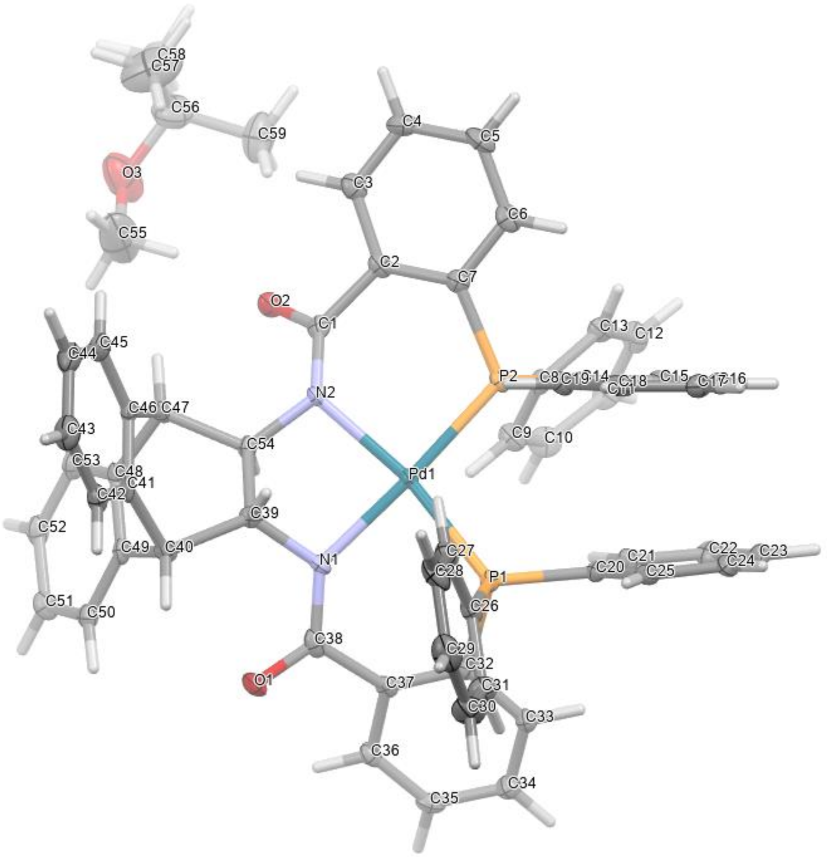

Supplementary Figure 126: Solid state molecular structure of **4[O]** with thermal ellipsoids plotted at 50% probability. Hydrogen atoms are shown as capped sticks. Asymmetric unit contains one molecule of *tert*-butyl methyl ether (TBME) solvent. Image generated using Mercury 2021.3.0. CCDC deposition number 2258960.

Supplementary Table 29. Crystal data and structure refinement for (4[O]).

Identification code	**4[O]**
Empirical formula	$C_{59}H_{52}N_2O_3P_2Pd$
Formula weight	1005.42
Temperature/K	99.93
Crystal system	monoclinic
Space group	$P2_1/n$
a/Å	15.203(11)
b/Å	21.082(15)
c/Å	16.227(12)
α/°	90
β/°	112.831(15)
γ/°	90
Volume/$Å^3$	4793(6)
Z	43
$\rho_{calc}g/cm^3$	1.393
μ/mm^{-1}	0.503
F(000)	2080.0
Crystal size/mm^3	0.01 × 0.008 × 0.007
Radiation	MoKα (λ = 0.71073)
2Θ range for data collection/°	3.118 to 52.68
Index ranges	-18 ≤ h ≤ 18, -23 ≤ k ≤ 25, -20 ≤ l ≤ 20
Reflections collected	38884
Independent reflections	9436 [R_{int} = 0.1010, R_{sigma} = 0.1003]
Data/restraints/parameters	9436/0/608
Goodness-of-fit on F^2	1.017
Final R indexes [I>=2σ (I)]	R_1 = 0.0511, wR_2 = 0.1045
Final R indexes [all data]	R_1 = 0.0940, wR_2 = 0.1192
Largest diff. peak/hole / e $Å^{-3}$	1.24/-0.86

Supplementary Table 30. Fractional Atomic Coordinates (×10^4) and Equivalent Isotropic Displacement Parameters ($Å^2$×10^3) for 4[O]. U_{eq} is defined as 1/3 of of the trace of the orthogonalised U_{IJ} tensor.

Atom	*x*	*y*	*z*	U(eq)
Pd_1	7118.8(2)	6429.4(2)	6947.4(2)	11.04(9)
P_1	6611.1(8)	6785.0(5)	8002.8(7)	12.5(2)
P_2	7509.2(8)	5415.5(5)	7423.2(7)	12.5(2)
O_1	5943(2)	8300.1(15)	6007(2)	32.9(9)
O_2	7473(2)	5650.9(13)	4745.8(18)	18.9(7)
N_1	6685(2)	7322.3(15)	6370(2)	13.1(7)
N_2	7671(2)	6299.8(15)	5949(2)	12.6(7)
C_1	7748(3)	5745(2)	5564(3)	14.4(9)
C_2	8330(3)	5207.6(19)	6160(3)	14.0(9)
C_3	8896(3)	4856(2)	5817(3)	20.9(10)
C_4	9462(3)	4357(2)	6282(3)	23.0(10)
C_5	9461(3)	4187(2)	7109(3)	23.7(11)

Supplementary Table 30. Fractional Atomic Coordinates (×10[4]) and Equivalent Isotropic Displacement Parameters (Å[2]×10[3]) for 4[O]. U_{eq} is defined as 1/3 of of the trace of the orthogonalised U_{IJ} tensor.

Atom	*x*	*y*	*z*	U(eq)
C_6	8897(3)	4523(2)	7464(3)	18.6(10)
C_7	8334(3)	5040(2)	6994(3)	15.3(9)
C_8	6454(3)	4906.0(19)	7000(3)	14.2(9)
C_9	5576(3)	5163(2)	6442(3)	19.9(10)
C_{10}	4753(3)	4795(2)	6144(3)	24.4(11)
C_{11}	4790(3)	4169(2)	6422(3)	24.5(11)
C_{12}	5648(3)	3901(2)	6955(3)	23.9(11)
C_{13}	6490(3)	4259(2)	7233(3)	18.9(10)
C_{14}	8081(3)	5299.1(19)	8624(3)	12.9(9)
C_{15}	7748(3)	4883.0(19)	9107(3)	13.7(9)
C_{16}	8222(3)	4846(2)	10031(3)	17.4(10)
C_{17}	9011(3)	5214(2)	10467(3)	19.8(10)
C_{18}	9355(3)	5635(2)	9992(3)	18.3(10)
C_{19}	8882(3)	5672.3(19)	9072(3)	15.9(9)
C_{20}	6415(3)	6236.3(19)	8779(3)	14.7(9)
C_{21}	5698(3)	5775(2)	8447(3)	16.9(9)
C_{22}	5516(3)	5345(2)	9013(3)	19.4(10)
C_{23}	6082(3)	5364(2)	9932(3)	21.6(10)
C_{24}	6799(3)	5816(2)	10258(3)	19.2(10)
C_{25}	6966(3)	6256.6(19)	9699(3)	15.5(9)
C_{26}	7445(3)	7388.6(19)	8677(3)	15.6(9)
C_{27}	8413(3)	7353(2)	8812(3)	20.5(10)
C_{28}	9072(3)	7784(2)	9354(3)	25.7(11)
C_{29}	8762(4)	8280(2)	9751(3)	27.5(11)
C_{30}	7800(4)	8334(2)	9605(3)	30.1(12)
C_{31}	7137(3)	7885(2)	9081(3)	21.9(10)
C_{32}	5467(3)	7199.7(19)	7501(2)	13.0(9)
C_{33}	4774(3)	7159(2)	7879(3)	17.3(9)
C_{34}	3910(3)	7474(2)	7506(3)	21.4(10)
C_{35}	3712(3)	7836(2)	6737(3)	18.3(10)
C_{36}	4400(3)	7901(2)	6381(3)	16.4(9)
C_{37}	5299(3)	7594.0(18)	6753(3)	12.9(9)
C_{38}	6027(3)	7757(2)	6353(3)	18.8(10)
C_{39}	7361(3)	7430.2(18)	5923(3)	13.1(9)
C_{40}	7367(3)	8040.6(19)	5372(3)	14.4(9)
C_{41}	8364(3)	8003(2)	5352(3)	16.2(9)
C_{42}	9092(3)	8456(2)	5700(3)	19.9(10)
C_{43}	9987(3)	8341(2)	5663(3)	24.9(11)
C_{44}	10153(3)	7775(2)	5298(3)	25.1(11)
C_{45}	9430(3)	7323(2)	4953(3)	22.3(10)
C_{46}	8538(3)	7436(2)	4977(3)	15.0(9)
C_{47}	7663(3)	7011.5(19)	4605(3)	15.7(9)
C_{48}	6853(3)	7430.7(19)	3986(3)	14.2(9)
C_{49}	6657(3)	7961(2)	4412(2)	14.4(9)
C_{50}	5888(3)	8361(2)	3951(3)	16.2(9)

Supplementary Table 30. Fractional Atomic Coordinates (×10^4) and Equivalent Isotropic Displacement Parameters (Å^2×10^3) for 4[O]. U_{eq} is defined as 1/3 of of the trace of the orthogonalised U_{IJ} tensor.

Atom	*x*	*y*	*z*	U(eq)
C_{51}	5329(3)	8237(2)	3055(3)	20.4(10)
C_{52}	5529(3)	7723(2)	2628(3)	21.3(10)
C_{53}	6287(3)	7311(2)	3088(3)	19.3(10)
C_{54}	7254(3)	6837.9(19)	5337(2)	13.3(9)
O_3	6932(3)	4982(2)	2092(2)	62.3(13)
C_{55}	7155(6)	5567(3)	2640(4)	81(3)
C_{56}	7477(4)	4439(3)	2519(4)	47.7(15)
C_{57}	8550(4)	4545(3)	2869(5)	72(2)
C_{58}	7154(4)	3949(3)	1773(3)	38.6(14)
C_{59}	7205(4)	4231(3)	3292(3)	52.2(17)

Supplementary Table 31. Anisotropic Displacement Parameters (Å^2×10^3) for 4[O]. The Anisotropic displacement factor exponent takes the form: $-2\pi^2[h^2a^{*2}U_{11}+2hka^*b^*U_{12}+\ldots]$.

Atom	U_{11}	U_{22}	U_{33}	U_{23}	U_{13}	U_{12}
Pd_1	12.03(17)	9.40(16)	10.69(15)	1.10(13)	3.30(12)	0.13(14)
P_1	13.0(6)	12.6(6)	11.5(5)	1.1(4)	4.3(4)	0.0(4)
P_2	12.4(6)	10.8(6)	11.3(5)	1.1(4)	1.4(4)	-1.3(4)
O_1	38(2)	21.7(19)	50(2)	19.7(16)	29.8(18)	15.5(16)
O_2	23.5(18)	16.3(16)	13.2(15)	-2.0(12)	3.2(13)	0.6(13)
N_1	12.9(19)	10.0(18)	14.2(17)	2.7(14)	2.8(15)	2.9(14)
N_2	15.3(19)	9.1(19)	13.8(17)	4.5(13)	6.1(15)	3.4(14)
C_1	9(2)	17(2)	17(2)	0.1(17)	4.5(18)	-2.9(17)
C_2	14(2)	12(2)	12(2)	-1.7(16)	0.3(18)	-2.5(17)
C_3	21(3)	20(3)	17(2)	-0.1(18)	3(2)	1(2)
C_4	20(3)	21(3)	27(2)	-3(2)	9(2)	5(2)
C_5	23(3)	18(3)	25(2)	5.7(19)	3(2)	7(2)
C_6	15(2)	20(2)	15(2)	1.3(18)	-1.1(18)	-1.5(19)
C_7	14(2)	15(2)	15(2)	-1.2(17)	4.1(18)	-1.0(18)
C_8	17(2)	13(2)	14(2)	-2.7(17)	7.1(18)	-5.9(18)
C_9	17(2)	18(2)	21(2)	-0.3(19)	3(2)	-2.4(19)
C_{10}	17(3)	29(3)	20(2)	-6(2)	-1(2)	-3(2)
C_{11}	22(3)	28(3)	25(2)	-12(2)	11(2)	-15(2)
C_{12}	32(3)	16(3)	24(2)	-5(2)	12(2)	-11(2)
C_{13}	22(3)	19(2)	13(2)	-4.3(18)	4.2(19)	-1.0(19)
C_{14}	13(2)	12(2)	12(2)	0.8(17)	2.8(18)	3.0(17)
C_{15}	13(2)	11(2)	16(2)	-0.4(17)	3.7(18)	0.5(17)
C_{16}	21(3)	14(2)	19(2)	2.7(18)	10(2)	4.2(18)
C_{17}	20(3)	25(3)	11(2)	-1.5(18)	2.5(19)	7(2)
C_{18}	15(2)	14(2)	19(2)	-7.6(18)	-1.1(19)	1.2(18)
C_{19}	17(2)	12(2)	17(2)	1.3(17)	3.9(18)	0.2(18)
C_{20}	19(2)	13(2)	12(2)	0.8(16)	7.0(18)	2.1(17)
C_{21}	17(2)	19(2)	13(2)	2.4(18)	4.5(18)	2.8(19)
C_{22}	12(2)	21(2)	28(2)	0.0(19)	10(2)	-2.4(19)

Supplementary Table 31. Anisotropic Displacement Parameters ($Å^2×10^3$) for 4[O]. The Anisotropic displacement factor exponent takes the form: $-2\pi^2[h^2a^{*2}U_{11}+2hka^*b^*U_{12}+…]$.

Atom	U_{11}	U_{22}	U_{33}	U_{23}	U_{13}	U_{12}
C_{23}	22(3)	24(3)	25(2)	11(2)	16(2)	8(2)
C_{24}	20(3)	22(3)	16(2)	5.7(18)	7.7(19)	8(2)
C_{25}	14(2)	14(2)	18(2)	0.5(17)	5.9(19)	2.9(17)
C_{26}	19(3)	12(2)	12(2)	1.6(17)	2.5(18)	-1.0(18)
C_{27}	23(3)	19(2)	18(2)	3.9(19)	6(2)	3(2)
C_{28}	21(3)	20(3)	28(3)	6(2)	0(2)	-2(2)
C_{29}	29(3)	25(3)	20(2)	-2(2)	0(2)	-10(2)
C_{30}	41(3)	23(3)	23(2)	-9(2)	9(2)	0(2)
C_{31}	21(3)	24(3)	21(2)	-3.9(19)	7(2)	-1(2)
C_{32}	14(2)	15(2)	6.9(19)	-1.6(16)	0.9(17)	0.7(17)
C_{33}	19(3)	18(2)	16(2)	4.1(18)	7.9(19)	2.8(19)
C_{34}	20(3)	24(3)	21(2)	2.3(19)	10(2)	3(2)
C_{35}	15(2)	16(2)	19(2)	-2.4(18)	2.0(19)	3.6(18)
C_{36}	18(3)	16(2)	11(2)	2.7(17)	1.2(18)	2.1(18)
C_{37}	16(2)	8(2)	13(2)	-3.2(16)	3.6(18)	-3.0(17)
C_{38}	24(3)	17(2)	11(2)	0.1(18)	1.9(19)	-3.0(19)
C_{39}	13(2)	11(2)	14(2)	-3.2(17)	3.8(18)	-3.0(17)
C_{40}	20(2)	10(2)	12(2)	-1.9(17)	4.9(18)	-0.6(18)
C_{41}	18(2)	20(2)	9.7(19)	7.5(17)	4.5(18)	-1.5(19)
C_{42}	27(3)	17(3)	16(2)	3.6(18)	8(2)	-3.7(19)
C_{43}	20(3)	29(3)	24(2)	1(2)	6(2)	-9(2)
C_{44}	20(3)	35(3)	22(2)	7(2)	10(2)	1(2)
C_{45}	28(3)	25(3)	16(2)	2.9(19)	11(2)	2(2)
C_{46}	18(2)	16(2)	13(2)	5.3(17)	8.3(18)	0.5(18)
C_{47}	25(3)	10(2)	13(2)	1.3(17)	7.4(19)	1.2(18)
C_{48}	21(3)	11(2)	12(2)	3.6(16)	7.0(18)	-3.3(18)
C_{49}	15(2)	17(2)	9.4(19)	3.6(17)	3.0(18)	-1.4(18)
C_{50}	17(2)	14(2)	19(2)	1.0(17)	8.1(19)	-2.5(18)
C_{51}	19(3)	20(2)	17(2)	0.9(19)	1.8(19)	-1.4(19)
C_{52}	29(3)	22(3)	12(2)	-0.2(18)	6(2)	-4(2)
C_{53}	27(3)	19(2)	13(2)	-0.7(18)	7.7(19)	-4(2)
C_{54}	14(2)	15(2)	10.5(19)	0.6(17)	4.0(17)	-1.7(17)
O_3	85(3)	50(3)	35(2)	9(2)	5(2)	5(2)
C_{55}	164(8)	42(4)	42(4)	-8(3)	47(5)	1(5)
C_{56}	38(4)	47(4)	60(4)	5(3)	20(3)	8(3)
C_{57}	46(4)	77(5)	98(6)	-48(4)	35(4)	-28(4)
C_{58}	43(4)	35(3)	45(3)	-2(3)	25(3)	-3(3)
C_{59}	60(4)	68(4)	28(3)	3(3)	16(3)	-12(3)

Supplementary Table 32. Bond Lengths for 4[O].

Atom	Atom	Length/Å	Atom	Atom	Length/Å
Pd_1	P_1	2.2621(16)	C_{23}	C_{24}	1.388(6)
Pd_1	P_2	2.2719(18)	C_{24}	C_{25}	1.389(6)

Supplementary Table 32. Bond Lengths for 4[O].

Atom	Atom	Length/Å	Atom	Atom	Length/Å
Pd_1	N_1	2.092(3)	C_{26}	C_{27}	1.402(6)
Pd_1	N_2	2.111(3)	C_{26}	C_{31}	1.408(6)
P_1	C_{20}	1.818(4)	C_{27}	C_{28}	1.386(6)
P_1	C_{26}	1.830(4)	C_{28}	C_{29}	1.401(7)
P_1	C_{32}	1.832(4)	C_{29}	C_{30}	1.393(7)
P_2	C_7	1.833(4)	C_{30}	C_{31}	1.404(6)
P_2	C_8	1.828(4)	C_{32}	C_{33}	1.413(6)
P_2	C_{14}	1.817(4)	C_{32}	C_{37}	1.410(5)
O_1	C_{38}	1.258(5)	C_{33}	C_{34}	1.384(6)
O_2	C_1	1.243(5)	C_{34}	C_{35}	1.393(6)
N_1	C_{38}	1.350(5)	C_{35}	C_{36}	1.384(6)
N_1	C_{39}	1.488(5)	C_{36}	C_{37}	1.417(6)
N_2	C_1	1.354(5)	C_{37}	C_{38}	1.525(6)
N_2	C_{54}	1.479(5)	C_{39}	C_{40}	1.569(5)
C_1	C_2	1.528(6)	C_{39}	C_{54}	1.540(5)
C_2	C_3	1.404(6)	C_{40}	C_{41}	1.530(6)
C_2	C_7	1.397(6)	C_{40}	C_{49}	1.521(5)
C_3	C_4	1.384(6)	C_{41}	C_{42}	1.404(6)
C_4	C_5	1.390(6)	C_{41}	C_{46}	1.413(6)
C_5	C_6	1.398(6)	C_{42}	C_{43}	1.406(6)
C_6	C_7	1.413(6)	C_{43}	C_{44}	1.398(7)
C_8	C_9	1.399(6)	C_{44}	C_{45}	1.396(6)
C_8	C_{13}	1.411(6)	C_{45}	C_{46}	1.392(6)
C_9	C_{10}	1.391(6)	C_{46}	C_{47}	1.520(6)
C_{10}	C_{11}	1.388(6)	C_{47}	C_{48}	1.531(6)
C_{11}	C_{12}	1.377(7)	C_{47}	C_{54}	1.581(5)
C_{12}	C_{13}	1.402(6)	C_{48}	C_{49}	1.406(6)
C_{14}	C_{15}	1.396(5)	C_{48}	C_{53}	1.399(5)
C_{14}	C_{19}	1.394(6)	C_{49}	C_{50}	1.399(6)
C_{15}	C_{16}	1.391(5)	C_{50}	C_{51}	1.395(6)
C_{16}	C_{17}	1.373(6)	C_{51}	C_{52}	1.383(6)
C_{17}	C_{18}	1.402(6)	C_{52}	C_{53}	1.404(6)
C_{18}	C_{19}	1.385(6)	O_3	C_{55}	1.482(7)
C_{20}	C_{21}	1.403(6)	O_3	C_{56}	1.424(7)
C_{20}	C_{25}	1.400(5)	C_{56}	C_{57}	1.522(8)
C_{21}	C_{22}	1.393(6)	C_{56}	C_{58}	1.521(8)
C_{22}	C_{23}	1.403(6)	C_{56}	C_{59}	1.530(7)

Supplementary Table 33. Bond Angles for 4[O].

Atom	Atom	Atom	Angle/˚	Atom	Atom	Atom	Angle/˚
P_1	Pd_1	P_2	100.10(5)	C_{27}	C_{26}	P_1	119.8(3)
N_1	Pd_1	P_1	84.26(10)	C_{27}	C_{26}	C_{31}	119.1(4)
N_1	Pd_1	P_2	173.78(9)	C_{31}	C_{26}	P_1	121.1(3)
N_1	Pd_1	N_2	85.58(13)	C_{28}	C_{27}	C_{26}	121.3(4)

Supplementary Table 33. Bond Angles for 4[O].

Atom	Atom	Atom	Angle/˚	Atom	Atom	Atom	Angle/˚
N_2	Pd_1	P_1	167.88(9)	C_{27}	C_{28}	C_{29}	119.5(5)
N_2	Pd_1	P_2	90.56(9)	C_{30}	C_{29}	C_{28}	120.0(4)
C_{20}	P_1	Pd_1	120.64(14)	C_{29}	C_{30}	C_{31}	120.5(4)
C_{20}	P_1	C_{26}	106.87(19)	C_{30}	C_{31}	C_{26}	119.5(4)
C_{20}	P_1	C_{32}	103.2(2)	C_{33}	C_{32}	P_1	121.2(3)
C_{26}	P_1	Pd_1	108.80(15)	C_{37}	C_{32}	P_1	119.2(3)
C_{26}	P_1	C_{32}	104.62(19)	C_{37}	C_{32}	C_{33}	119.5(4)
C_{32}	P_1	Pd_1	111.44(13)	C_{34}	C_{33}	C_{32}	121.5(4)
C_7	P_2	Pd_1	113.84(14)	C_{33}	C_{34}	C_{35}	119.5(4)
C_8	P_2	Pd_1	110.47(15)	C_{36}	C_{35}	C_{34}	119.5(4)
C_8	P_2	C_7	103.5(2)	C_{35}	C_{36}	C_{37}	122.5(4)
C_{14}	P_2	Pd_1	116.41(14)	C_{32}	C_{37}	C_{36}	117.3(4)
C_{14}	P_2	C_7	103.65(19)	C_{32}	C_{37}	C_{38}	125.5(4)
C_{14}	P_2	C_8	107.97(19)	C_{36}	C_{37}	C_{38}	117.0(4)
C_{38}	N_1	Pd_1	136.3(3)	O_1	C_{38}	N_1	125.0(4)
C_{38}	N_1	C_{39}	122.2(3)	O_1	C_{38}	C_{37}	115.5(4)
C_{39}	N_1	Pd_1	101.4(2)	N_1	C_{38}	C_{37}	119.5(4)
C_1	N_2	Pd_1	126.7(3)	N_1	C_{39}	C_{40}	124.1(3)
C_1	N_2	C_{54}	116.3(3)	N_1	C_{39}	C_{54}	104.5(3)
C_{54}	N_2	Pd_1	102.9(2)	C_{54}	C_{39}	C_{40}	109.6(3)
O_2	C_1	N_2	125.3(4)	C_{41}	C_{40}	C_{39}	101.2(3)
O_2	C_1	C_2	115.5(4)	C_{49}	C_{40}	C_{39}	109.5(3)
N_2	C_1	C_2	118.8(3)	C_{49}	C_{40}	C_{41}	106.8(3)
C_3	C_2	C_1	115.7(4)	C_{42}	C_{41}	C_{40}	126.1(4)
C_7	C_2	C_1	125.8(4)	C_{42}	C_{41}	C_{46}	119.9(4)
C_7	C_2	C_3	118.5(4)	C_{46}	C_{41}	C_{40}	113.9(4)
C_4	C_3	C_2	122.2(4)	C_{41}	C_{42}	C_{43}	119.4(4)
C_3	C_4	C_5	119.3(4)	C_{44}	C_{43}	C_{42}	120.1(4)
C_4	C_5	C_6	119.9(4)	C_{45}	C_{44}	C_{43}	120.6(4)
C_5	C_6	C_7	120.6(4)	C_{46}	C_{45}	C_{44}	119.7(4)
C_2	C_7	P_2	120.8(3)	C_{41}	C_{46}	C_{47}	112.8(4)
C_2	C_7	C_6	119.5(4)	C_{45}	C_{46}	C_{41}	120.3(4)
C_6	C_7	P_2	119.2(3)	C_{45}	C_{46}	C_{47}	126.9(4)
C_9	C_8	P_2	119.5(3)	C_{46}	C_{47}	C_{48}	106.2(3)
C_9	C_8	C_{13}	118.5(4)	C_{46}	C_{47}	C_{54}	112.1(3)
C_{13}	C_8	P_2	122.0(3)	C_{48}	C_{47}	C_{54}	100.2(3)
C_{10}	C_9	C_8	120.8(4)	C_{49}	C_{48}	C_{47}	114.0(3)
C_{11}	C_{10}	C_9	120.1(4)	C_{53}	C_{48}	C_{47}	126.3(4)
C_{12}	C_{11}	C_{10}	120.2(4)	C_{53}	C_{48}	C_{49}	119.6(4)
C_{11}	C_{12}	C_{13}	120.3(4)	C_{48}	C_{49}	C_{40}	112.9(4)
C_{12}	C_{13}	C_8	120.0(4)	C_{50}	C_{49}	C_{40}	126.5(4)
C_{15}	C_{14}	P_2	124.1(3)	C_{50}	C_{49}	C_{48}	120.6(4)
C_{19}	C_{14}	P_2	116.2(3)	C_{51}	C_{50}	C_{49}	119.3(4)
C_{19}	C_{14}	C_{15}	119.7(4)	C_{52}	C_{51}	C_{50}	120.4(4)
C_{16}	C_{15}	C_{14}	119.4(4)	C_{51}	C_{52}	C_{53}	120.9(4)
C_{17}	C_{16}	C_{15}	120.5(4)	C_{48}	C_{53}	C_{52}	119.2(4)

Supplementary Table 33. Bond Angles for 4[O].

Atom	Atom	Atom	Angle/˚	Atom	Atom	Atom	Angle/˚
C_{16}	C_{17}	C_{18}	120.8(4)	N_{2}	C_{54}	C_{39}	106.9(3)
C_{19}	C_{18}	C_{17}	118.7(4)	N_{2}	C_{54}	C_{47}	119.5(3)
C_{18}	C_{19}	C_{14}	120.9(4)	C_{39}	C_{54}	C_{47}	107.3(3)
C_{21}	C_{20}	P_{1}	119.1(3)	C_{56}	O_{3}	C_{55}	115.0(5)
C_{25}	C_{20}	P_{1}	121.9(3)	O_{3}	C_{56}	C_{57}	113.7(5)
C_{25}	C_{20}	C_{21}	119.0(4)	O_{3}	C_{56}	C_{58}	102.2(5)
C_{22}	C_{21}	C_{20}	121.4(4)	O_{3}	C_{56}	C_{59}	109.8(5)
C_{21}	C_{22}	C_{23}	119.0(4)	C_{57}	C_{56}	C_{59}	109.1(5)
C_{24}	C_{23}	C_{22}	119.5(4)	C_{58}	C_{56}	C_{57}	111.4(5)
C_{23}	C_{24}	C_{25}	121.7(4)	C_{58}	C_{56}	C_{59}	110.5(5)
C_{24}	C_{25}	C_{20}	119.4(4)				

Supplementary Table 34. Torsion Angles for 4[O].

A	B	C	D	Angle/˚	A	B	C	D	Angle/˚
Pd_{1}	P_{1}	C_{20}	C_{21}	-64.3(4)	C_{23}	C_{24}	C_{25}	C_{20}	-1.7(6)
Pd_{1}	P_{1}	C_{20}	C_{25}	115.0(3)	C_{25}	C_{20}	C_{21}	C_{22}	1.0(6)
Pd_{1}	P_{1}	C_{26}	C_{27}	-30.8(3)	C_{26}	P_{1}	C_{20}	C_{21}	170.9(3)
Pd_{1}	P_{1}	C_{26}	C_{31}	150.5(3)	C_{26}	P_{1}	C_{20}	C_{25}	-9.8(4)
Pd_{1}	P_{1}	C_{32}	C_{33}	144.8(3)	C_{26}	P_{1}	C_{32}	C_{33}	-97.8(4)
Pd_{1}	P_{1}	C_{32}	C_{37}	-38.8(3)	C_{26}	P_{1}	C_{32}	C_{37}	78.6(3)
Pd_{1}	P_{2}	C_{7}	C_{2}	-30.5(4)	C_{26}	C_{27}	C_{28}	C_{29}	-2.2(6)
Pd_{1}	P_{2}	C_{7}	C_{6}	157.1(3)	C_{27}	C_{26}	C_{31}	C_{30}	0.3(6)
Pd_{1}	P_{2}	C_{8}	C_{9}	1.9(4)	C_{27}	C_{28}	C_{29}	C_{30}	0.4(7)
Pd_{1}	P_{2}	C_{8}	C_{13}	-176.8(3)	C_{28}	C_{29}	C_{30}	C_{31}	1.8(7)
Pd_{1}	P_{2}	C_{14}	C_{15}	125.3(3)	C_{29}	C_{30}	C_{31}	C_{26}	-2.1(7)
Pd_{1}	P_{2}	C_{14}	C_{19}	-52.5(3)	C_{31}	C_{26}	C_{27}	C_{28}	1.9(6)
Pd_{1}	N_{1}	C_{38}	O_{1}	173.9(3)	C_{32}	P_{1}	C_{20}	C_{21}	60.9(4)
Pd_{1}	N_{1}	C_{38}	C_{37}	-7.8(6)	C_{32}	P_{1}	C_{20}	C_{25}	-119.8(3)
Pd_{1}	N_{1}	C_{39}	C_{40}	180.0(3)	C_{32}	P_{1}	C_{26}	C_{27}	-150.0(3)
Pd_{1}	N_{1}	C_{39}	C_{54}	53.6(3)	C_{32}	P_{1}	C_{26}	C_{31}	31.3(4)
Pd_{1}	N_{2}	C_{1}	O_{2}	130.5(4)	C_{32}	C_{33}	C_{34}	C_{35}	0.5(7)
Pd_{1}	N_{2}	C_{1}	C_{2}	-58.0(5)	C_{32}	C_{37}	C_{38}	O_{1}	-147.9(4)
Pd_{1}	N_{2}	C_{54}	C_{39}	44.4(3)	C_{32}	C_{37}	C_{38}	N_{1}	33.6(6)
Pd_{1}	N_{2}	C_{54}	C_{47}	166.5(3)	C_{33}	C_{32}	C_{37}	C_{36}	-4.2(6)
P_{1}	C_{20}	C_{21}	C_{22}	-179.7(3)	C_{33}	C_{32}	C_{37}	C_{38}	171.1(4)
P_{1}	C_{20}	C_{25}	C_{24}	-178.5(3)	C_{33}	C_{34}	C_{35}	C_{36}	-3.1(6)
P_{1}	C_{26}	C_{27}	C_{28}	-176.8(3)	C_{34}	C_{35}	C_{36}	C_{37}	2.1(6)
P_{1}	C_{26}	C_{31}	C_{30}	179.0(3)	C_{35}	C_{36}	C_{37}	C_{32}	1.5(6)
P_{1}	C_{32}	C_{33}	C_{34}	179.6(3)	C_{35}	C_{36}	C_{37}	C_{38}	-174.1(4)
P_{1}	C_{32}	C_{37}	C_{36}	179.4(3)	C_{36}	C_{37}	C_{38}	O_{1}	27.3(5)
P_{1}	C_{32}	C_{37}	C_{38}	-5.4(5)	C_{36}	C_{37}	C_{38}	N_{1}	-151.2(4)
P_{2}	C_{8}	C_{9}	C_{10}	-176.9(3)	C_{37}	C_{32}	C_{33}	C_{34}	3.3(6)
P_{2}	C_{8}	C_{13}	C_{12}	174.5(3)	C_{38}	N_{1}	C_{39}	C_{40}	-1.7(6)
P_{2}	C_{14}	C_{15}	C_{16}	-177.8(3)	C_{38}	N_{1}	C_{39}	C_{54}	-128.1(4)

Supplementary Table 34. Torsion Angles for 4[O].

A	B	C	D	Angle/˚	A	B	C	D	Angle/˚
P_2	C_{14}	C_{19}	C_{18}	178.1(3)	C_{39}	N_1	C_{38}	O_1	-3.7(6)
O_2	C_1	C_2	C_3	32.1(5)	C_{39}	N_1	C_{38}	C_{37}	174.7(3)
O_2	C_1	C_2	C_7	-147.3(4)	C_{39}	C_{40}	C_{41}	C_{42}	-119.6(4)
N_1	C_{39}	C_{40}	C_{41}	161.7(3)	C_{39}	C_{40}	C_{41}	C_{46}	57.3(4)
N_1	C_{39}	C_{40}	C_{49}	-85.9(4)	C_{39}	C_{40}	C_{49}	C_{48}	-58.6(4)
N_1	C_{39}	C_{54}	N_2	-69.7(4)	C_{39}	C_{40}	C_{49}	C_{50}	122.9(4)
N_1	C_{39}	C_{54}	C_{47}	161.0(3)	C_{40}	C_{39}	C_{54}	N_2	155.3(3)
N_2	C_1	C_2	C_3	-140.2(4)	C_{40}	C_{39}	C_{54}	C_{47}	26.0(4)
N_2	C_1	C_2	C_7	40.3(6)	C_{40}	C_{41}	C_{42}	C_{43}	177.4(4)
C_1	N_2	C_{54}	C_{39}	-172.6(3)	C_{40}	C_{41}	C_{46}	C_{45}	-177.0(3)
C_1	N_2	C_{54}	C_{47}	-50.6(5)	C_{40}	C_{41}	C_{46}	C_{47}	4.8(5)
C_1	C_2	C_3	C_4	179.9(4)	C_{40}	C_{49}	C_{50}	C_{51}	176.9(4)
C_1	C_2	C_7	P_2	6.6(6)	C_{41}	C_{40}	C_{49}	C_{48}	50.2(4)
C_1	C_2	C_7	C_6	178.9(4)	C_{41}	C_{40}	C_{49}	C_{50}	-128.3(4)
C_2	C_3	C_4	C_5	1.2(7)	C_{41}	C_{42}	C_{43}	C_{44}	-1.2(6)
C_3	C_2	C_7	P_2	-172.8(3)	C_{41}	C_{46}	C_{47}	C_{48}	51.5(4)
C_3	C_2	C_7	C_6	-0.5(6)	C_{41}	C_{46}	C_{47}	C_{54}	-56.9(4)
C_3	C_4	C_5	C_6	-0.5(7)	C_{42}	C_{41}	C_{46}	C_{45}	0.1(6)
C_4	C_5	C_6	C_7	-0.6(7)	C_{42}	C_{41}	C_{46}	C_{47}	-178.0(3)
C_5	C_6	C_7	P_2	173.6(3)	C_{42}	C_{43}	C_{44}	C_{45}	1.1(6)
C_5	C_6	C_7	C_2	1.1(6)	C_{43}	C_{44}	C_{45}	C_{46}	-0.4(6)
C_7	P_2	C_8	C_9	-120.3(3)	C_{44}	C_{45}	C_{46}	C_{41}	-0.2(6)
C_7	P_2	C_8	C_{13}	60.9(4)	C_{44}	C_{45}	C_{46}	C_{47}	177.6(4)
C_7	P_2	C_{14}	C_{15}	-108.8(4)	C_{45}	C_{46}	C_{47}	C_{48}	-126.5(4)
C_7	P_2	C_{14}	C_{19}	73.3(3)	C_{45}	C_{46}	C_{47}	C_{54}	125.1(4)
C_7	C_2	C_3	C_4	-0.7(6)	C_{46}	C_{41}	C_{42}	C_{43}	0.6(6)
C_8	P_2	C_7	C_2	89.4(4)	C_{46}	C_{47}	C_{48}	C_{49}	-58.7(4)
C_8	P_2	C_7	C_6	-82.9(4)	C_{46}	C_{47}	C_{48}	C_{53}	125.1(4)
C_8	P_2	C_{14}	C_{15}	0.5(4)	C_{46}	C_{47}	C_{54}	N_2	-84.2(4)
C_8	P_2	C_{14}	C_{19}	-177.3(3)	C_{46}	C_{47}	C_{54}	C_{39}	37.6(4)
C_8	C_9	C_{10}	C_{11}	1.9(7)	C_{47}	C_{48}	C_{49}	C_{40}	6.4(5)
C_9	C_8	C_{13}	C_{12}	-4.3(6)	C_{47}	C_{48}	C_{49}	C_{50}	-175.0(4)
C_9	C_{10}	C_{11}	C_{12}	-3.3(7)	C_{47}	C_{48}	C_{53}	C_{52}	175.8(4)
C_{10}	C_{11}	C_{12}	C_{13}	0.9(6)	C_{48}	C_{47}	C_{54}	N_2	163.6(3)
C_{11}	C_{12}	C_{13}	C_8	2.9(6)	C_{48}	C_{47}	C_{54}	C_{39}	-74.6(4)
C_{13}	C_8	C_9	C_{10}	1.9(6)	C_{48}	C_{49}	C_{50}	C_{51}	-1.5(6)
C_{14}	P_2	C_7	C_2	-157.9(3)	C_{49}	C_{40}	C_{41}	C_{42}	125.8(4)
C_{14}	P_2	C_7	C_6	29.7(4)	C_{49}	C_{40}	C_{41}	C_{46}	-57.2(4)
C_{14}	P_2	C_8	C_9	130.2(3)	C_{49}	C_{48}	C_{53}	C_{52}	-0.2(6)
C_{14}	P_2	C_8	C_{13}	-48.5(4)	C_{49}	C_{50}	C_{51}	C_{52}	0.4(6)
C_{14}	C_{15}	C_{16}	C_{17}	0.0(6)	C_{50}	C_{51}	C_{52}	C_{53}	0.9(7)
C_{15}	C_{14}	C_{19}	C_{18}	0.2(6)	C_{51}	C_{52}	C_{53}	C_{48}	-1.0(6)
C_{15}	C_{16}	C_{17}	C_{18}	0.0(6)	C_{53}	C_{48}	C_{49}	C_{40}	-177.2(4)
C_{16}	C_{17}	C_{18}	C_{19}	0.1(6)	C_{53}	C_{48}	C_{49}	C_{50}	1.5(6)
C_{17}	C_{18}	C_{19}	C_{14}	-0.2(6)	C_{54}	N_2	C_1	O_2	-2.4(6)
C_{19}	C_{14}	C_{15}	C_{16}	-0.1(6)	C_{54}	N_2	C_1	C_2	169.2(3)

Supplementary Table 34. Torsion Angles for 4[O].

A B C D	Angle/˚	A B C D	Angle/˚
C_{20} P_1 C_{26} C_{27}	100.9(3)	C_{54} C_{39} C_{40} C_{41}	-74.2(4)
C_{20} P_1 C_{26} C_{31}	-77.8(4)	C_{54} C_{39} C_{40} C_{49}	38.3(4)
C_{20} P_1 C_{32} C_{33}	13.9(4)	C_{54} C_{47} C_{48} C_{49}	58.0(4)
C_{20} P_1 C_{32} C_{37}	-169.7(3)	C_{54} C_{47} C_{48} C_{53}	-118.2(4)
C_{20} C_{21} C_{22} C_{23}	-1.9(6)	C_{55} O_3 C_{56} C_{57}	-54.8(7)
C_{21} C_{20} C_{25} C_{24}	0.8(6)	C_{55} O_3 C_{56} C_{58}	-175.0(5)
C_{21} C_{22} C_{23} C_{24}	1.1(6)	C_{55} O_3 C_{56} C_{59}	67.7(7)
C_{22} C_{23} C_{24} C_{25}	0.8(6)		

Supplementary Table 35. Hydrogen Atom Coordinates (Å×10^4) and Isotropic Displacement Parameters (Å^2×10^3) for 4[O].

Atom	*x*	*y*	*z*	U(eq)
H_3	8891.17	4965.14	5246.97	25
H_4	9847.6	4132.59	6038.72	28
H_5	9842.98	3842.28	7432.46	28
H_6	8892.74	4403.06	8027.13	22
H_9	5542.5	5595	6263.09	24
H_{10}	4164.94	4971.24	5751	29
H_{11}	4220.57	3924.79	6243.77	29
H_{12}	5669.99	3470.32	7136.25	29
H_{13}	7084.53	4066.49	7577.89	23
H_{15}	7202.26	4627.37	8807.39	16
H_{16}	7998.64	4562.99	10362.48	21
H_{17}	9328.23	5184.17	11098.68	24
H_{18}	9901.61	5889.28	10295.1	22
H_{19}	9106.16	5955.98	8742.32	19
H_{21}	5329.18	5755.34	7822.24	20
H_{22}	5014.98	5043.87	8781.28	23
H_{23}	5975.67	5070.31	10327.18	26
H_{24}	7186.11	5823.31	10878.99	23
H_{25}	7449.26	6568.78	9937.77	19
H_{27}	8620.46	7026.41	8525.98	25
H_{28}	9728.2	7744.45	9455.23	31
H_{29}	9209.02	8578.64	10120.96	33
H_{30}	7590.53	8677.3	9861.51	36
H_{31}	6484.46	7915.35	8999	26
H_{33}	4904.24	6910.6	8401.76	21
H_{34}	3455.3	7443.95	7773.43	26
H_{35}	3109.11	8036.94	6458.37	22
H_{36}	4265.02	8160.51	5868.08	20
H_{39}	8014.25	7402.39	6406.66	16
H_{40}	7263.09	8438.95	5656.1	17
H_{42}	8981.23	8836.26	5957.85	24
H_{43}	10480.09	8648.28	5886.21	30

Supplementary Table 35. Hydrogen Atom Coordinates (Å×10^4) and Isotropic Displacement Parameters (Å^2×10^3) for 4[O].

Atom	*x*	*y*	*z*	U(eq)
H_{44}	10762.48	7696.29	5285.3	30
H_{45}	9546.69	6940.34	4702.91	27
H_{47}	7772.31	6628.64	4293.77	19
H_{50}	5748.31	8712.67	4245.71	19
H_{51}	4808.48	8507.8	2736.59	24
H_{52}	5149.07	7647.3	2015.08	26
H_{53}	6414.09	6954.61	2792.14	23
H_{54}	6556.22	6752.72	5018.48	16
H_{55A}	6686.47	5896.48	2333.9	121
H_{55B}	7795.53	5715.34	2725.02	121
H_{55C}	7130.44	5477.98	3223.31	121
H_{57A}	8727.87	4680.97	2376.19	108
H_{57B}	8881.46	4148.75	3122.6	108
H_{57C}	8733.27	4873.63	3332.04	108
H_{58A}	6465.77	3878.52	1578.88	58
H_{58B}	7493.82	3549.27	1992.98	58
H_{58C}	7294.9	4102.51	1267.62	58
H_{59A}	7409.87	4554.08	3762.54	78
H_{59B}	7519.87	3827.26	3534.29	78
H_{59C}	6511.58	4176.59	3074.82	78

Refinement model description

Number of restraints - 0, number of constraints - unknown.

Details:

1. Fixed Uiso

At 1.2 times of:

All C(H) groups

At 1.5 times of:

All C(H,H,H) groups

2.a Ternary CH refined with riding coordinates:

C39(H39), C40(H40), C47(H47), C54(H54)

2.b Aromatic/amide H refined with riding coordinates:

C3(H3), C4(H4), C5(H5), C6(H6), C9(H9), C10(H10), C11(H11), C12(H12), C13(H13), C15(H15), C16(H16), C17(H17), C18(H18), C19(H19), C21(H21), C22(H22), C23(H23), C24(H24), C25(H25), C27(H27), C28(H28), C29(H29), C30(H30), C31(H31), C33(H33), C34(H34), C35(H35), C36(H36), C42(H42), C43(H43), C44(H44), C45(H45), C50(H50), C51(H51), C52(H52), C53(H53)

2.c Idealised Me refined as rotating group:

C55(H55A,H55B,H55C), C57(H57A,H57B,H57C), C58(H58A,H58B,H58C), C59(H59A,H59B,H59C)

(*S*)-^{iPr}PHOX-Pd-MAH (6)

Single crystals of $C_{28}H_{26}NO_4PPd$ (**6**) were selected using a MitEGen loop and paratone oil. A suitable crystal was selected and run on a Bruker APEX-II CCD diffractometer. The crystal was kept at 100.02 K during data collection. Using Olex2[22], the structure was solved with the olex2.solve[23] structure solution program using Charge Flipping and refined with the XL[24] refinement package using Least Squares minimisation. SQUEEZE[25] was used on a solvent accessible void containing a co-crystallized hexane molecule due to it being highly disordered.

Crystal structure determination of 6

Crystal Data for $C_{28}H_{26}NO_4PPd$ (M =577.87 g/mol): monoclinic, space group I2 (no. 5), a = 14.747(9) Å, b = 10.539(6) Å, c = 18.153(16) Å, β = 97.830(8)°, V = 2795(3) Å^3, Z = 24, T = 100.02 K, μ(MoKα) = 0.752 mm^{-1}, *Dcalc* = 1.373 g/cm^3, 13250 reflections measured (3.344° ≤ 2Θ ≤ 52.58°), 4943 unique (R_{int} = 0.0807, R_{sigma} = 0.1023) which were used in all calculations. The final R_1 was 0.0789 (I > 2σ(I)) and wR_2 was 0.2078 (all data).

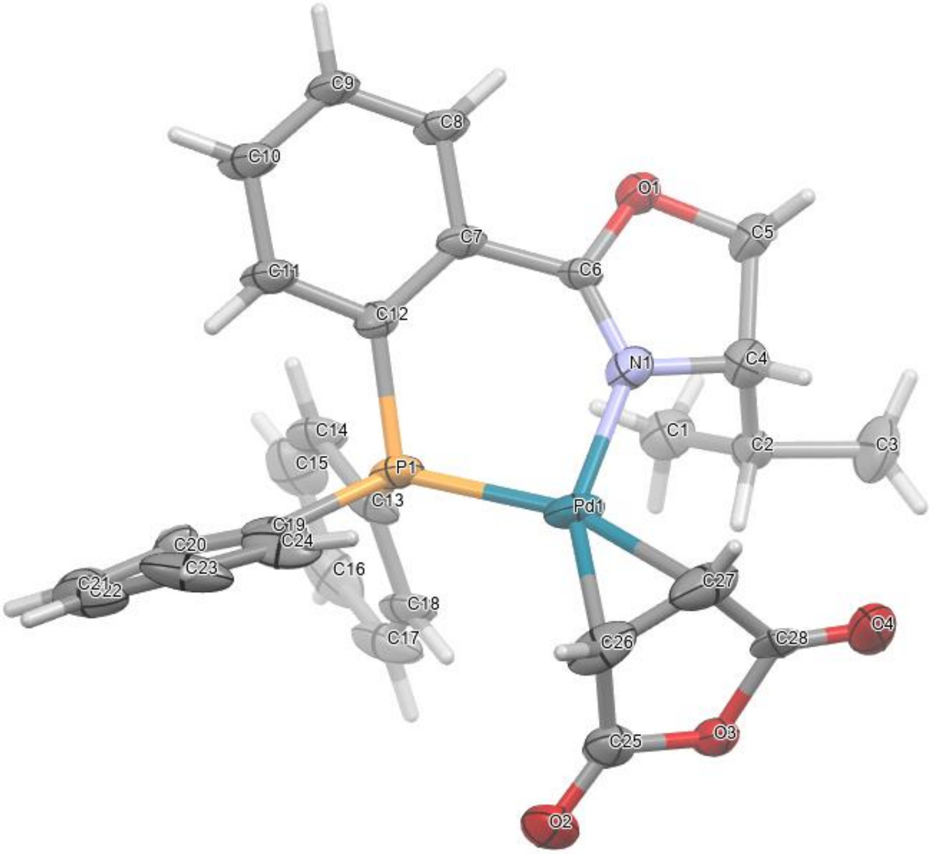

Supplementary Figure 127: Solid state molecular structure of **6** with thermal ellipsoids plotted at 50% probability. Hydrogen atoms are shown as capped sticks. Image generated using Mercury 2021.3.0. CCDC deposition number 2258957.

Supplementary Table 36. Crystal data and structure refinement for 6.

Identification code	**6**
Empirical formula	$C_{28}H_{26}NO_4PPd$
Formula weight	577.87
Temperature/K	100.02
Crystal system	monoclinic
Space group	I2
a/Å	14.747(9)
b/Å	10.539(6)
c/Å	18.153(16)
α/°	90
β/°	97.830(8)
γ/°	90
Volume/Å^3	2795(3)
Z	24
ρ_{calc}g/cm^3	1.373
μ/mm^{-1}	0.752
F(000)	1176.0
Crystal size/mm^3	0.019 × 0.006 × 0.005
Radiation	MoKα (λ = 0.71073)
2Θ range for data collection/°	3.344 to 52.58
Index ranges	-18 ≤ h ≤ 18, -13 ≤ k ≤ 11, -22 ≤ l ≤ 22
Reflections collected	13250
Independent reflections	4943 [R_{int} = 0.0807, R_{sigma} = 0.1023]
Data/restraints/parameters	4943/104/318
Goodness-of-fit on F^2	1.017
Final R indexes [I>=2σ (I)]	R_1 = 0.0789, wR_2 = 0.1879
Final R indexes [all data]	R_1 = 0.1098, wR_2 = 0.2078
Largest diff. peak/hole / e Å^{-3}	3.72/-1.11
Flack parameter	0.05(4)

Supplementary Table 37. Fractional Atomic Coordinates (×10^4) and Equivalent Isotropic Displacement Parameters (Å^2×10^3) for 6. U_{eq} is defined as 1/3 of of the trace of the orthogonalised U_{IJ} tensor.

Atom	*x*	*y*	*z*	U(eq)
Pd_1	-280.4(7)	-2.1(10)	-1625.2(5)	38.6(3)
P_1	1254(2)	114(5)	-1210.4(15)	32.9(8)
O_1	280(6)	-3960(9)	-1248(5)	34(2)
O_2	-517(9)	3025(12)	-2738(7)	57(3)
O_3	-1394(7)	1273(10)	-3057(5)	40(2)
O_4	-2384(7)	-367(12)	-3076(8)	68(4)
N_1	-145(7)	-1981(14)	-1607(5)	38(3)
C_1	520(10)	-2793(17)	-3021(9)	45(4)
C_2	-483(10)	-2537(15)	-2923(8)	35(3)
C_3	-1146(13)	-3340(20)	-3502(9)	65(5)

Supplementary Table 37. Fractional Atomic Coordinates (×10⁴) and Equivalent Isotropic Displacement Parameters (Å²×10³) for 6. U_{eq} is defined as 1/3 of of the trace of the orthogonalised U_{IJ} tensor.

Atom	*x*	*y*	*z*	U(eq)
C_4	-715(11)	-2828(16)	-2175(8)	42(3)
C_5	-460(11)	-4177(17)	-1860(9)	47(4)
C_6	369(9)	-2699(14)	-1159(7)	33(3)
C_7	1107(10)	-2311(15)	-547(7)	28(3)
C_8	1367(12)	-3248(16)	-31(8)	34(4)
C_9	2038(11)	-2953(15)	581(8)	41(4)
C_{10}	2463(12)	-1828(15)	629(7)	46(4)
C_{11}	2200(10)	-897(15)	98(7)	38(3)
C_{12}	1534(9)	-1134(14)	-519(7)	29(3)
C_{13}	2001(9)	-240(12)	-1912(6)	33(3)
C_{14}	2767(11)	-1002(14)	-1781(8)	38(3)
C_{15}	3317(13)	-1178(16)	-2313(9)	51(4)
C_{16}	3103(14)	-576(17)	-3002(9)	55(4)
C_{17}	2354(14)	176(19)	-3128(8)	56(4)
C_{18}	1802(11)	334(12)	-2589(7)	40(3)
C_{19}	1752(11)	1543(15)	-770(7)	36(3)
C_{20}	2648(10)	1881(14)	-776(7)	36(3)
C_{21}	2994(12)	2997(16)	-365(7)	44(3)
C_{22}	2439(15)	3587(16)	54(8)	57(4)
C_{23}	1562(18)	3239(19)	108(10)	60(5)
C_{24}	1182(15)	2270(19)	-334(8)	52(4)
C_{25}	-917(11)	2128(16)	-2521(8)	42(3)
C_{26}	-1050(11)	1641(17)	-1804(7)	44(3)
C_{27}	-1660(11)	600(20)	-1895(8)	54(4)
C_{28}	-1903(10)	387(14)	-2682(8)	42(3)

Supplementary Table 38. Anisotropic Displacement Parameters (Å²×10³) for 6. The Anisotropic displacement factor exponent takes the form: $-2\pi^2[h^2a^{*2}U_{11}+2hka^*b^*U_{12}+\ldots]$.

Atom	U_{11}	U_{22}	U_{33}	U_{23}	U_{13}	U_{12}
Pd_1	45.9(6)	32.4(6)	36.8(5)	15.0(6)	3.2(4)	13.6(7)
P_1	42.1(17)	23(2)	33.0(13)	10.3(18)	1.4(12)	9(2)
O_1	33(5)	31(5)	35(4)	4(4)	-5(4)	3(4)
O_2	74(8)	25(7)	66(8)	9(5)	-14(6)	-5(6)
O_3	53(6)	26(6)	37(4)	8(4)	-14(4)	-2(4)
O_4	42(6)	42(9)	108(9)	24(6)	-31(6)	-1(5)
N_1	35(7)	42(6)	33(5)	9(5)	-4(4)	2(5)
C_1	43(7)	42(10)	47(8)	5(7)	2(6)	4(7)
C_2	40(7)	20(8)	40(6)	1(5)	-13(6)	-1(6)
C_3	66(11)	69(15)	51(8)	-3(9)	-26(8)	-15(10)
C_4	35(8)	43(8)	45(6)	9(6)	-3(5)	-4(7)
C_5	38(8)	48(8)	49(8)	13(6)	-20(6)	-4(7)

Supplementary Table 38. Anisotropic Displacement Parameters (Å^2×10^3) for 6. The Anisotropic displacement factor exponent takes the form: $-2\pi^2[h^2a^{*2}U_{11}+2hka^*b^*U_{12}+\ldots]$.

Atom	U_{11}	U_{22}	U_{33}	U_{23}	U_{13}	U_{12}
C_6	27(7)	25(6)	44(6)	18(5)	-1(5)	-1(5)
C_7	31(7)	23(7)	32(6)	9(5)	4(5)	9(5)
C_8	49(9)	19(8)	33(7)	4(5)	-3(6)	12(6)
C_9	54(9)	23(7)	42(7)	11(6)	-12(6)	7(6)
C_{10}	82(12)	20(8)	30(6)	3(5)	-14(6)	4(7)
C_{11}	53(9)	24(7)	36(6)	11(5)	0(5)	9(6)
C_{12}	35(7)	29(7)	22(5)	6(5)	0(4)	-1(5)
C_{13}	48(6)	13(9)	38(5)	1(5)	11(5)	-5(5)
C_{14}	55(8)	19(8)	41(6)	5(6)	6(6)	9(6)
C_{15}	59(10)	25(10)	70(9)	-11(7)	13(7)	4(7)
C_{16}	80(11)	38(10)	51(7)	-25(6)	29(8)	-23(7)
C_{17}	107(12)	22(11)	39(6)	3(7)	14(7)	-13(8)
C_{18}	69(9)	14(9)	33(5)	5(5)	-3(5)	-2(6)
C_{19}	63(8)	25(7)	22(6)	13(5)	10(6)	9(6)
C_{20}	41(7)	26(8)	37(7)	-9(5)	-13(6)	14(5)
C_{21}	60(9)	33(9)	33(7)	3(6)	-15(6)	9(6)
C_{22}	120(13)	23(9)	27(6)	-2(6)	2(8)	5(8)
C_{23}	120(14)	24(10)	46(9)	11(6)	49(11)	19(9)
C_{24}	86(11)	45(10)	33(8)	11(6)	36(8)	8(8)
C_{25}	49(9)	27(8)	45(7)	5(5)	-7(6)	6(6)
C_{26}	62(9)	50(8)	21(5)	-5(5)	4(6)	30(6)
C_{27}	38(7)	79(11)	46(6)	32(7)	14(6)	22(6)
C_{28}	37(7)	27(9)	58(7)	23(5)	-11(6)	10(5)

Supplementary Table 39. Bond Lengths for 6.

Atom	Atom	Length/Å	Atom	Atom	Length/Å
Pd_1	P_1	2.289(3)	C_7	C_{12}	1.39(2)
Pd_1	N_1	2.095(14)	C_8	C_9	1.42(2)
Pd_1	C_{26}	2.072(16)	C_9	C_{10}	1.34(2)
Pd_1	C_{27}	2.123(15)	C_{10}	C_{11}	1.393(19)
P_1	C_{12}	1.826(14)	C_{11}	C_{12}	1.407(19)
P_1	C_{13}	1.832(13)	C_{13}	C_{14}	1.38(2)
P_1	C_{19}	1.813(17)	C_{13}	C_{18}	1.364(17)
O_1	C_5	1.465(17)	C_{14}	C_{15}	1.36(2)
O_1	C_6	1.343(17)	C_{15}	C_{16}	1.40(3)

Supplementary Table 39. Bond Lengths for 6.

Atom	Atom	Length/Å	Atom	Atom	Length/Å
O_2	C_{25}	1.21(2)	C_{16}	C_{17}	1.35(3)
O_3	C_{25}	1.438(18)	C_{17}	C_{18}	1.37(2)
O_3	C_{28}	1.427(18)	C_{19}	C_{20}	1.37(2)
O_4	C_{28}	1.228(18)	C_{19}	C_{24}	1.45(2)
N_1	C_4	1.526(19)	C_{20}	C_{21}	1.45(2)
N_1	C_6	1.281(17)	C_{21}	C_{22}	1.34(2)
C_1	C_2	1.54(2)	C_{22}	C_{23}	1.36(3)
C_2	C_3	1.58(2)	C_{23}	C_{24}	1.37(3)
C_2	C_4	1.48(2)	C_{25}	C_{26}	1.44(2)
C_4	C_5	1.56(2)	C_{26}	C_{27}	1.41(3)
C_6	C_7	1.501(19)	C_{27}	C_{28}	1.44(2)
C_7	C_8	1.38(2)			

Supplementary Table 40. Bond Angles for 6.

Atom	Atom	Atom	Angle/˚	Atom	Atom	Atom	Angle/˚
N_1	Pd_1	P_1	87.7(3)	C_{10}	C_{11}	C_{12}	121.6(15)
N_1	Pd_1	C_{27}	112.8(7)	C_7	C_{12}	P_1	124.0(10)
C_{26}	Pd_1	P_1	120.2(5)	C_7	C_{12}	C_{11}	116.8(12)
C_{26}	Pd_1	N_1	152.1(6)	C_{11}	C_{12}	P_1	119.2(11)
C_{26}	Pd_1	C_{27}	39.4(7)	C_{14}	C_{13}	P_1	123.6(10)
C_{27}	Pd_1	P_1	158.9(6)	C_{18}	C_{13}	P_1	117.4(11)
C_{12}	P_1	Pd_1	107.9(5)	C_{18}	C_{13}	C_{14}	119.0(13)
C_{12}	P_1	C_{13}	103.3(6)	C_{15}	C_{14}	C_{13}	120.8(13)
C_{13}	P_1	Pd_1	114.9(4)	C_{14}	C_{15}	C_{16}	119.3(16)
C_{19}	P_1	Pd_1	120.8(5)	C_{17}	C_{16}	C_{15}	119.7(15)
C_{19}	P_1	C_{12}	104.8(6)	C_{16}	C_{17}	C_{18}	120.2(15)
C_{19}	P_1	C_{13}	103.3(6)	C_{13}	C_{18}	C_{17}	121.0(15)
C_6	O_1	C_5	107.2(11)	C_{20}	C_{19}	P_1	123.1(11)
C_{28}	O_3	C_{25}	109.1(11)	C_{20}	C_{19}	C_{24}	119.9(16)
C_4	N_1	Pd_1	122.0(9)	C_{24}	C_{19}	P_1	116.7(14)
C_6	N_1	Pd_1	130.1(11)	C_{19}	C_{20}	C_{21}	118.7(14)
C_6	N_1	C_4	107.9(14)	C_{22}	C_{21}	C_{20}	117.9(16)
C_1	C_2	C_3	110.3(14)	C_{21}	C_{22}	C_{23}	125.0(17)
C_4	C_2	C_1	114.6(12)	C_{22}	C_{23}	C_{24}	118.4(18)
C_4	C_2	C_3	107.5(14)	C_{23}	C_{24}	C_{19}	120(2)
N_1	C_4	C_5	101.7(11)	O_2	C_{25}	O_3	119.0(14)
C_2	C_4	N_1	108.9(12)	O_2	C_{25}	C_{26}	135.0(16)
C_2	C_4	C_5	117.0(15)	C_{26}	C_{25}	O_3	105.9(14)
O_1	C_5	C_4	104.7(12)	C_{25}	C_{26}	Pd_1	107.5(10)
O_1	C_6	C_7	114.0(11)	C_{27}	C_{26}	Pd_1	72.3(10)
N_1	C_6	O_1	117.9(13)	C_{27}	C_{26}	C_{25}	109.5(13)
N_1	C_6	C_7	128.0(14)	C_{26}	C_{27}	Pd_1	68.4(8)
C_8	C_7	C_6	114.5(14)	C_{26}	C_{27}	C_{28}	107.7(13)
C_8	C_7	C_{12}	122.1(13)	C_{28}	C_{27}	Pd_1	106.4(11)

Supplementary Table 40. Bond Angles for 6.

Atom	Atom	Atom	Angle/°	Atom	Atom	Atom	Angle/°
C_{12}	C_7	C_6	123.3(12)	O_3	C_{28}	C_{27}	107.2(13)
C_7	C_8	C_9	118.4(16)	O_4	C_{28}	O_3	116.4(14)
C_{10}	C_9	C_8	121.0(14)	O_4	C_{28}	C_{27}	136.3(15)
C_9	C_{10}	C_{11}	119.7(14)				

Supplementary Table 41. Torsion Angles for 6.

A	B	C	D	Angle/°	A	B	C	D	Angle/°
Pd_1	P_1	C_{12}	C_7	33.9(14)	C_7	C_8	C_9	C_{10}	-5(3)
Pd_1	P_1	C_{12}	C_{11}	-144.0(11)	C_8	C_7	C_{12}	P_1	177.1(12)
Pd_1	P_1	C_{13}	C_{14}	-135.8(11)	C_8	C_7	C_{12}	C_{11}	-5(2)
Pd_1	P_1	C_{13}	C_{18}	47.1(12)	C_8	C_9	C_{10}	C_{11}	5(3)
Pd_1	P_1	C_{19}	C_{20}	-153.4(10)	C_9	C_{10}	C_{11}	C_{12}	-4(2)
Pd_1	P_1	C_{19}	C_{24}	33.0(12)	C_{10}	C_{11}	C_{12}	P_1	-177.7(12)
Pd_1	N_1	C_4	C_2	63.4(15)	C_{10}	C_{11}	C_{12}	C_7	4(2)
Pd_1	N_1	C_4	C_5	-172.6(10)	C_{12}	P_1	C_{13}	C_{14}	-18.5(14)
Pd_1	N_1	C_6	O_1	176.0(9)	C_{12}	P_1	C_{13}	C_{18}	164.5(11)
Pd_1	N_1	C_6	C_7	-7(2)	C_{12}	P_1	C_{19}	C_{20}	84.8(13)
Pd_1	C_{26}	C_{27}	C_{28}	-101.1(12)	C_{12}	P_1	C_{19}	C_{24}	-88.9(11)
Pd_1	C_{27}	C_{28}	O_3	-69.1(13)	C_{12}	C_7	C_8	C_9	5(2)
Pd_1	C_{27}	C_{28}	O_4	107.2(19)	C_{13}	P_1	C_{12}	C_7	-88.2(13)
P_1	C_{13}	C_{14}	C_{15}	-176.7(12)	C_{13}	P_1	C_{12}	C_{11}	93.9(13)
P_1	C_{13}	C_{18}	C_{17}	176.3(12)	C_{13}	P_1	C_{19}	C_{20}	-23.2(13)
P_1	C_{19}	C_{20}	C_{21}	-175.9(10)	C_{13}	P_1	C_{19}	C_{24}	163.1(11)
P_1	C_{19}	C_{24}	C_{23}	169.5(13)	C_{13}	C_{14}	C_{15}	C_{16}	0(2)
O_1	C_6	C_7	C_8	-20.0(19)	C_{14}	C_{13}	C_{18}	C_{17}	-1(2)
O_1	C_6	C_7	C_{12}	156.7(13)	C_{14}	C_{15}	C_{16}	C_{17}	0(3)
O_2	C_{25}	C_{26}	Pd_1	-108(2)	C_{15}	C_{16}	C_{17}	C_{18}	-1(3)
O_2	C_{25}	C_{26}	C_{27}	174.9(19)	C_{16}	C_{17}	C_{18}	C_{13}	1(2)
O_3	C_{25}	C_{26}	Pd_1	71.4(13)	C_{18}	C_{13}	C_{14}	C_{15}	0(2)
O_3	C_{25}	C_{26}	C_{27}	-5.5(17)	C_{19}	P_1	C_{12}	C_7	163.8(12)
N_1	C_4	C_5	O_1	-7.6(16)	C_{19}	P_1	C_{12}	C_{11}	-14.1(14)
N_1	C_6	C_7	C_8	162.8(15)	C_{19}	P_1	C_{13}	C_{14}	90.5(13)
N_1	C_6	C_7	C_{12}	-21(2)	C_{19}	P_1	C_{13}	C_{18}	-86.5(12)
C_1	C_2	C_4	N_1	62.0(17)	C_{19}	C_{20}	C_{21}	C_{22}	6(2)
C_1	C_2	C_4	C_5	-52.5(19)	C_{20}	C_{19}	C_{24}	C_{23}	-4(2)
C_2	C_4	C_5	O_1	110.9(14)	C_{20}	C_{21}	C_{22}	C_{23}	-2(2)
C_3	C_2	C_4	N_1	-175.0(14)	C_{21}	C_{22}	C_{23}	C_{24}	-5(3)
C_3	C_2	C_4	C_5	70.6(18)	C_{22}	C_{23}	C_{24}	C_{19}	8(3)
C_4	N_1	C_6	O_1	-3.0(17)	C_{24}	C_{19}	C_{20}	C_{21}	-2(2)
C_4	N_1	C_6	C_7	174.2(14)	C_{25}	O_3	C_{28}	O_4	176.5(12)
C_5	O_1	C_6	N_1	-2.4(18)	C_{25}	O_3	C_{28}	C_{27}	-6.4(15)
C_5	O_1	C_6	C_7	-180.0(12)	C_{25}	C_{26}	C_{27}	Pd_1	102.8(11)
C_6	O_1	C_5	C_4	6.4(17)	C_{25}	C_{26}	C_{27}	C_{28}	1.6(18)
C_6	N_1	C_4	C_2	-117.6(14)	C_{26}	C_{27}	C_{28}	O_3	2.9(17)

Supplementary Table 41. Torsion Angles for 6.

A	B	C	D	Angle/˚	A	B	C	D	Angle/˚
C_6	N_1	C_4	C_5	6.5(15)	C_{26}	C_{27}	C_{28}	O_4	179.1(17)
C_6	C_7	C_8	C_9	-177.8(14)	C_{28}	O_3	C_{25}	O_2	-173.0(14)
C_6	C_7	C_{12}	P_1	1(2)	C_{28}	O_3	C_{25}	C_{26}	7.3(15)
C_6	C_7	C_{12}	C_{11}	178.6(14)					

Supplementary Table 42. Hydrogen Atom Coordinates (Å×10^4) and Isotropic Displacement Parameters (Å^2×10^3) for 6.

Atom	*x*	*y*	*z*	U(eq)
H_{1A}	909.9	-2568.78	-2573.17	67
H_{1B}	679.38	-2292.77	-3426.75	67
H_{1C}	595.57	-3676.22	-3126.03	67
H_2	-609.21	-1635.48	-3022.78	42
H_{3A}	-1353.57	-4071.52	-3260.23	98
H_{3B}	-824.72	-3601.06	-3901.74	98
H_{3C}	-1662.68	-2826.66	-3696.37	98
H_4	-1367.03	-2668.96	-2162.99	50
H_{5A}	-981.24	-4574.01	-1679.74	57
H_{5B}	-255.05	-4714.57	-2237.79	57
H_8	1108.72	-4053	-83.58	41
H_9	2185.34	-3551.03	954.78	49
H_{10}	2931.85	-1668.41	1014.92	55
H_{11}	2470.13	-99.64	152.77	46
H_{14}	2906.83	-1399.86	-1322.56	46
H_{15}	3832.4	-1692.62	-2220.6	61
H_{16}	3473.65	-692.11	-3372.29	66
H_{17}	2215.09	587.2	-3582.57	67
H_{18}	1282.27	839.08	-2684.47	48
H_{20}	3026.27	1403.62	-1038.8	44
H_{21}	3582.27	3296.32	-391.04	53
H_{22}	2671.79	4287.7	328.87	69
H_{23}	1228.57	3647.73	437.66	72
H_{24}	562.98	2080.2	-354.97	63
H_{26}	-1078.14	2232.99	-1391.22	53
H_{27}	-2106.11	459.74	-1549.36	65

Refinement model description

Number of restraints - 104, number of constraints - unknown.

Details:

1. Fixed Uiso

At 1.2 times of:

All C(H) groups, All C(H,H) groups

At 1.5 times of:

All C(H,H,H) groups

2. Rigid bond restraints

All non-hydrogen atoms

with sigma for 1-2 distances of 0.01 and sigma for 1-3 distances of 0.01

3. Uiso/Uaniso restraints and constraints

C19: within 1.7A with sigma of 0.04 and sigma for terminal atoms of 0.08 within 1.7A

4.a Ternary CH refined with riding coordinates:

C2(H2), C4(H4), C26(H26), C27(H27)

4.b Secondary CH2 refined with riding coordinates:

C5(H5A,H5B)

4.c Aromatic/amide H refined with riding coordinates:

C8(H8), C9(H9), C10(H10), C11(H11), C14(H14), C15(H15), C16(H16), C17(H17), C18(H18), C20(H20), C21(H21), C22(H22), C23(H23), C24(H24)

4.d Idealised Me refined as rotating group:

C1(H1A,H1B,H1C), C3(H3A,H3B,H3C)

Supplementary References

(1) (a) Huang, J.; Isaac, M.; Watt, R.; Becica, J.; Dennis, E.; Saidaminov, M. I.; Sabbers, W. A.; & Leitch, D. C. ^{DMP}DAB-Pd-MAH: a versatile Pd(0) source for precatalyst formation, reaction screening, and preparative-scale synthesis. *ACS Catal.* **11**, 5636–5646 (2021). (b) Zalesskiy, S. S. & Ananikov, V. P. $Pd_2(dba)_3$ as a precursor of soluble metal complexes and nanoparticles: determination of palladium active species for catalysis and synthesis. *Organometallics* **31**, 2302-2309 (2012).

(2) Fuchs, S., Berl, V. & Lepoittevin, J.-P. A highly stereoselective divergent synthesis of bicyclic models of photoreactive sesquiterpene lactones. *Eur. J. Org. Chem.* **2007**, 1145–1152 (2007).

(3) Trost, B. M., Van Vranken, D. L. & Bingel, C. A modular approach for ligand design for asymmetric allylic alkylations via enantioselective palladium-catalyzed ionizations. *J. Am. Chem. Soc.* **114**, 9327–9343 (1992).

(4) Koch, G.; Lloyd-Jones, G. C.; Loiseleur, O.; Pfaltz, A.; Prétôt, R.; Schaffner, S.; Schnider, P.; von Matt, P. Synthesis of chiral (phosphinoaryl)oxazolines, a versatile class of ligands for asymmetric catalysis. *Recl. Trav. Chim. Pays-Bas* **114**, 206–210 (1995).

(5) Neese, F., Wennmohs, F., Becker, U. & Riplinger, C. The ORCA quantum chemistry program package. *J. Chem. Phys.* **152**, 224108 (2020).

(6) Barone, V. & Cossi, M. Quantum calculation of molecular energies and energy gradients in solution by a conductor solvent model. *J. Phys. Chem. A* **102**, 1995–2001 (1998).

(7) Iwamoto, H., Ozawa, Y., Takenouchi, Y., Imamoto, T. & Ito, H. Backbone-modified C_2-symmetrical chiral bisphosphine TMS-QuinoxP*: asymmetric borylation of racemic allyl electrophiles. *J. Am. Chem. Soc.* **143**, 6413–6422 (2021).

(8) Betz, P. & Krueger, A. Surface modification of nanodiamond under Bingel-Hirsch conditions. *ChemPhysChem* **13**, 2578–2584 (2012).

(9) Fukuda, Y., Kondo, K. & Aoyama, T. Development of novel hemilabile SEGPHOS P–P=O Ligands. *Chem. Pharm. Bull. (Tokyo)* **55**, 955–956 (2007).

(10) An, Q., Liu, D., Shen, J., Liu, Y. & Zhang, W. The construction of chiral fused azabicycles using a Pd-catalyzed allylic substitution cascade and asymmetric desymmetrization strategy. *Org. Lett.* **19**, 238–241 (2017).

(11) Wang, Y. H., Zhu, L. L., Zhang, Y. X. & Chen, Z. Diastereoselective γ-vinyl butyrolactone synthesis via gold catalyzed cyclization of allylic acetate. *Chem. Commun.* **46**, 577–579 (2010).

(12) Xiong, H.; Chen, B.; Durand-Réville, T. F.; Joubran, C.; Alelyunas, Y. W.; Wu, D.; Huynh, H. Enantioselective synthesis and profiling of two novel diazabicyclooctanone β-lactamase inhibitors. *ACS Med. Chem. Lett.* **5**, 1143–1147 (2014).

(13) Garg, Y. & Pandey, S. K. An Enantioselective approach to functionalized amino acids: total synthesis of antiepileptic drug (*R*)-lacosamide. *J. Org. Chem.* **80**, 4201–4203 (2015).

(14) Kawatsura, M. & Hartwig, J. F. Simple, highly active palladium catalysts for ketone and malonate arylation: dissecting the importance of chelation and steric hindrance. *J Am Chem Soc* **121**, 1473–1478 (1999).

(15) Trost, B. M., Xu, J. & Schmidt, T. Palladium-catalyzed decarboxylative asymmetric allylic alkylation of enol carbonates. *J Am Chem Soc* **131**, 18343–18357 (2009).

(16) Kano, T., Hayashi, Y. & Maruoka, K. Construction of a chiral quaternary carbon center by catalytic asymmetric alkylation of 2-arylcyclohexanones under phase-transfer conditions. *J Am Chem Soc* **135**, 7134–7137 (2013).

(17) Leutenegger, U., Umbricht, G., Fahrni, C., von Matt, P. & Pfaltz, A. 5-aza-semicorrins: a new class of bidentate nitrogen ligands for enantioselective catalysis. *Tetrahedron* **48**, 2143–2156 (1992).

(18) Beaumier, F., Dupuis, M., Spino, C. & Legault, C. Y. Formal intramolecular (4 + 1)-cycloaddition of dialkoxycarbenes: control of the stereoselectivity and a mechanistic portrait. *J. Am. Chem. Soc.* **134**, 5938–5953 (2012).

(19) Fournier, J., Lozano, O., Menozzi, C., Arseniyadis, S. & Cossy, J. Palladium-catalyzed asymmetric allylic alkylation of cyclic dienol carbonates: Efficient route to enantioenriched γ-butenolides bearing an all-carbon α-quaternary stereogenic center. *Angew. Chem. Int. Ed.* **52**, 1257–1261 (2013).

(20) Aubert, S., Katsina, T. & Arseniyadis, S. A Sequential Pd-AAA/cross-metathesis/Cope rearrangement strategy for the stereoselective synthesis of chiral butenolides. *Org. Lett.* **21**, 2231–2235 (2019).

(21) Keenan, T., Jean, A. & Arseniyadis, S. Phase-transfer-catalyzed alkylation of hydantoins. *ACS Org. Inorg. Au* **2**, 312–317 (2022).

(22) Dolomanov, O. V., Bourhis, L. J., Gildea, R. J., Howard, J. a. K. & Puschmann, H. OLEX2: a complete structure solution, refinement and analysis program. *J. Appl. Crystallogr.* **42**, 339–341 (2009).

(23) Bourhis, L. J., Dolomanov, O. V., Gildea, R. J., Howard, J. A. K. & Puschmann, H. The anatomy of a comprehensive constrained, restrained refinement program for the modern computing environment - Olex2 dissected. *Acta Crystallogr. Sect. Found. Adv.* **71**, 59–75 (2015).

(24) Sheldrick, G. M. A short history of SHELX. *Acta Crystallogr. A* **64**, 112–122 (2008).

(25) Spek, A. L. PLATON SQUEEZE: a tool for the calculation of the disordered solvent contribution to the calculated structure factors. *Acta Crystallogr. Sect. C Struct. Chem.* **71**, 9–18 (2015).
